# On-Demand Photoprotection by UV-Transparent Small Molecules via Aggregation-Induced Absorption: The Origin and Modern Application

**DOI:** 10.1021/acscentsci.6c01033

**Published:** 2026-08-17

**Authors:** Bing-Yue Zhao, Yao Hui, Wan-Jun Qin, Qing Liu, Xiao Wang

**Affiliations:** † State Key Laboratory of Coordination Chemistry, 12581 School of Chemistry and Chemical Engineering, Nanjing University, Nanjing 210023, Jiangsu, China; ‡ Frontiers Science Center for Critical Earth Material Cycling, Nanjing University, Nanjing 210023, Jiangsu, China

## Abstract

A central challenge across modern chemistry, biology, materials science, and origins-of-life research is obtaining a balance between harnessing beneficial photoreactions and minimizing detrimental photodamage. Here, we have discovered an aggregation-induced photoprotection pathway provided by nonconjugated small molecules. Although conventionally classified as UV-transparent due to the lack of chromophores, these molecules are found to form supramolecular assemblies at elevated concentrations and develop emergent UV-absorbing properties, likely via slip-stacked J-aggregation, as confirmed by multiple spectroscopic characterizations. Density functional theory calculations further elucidated the mechanism of the observed concentration-dependent absorption. A predictive structure–property relationship has been proposed. Regulated by concentration, this effect can smartly allow necessary photoreactions while shielding critical biomolecules from excessive UV radiation. It also suggests that self-photoprotection of the aggregatable molecules could have enabled their prebiotic survival and enduring presence under harsh UV conditions. Furthermore, these findings prompted us to develop a convenient and noninvasive photoprotection strategy for contemporary plastic materials using inexpensive and environmentally friendly small agents.

## Graphical abstract

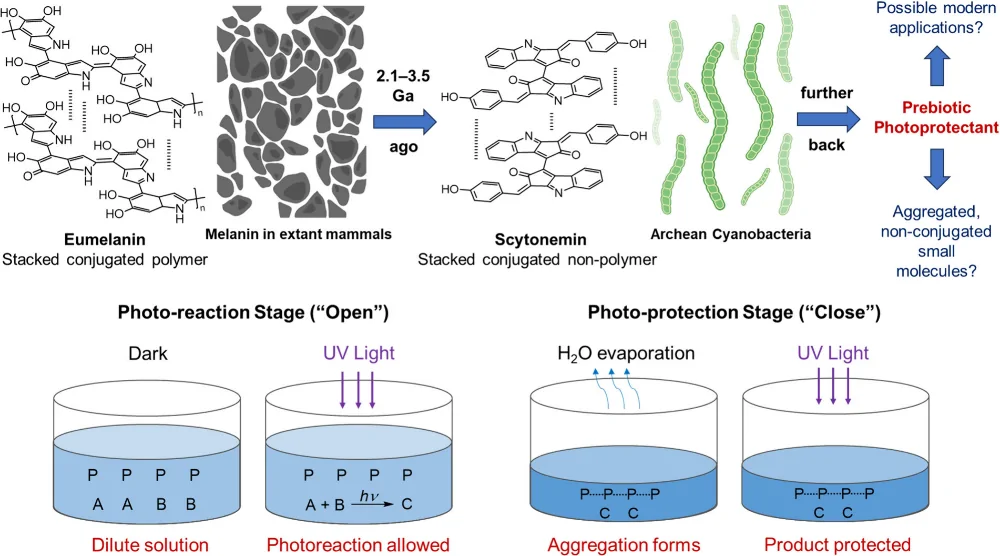

## Introduction

Ultraviolet (UV) light presents a fundamental paradox in chemistry: it is a potent driver of synthesis^1^ yet a potent agent of degradation.^2^ Chemists sought to mitigate its destructive side effects by developing photoprotectants.^3,4^ Living organisms use substances such as melanin^5^ and glutathione^6^ as a light filter. Although eumelanin (a major type of melanin) has a broadband UV absorption, the core structure—covalently bonded 5,6-di­hydroxy­indole-2-carb­oxylic acid (DHICA)—and its quinone form have relatively narrow and limited absorbance,^7^ until it polymerizes and forms a stacked aggregation (Figure 1A). The UV-shielding ability of melanin that derives from aggregation-modulated chromophore interactions has been confirmed by both experimental^8^ and computational studies.^9^ In materials science, interactions such as J-aggregation of functional dye molecules result in a similar observation.^10^ Such molecular aggregation is reported to reduce the HOMO–LUMO energy gap through intermolecular orbital interactions,^11^ resulting in a bathochromic shift or a newly emerged band in the UV–vis spectrum. Such a phenomenon, known as aggregation-induced absorption (AIA), has recently been exploited in application fields such as organic solar cells^12^ and photoacoustic imaging.^13^ AIA has seen a few recent examples in organic synthesis as well,^14,15^ although not aimed for photoprotection but for activating reaction substrates. However, to the best of our knowledge, previous studies on AIA and J-aggregation have been largely confined to compounds that are intrinsically UV-responsive (such as dyes or other π-conjugated substances), whereas examples with nonabsorbing molecules without a UV-chromophore are exceptionally scarce. Bearing a similar acronym, aggregation-induced emission (AIE) has emerged as a landmark technology in recent years.^16^ However, unlike AIA, its research centers on radiative decay pathways (emission) of aromatic^17^ or nonaromatic^18^ compounds, whereas the accompanying variations in absorption spectra are often subtle and remain secondary to its core focus.

**Figure 1:**
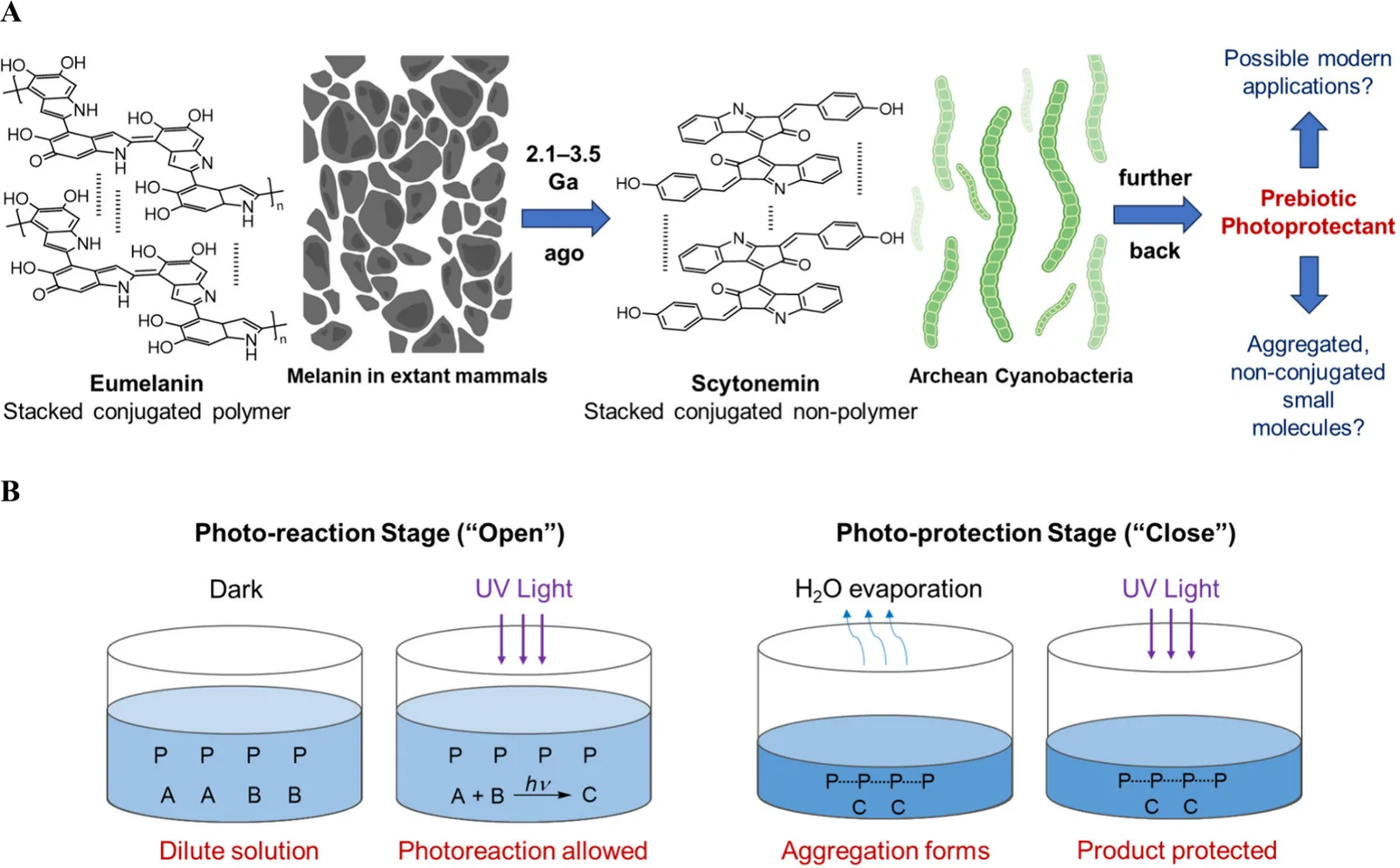
Overview of a desired UV protectant with aggregation and autoregulation capabilities. (A) Potential evolutionary connection between photoprotectant in modern organisms and its ancestral analogues. (B) Illustration of the desired open-close mechanism for photoreaction and photoprotection (“A” and “B”, reactants; “C”, product; “P”, protectant).

The imperative need for UV protection arose with the first life forms, or even predates life itself. UV light was essential to many prebiotically relevant molecules on early Earth.^19^ It facilitated processes such as the photosynthesis of nucleobases,^20^ nucleosides,^21,22^ and amino acids,^23^ the photoreduction of carbon dioxide,^24^ and the photoproduction of ammonia.^25^ However, UV radiation is also destructive to these products, thus hindering their accumulation and polymerization,^26^ not to mention that the primordial irradiation was far more intensive and broadband than nowadays.^27^ For example, UVC (ultraviolet C, 100–280 nm) light can induce the dimerization of pyrimidine nucleobases.^28^ Azole derivatives, which are critical building blocks in prebiotic synthesis, undergo rapid decomposition in the 215–255 nm range.^29^ The mechanism by which primitive biomolecules achieved UV protection during the origins of life remains incompletely understood. The early Earth’s atmosphere was able to shield UVC radiation below 204 nm but not any longer wavelengths.^30^ Ponnamperuma reported the UV-shielding function of a coacervate consisting of a short peptide.^31^ Miller believed that prebiotic polymers, along with several inorganic compounds (such as Fe­(II) salts) in the primitive ocean, protected organic compounds from UV degradation.^32^ Clay minerals are reported to provide shelter for RNA-like molecules from UV radiation.^33^ Sasselov’s team reported that modest concentrations of specific nucleosides can be UV-shielding for photosensitive molecules.^34^ More recently, Todd’s team studied UV transmittance through solutions of various organic solutes.^35^ Adenine, the genetic nucleobase precursor to adenosine and 2′-deoxyadenosine, exhibits strong UV absorption at 250 nm in aqueous solution. However, most organic feedstocks investigated in their report absorb light of short wavelengths (∼200 nm), with decreasing absorbance at longer wavelengths (>200 nm).^35^ Therefore, developing effective protection strategies against irradiation of the upper UVC region (204–280 nm) warrants more extensive investigation, as it is usually the most damaging wavelength range to RNA^36^ and protein.^37^

Some of the previously proposed conditions require compounds with aromatic chromophores, or macromolecules, or other substances with higher complexity than simple feedstock, whose formation might demand UV protection as well. This raises the possibility that before such complex photoprotectants could emerge, earlier stages of chemical evolution likely relied on simpler, smaller protective molecules. However, given their structural simplicity and the lack of chromophores, the desired protectants are unlikely to be UV-absorbing between 200 and 280 nm. In a prebiotic context, might there have existed molecules that were UV-transparent in a monomeric state but that became UV-absorbent upon aggregation—an analogue to the protective function of eumelanin in later evolution? We believe this concept is not merely speculative, as it can find a tangible analogue in scytonemin,^38^ a natural sunscreen pigment in cyanobacteria whose origins trace back 2.1 to 3.5 billion years (Figure 1A). While a direct evolutionary link to eumelanin may be distant, scytonemin demonstrates that the principle of aggregation-induced photoprotection was an ancient and viable strategy.^38^ The contrast between eumelanin (stacked π-conjugated polymer) for higher animals and scytonemin (stacked π-conjugated nonpolymer) for single cell organisms suggests that smaller molecules, perhaps as simple as an unconjugated nonpolymer, could have served as primordial UV protectants (Figure 1A).

On the other hand, the delicate balance between UV-driven synthesis and degradation remains a key consideration in understanding how life’s molecular precursors persisted under early Earth conditions. It is dilemmatic that complete shielding from UV light impedes the needed photoprocess, while leaving the products unshielded leads to photodegradation. It would be ideal if the UV protectant had an open-close mechanism as depicted in Figure 1B. At low concentrations, UV light penetrates the solution of the protectant (“P”) to drive photochemical reactions. As the solvent (most likely water) evaporates, the concentration of the protectant reaches a threshold at which it undergoes intermolecular interactions (e.g., self-assembly into aggregates) that are UV-absorbing. This process could have been synchronous with the evaporative concentration of the photoproduct (protectee) generated during the earlier dilute stage. The photoproducts become most vulnerable after significant water loss because (1) the higher substrate concentration accelerates photodegradation and (2) the shallower water layer results in stronger UV irradiation according to the Lambert–Beer law. At this critical point, the activated protectant steps in to provide photoprotection. In contrast, deep water is self-protective, as effective irradiation only covers several meters below the water surface.^39^ In brief, this desired protectant should be UV-inactive in dilute solution but UV-active when concentrated—this again exemplifies a probable case of aggregation-induced absorption. Given that prebiotic environments (e.g., tidal pools, hydrothermal vents, or mineral surfaces) likely experienced fluctuating solute concentrations due to evaporation, wet–dry cycles, or temperature gradients, dynamic aggregation could have been a common feature of early organic systems.^40^ However, to the best of our knowledge, such prebiotic photoprotection through aggregation-induced absorption has not been explored. Here, we report our recent study on this problem and its potential application in protecting modern polymeric materials.

## Results and Discussion

### Unusual UV Response of a UV-Transparent Compound

Before initiating a systematic screen of relevant small molecules for a photoprotectant described above, we had no prior indication of what a viable candidate might look like. However, it came to our attention by chance that diamidophosphate (DAP),^41^ a versatile substance for prebiotic phosphorylation^42^ and vesicle formation,^43^ displayed some abnormal UV responses at high concentrations. Following the established understanding of most nonaromatic phosphorus­(V) compounds, DAP is not expected to absorb UV light at 254 nm. The UV–vis absorption of BDMDAP (bis­(dimethyl­amido)­phos­phate),^44^ the *N*-methylated derivative of DAP, showed a similar concentration-dependent pattern. This serendipitous observation prompted a systematic investigation into their inherent photophysical behavior, as we suspected that an equilibrium exists between free phosphoramidate molecules and diverse higher order aggregates.

We set out to explore their UV absorption behavior as a function of concentration in more detail. At high concentrations, the absorbance (*A*) may deviate from the Lambert–Beer law, and an *A* value above 2.0 is traditionally considered underestimated and unsuitable for the spectrophotometric method. Using an ultrashort path length cell could reduce the high absorbance back to the linear response range, but the data at low to medium concentrations may fall outside the linear range as well (*A* < 0.1). Providentially, the focus of this study is exactly the unusual light-absorbing behavior itself under concentrated conditions (e.g., after wet–dry cycling), where a small quantity of a compound is enriched. In addition, the PerkinElmer LAMBDA 750S spectrometer used in this study is designed to achieve highly accurate measurements up to an *A* value of 6.0. Therefore, the absorbance reading should still be informative, provided that it is primarily contributed by the intrinsic light-absorbing ability of the analyte and no other factors are interfering. The most crucial step is to rule out the interference from light scattering on the absorbance measurements. Thus, before moving on, a concentrated but clear solution of DAP (0.01 M) was prepared and centrifuged at 8000 rpm for 10 min (see the Supporting Information). The UV spectrum of the supernatant was almost identical to the one obtained before centrifugation (Figure S44), indicating the absence of any macro-scale precipitates or flocs that would cause intense light scattering and inflated absorbance. Having verified these prerequisites, we carried on with the UV–vis spectroscopy of phosphoramidates. The UV spectra of DAP exhibited distinct characteristics, showing an overall low absorbance under dilute conditions (0.001–0.009 M) (Figure 2A). At 0.01 M, a large new absorption band emerged abruptly at around 235 nm (for clarity, see Figure S14 in the Supporting Information for the individual spectrum at each concentration). A second new absorption band was observed at 287 nm, whose intensity increased sharply when the concentration was above 0.02 M, suggesting the emergence of higher order aggregates. This spectral behavior agreed well with the aggregation pattern observed in mass spectrometry (MS) data (see Figure 3A). The emergence of the long-wave secondary band (∼287 nm) indicates that aggregated DAP might be able to attenuate broader band UV than its monomer. The UV absorption spectra of BDMDAP were recorded across increasing concentrations (0.005–0.36 M) (Figure 2B and Figure S17). Starting from 0.02 M, a sharp absorption bands emerged at 228 nm and grew larger with increasing concentration. The critical aggregation concentration (CAC) was determined by monitoring the UV extinction coefficient as a function of concentration (Figure 2C,D for DAP and Figure 2E for BDMDAP). A narrow concentration range was identified across which a distinct, abrupt transition in the extinction coefficient occurred. The measured extinction coefficients at concentrations immediately below and above this threshold clearly demonstrated a discontinuous increase, consistent with cooperative self-assembly. This sharp transition defines the CAC interval for each compound, providing quantitative evidence for concentration-triggered aggregation and the onset of the aggregation-induced absorption (AIA) effect. The saturated regions are outside the reliable quantitative range and were generally excluded from the calculation of CAC.

**Figure 2:**
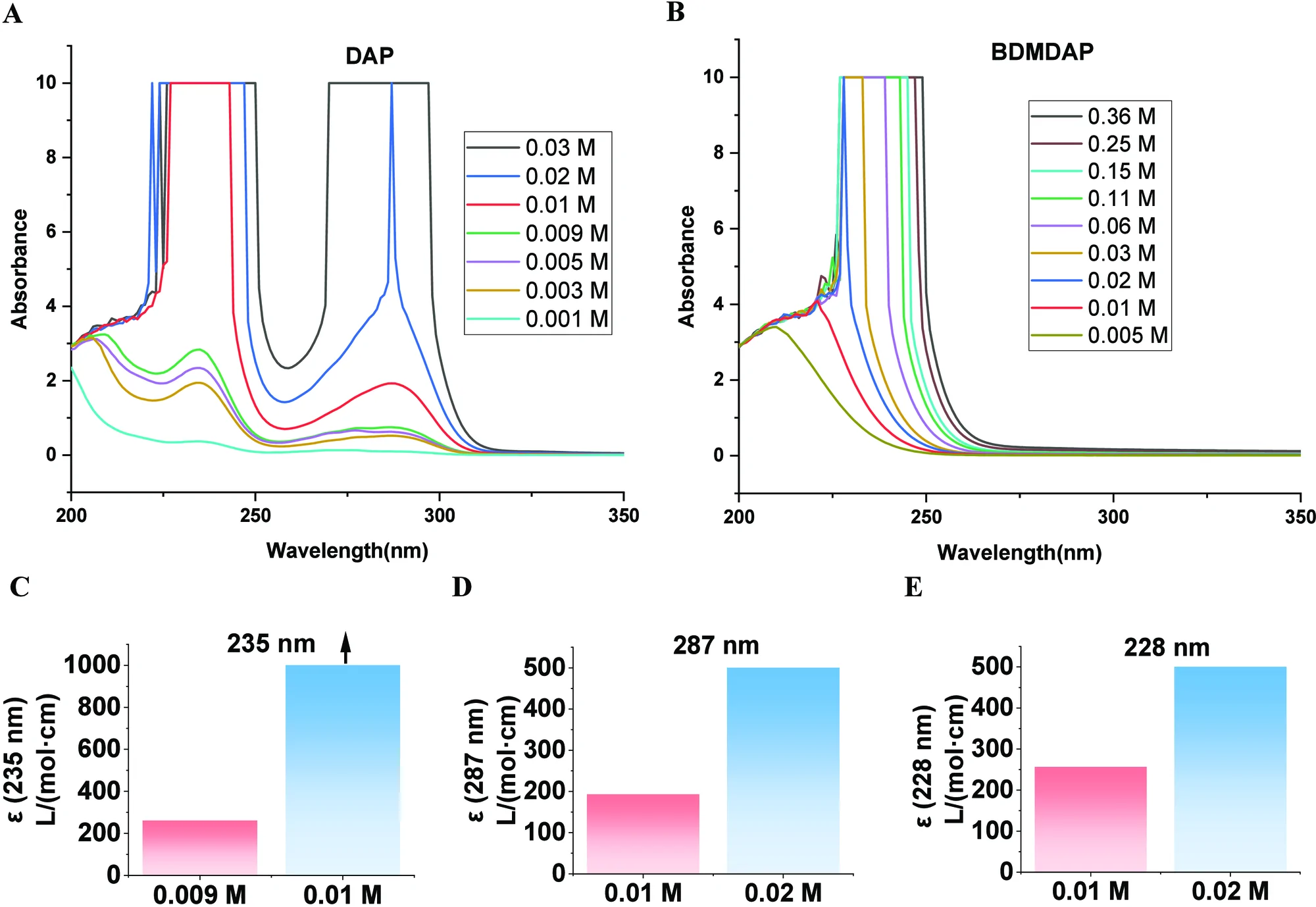
Analysis of the unusual UV absorption and aggregation of phosphoramidates. (A) UV spectra of DAP at different concentrations. (B) UV spectra of BDMDAP at different concentrations. (C) Change of extinction coefficient at 235 nm near the first critical aggregation concentration (CAC) of DAP, showing a pronounced increase from 0.009 to 0.01 M (the up arrow indicates that the actual value may be higher, as the absorbance is near or above the detection limit). (D) Change of extinction coefficient at 287 nm near the second CAC of DAP, showing a pronounced increase from 0.01 to 0.02 M. (E) Change of extinction coefficient at 228 nm near the CAC of BDMDAP, showing a pronounced increase from 0.01 to 0.02 M.

**Figure 3:**
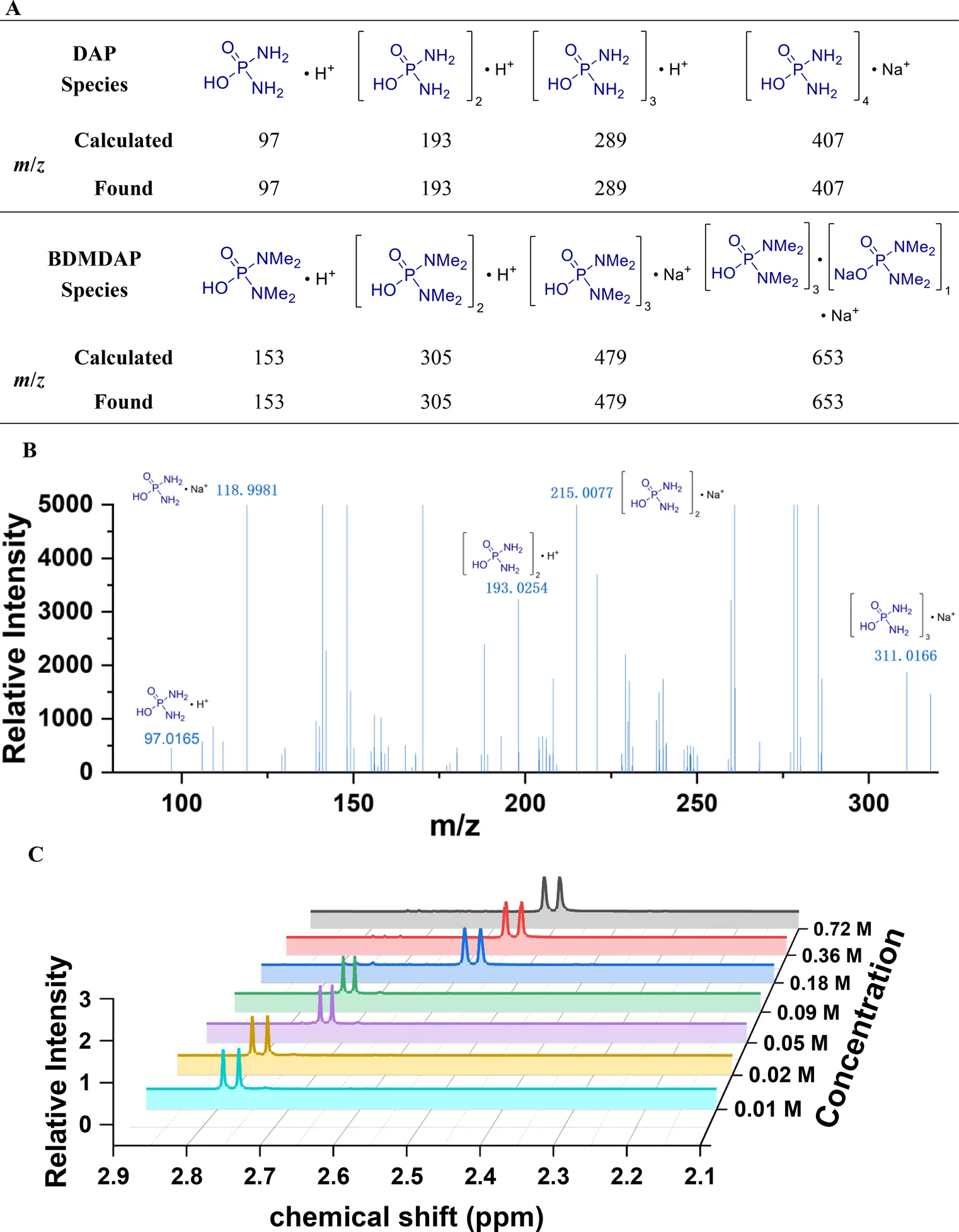
Further analysis of the aggregation of phosphoramidates. (A) Masses (low-resolution) found for the phosphoramidate aggregates. (B) High-resolution mass spectrum of DAP with structures of representative aggregates. (C) Change of ^1^H NMR signals of BDMDAP with its concentration (spectra recorded on a 400 MHz spectrometer; solvent: D_2_O).

The formation of phosphoramidate aggregates was further validated by mass spectrometry (MS) analysis. For both DAP and BDMDAP, mass spectrometry revealed periodic signal series with characteristic *m*/*z* intervals (+96 and +152, respectively; see Figures S2 and S9 in the Supporting Information for the MS spectra), strongly suggesting the formation of molecular aggregates. The formulas of the detected aggregates are listed in Figure 3A. Despite employing a low injection volume (0.1 μL) for MS analysis, DAP consistently exhibited aggregation, with spectral data revealing dimer (*m*/*z* 193), trimer (*m*/*z* 289), and tetramer (*m*/*z* 407) species. Such aggregates were also detected in the MS analysis of BDMDAP. For both phosphoramidates, masses of sodium-adducted aggregates were detected alongside protonated species (Figure S2 and S9), suggesting that the aggregation mechanism exhibits versatile cation dependence rather than requiring specifically acidic environments. Figure 3B presents the high-resolution mass spectrum (HRMS) of DAP, with the masses of representative aggregates assigned. The zoomed-in MS spectra of both DAP and BDMDAP, both low- and high-resolution ones, are provided in the Supporting Information. MS data cannot be used to determine the actual population distribution of aggregates in solution due to the ionization conditions. Instead, they are presented as supportive evidence that aggregate-related ionic species can be detected under the measurement conditions.^45^ BDMDAP retains the capability to mediate the cyclophosphorylation of nucleosides in aqueous media while offering enhanced steric and electronic properties.^46^ Its prebiotic relevance may be conditional upon the probable existence of alkylammonium species in primordial environments.^47^ Thus, BDMDAP was mainly studied as a model compound due to the structural advantage that its methyl groups provide distinct ^1^H NMR signals which are absent in DAP, thereby facilitating direct observation of the aggregate’s electronic environment. The concentration-dependent behavior of BDMDAP in D_2_O was then investigated by ^1^H NMR, which is diagnostic for monitoring aggregation.^48^ From 0.01 to 0.02 M, the chemical shift of the methyl protons (−N­(CH_3_)_2_) remained largely constant (Figure 3C). From 0.02 to 0.05 M, a notable upfield shift of the methyl signals was observed (Δδ ≈ −0.06 ppm) and continued with increasing concentration (from 0.09 to 0.18 M, Δδ ≈ −0.13 ppm; from 0.18 to 0.36 M, Δδ ≈ −0.04 ppm; and from 0.36 to 0.72 M, Δδ ≈ −0.03 ppm). The observed upfield shift indicated enhanced magnetic shielding around the methyl groups, likely due to aggregation at higher concentrations. These interactions would constrain the conformational freedom of the −N­(CH_3_)_2_ groups, placing the methyl protons in a more shielded microenvironment. Furthermore, the NMR analysis ruled out the formation of polymeric species through dehydrative condensation, as evidenced by the well-resolved and unaltered signal pattern of the −CH_3_ protons.

### More Prebiotic Molecules with Aggregation-Induced Absorption

Beyond phosphorus compounds, these data intrigued us to examine whether other life-related primordial substances have similar characteristics of concentration-dependent absorption to block upper UVC radiation. The UV spectra of these compounds were recorded at varying concentrations (Figure 4A–F), with a narrow CAC range shown in the bar charts, which was determined by progressively narrowing the concentration interval based on the most evident spectral change. A diagnostic AIA-responsive wavelength was selected for each compound, and additional concentrations were measured near the transition. Most of the CAC ranges reported here are the first detectable onset of AIA aggregation (the first CAC range). Above that, aggregation may further evolve without forming a single defined structure. Cysteine, although conventionally not thought to be derived from prebiotic chemistry, has been proven to be synthetically relevant to prebiotic peptides and available as a potential catalyst.^49^ Below 0.025 M, the UV spectra of cysteine exhibited a weak bathochromic shift of the main absorption band (Figure 4A). However, at 0.05 M, multiple narrow absorption bands abruptly arose between 220 and 230 nm, which grew rapidly with concentration (see Figure S20 in the Supporting Information for the UV spectrum at each concentration). Lysine, although traditionally considered more synthetically challenging than simpler amino acids, displayed a drastic concentration-related UV response (Figure 4B and Figure S23). At 0.06 M, multiple new absorption bands sharply appeared between 220 and 230 nm. From 0.10 to 0.12 M, the absorbance between 220 and 320 nm rose rapidly. Above 0.6 M, a group of strong new bands centered at 350 nm emerged abruptly. These absorption bands extended to approximately 380 nm (and further). Therefore, lysine seems to be a compound whose absorption potentially covers the entire UVA, UVB, and UVC regions. Bearing a remote basic group like lysine, arginine displayed an obvious concentration-dependent UV response, with a sharp new band emerging at 222 nm at 0.1 M and a second new band at 230 nm at 0.3 M (Figure 4C and Figure S25). The direct role of lactic acid in the origins of life remains an active area of research.^50^ Below 0.1 M, the main UV absorption peak gradually shifted toward longer wavelengths. A narrow new band emerged at 230 nm starting from 0.15 M, which grew larger with concentration and exhibited a continued redshift (Figure 4D and Figure S27). Citric acid, the central substance of the Krebs cycle,^51^ demonstrated a small redshift at low concentrations (200 nm at 0.003 M, 210 nm at 0.01 M, and 223 nm at 0.05 M). Then at 0.06 M, the sudden appearance of a sharp band at 228 nm was observed (Figure 4E and Figure S29). Oxalic acid, a key compound in the glyoxylate cycle, like lactic acid,^52^ has been found in interstellar ice analogues,^53^ despite the scarcity of direct evidence for abiogenesis. Nevertheless, it exhibited obvious AIA capacity at a fairly low concentration (0.02 M), with the emergence of multiple new bands around 225 nm (Figure 4F and Figure S31). In summary, the abrupt emergence of sharp band(s) at longer wavelengths at the CAC in these examples suggests that such an AIA effect could be prevalent among many common prebiotic substances. It is noteworthy that some of the investigated compounds such as cysteine can be formed via photoprocesses.^54^ Due to their potential UV-shielding ability, rapid degradation of these compounds under prolonged irradiation may be suppressed (see Figure 10A for a consumption test). Such self-protection could have contributed to the prebiotic selection and persistence of these molecules.

**Figure 4:**
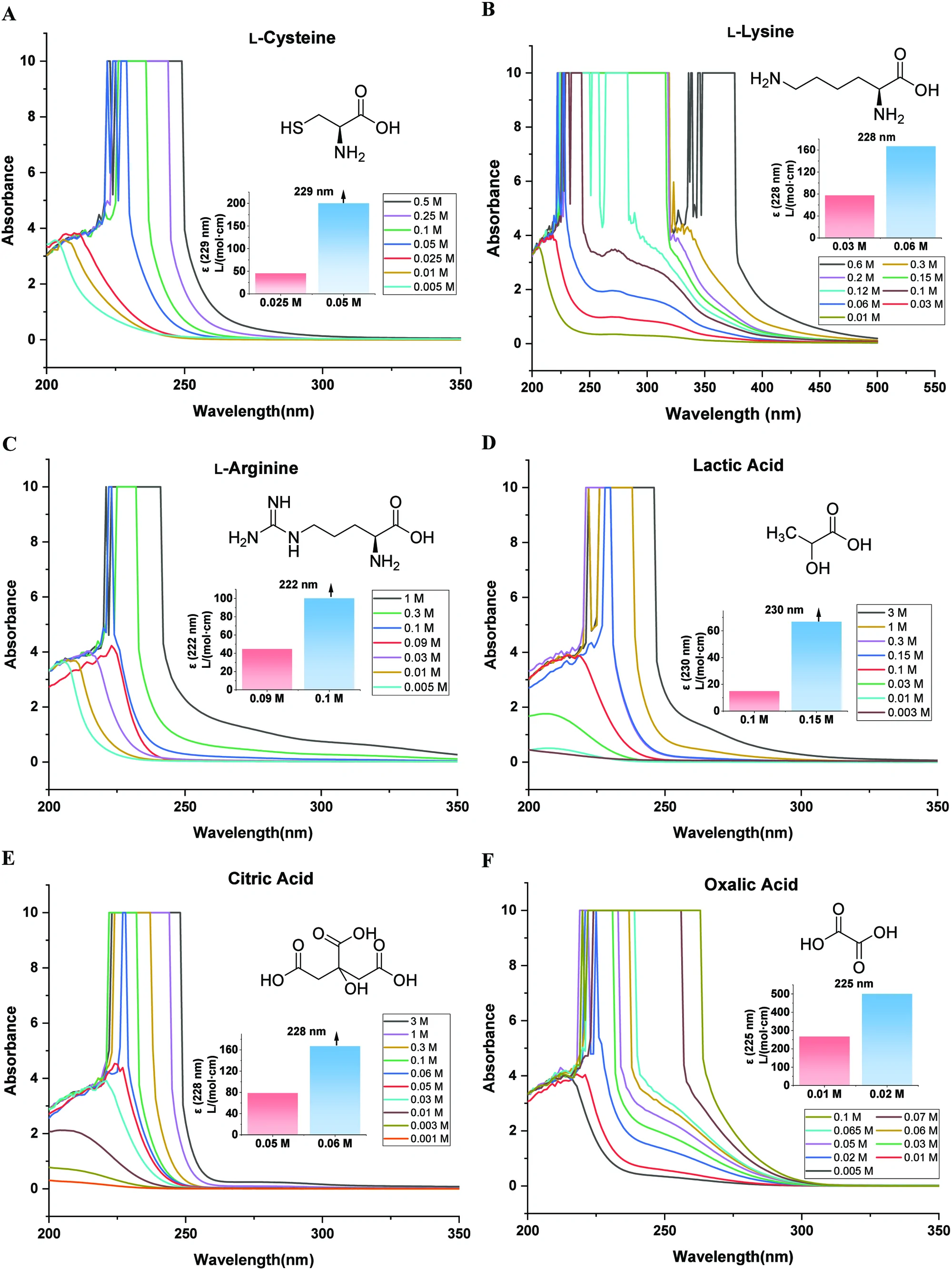
UV spectra of other key compounds at varying concentrations and the change of extinction coefficient near their first CAC: (A) l-cysteine, (B) l-lysine, (C) l-arginine, (D) dl-lactic acid, (E) citric acid, and (F) oxalic acid.

Other popular prebiotic compounds, especially amino acids and several key C2 and C3 molecules, were also examined (see the Supporting Information). The UV spectra of glycine, alanine, serine, glutamic acid, and histidine were recorded at different concentrations. However, their AIA effect was generally not as significant as that of the compounds in Figure 4. Because of the limited solubility of glutamic acid, the tested concentrations could not exceed 0.05 M (Figure S37). Among the tested amino acids, glycine had the weakest AIA (Figure S40). Notably, besides amino acids, we also evaluated the potential AIA capability of several short peptides mostly consisting of nonaromatic amino acids (Cys-Cys, acetyl tetrapeptide-11, and hexapeptide-9), which showed emergent UV absorption bands at longer wavelengths as concentration increased (see the Supporting Information). For glycolaldehyde and glyceraldehyde, no concentration-dependent absorption was detected (Figures S41 and S42). Nevertheless, the absorption around 250 nm at higher concentrations of glycolaldehyde might offer weak protection for sugar products from the Formose reaction, where it serves as an intermediate as well as a critical catalyst.^55^ Finally, we extended the investigation to inorganic phosphate.^56^ The UV spectra of NH_4_H_2_PO_4_ at different levels of concentrations were recorded (Figure S43). However, its overall absorbance was negligible compared to that of the aggregatable organic compounds.

### Unraveling the Structure of Aggregates and the Mechanism of AIA

For nonconjugated molecules, the understanding of their aggregation models has been a challenge.^57^ To gain insight into the aggregate formation of phosphoramidate, dynamic light scattering (DLS) measurements were first performed on an aqueous DAP solution (0.01 M; filtered with a 0.22 μm syringe filter), which revealed the presence of large-scale aggregates with an effective diameter of approximately 933 nm (Figure S59 in the Supporting Information). These unusual data, together with the UV and MS analyses, strongly indicate that DAP molecules undergo extensive supramolecular self-assembly in aqueous media. Unlike conventional precipitates or large aggregates that cause severe light scattering and distort UV measurements, our system forms highly hydrated, stable nano/submicron colloidal assemblies. The DLS analysis of lysine (0.6 M; filtered with a 0.22 μm syringe filter) was also performed, which showed an aggregation with an effective diameter of 144 nm (Figure S60).

In our UV–vis experiments, the emergence of sharp and intense new band(s) at longer wavelengths upon concentration increasing served as a characteristic signature of J-aggregates.^12^ The proposed aggregation mechanism was further established by a combination of direct imaging and spectroscopic techniques. First, to gain insight into the molecular packing responsible for the aggregation, single crystal X-ray diffraction data of BDMDAP (as sodium salt)^44^ were obtained (Figure 5A,B; DAP failed to form crystals). The structure revealed a slip-stacked arrangement of adjacent molecules, with a slip angle of 12.5°. This head-to-tail geometry is characteristic of J-aggregates, aligning the transition dipoles in a manner that favors exciton coupling and the rise of a narrow longer-wavelength absorption band. Besides this alignment, regularly ordered interactions of BDMDAP with the surrounding hydrated sodium ions was also observed. Although this structure was obtained from the solid state, it is highly suggestive of the packing mode in concentrated solution that drives the observed AIA effect. Second, cryogenic transmission electron microscopy (Cryo-TEM) of a DAP solution (0.03 M) directly visualized the hierarchical self-assembly, revealing straight, elongated fibrillar bundles with dimensions of approximately 100–500 nm in width and 600–5000 nm in length (Figure 5C,D). This is consistent with (1) the one-dimensional, anisotropic morphology expected for aggregates such as J-type ones, as opposed to spherical or amorphous clusters, and (2) the hydrodynamic diameter (ca. 900 nm) obtained from DLS. Next, circular dichroism (CD) spectroscopy of key chiral AIA compounds (l-lysine, d-lysine, l-cysteine, and l-lactic acid) revealed bisignate Cotton effects characteristic of exciton coupling (for l-lysine, see Figure 5E; for others, see the Supporting Information). The concentration-dependent CD spectra of l-lysine showed a bisignate Cotton effect that emerged above a threshold concentration and grew in intensity with a further increase (Figure 5E). The opposite signs observed for l-lysine and d-lysine provided evidence that the optical activity originates from chiral supramolecular packing rather than nonspecific aggregation or instrumental artifacts (Figure S47). Finally, variable-temperature UV–vis spectroscopy revealed the thermotropic behavior of the aggregates (see the Supporting Information). The sharp J-band was reversible upon heating and cooling. At elevated temperatures, the peak broadened, indicating a transition from an ordered aggregate to a more disordered, heterogeneous ensemble of oligomers. The restoration of J-band upon cooling ruled out irreversible nonspecific clustering and confirmed that the aggregation is under thermodynamic control. These data collectively provided an orthogonal and compelling set of evidence for a dynamic, yet well-defined, J-aggregation mechanism.

**Figure 5:**
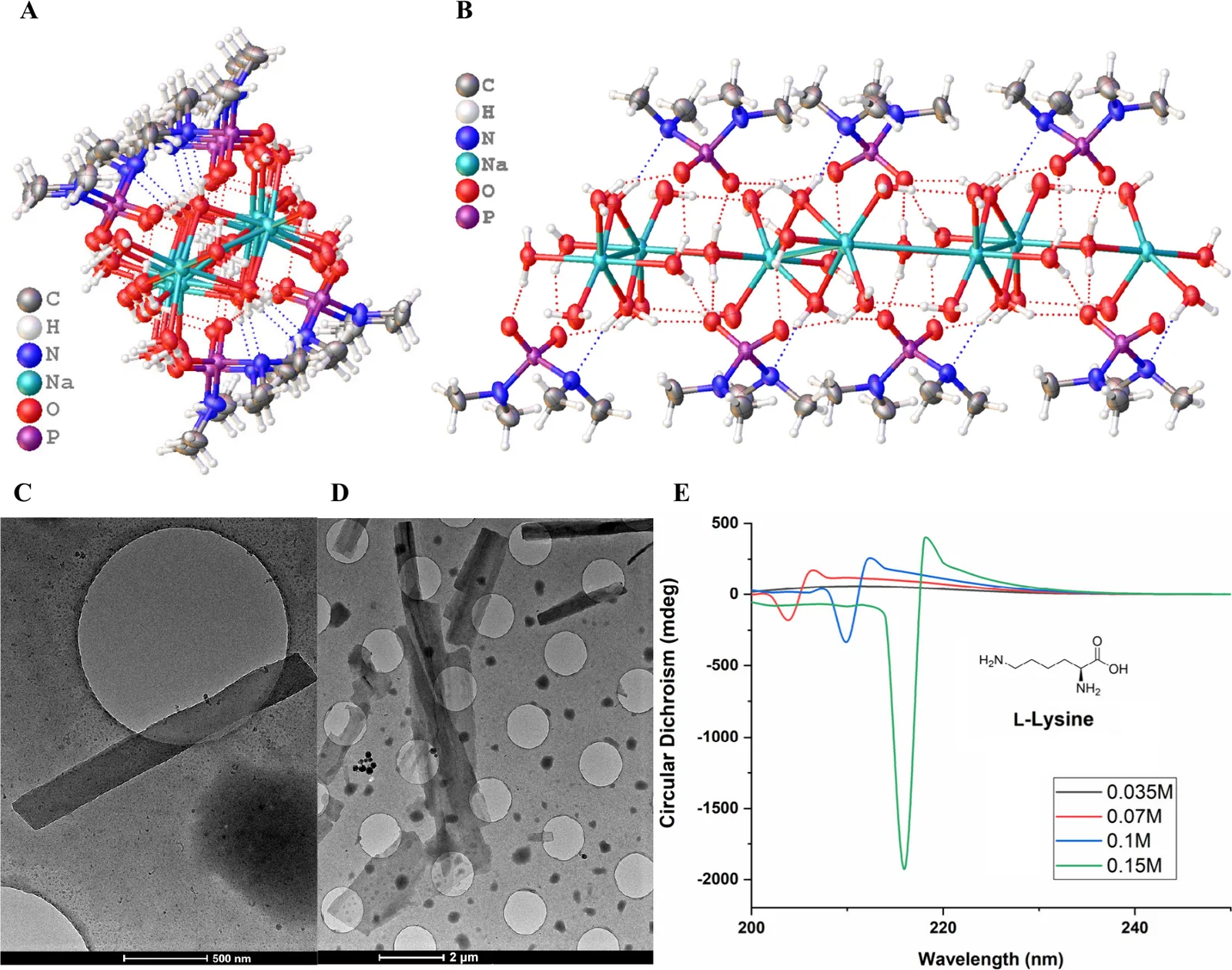
Spectroscopic characterization of aggregatable molecules. (A) Crystal packing structure of BDMDAP in a stacked view. (B) Crystal packing structure of BDMDAP in a planar view. (C) Cryo-TEM image of a single bundle formed by DAP aggregates (concentration: 0.03 M; bundle/rod: DAP aggregate; bubble: ethane). (D) Cryo-TEM image of multiple bundles formed by DAP aggregates. (E) CD spectra of l-lysine at different concentrations.

To further explore the mechanism of the observed spectral change, density functional theory (DFT) computation was carried out with Gaussian 16 (Revision A.03) to identify plausible aggregation forms. DAP was selected as the representative molecule, since its AIA effect was the strongest among the tested compounds. The geometries of the DAP monomer and aggregates in water were optimized with CAM-B3LYP as the density function and 6-31+G­(d,p) as the basis, which are frequently employed to calculate systems rich in hydrogen-bond donors and acceptors.^58^ The excited states (*n* = 10) were calculated using the TD-SCF method, with CAM-B3LYP as the density function and 6-311++G­(d,p) as the basis for enhanced accuracy. The solvation effect was considered (water, PCM) for both geometry optimization and excited state calculations. The longest absorption wavelength (λ_longest_) and the oscillator strength (*f*) were computed as the key parameters. The optimized geometries of the monomer and aggregated forms as well as λ_longest_ and *f* values are displayed in Figure 6. DAP primarily exists in zwitterionic form with the positively charged −NH_3_^+^ and the negatively charged −O^–^, thus possessing a strong dipole moment. The calculated λ_longest_ of the DAP monomer was 180.8 nm with *f* = 0.0297 (Figure 6A; for the complete set of calculated λ and *f*, see the Supporting Information). The experimentally observed AIA effect, particularly a redshift or a newly emerged band at a longer wavelength, is a hallmark of specific molecular packing (such as J-aggregation)^10^ where transition dipole moments couple to lower the excitation energy. However, before we realized that, the lowest aggregated form of DAP was first proposed with a conventional thought to be a dimer constructed through two intermolecular P–O^–^···H–^+^NH_2_–P hydrogen bonds (Figure 6B). The calculated λ_longest_ and *f* of such a DAP dimer with two equatorial P=O bonds were 177.7 nm and 0.0487, indicating a slight blueshift. Switching the orientations of the two P=O bonds between axial and equatorial did not result in much change in the λ_longest_ and *f* values (Figure 6C, λ_longest_ = 178.0 nm and *f* = 0.0602 for axial-axial; Figure 6D, λ_longest_ = 178.5 nm and *f* = 0.0239 for equatorial-axial). After finding that the calculated λ_longest_ and *f* of the hydrogen-bonded dimers did not match the experimental results, we turned our attention to J-aggregation, which is predicated by the interaction between “head-to-tail” transition dipoles.^12^ Accordingly, a model was constructed with two DAP molecules organized parallelly in a slip-stacked arrangement. Following geometric optimization of this stacked dimer (Figure 6E), the excitation properties were calculated. The results showed that in addition to the appearance of a new band at 195.8 nm (*f* = 0.0279), the intrinsic absorption of DAP itself redshifted to 186.2 nm and intensified (*f* = 0.0515, see the Supporting Information). While deviations may exist between calculated and experimental wavelengths, the simulated trend aligns with observations. Furthermore, the tail of the longest absorption peak (centered at λ_longest_) may extend further into the red region. The trimer and tetramer of DAP were optimized with a similar structure, and their calculated λ_longest_ values were 201.9 and 204.5 nm, respectively (Figure 6F,G). Their corresponding oscillator strengths (0.0054 and 0.0018) at λ_longest_ were not as high as those of the monomer and the stacked dimer. However, it was noteworthy that the absorption at λ_longest_ of the trimer and tetramer actually belonged to newly emerged bands, while absorptions near 180 nm (as in the monomer and stacked dimer) and 195 nm (as in the stacked dimer) were also present with normal *f* values and more redshifts from the monomer (see the Supporting Information). These results implied that a greater AIA effect can be expected upon the growth of such aggregates.

**Figure 6:**
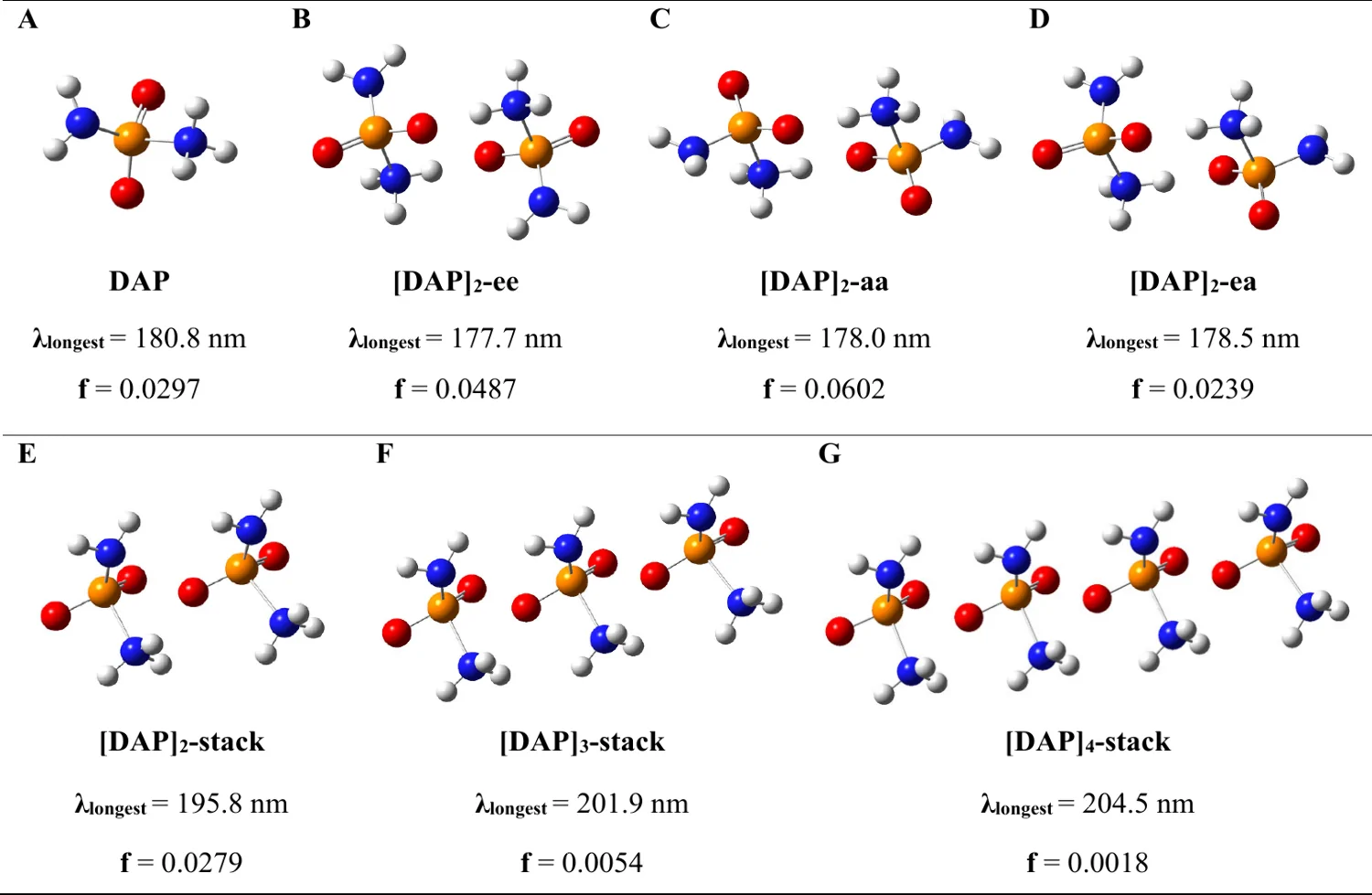
Optimized geometries of DAP monomer and aggregates in water with the longest absorption wavelengths and oscillator strengths. (A) DAP monomer; (B) hydrogen-bonded DAP dimer with two P=O bonds being equatorial-equatorial; (C) hydrogen-bonded DAP dimer with two P=O bonds being axial-axial; (D) hydrogen-bonded DAP dimer with two P=O bonds being equatorial-axial; (E) slip-stacked DAP dimer; (F) slip-stacked DAP trimer; and (G) slip-stacked DAP tetramer.

The experimental and computational results prompted us to explore the empirical correlation between the intensity of AIA and certain structural factors of a prebiotic molecule. Since a simplistic dipole moment cannot give a perfect correlation of the observed order of AIA, charge-transfer interaction was also considered, which can be a prerequisite for J-aggregation.^11^ Based on the apparent AIA effects (redshift and new long-wave bands) in DAP, cysteine, lysine, arginine, lactic acid, citric acid, and oxalic acid and the less significant AIA in alanine, serine and other amino acids, and complete absence of AIA in glycolaldehyde and glyceraldehyde, a more fundamental and predictive structure–property relationship is established as follows. For an effective electronic coupling, the molecule must possess a strong electron acceptor (e.g., P=O or C=O). For the electron donor, a geminal one (e.g., −O^–^ and −NH_2_ in DAP) that directly connects to the acceptor offers the strongest electron-donating effect (Figure 7A). A vicinal electron donor, such as α-OH in lactic acid or citric acid (oxalic acid can also be considered as having an α-OH group) or unprotonated −NH_2_ in an amino acid, is also sufficient (Figure 7B). Noteworthily, the α-NH_2_ must remain unprotonated to maintain its lone pair electrons as a strong donor. That means the amino acid must possess a proton-buffering group (e.g., a remote −NH_2_, guanidyl, or other basic groups stronger than α-NH_2_) that takes the proton from −COOH (Figure 7C). This is likely the reason that lysine and arginine have strong AIA, while glycine, alanine, and glutamic acid do not. The strong AIA in DAP can also be partially attributed to this interaction, with one geminal −NH_2_ acting as the proton buffer to take H^+^ from the −OH. The necessity of a proton-buffering group was confirmed by a pH-dependence study of lysine’s UV absorption, where the AIA effect was much stronger at basic pH than at neutral or acidic pH (see the Supporting Information). In histidine, the imidazole group (p*K*_a_ ≈ 6.0) is less basic than α-NH_2_ (p*K*_a_ ≈ 9.2); thus, its AIA is not intense (see Figure S33 in the Supporting Information). A β-donor may also work, provided that it is highly polarizable to ensure long-range interaction. This is well reflected in the cases of cysteine and serine. Although their α-NH_2_ are both shielded with a proton, cysteine is still strongly AIA-active because of the soft β-SH (Figure 7D), while serine has a much reduced AIA due to the absence of such a donor, as the β-OH is less polarizable (Figure S38). If both the α- and β-donor are protonated or absent, as long as the amino acid bears an alkyl side chain, its hyperconjugation effect may still render it a weak electron donor. This is probably the reason that weak AIA is still observed for alanine and serine, while such an effect in glycine is the weakest (Figure S40). In principle, glycolaldehyde and glyceraldehyde both meet requirements of having an acceptor (C=O) and a donor (α-OH), but their predominant form in aqueous media is the hydrate (geminal diol) as opposed to aldehyde, thereby hampering the charge-transfer process.

**Figure 7:**
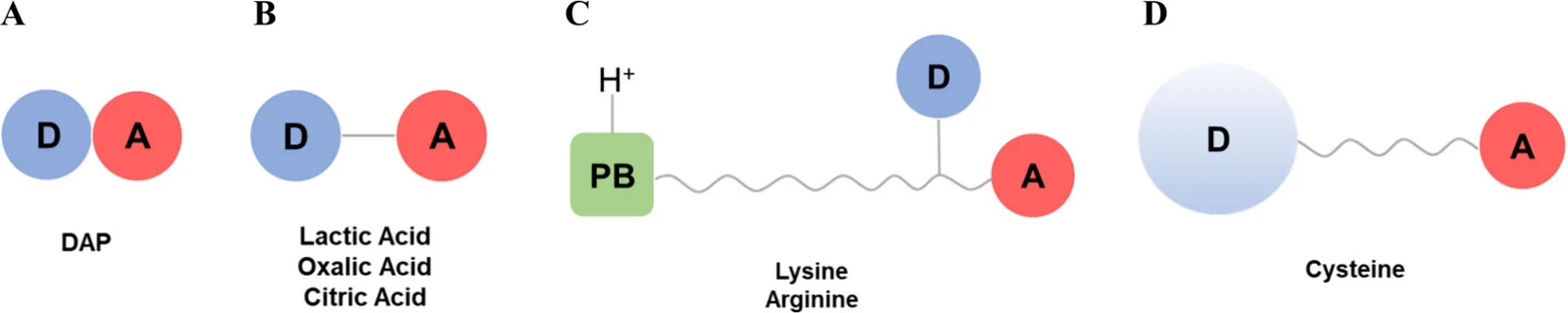
A predictive structure–property relationship based on charge-transfer interaction. (A) Acceptor with a geminal donor; (B) acceptor with a neutral vicinal donor; (C) acceptor with a basic vicinal donor and a remote proton-buffering (“PB”) group; and (D) acceptor with a polarizable donor. (The straight line denotes a shorter interatomic distance; the longer, wavy line denotes a greater distance.).

### A Concentration-Gated Switch between Photoreaction and Photoprotection

To examine the efficacy of AIA-active early small molecules as potential photoprotectants, we first compared the UV-filtering ability of phosphoramidate and Fe­(II)^32^ at the same concentration (0.01 M). It was found that DAP demonstrated a much greater absorbance than FeCl_2_ over the entire range of 200–300 nm (Figure 8A). We then selected 2-aminooxazole, a highly photolabile molecule^29^ and key intermediate in prebiotic nucleoside and nucleotide synthesis,^59,60^ as the model compound. To isolate the UV-filtering effect of phosphoramidate from its potential chemical interference with 2-aminooxazole, we employed a concentric vial design: a small quartz vial containing 2-aminooxazole was placed inside a larger quartz vial (see the reaction setup in Figure 8C); the chamber was filled with 1.0 M phosphoramidate solution as the protectant or water as the blank, maintaining physical separation from 2-aminooxazole while allowing light transmission (see Figure S51 in the Supporting Information). Samples were irradiated using a dual low-pressure Hg lamp set (λ = 254 nm, placed on both sides of the quartz vials for even irradiation) for 8 h. HPLC analysis of the postirradiation mixture revealed the complete consumption of 2-aminooxazole in the absence of the protectant, with the concomitant appearance of multiple minor peaks of degradation products. After 8 h of irradiation, 2-aminooxazole remained largely intact with a shield provided by phosphoramidate (Figure 8B). Based on the HPLC results, the reaction shielded by BDMDAP suffered a little degradation, whereas the shield by DAP was more efficient.

**Figure 8:**
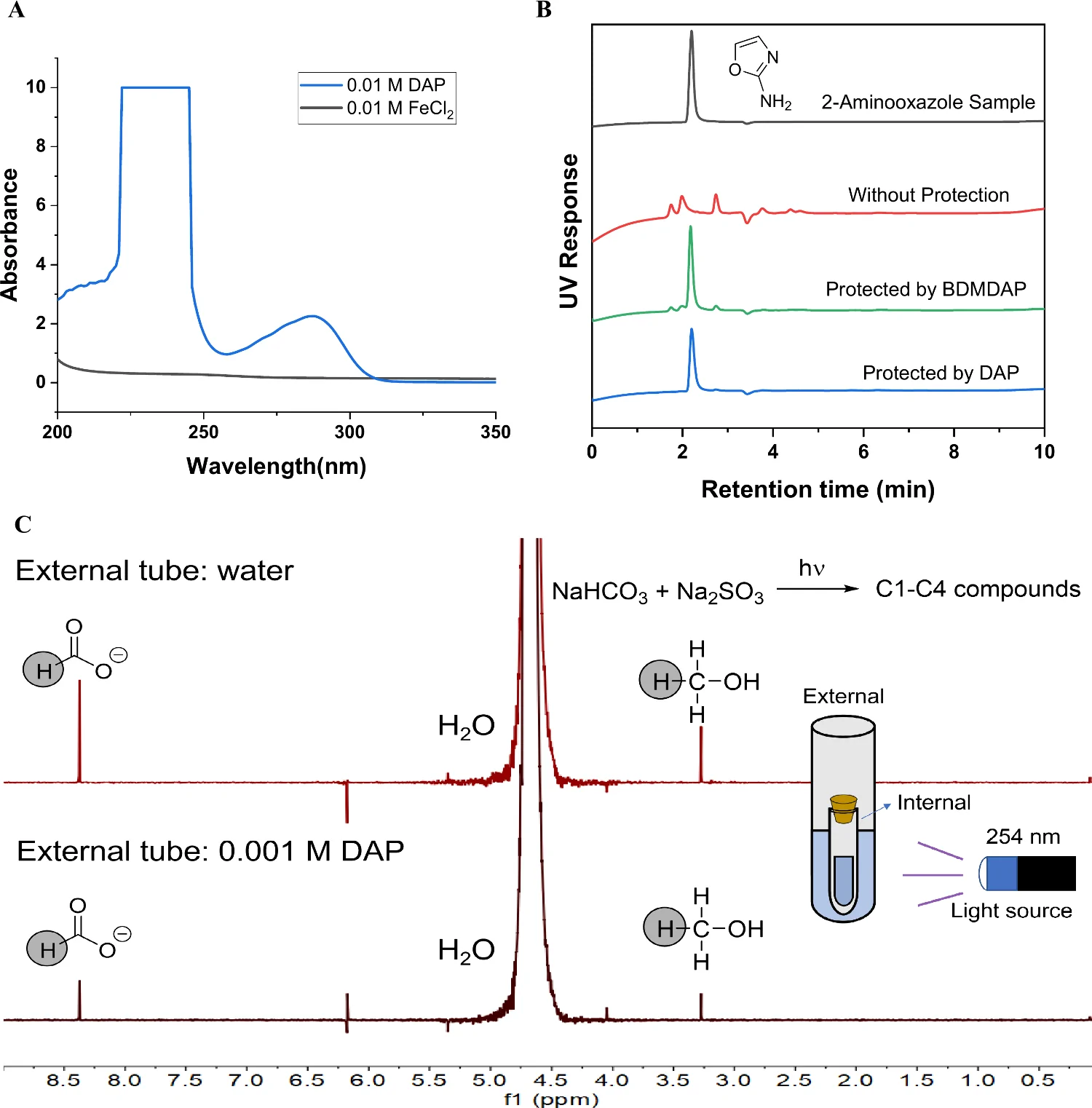
Dual function of phosphoramidate regulated by concentration. (A) Comparison of the UV spectra of DAP and FeCl_2_, both at 0.01 M. (B) UV protection of 2-aminooxazole by concentrated phosphoramidate solutions (1 M). (C) Reaction setup of the photoredox transformation of bicarbonate and sulfite to small organic molecules in the absence and presence of dilute DAP solution (0.001 M) and the ^1^H NMR spectra (400 MHz, D_2_O).

Subsequently, we examined whether a dilute solution of phosphoramidate can allow the necessary photoreaction or not. It was reported that carbonate and sulfite can undergo a photoredox reaction to generate a series of simple C1 to C4 compounds under UV irradiation.^61^ Within the concentric setup, water was charged in the external vial, and a basic solution (pH = 9) of NaHCO_3_ (0.02 M) and Na_2_SO_3_ (0.04 M) was added to the internal vial. The mixture was irradiated at 254 nm for 4 h. ^1^H NMR analysis of the mixture in D_2_O showed the formation of small amounts of formate (δ 8.38 ppm) and methanol (δ 3.28 ppm) (Figure 8C, top). Replacing water in the external quartz vial with dilute DAP solution (0.001 M) did not block the reaction, as formate and methanol were also detected by NMR (Figure 8C, bottom). This observation demonstrated that a dilute solution of photoprotectant where molecular aggregation is minimal can maintain sufficient UV transparency to permit targeted photoreactions.

### On-Site Photoprotection of Essential Biopolymers by AIA-Active Small Molecules

Building on the core mechanism proposed earlier, we further validated the applicability of these potential photoprotectants to key biopolymers that emerged during the origins of life. The UV-shielding efficacy of phosphoramidate (e.g., DAP) and amino acid (e.g., cysteine) was investigated with a short RNA strand and a peptide, respectively. In this scenario, the photoprotectant also serves as the actual reagent or building block to synthesize these substrates. Considering that these protectants and biomolecules were more likely to coexist in a mixed state rather than physically separated, the experimental design was adjusted such that the protectant was directly mixed with the substrate in one quartz vial for UV irradiation without the concentric design. A 5-nt RNA oligomer (sequence: 5′-UACGU-3′) was selected as the substrate and dissolved in RNase-free water. The RNA solution was placed in a quartz vial and irradiated for a short period (4 h) using a dual low-pressure Hg lamp system (λ = 254 nm), with lamps symmetrically positioned on both sides of the quartz tube to ensure uniform irradiation. LCMS analysis of the postirradiation mixture revealed some degradation of the oligomer (<10% conversion to a new peak) in the absence of DAP (Figure 9A). The mass spectrum of the new LC peak showed signals at *m*/*z* 340 and 362 (see Figure S55B for the MS spectrum), which likely correspond to [M + H]^+^ and [M + Na]^+^ of something with a molecular weight of 339. One plausible structure of this product could be a derivative of cytidine monophosphate (5′-CMP; or 2′/3′-CMP via the hydrolysis of cyclic phosphate),^62^ which was probably derived from the photodegradation of the RNA oligomer followed by a photohydration-dehydrogenation sequence^63^ (Figure 9C). The detection of this derivatized CMP suggested that multiple cleavages of the oligomer (5′-UACGU-3′) likely took place to release its central fragment. Subsequently, the same oligomer was mixed with DAP (0.03 M after mixing) and irradiated under identical UV conditions for 4 h. LCMS analysis revealed that the oligomer remained intact without degradation (Figure 9A). For evaluating the UV-shielding efficacy of cysteine, hexapeptide-11 (H-Phe-Val-Ala-Pro-Phe-Pro-OH) was employed as the target peptide. Without cysteine protection, the solution of hexapeptide-11 alone was irradiated under the aforementioned UV conditions for 4 h. LCMS analysis showed a new peak with *m*/*z* 292 (Figure 9B; see Figure S57B for the MS spectrum), which may correspond to the fragmentation of hexapeptide-11 into a dipeptide derivative (Figure 9C). In contrast, the irradiation of the mixed solution of hexapeptide-11 and cysteine did not result in any degradation product (Figure 9B). A control experiment was performed in the presence of ammonium dihydrogen phosphate as an AIA-inactive compound, and the same decomposition product at *t*_R_ = 15 min was observed (Supporting Information). The mechanism of such photoprotection does not rely on encapsulation, surface coating, or any specific spatial relationship between AIA aggregates and biomolecules. Instead, it follows directly from the Lambert–Beer law that AIA aggregates strongly absorb UV light within a very short path length near the solution surface, drastically reducing UV penetration into the bulk. Thus, molecules in the deeper solution, including AIA molecules themselves, are protected simply by the attenuation of incident photons.

**Figure 9:**
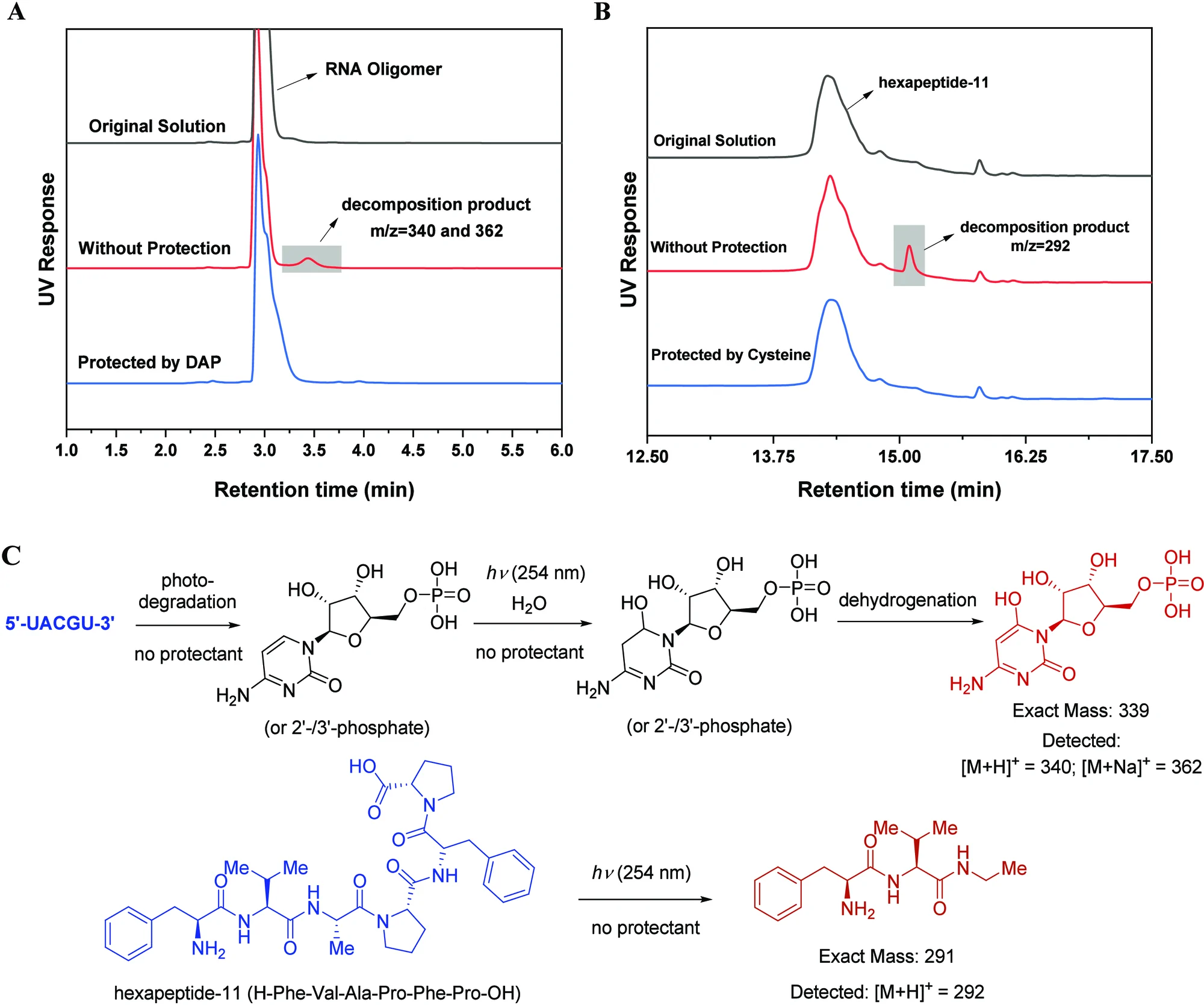
On-site photoprotection of RNA oligomer and oligopeptide by aggregatable phosphoramidate and amino acid. (A) LC chromatograms of the experiments of an RNA oligomer (5′-UACGU-3′, *t*_R_ = 2.9 min; decomposition product, *t*_R_ = 3.4 min). (B) LC chromatograms of the experiments of a hexapeptide (hexapeptide-11, *t*_R_ = 14.3 min; decomposition product, *t*_R_ = 15.1 min). (C) Possible photodegradation pathways and detected products in the absence of photoprotectant.

### Photoprotection of Synthetic Polymeric Materials by Aggregatable Small Agents

The aforementioned studies have demonstrated that photoprotection via concentration-dependent absorption can effectively resist UV damage to life-related biopolymers without interfering with the key photoreactions. Modern polymers commonly suffer from UV-induced degradation,^2^ which triggers molecular chain scission and cross-linking, leading to a reduction of mechanical strength and shortening of service life. Aside from being a potential UV shield for primitive biopolymers, can these AIA-active molecules offer photoprotection to contemporary materials? For example, the durability of plastic components within UV disinfection systems presents a persistent and costly challenge for industrial and consumer use.^64^ While internal UV irradiation is essential for sterilization, it may simultaneously degrade the materials (e.g., wiring insulation, sensor housings, polymer mounts) that constitute the equipment, leading to yellowing, embrittlement, and premature failure. Traditional solutions such as compounding polymers with molecular UV absorbers^65^ (e.g., benzophenones) are often economically impractical, since the permanent modification of the entire material is an overengineering for low-value, nonstructural parts. Moreover, toxicity is another serious disadvantage.^66^ Switching to postmanufacture protection by uniform solid coating is also difficult due to the complex internal geometries of such equipment.^67^ Our envisioned approach addresses this problem by leveraging AIA-active molecules (such as lactic acid and lysine) that aggregate to protect the plastic surface. The advantages of this strategy include the following: (1) It is a noninvasive, postfabrication process applicable to complex assemblies. (2) It is employed as a sacrificial and renewable UV shield that can be reapplied (e.g., by spraying) during routine maintenance without device disassembly. (3) It utilizes inexpensive, biologically compatible reagents that circumvent the toxicity and environmental concerns associated with leachable synthetic absorbers.

Molecules that have been verified to exhibit AIA effects were selected as candidates for the protection of the polymeric materials. Lactic acid is commercially produced via the microbial fermentation of carbohydrates, making it a cost-effective and renewable platform chemical.^68^ Lysine, as we previously investigated, has the broadest UV absorption when concentrated (Figure 4B). Polypropylene (PP) and polycarbonate (PC) were adopted as model systems to validate the efficacy of the photoprotection. To ensure the applicability of the photoprotectant, we first evaluated its intrinsic stability under UV irradiation. An aqueous solution of lactic acid (3 M) was prepared and exposed closely to UV lamps (254 nm) for 12 h, during which the volume of solution was maintained. HPLC analysis of the solutions before and after UV exposure was performed by applying the same injection volume (Figure 10A). Using the LC peak area of lactic acid as a key quantitative parameter, the ratio of the peak area after irradiation to that before was calculated as 0.91, indicating that lactic acid underwent no significant degradation under the UV conditions. This allows lactic acid to avoid the attenuation of protective efficacy caused by rapid consumption during the exertion of its photoprotection function.

**Figure 10:**
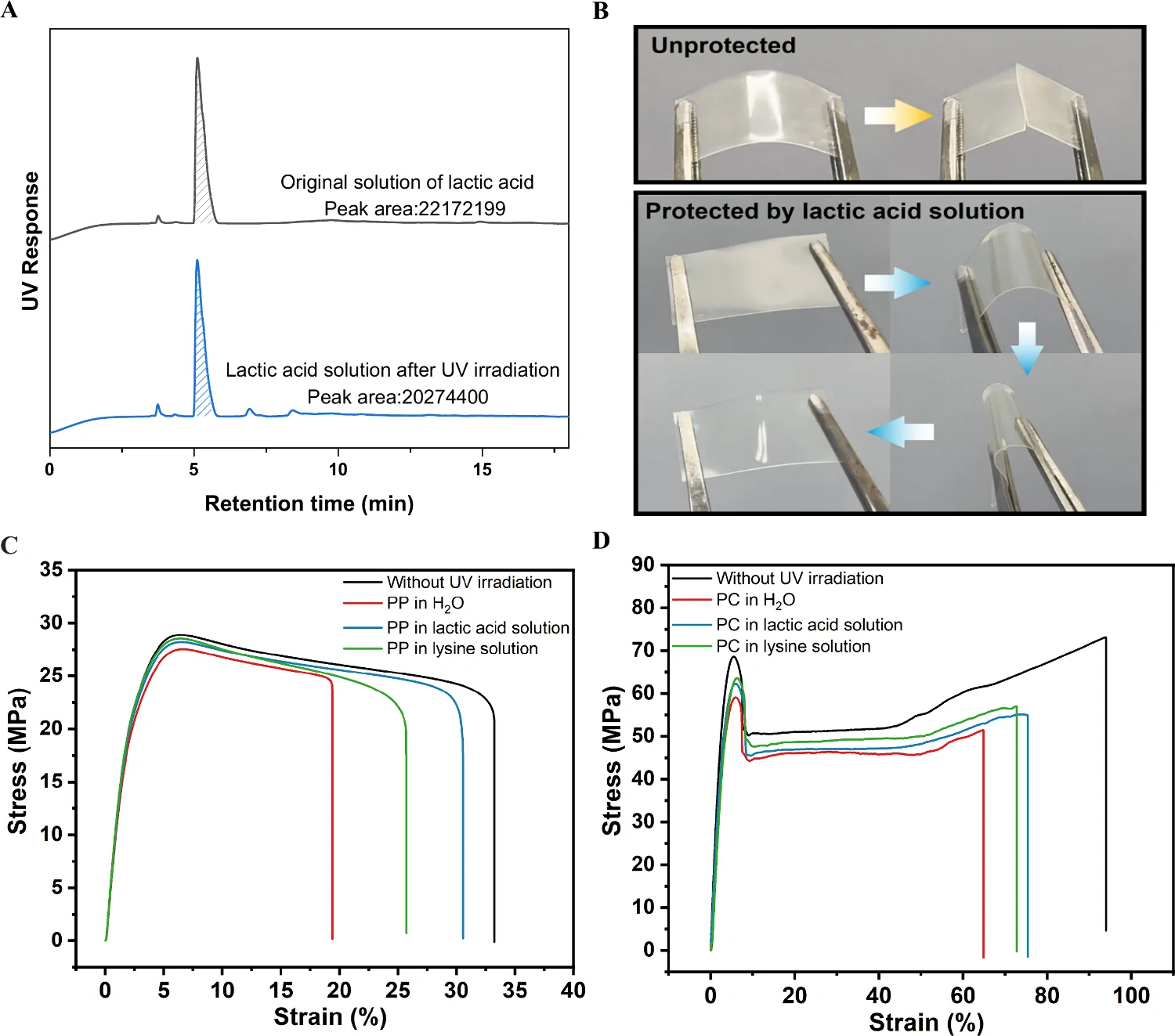
Photoprotection of polymeric materials utilizing lactic acid and lysine. (A) Consumption test of lactic acid upon UV irradiation at 254 nm for 12 h. (B) Irradiated PP in the absence and presence of lactic acid (3 M in water) as photoprotectant. (C) Tensile stress–strain diagrams of PP protected by lactic acid or lysine. (D) Tensile stress–strain diagrams of PC protected by lactic acid or lysine.

The UV-protective efficacy of lactic acid for PP and PC materials was further investigated. For each material, two experiments were conducted in parallel: identical specimens were placed horizontally and separately in two clean Petri dishes (see Figure S64 for the dimensions of the specimens). The control group was added with water, while the experimental group was supplemented with 3 M aqueous lactic acid solution. The specimens were fully immersed in the respective solutions. Both groups were placed under the same UV conditions (254 nm). After 24 h, the specimens were flipped over for an additional 24 h of irradiation to ensure uniform irradiation on both sides (total UV exposure duration: 48 h). Upon completion of the irradiation, the specimens were retrieved for mechanical tensile testing. The UV-protective function of lactic acid was evaluated by comparing the mechanical properties between the unprotected and protected groups. The results are shown in Figure 10C,D. The data clearly showed that the specimens without any protection exhibited distinct reductions in mechanical properties: stress decreased by 1.33 MPa for PP and 9.52 MPa for PC, and strain declined by 13.8% for PP and 29.1% for PC. In stark contrast, protection with lactic acid solution led to much more moderate declines. For PP specimens protected by lactic acid solution, stress and strain decreased by only 0.68 MPa and 2.7%, respectively. Similarly, the stress and strain of PC specimens protected by lactic acid solution decreased by only 6.32 MPa and 18.6%, respectively. To further demonstrate the protective effect and to seek an operationally easier method, the surfaces of two PP specimens (20 × 10 × 0.5 mm) were treated by respectively spraying distilled water and a 3 M lactic acid aqueous solution onto them, followed by 24 h of UV irradiation (see the Supporting Information). As clearly shown in the supplementary videos, the PP sheet without lactic acid protection became brittle after irradiation and fractured readily upon bending (Figure 10B and Supplementary Video 1). In contrast, the PP sheet protected by lactic acid could be bent nearly 180° without fracture (Figure 10B and Supplementary Video 2). These results demonstrated conclusively that lactic acid can provide effective protection against UV-induced degradation of plastics.

In addition, parallel validation experiments on the UV-protective efficacy of lysine for PP and PC specimens were simultaneously conducted. Following the standardized procedure described above, an aqueous solution of lysine (0.6 M) was prepared and the PP and PC specimens were subjected to UV irradiation for 48 h. Then, mechanical tensile tests were also conducted (Figure 10C,D). Under the protection by lysine solution, PP and PC specimens exhibited slightly decreased mechanical properties of 0.35 MPa and 7.5% (for PP) and 5.02 MPa and 21.2% (for PC), respectively. These reductions were less severe than those observed in unprotected specimens, underscoring the protective efficacy of lysine.

## Conclusions

A broad category of small molecules that form UV-shielding aggregates has been identified, which allows photoreactions in dilute solution but protects biomolecules from overirradiation when concentrated. The concentration-dependent self-assembly behavior of phosphoramidates, amino acids, hydroxy carboxylic acids, and others has been collectively revealed by UV–vis, MS, and NMR analyses. Contrary to conventional thought, these simple small molecules develop aggregation-induced absorption in the upper UVC range (204–280 nm), despite lacking traditional chromophores. Dynamic light scattering, single crystal X-ray diffraction, Cryo-TEM, circular dichroism, and variable-temperature UV–vis spectroscopy collectively revealed that this effect originates from the supramolecular self-assembly of such molecules, likely via slip-stacked J-aggregation. Density functional theory calculations of the geometries and excited states in water elucidated the emergence of new absorption bands at longer wavelengths. A predictive structure–property relationship of potential AIA primitive molecules has been proposed. As a plausible ancestral analogue for scytonemin and eumelanin, these aggregatable small molecules can act as effective prebiotic UV shields for safeguarding photolabile yet critical life-related macromolecules. Conversely, in a dilute solution of such protectants, necessary photoreactions keep occurring smoothly. These observations may further imply that many prebiotically essential molecules may possess an innate ability to shield themselves from excessive irradiation damage. Aside from being used as prebiotic UV shields, the utility of these AIA molecules has been cross-disciplinarily extended to the protection of polymeric materials. This strategy employs a simple formulation of reagents such as lactic acid and lysine, thereby creating greener, maintainable, and cost-effective photoprotection protocol for vulnerable plastic components in critical artificial UV environments.

Unlike conventional aromatic UV absorbers, these small AIA molecules (e.g., phosphoramidates) offer several advantages: They require no extended π-conjugation, enabling upper UVC absorption via the aggregation of heteroatom-rich molecules. They are polar and water-compatible for prebiotic relevance. Their concentration-dependent spectral behavior allows photoreactions under dilute conditions. Lastly, they are greener for polymer protection than toxic aromatics (e.g., benzophenones).

In conclusion, these findings highlight how molecular crowding dynamically modulates the photophysics of simple building blocks of life. The observed self-assembly likely served the role of on-demand, autoregulated protection of photolabile intermediates and products. The results also offer new insights into the development of sustainable, safe, and precisely controllable photoprotection strategies for synthetic materials.

## Supplementary Material







## Data Availability

The data underlying this study are available in the published article and its Supporting Information.

## Notes

Full experimental procedures, characterization data for key compounds, HPLC traces, MS spectra, ^31^P NMR spectra, UV–vis spectra, DLS analysis, and DFT calculation data (PDF)
Supplementary Video 1: Bending test of PP sheet without lactic acid protection, which fractured readily (MP4)
Supplementary Video 2: Bending test of PP sheet with lactic acid protection, which could be bent nearly 180° (MP4)
