# Supplementary material for: On-Demand Photoprotection by UV-Transparent Small Molecules via Aggregation-Induced Absorption: The Origin and Modern Application
Source: ACS Cent Sci. 2026 Aug 17;12(9):1489–503. doi: 10.1021/acscentsci.6c01033 (PMC13614032; doi:10.1021/acscentsci.6c01033)
Supplement: Supplementary file 1 [file oc6c01033_si_001.pdf]

Supporting Information

On-Demand Photoprotection by UV-Transparent Small Molecules via Aggregation-Induced Absorption: The Origin and Modern Application

Bing-Yue Zhao[1], Yao Hui[1], Wan-Jun Qin[1], Qing Liu[1], Xiao Wang[1,2,*]

Corresponding author: wangxiao@nju.edu.cn

[1] State Key Laboratory of Coordination Chemistry, School of Chemistry and Chemical Engineering, Nanjing University, Nanjing, 210023, Jiangsu, China

[2] Frontiers Science Center for Critical Earth Material Cycling, Nanjing University, Nanjing, 210023, Jiangsu, China

Table of Contents

General Information of Reagents……………………..…………………………..……..………......…S3

General Information of Instruments and Methods…………………………..……...................S3

Synthesis and characterization of diamidophosphate (DAP)………………………….…….....S5

Cryogenic transmission electron microscopy (Cryo-TEM) of DAP solution……..……..……....S8

ICP-MS elemental analysis of DAP……..………….…………….……...……….……..……....S10

Synthesis and characterization of BDMDAP……………..…………………...………………S11

Concentration-dependent ^{1}H NMR spectra of BDMDAP ………….……..……...………….S17

Single crystal X-ray diffraction data of BDMDAP……...…………...…………………...………S18

UV-Vis spectra of prebiotic relevant small molecules……..……………………………………S21

UV absorption of DAP before and after centrifugation…………………………………………S40

Temperature-dependent UV-Vis absorption measurements of DAP……………………………S41

pH-dependent UV-Vis absorption of lysine……………………………………………………S41

Circular dichroism evidence for chiral excitonic coupling in AIA aggregates…………………S42

UV-Vis spectra of short peptides………………..……………………………………………S43

Photoprotection of 2-aminooxazole by phosphoramidate…………………………………......S45

Photoreaction between bicarbonate and sulfite to produce C1 compounds……………...…....S46

On-site photoprotection of an RNA oligomer by DAP……………………………….………S48

On-site photoprotection of a hexapeptide by cysteine………..…………………………….....S50

Dynamic light scattering analysis of DAP solution………..…..……..…………………………S52

Dynamic light scattering analysis of lysine solution………..…..…………………..……………S54

Dynamic light scattering analysis of filtered water……..…..…..…..……..…………………..…S54

Dynamic light scattering analysis of glyceraldehyde…....…..…..…...…..……………....…..……S55

Intrinsic photostability test for lactic acid………….………………..…………...………......S56

UV-protective performance of aggregatable molecules for synthetic polymeric materials……..S57

Photoprotection of polypropylene sheet by spraying aqueous lactic acid solution…………..……S58

DFT calculation of the geometries and excited states of DAP and aggregates in water …..…..…S59

General Information of Reagents:

Bis(dimethylamino)phosphorochloridate (CAS 1605-65-8) was purchased from Shanghai Bidepharm Co., Ltd (93%). Phenyl phosphorodiamidate (CAS 7450-69-3) was purchased from Shanghai Bidepharm Co., Ltd (98%). L-Cysteine (CAS 52-90-4) was purchased from Aladdin (99%). L-Lysine (CAS 56-87-1) was purchased from Tianjin Xiansi Biochemical Technology Co., Ltd (98%). L-Arginine (CAS 74-79-3) was purchased from Adamas-beta (99%). DL-Lactic acid (CAS 50-21-5) was purchased from Sinopharm Chemical Reagent Co., Ltd (85%). L-Lactic acid (CAS 79-33-4) was purchased from Shanghai D&B Biological Science and Technology Co., Ltd. Citric acid monohydrate (CAS 5949-29-1) was purchased from Sinopharm Chemical Reagent Co., Ltd. Oxalic acid (CAS 144-52-7) was purchased from Shanghai Bidepharm Co., Ltd (99.47%). L-Histidine (CAS 71-00-1) was purchased from Adamas-beta (98%). L-Alanine (CAS 56-41-7) was purchased from Shanghai Yuanye Bio-Technology Co., Ltd. L-Glutamic acid (CAS 56-86-0) was purchased from Shanghai Titanchem Co., Ltd (99%). L-Serine (CAS 56-45-1) was purchased from Aladdin (98.5−101.0%). Glycine (CAS 56-40-6) was purchased from Shanghai Titanchem Co., Ltd (AR, 99.5−100.5%). Glycolaldehyde dimer (CAS 23147-58-2) was purchased from Shanghai Bidepharm Co., Ltd (95%). DL-Glyceraldehyde (CAS 56-82-6) was purchased from Shanghai Macklin Biochemical Co., Ltd (90%). Ammonium dihydrogen phosphate (CAS 7722-76-1) was purchased from Shanghai Titanchem Co., Ltd (99%). Oxazole-2-amine (2-aminooxazole, CAS 4570-45-0) was purchased from Shanghai Bidepharm Co., Ltd (98%). Sodium sulfite (CAS 7757-83-7) was purchased from Sinopharm Chemical Reagent Co., Ltd (AR). Sodium hydrogen carbonate (CAS 144-55-8) was purchased from Sinopharm Chemical Reagent Co., Ltd (AR). The 5-nt RNA oligomer (sequence: 5'-UACGU-3') in RNase-free water were purchased from Beijing Tsingke Biotechnology Co., Ltd. Hexapeptide-11 (H-Phe-Val-Ala-Pro-Phe-Pro-OH, 95%), hexapeptide-9 (H-Gly-His-Pro-Lys-Ser-Thr-OH, 98%), and acetyl tetrapeptide-11 (Ac-Pro-Pro-Tyr-Leu-OH, 98%) were purchased from Shandong Jitai Biotechnology Co., Ltd. Cys-Cys dipeptide was purchased from Anhui ZT Biotech Co., Ltd. Ultrapure water (DNase/RNase-Free, Sterile) was purchased from Adamas Life.

General Information of Instruments:

HPLC analyses were performed on Shimadzu LC-2050 with SymmetryShield™ RP18 column (5 μm, 4.6 mm I.D. × 250 mm), at a detection wavelength of 254 nm (or other wavelength specified in individual procedures), with water (adjusted with 0.1% formic acid, as phase A) and acetonitrile (as phase B) as the eluents. HPLC analysis was performed at a flow rate of 0.33 mL/min, and the gradient condition was: 0→45 min, 100% A to 33% A; 45→45.2 min, 33% A to 100% A.

^{1}H, ^{13}C and ^{31}P NMR spectra and ^{1}H–^{13}C HSQC data were recorded on a Bruker AVANCE III 400 MHz spectrometer. Chemical shifts (δ values) were reported in parts per million (ppm)

relative to residual D_2O (δ 4.79) for ^1H and internal 85% H_3PO_4 (δ 0.00) for ^{31}P. Data are presented as: chemical shift (δ), multiplicity, coupling constant (*J* value in Hz), integration.

LC-MS analyses were performed on a SHIMADZU LCMS-8040, with SHIMADZU LC-20AD pump, SPD-M20A UV detector, SIL-20A HT auto-sampler, and electrospray ionization (ESI) MS detection mode. The column and gradient conditions were the same as those of the HPLC analysis.

High-resolution mass spectra (HRMS) were recorded on an Agilent 6540 Q-TOF mass spectrometer (Agilent Technologies, USA).

Inductively coupled plasma mass spectrometry (ICP-MS) measurements were performed using an Agilent 7850 ICP-MS instrument (Agilent Technologies, USA).

UV-Vis absorption spectra were acquired using a PerkinElmer LAMBDA 750S UV/Vis/NIR spectrophotometer, which is able to make highly accurate measurements up to 6 Absorbance units. Measurements were performed in transmission mode (185–3300 nm) or equipped with an integrating sphere (220–2600 nm). The instrument offers a spectral resolution of 0.1 nm and selectable bandwidths (UV/Vis: 0.1, 0.2, 0.5, 1, 2, 3, 5 or 8 nm). Quartz cuvettes (model 751 (722), path length = 10 mm) were used for solution samples.

Temperature-dependent UV-Vis absorption measurements were performed on a Lambda 1050+ UV/Vis/NIR spectrophotometer (PerkinElmer, USA) using a DC-2015 thermostatic bath/circulator (Scientz, China) for temperature control.

Dynamic light scattering (DLS) measurements were performed on a Brookhaven (USA) DLS instrument, equipped with dual laser sources (640/532 nm), interchangeable aperture wheels (100/200 μm), and a high-precision temperature control system (range: –5 to 80 °C, accuracy: ±0.1 °C). Its particle size measurement range spans from 1 nm to 6 μm. Measurements were conducted at 25.0 °C with the following parameters: laser wavelength 532.0 nm, detection angle 90.0°, solvent refractive index 1.334.

Circular dichroism (CD) spectra were recorded on a Chirascan V100 (Applied Photophysics, UK) spectropolarimeter using a quartz cuvette with a path length of 0.5 mm. Samples were prepared in ultrapure water. The spectra were collected from 200 to 250 nm at room temperature. Each spectrum was recorded from a single scan, and each sample was measured in triplicate. The corresponding solvent spectrum was subtracted as the baseline. The CD signals are reported as the observed ellipticity in millidegrees (mdeg).

Cryogenic transmission electron microscopy (Cryo-TEM) images were acquired using a cryogenic transmission electron microscope (FEI Talos F200C, Thermo Fisher Scientific, USA) operated at an accelerating voltage of 200 kV.

Single-crystal X-ray diffraction data were collected on a Bruker D8 Venture diffractometer (Bruker AXS GmbH, Germany).

The centrifugation processes were performed on a centrifuge manufactured by Flying Pigeon.

Quartz vials were purchased from Kuke Quartz Products Co., Ltd. (Donghai, Jiangsu), with dimensions of 15 cm (L) × 15 mm (OD) × 1 mm (WT). Magnetic stirrers (C-MAG HS7) were manufactured by IKA®.

Photoreactions were carried out in open-top, cylindrical quartz vials, using a dual-lamp UV irradiation setup. The reaction solution was irradiated in an open-top quartz vial positioned between two UV lamps: (1) Low-pressure mercury vapor lamp (long). Primary emission wavelengths: 254 nm. Manufacturer and model: Shanghai Jiguang Special Lighting Electrical Appliance Factory. Power: 28 W. Lamp dimensions: tubular, 45 cm. (2) Low-pressure mercury vapor lamp (short). Primary emission wavelength(s): 254 nm. Manufacturer and model: Guangzhou Xingchuang Electronics Co., LTD. Power: 40 W. Lamp dimensions: tubular, 15 cm.

Syringe filters (polyethersulfone, PES) were purchased from Shanghai ANPEL Laboratory Technologies Inc (SCAA-102, 13 mm × 0.22 μm).

Tensile tests of all specimens were performed using a universal material testing machine (SHIMADZU AGX-V, 10 kN, Shimadzu, Japan) at a strain rate of 50 mm/min for PP and 5 mm/min for PC. The dumbbell-shaped plastic specimens (PP and PC) were purchased from Dongguan Yingxinda Plastic Materials Co., Ltd. All specimens had an overall length of 150 mm and a parallel section with dimensions of 60.00 × 10.20 × 2.00 mm.

Synthesis and characterization of diamidophosphate (DAP):[1]

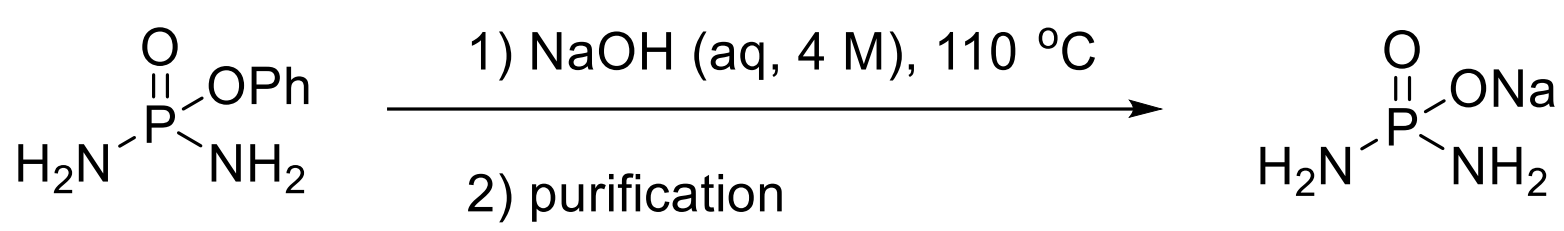

Phenyl phosphorodiamidate (1.0 g, 1.0 equiv.) was suspended in a 4 M sodium hydroxide solution (3 mL, 2.0 equiv.) and stirred at 110 °C for 10 min. The reaction mixture was washed consecutively with dichloromethane (10 mL × 2) and ethyl acetate (10 mL × 2). The aqueous phase was concentrated, and cold ethanol was added dropwise under vigorous stirring. The resulting precipitate was filtered and dried to afford the target product.

The results of NMR and MS analyses are shown in Figure S1 and S2. ^{31}P NMR (162 MHz, D_2O) δ 13.89 (s)· MS (ESI$^+$): chemical formula: $H_5N_2O_2P$; calculated: *m/z* 96 (DAP); found: *m/z* 97 $[M+H]^+$, 193 $[2M+H]^+$, 289 $[3M+H]^+$, 407 $[4M+Na]^+$ and other minor sodium-adducted aggregates.

HRMS (ESI/Q-TOF) *m/z* calculated for $H_5N_2O_2P$ $[M+H]^+$ 97.0161, found: 97.0165; *m/z* calculated for $[M+Na]^+$ 118.9981, found: 118.9981; *m/z* calculated for $[2M+Na]^+$ 215.0069, found: 215.0077; *m/z* calculated for $[2M+H]^+$ 193.0250, found: 193.0254; *m/z* calculated for $[3M+Na]^+$ 311.0158, found: 311.0166 (Figure S3).

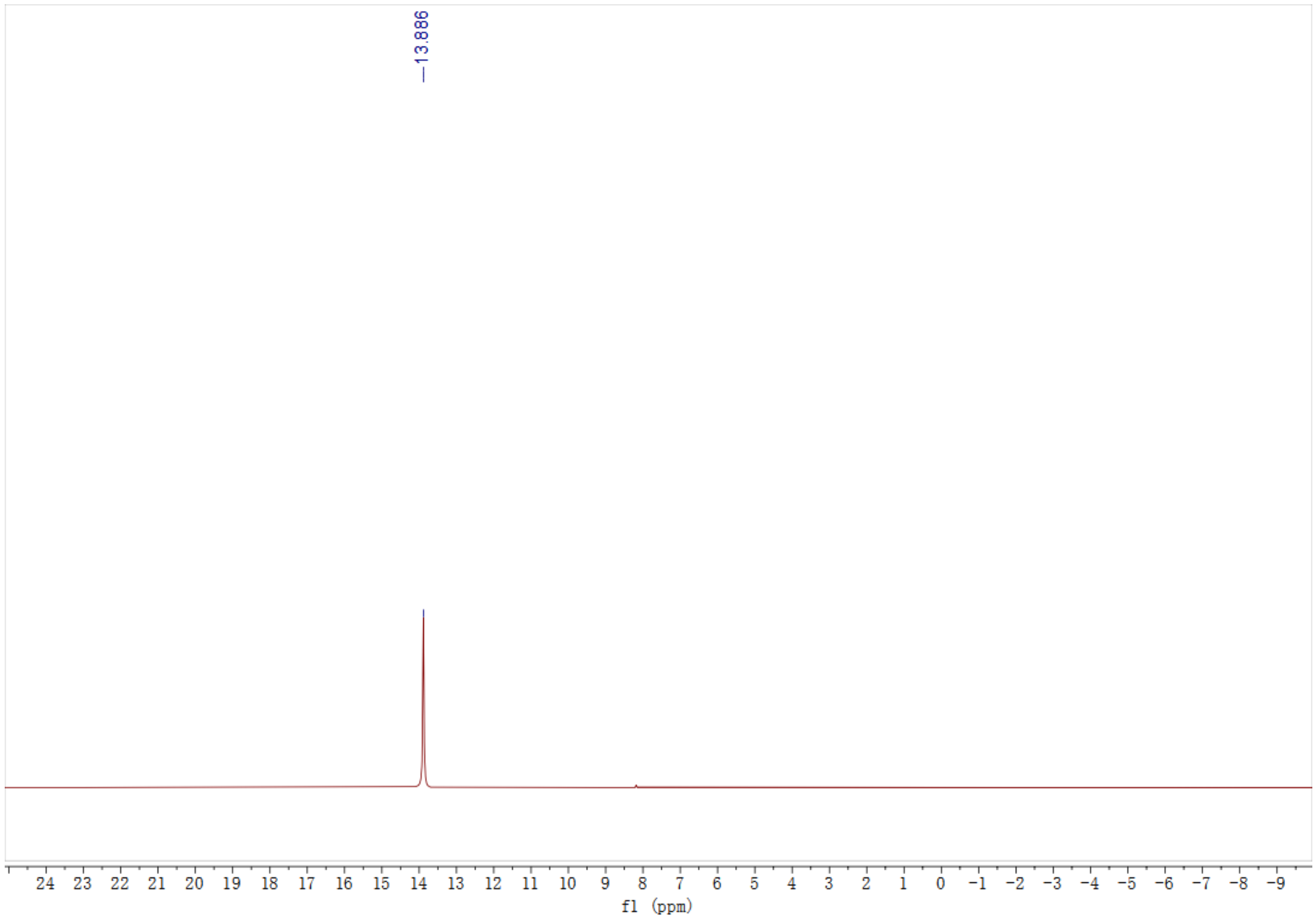

Figure S1. ^{31}P NMR spectrum of DAP (solvent: D_2O; 162 MHz).

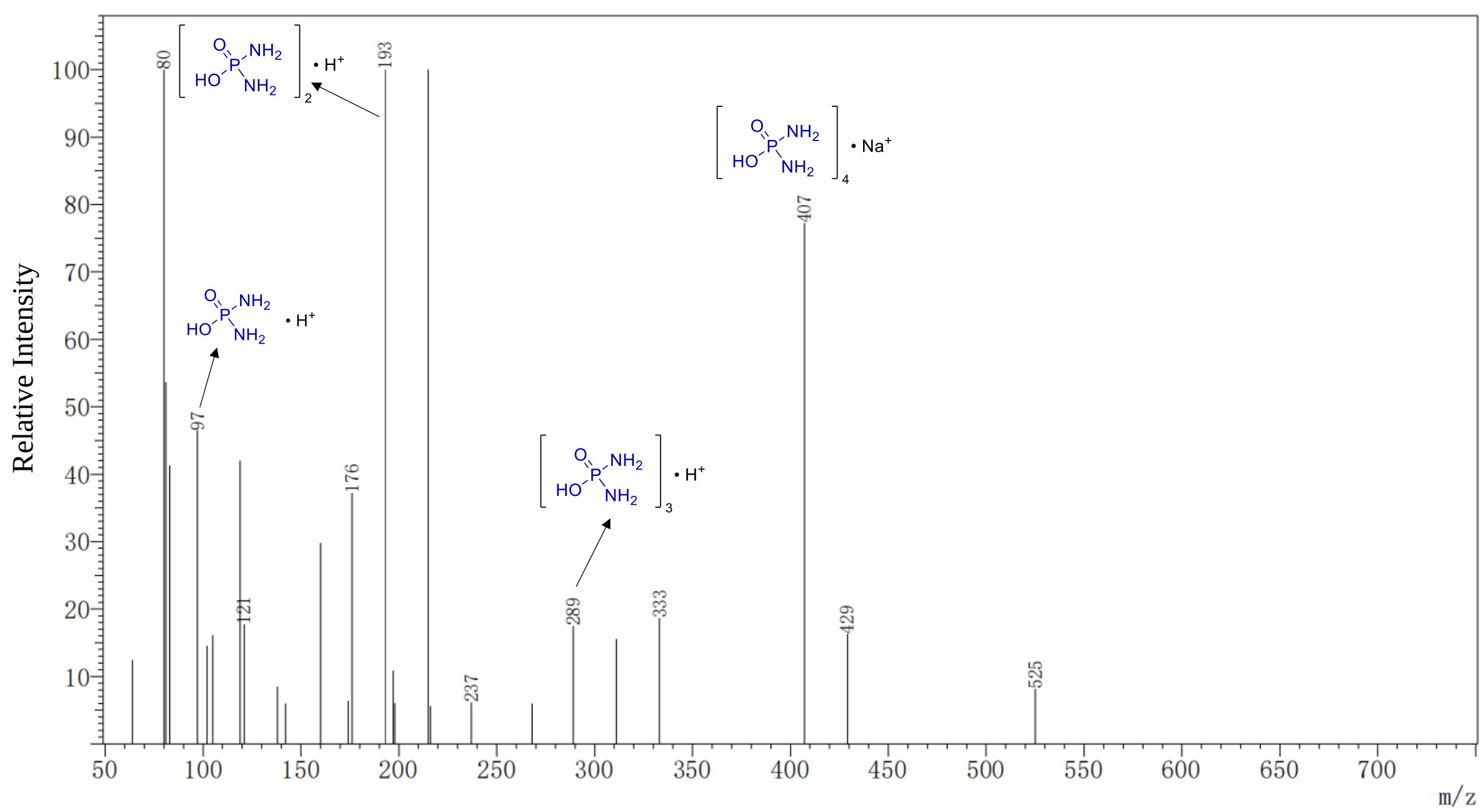

Figure S2. MS spectrum of DAP (concentration of sample: 0.03 M; injection volume: 20 µL).

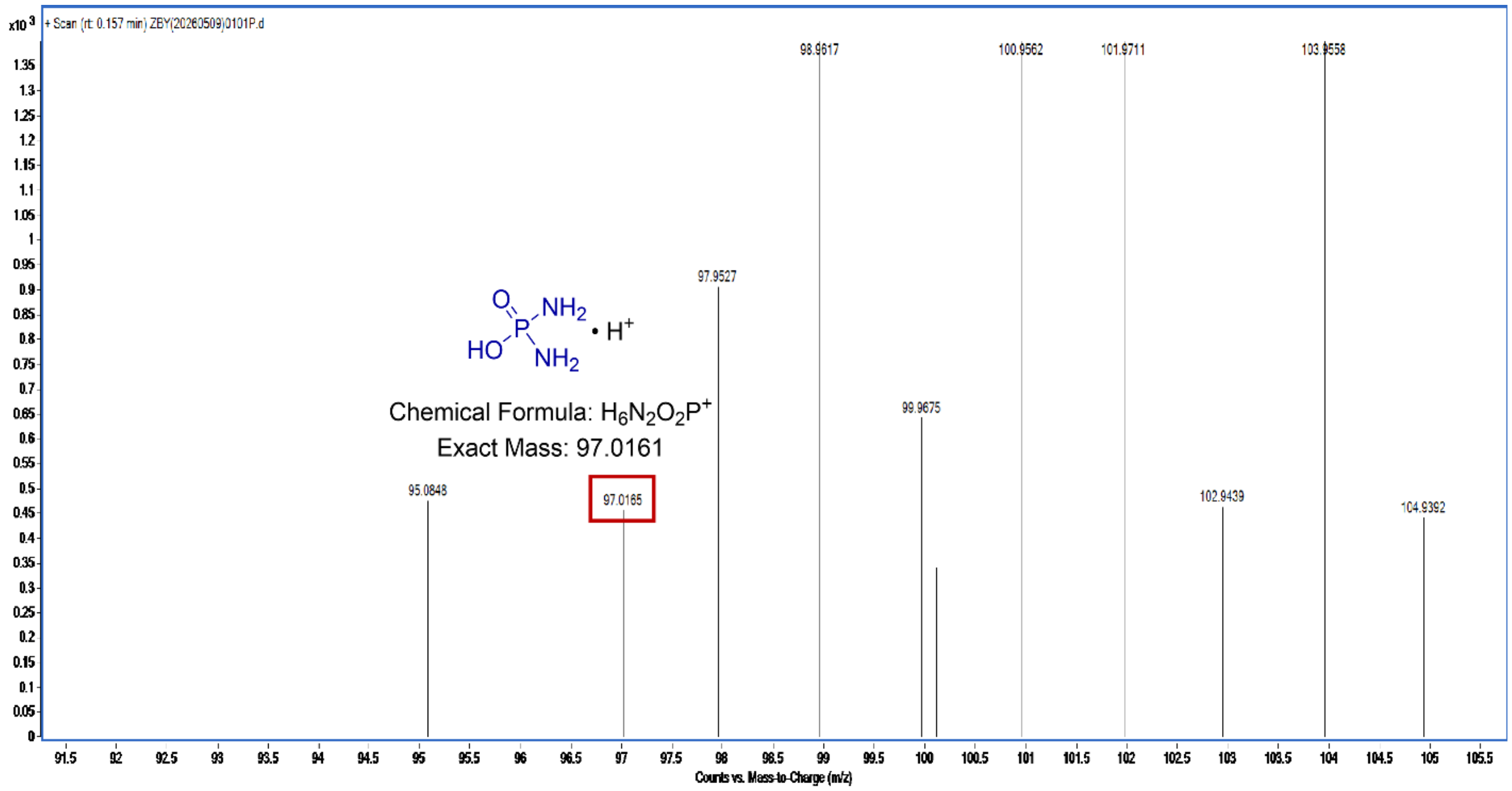
+ Scan (rt: 0.157 min) ZBY(20260509)0101P.d
x10^3
Chemical Formula: $H_6N_2O_2P^+$
Exact Mass: 97.0161
95.0848
97.0165
97.9527
98.9617
99.9675
100.9562
101.9711
102.9439
103.9558
104.9392
Counts vs. Mass-to-Charge (m/z)

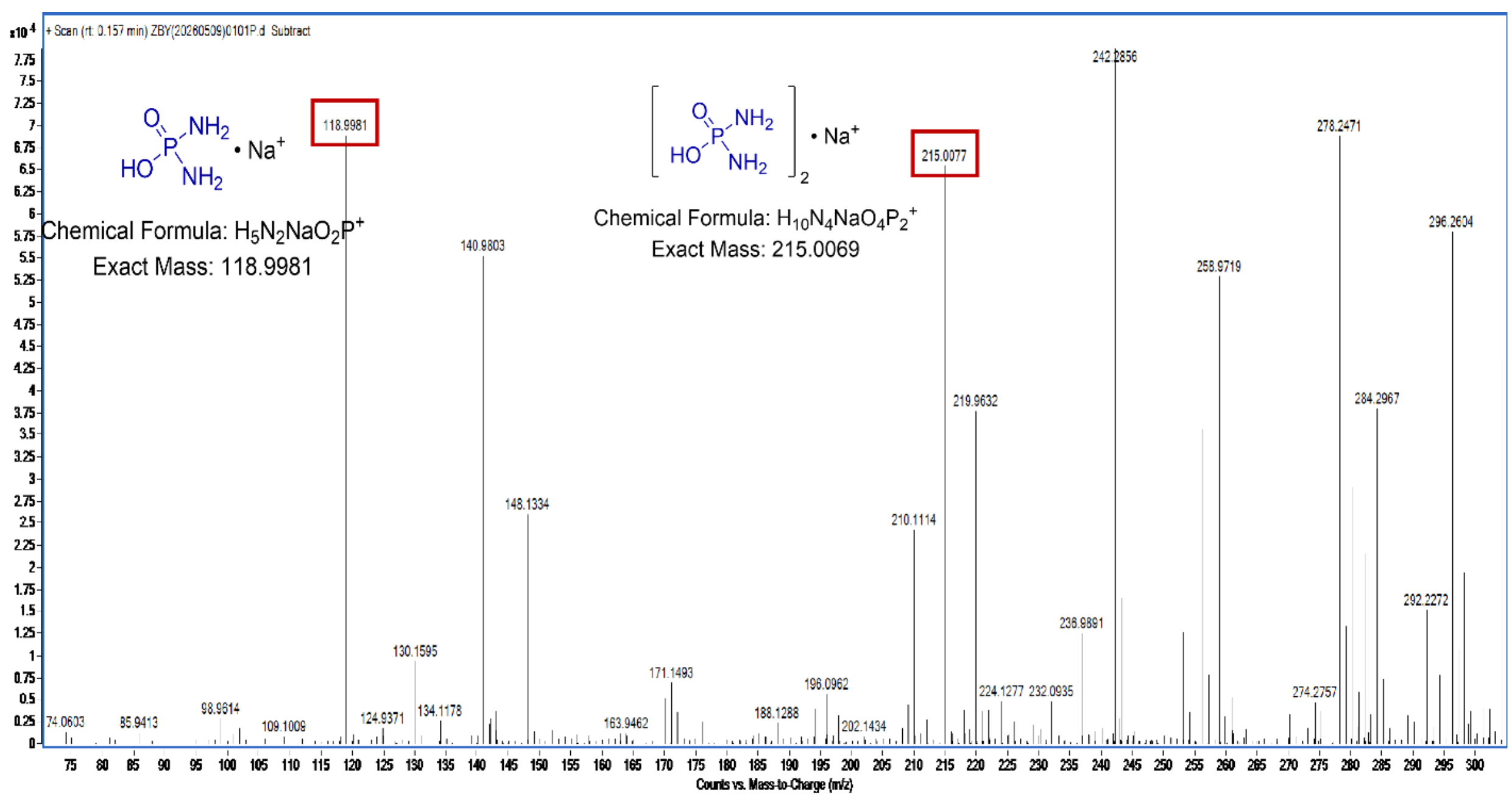
+ Scan (rt: 0.157 min) ZBY(20260509)0101P.d Subtract
x10^4
Chemical Formula: $H_5N_2NaO_2P^+$
Exact Mass: 118.9981
Chemical Formula: $H_{10}N_4NaO_4P_2^+$
Exact Mass: 215.0069
118.9981
215.0077
140.9803
148.1334
130.1595
210.1114
219.9632
242.2856
258.9719
278.2471
284.2967
292.2272
296.2604
238.9891
Counts vs. Mass-to-Charge (m/z)

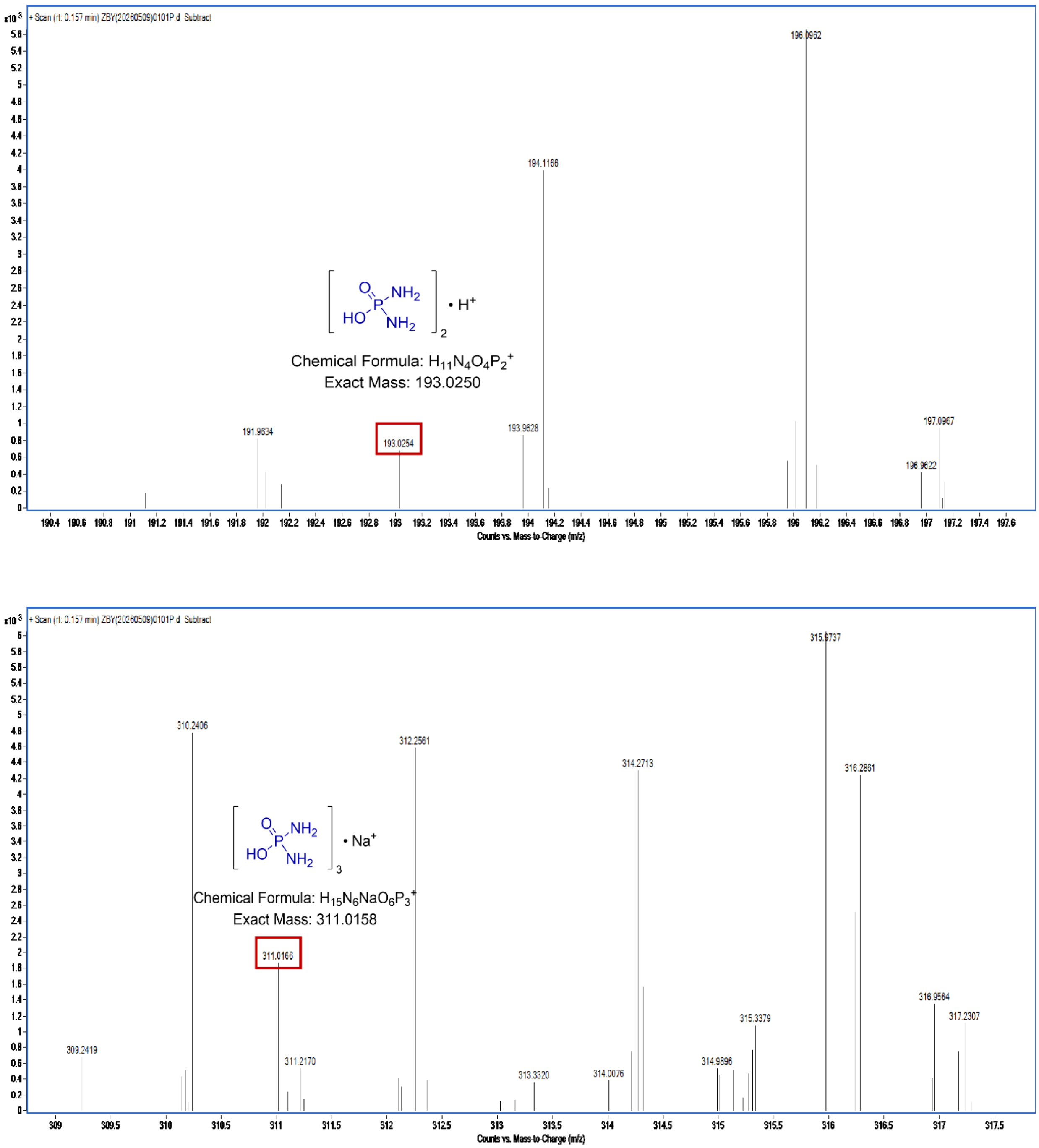

Figure S3. HRMS spectra of DAP with representative aggregate structures assigned.

Cryogenic transmission electron microscopy (Cryo-TEM) of DAP solution:

Cryogenic transmission electron microscopy (Cryo-TEM) was performed to directly visualize the supramolecular aggregates formed by DAP in aqueous solution (Figure S4).

Cryo-TEM specimens were prepared from a 0.03 M DAP aqueous solution using a standard plunge-freezing protocol. Briefly, a small aliquot of the sample solution was deposited onto a hydrophilized Cryo-TEM copper grid. After equilibration in an FEI Vitrobot chamber under controlled temperature and humidity, excess solution was removed by blotting with filter paper. The grid was then rapidly plunged into liquid ethane to vitrify the sample and stored in liquid nitrogen prior to Cryo-TEM observation.

The Cryo-TEM images revealed elongated one-dimensional bundle/rod-like assemblies with dimensions of approximately 100–500 nm in width and 600–5000 nm in length. Such morphology is consistent with the proposed ordered aggregation model and provides morphological evidence supporting aggregate-associated assembly in aqueous solution.

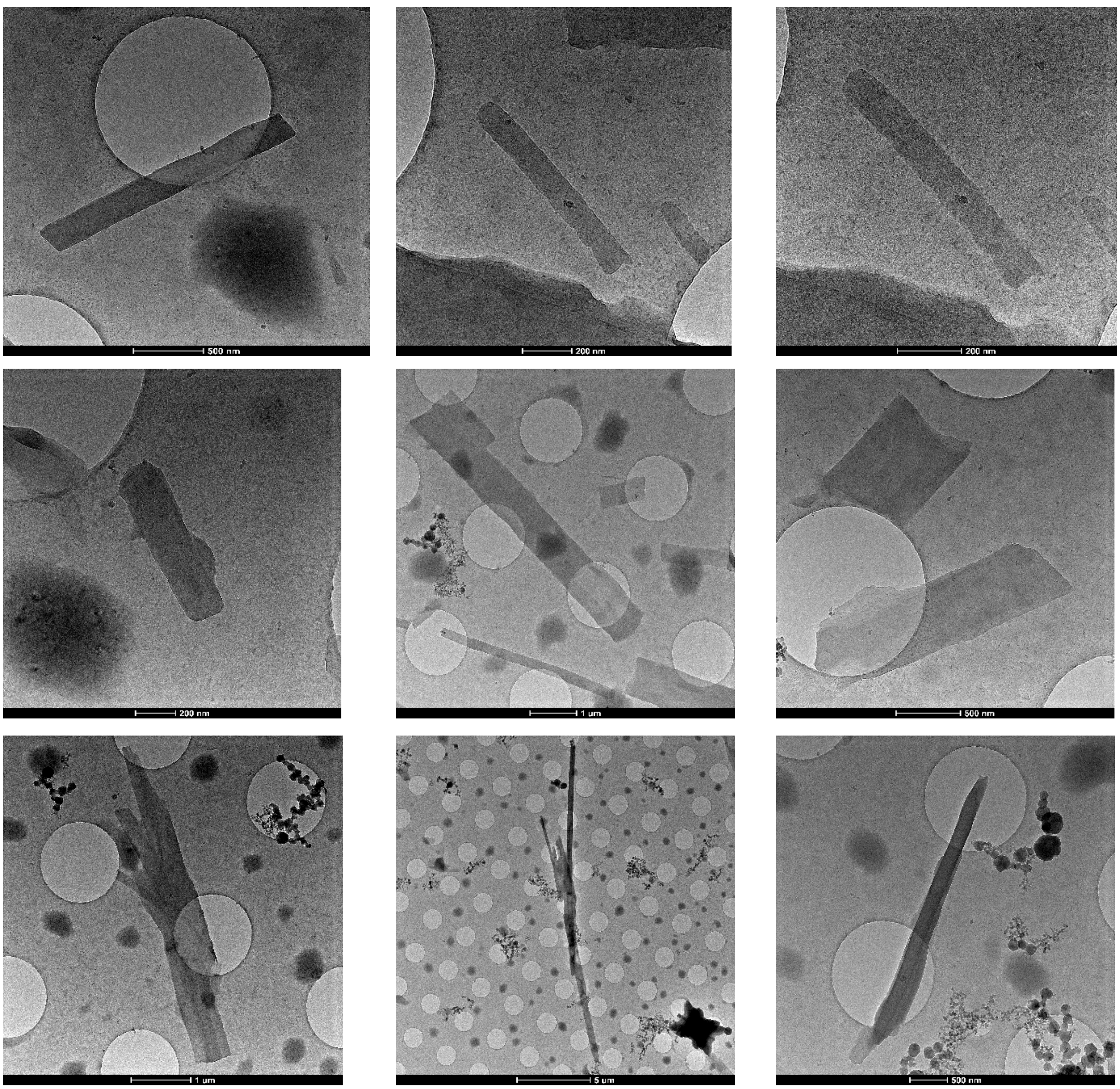

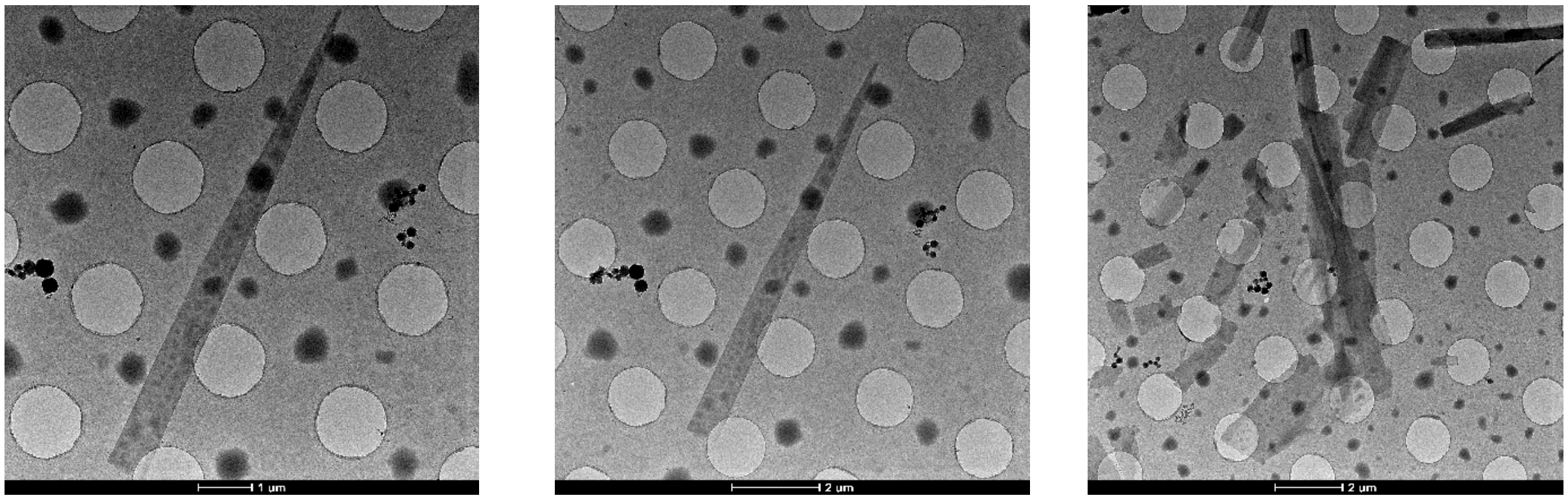

Figure S4. Cryo-TEM images of DAP aggregates in aqueous solution (black/gray rods: aggregates; white circles: ethane bubbles).

ICP-MS elemental analysis of DAP:

ICP-MS analysis was performed to evaluate the inorganic elemental composition and trace metal residues in the purified DAP sample (Table S1). As expected, P and Na were the dominant elements, corresponding to the phosphoramidate framework. In addition to these intrinsic elements, several trace elements, including Ca, K, Zn, Mg, Al, Fe, B, Cr, As and Ni, were detected at much lower levels. The relatively higher contents of Ca, K, Mg, Al, Fe, Zn and B are likely associated with trace inorganic residues introduced during sample handling and preparation, including the use of inorganic base, aqueous processing, glassware, filtration/centrifugation containers, and the acid-digestion/dilution procedure required for ICP-MS analysis. The presence of non-zero signals in the procedural blanks also indicates that part of the detected trace elements may originate from the analytical workflow rather than from the molecular structure itself.

Table S1. Elemental contents of the purified DAP sample determined by ICP-MS.

**Element**	**Mean Content / μg kg^{-1}**	**Comment**
P	1.67×10^8	sample component
Na	1.22×10^8	sample component
Ca	1.87×10^6	trace inorganic residue
K	1.99×10^5	trace inorganic residue
Zn	1.14×10^5	trace inorganic residue
Mg	4.53×10^4	trace inorganic residue
Al	1.82×10^4	trace inorganic residue
Fe	9.67×10^3	trace inorganic residue
B	5.94×10^3	likely glassware/reagent background
Cr	2.02×10^3	trace inorganic residue
As	1.17×10^3	trace inorganic residue
Ni	5.96×10^2	trace inorganic residue

Synthesis of and characterization of bis(dimethylamino)phosphorodiamidate (BDMDAP):[2]

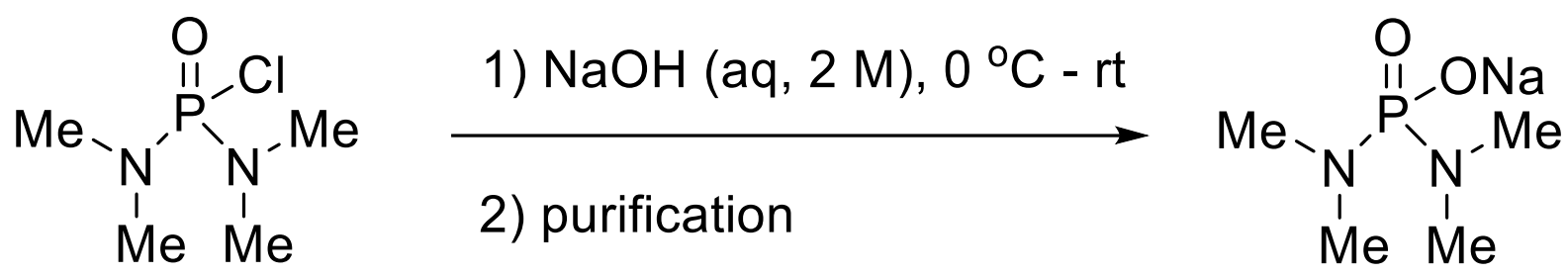

Bis(dimethylamino)phosphorochloridate (0.85 g, 5.0 mmol) was added dropwise (over ~10 minutes) to a stirred solution of aqueous NaOH (2 M, 5 mL) in an ice bath. The reaction mixture was stirred at room temperature for 30 minutes. The reaction mixture was then concentrated under vacuum to afford bis(dimethylamino)phosphorodiamidate (with sodium salt). The crude product was dissolved in EtOH and filtered. The filtrate was concentrated under vacuum to afford the crude BDMDAP. The obtained white solid was dissolved in H_2O (1 mL), followed by the addition of cold EtOH (20 mL) to induce crystallization. The recrystallized solid was collected by centrifugation and dried to afford purified BDMDAP as a white solid.

The NMR data of BDMDAP are shown in Figures S5 to S8.

^{1}H NMR (400 MHz, D_2O) δ 2.52 (d, J = 10.1 Hz, 12H, ^{31}P–^{1}H coupling through the P–N–C–H linkage); ^{13}C NMR (101 MHz, D_2O) δ 36.95, 36.93. ^{31}P NMR (162 MHz, D_2O) δ 18.64.

2D ^{1}H–^{13}C HSQC NMR spectrum was recorded to support the structural assignment of BDMDAP. A distinct cross-peak was observed at δH/δC = 2.44/37.0 ppm, corresponding to the directly bonded N–CH_3 proton and carbon resonances of the dimethylamino groups.

MS (ESI$^+$): chemical formula: $C_4H_{13}N_2O_2P$; calculated: m/z 152 (BDMDAP); found: m/z 153 $[M+H]^+$, 305 $[2M+H]^+$, 479 $[3M+Na]^+$, 653 $[4M–H+2Na]^+$ and other minor sodium-adducted aggregates (Figure S9).

HRMS (ESI/Q-TOF) m/z calculated for $C_4H_{13}N_2O_2P$ $[M+H]^+$ 153.0787, found: 153.0789; m/z calculated for $[M+Na]^+$ 175.0607, found: 175.0607; m/z calculated for $[2M+H]^+$ 305.1502, found: 305.1508; m/z calculated for $[2M+Na]^+$ 327.1322, found: 327.1322; m/z calculated for $[3M+H]^+$ 457.2217, found: 457.2228; m/z calculated for $[3M+Na]^+$ 479.2036, found: 479.2034; m/z calculated for $[4M+Na]^+$ 631.2751, found: 631.2752 (Figure S10).

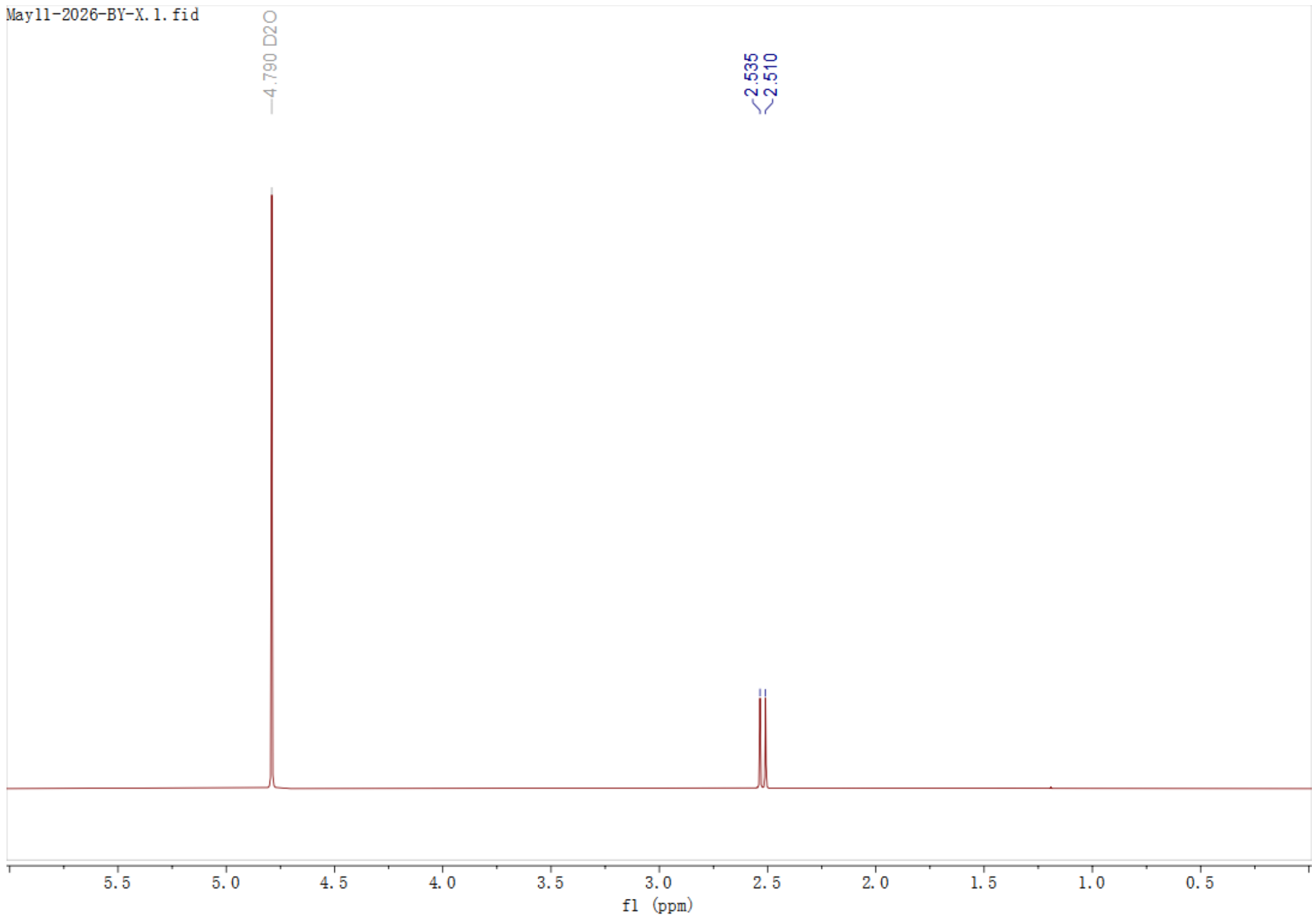

Figure S5. ^{1}H NMR spectrum of BDMDAP (solvent: D_2O; 400 MHz).

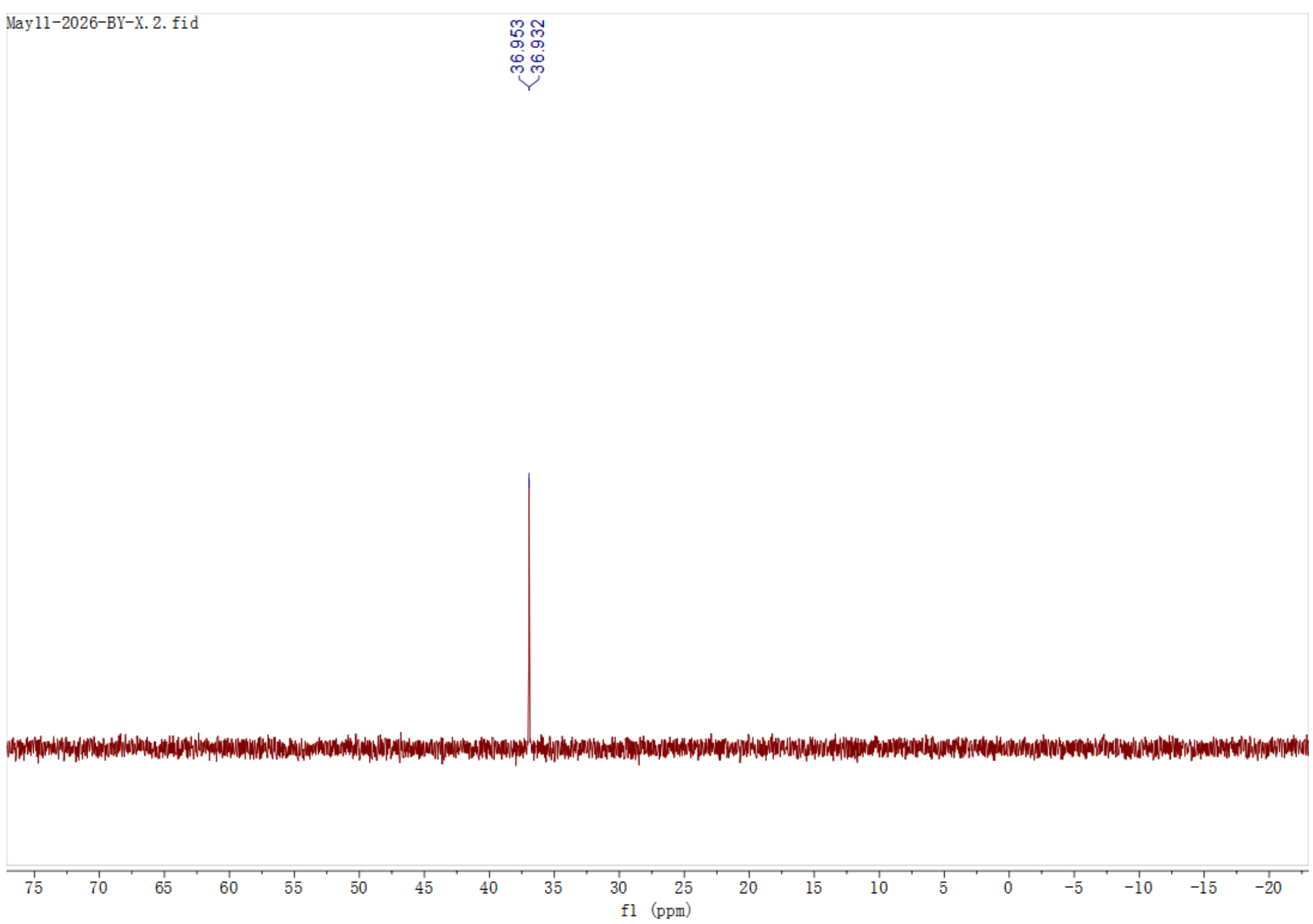

Figure S6. ^{13}C NMR spectrum of BDMDAP (solvent: D_2O; 101 MHz).

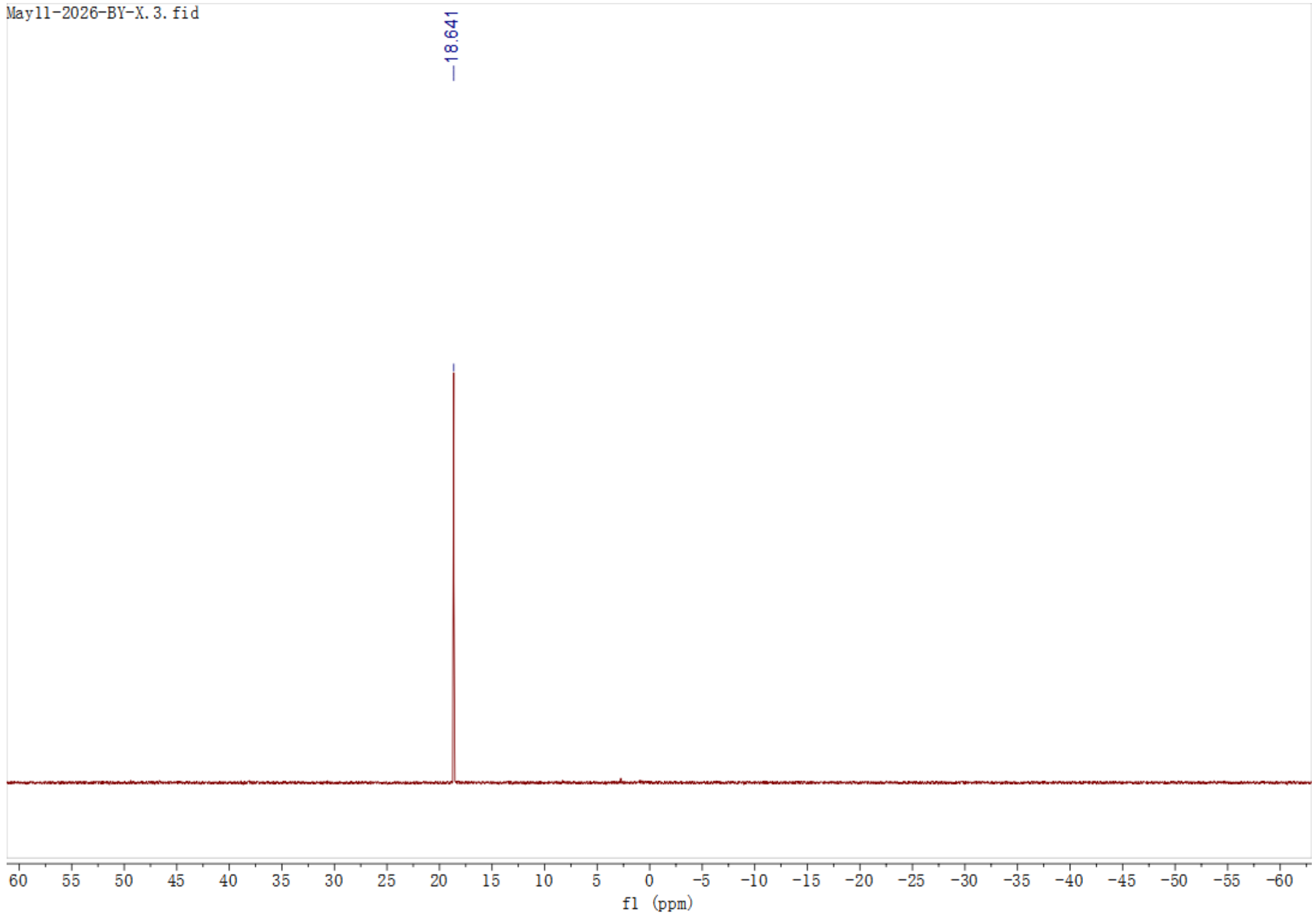

Figure S7. ^{31}P NMR spectrum of BDMDAP (solvent: D_2O; 162 MHz).

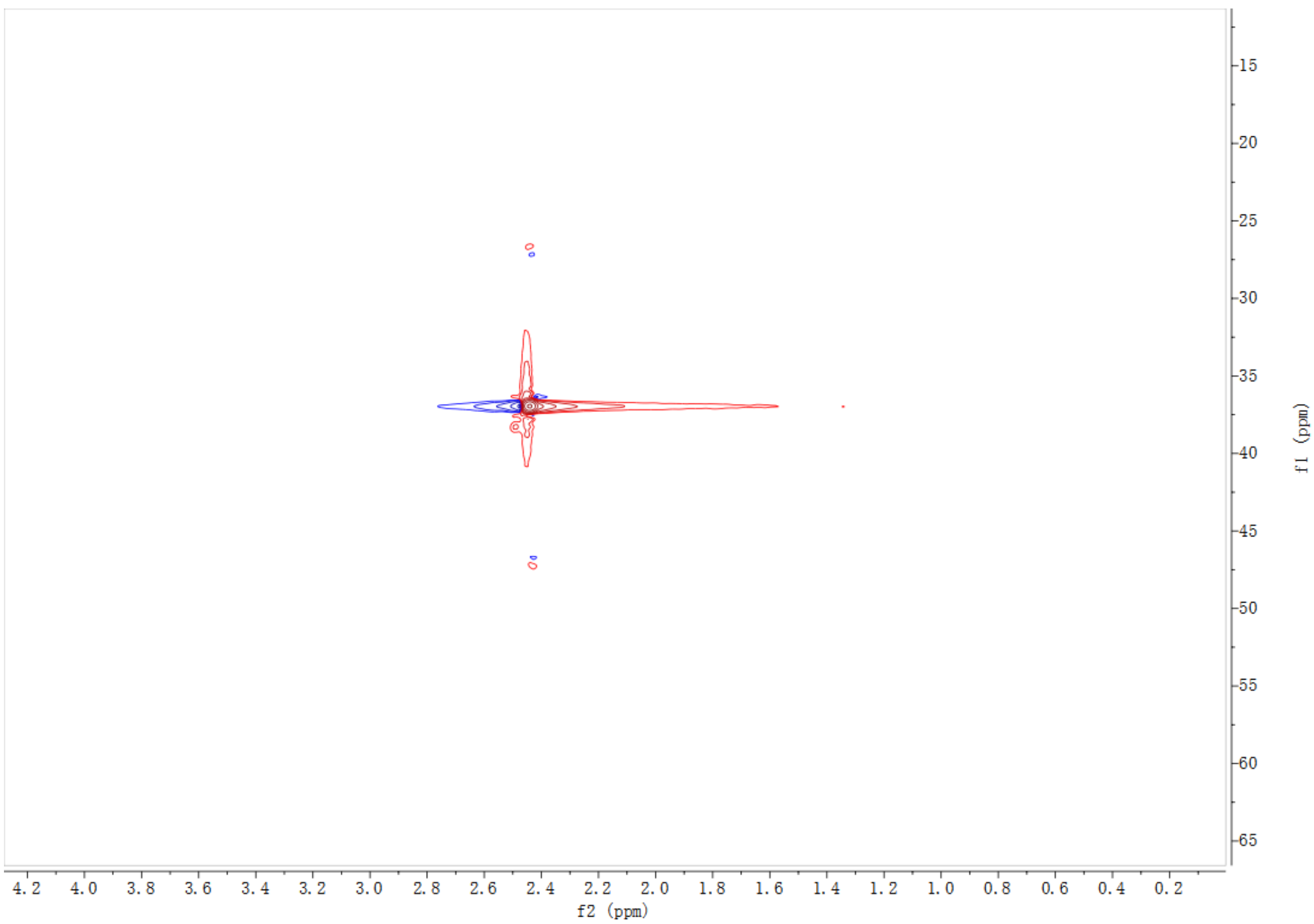

Figure S8. 2D NMR (^{1}H–^{13}C HSQC) spectrum of BDMDAP (solvent: D_2O).

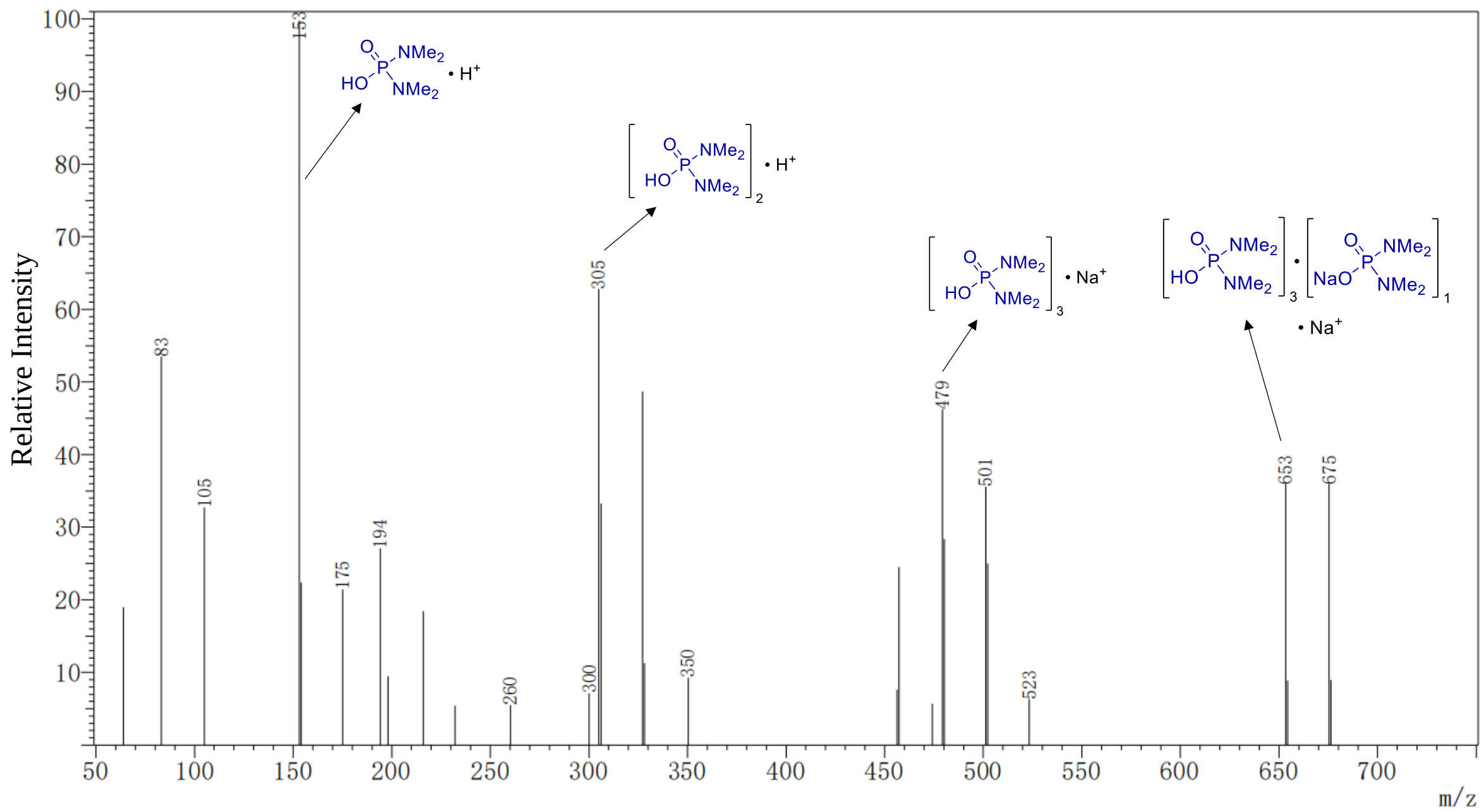

Figure S9. MS spectrum of BDMDAP (concentration of sample: 0.57 M; injection volume: 20 μL).

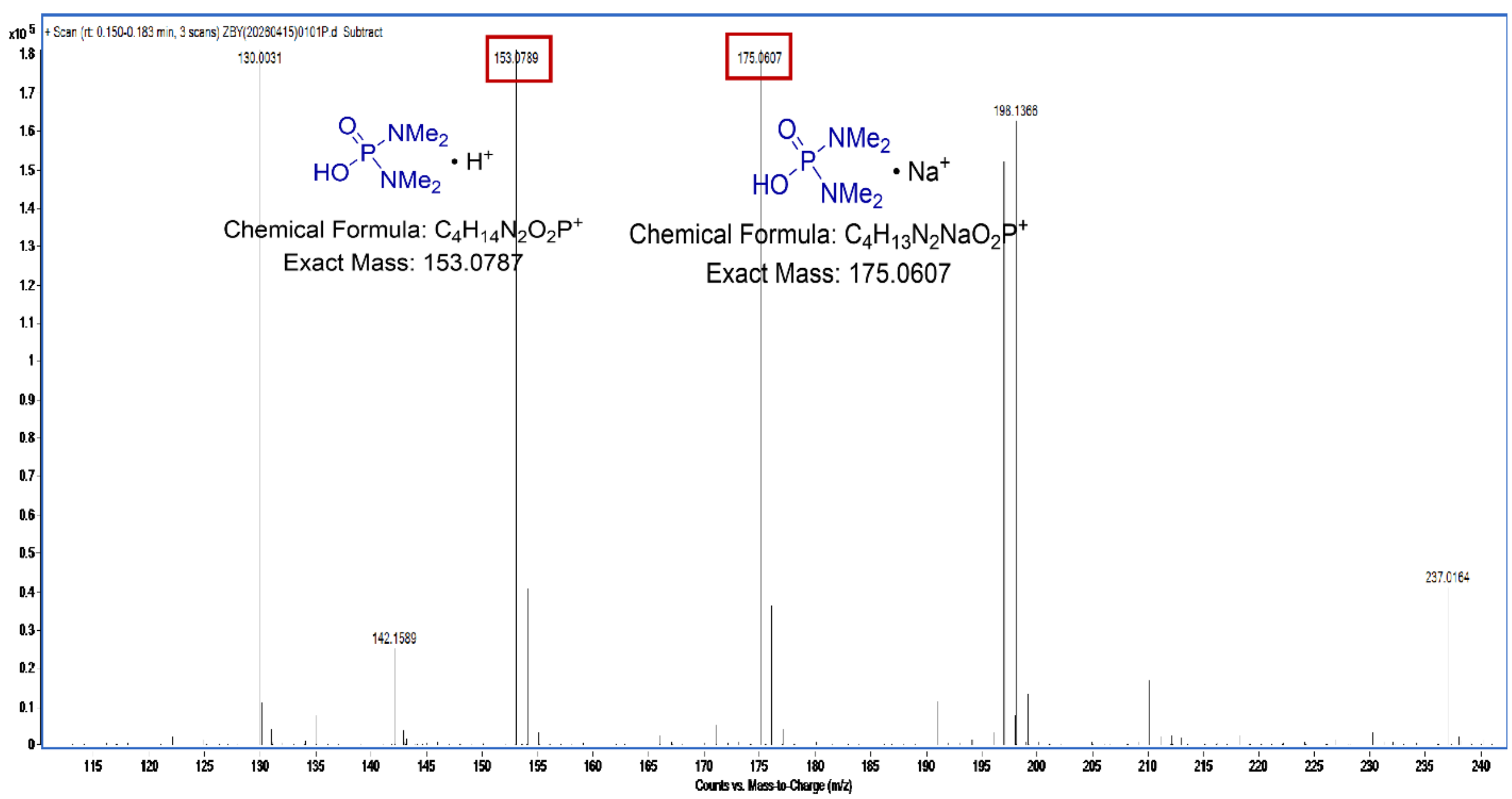

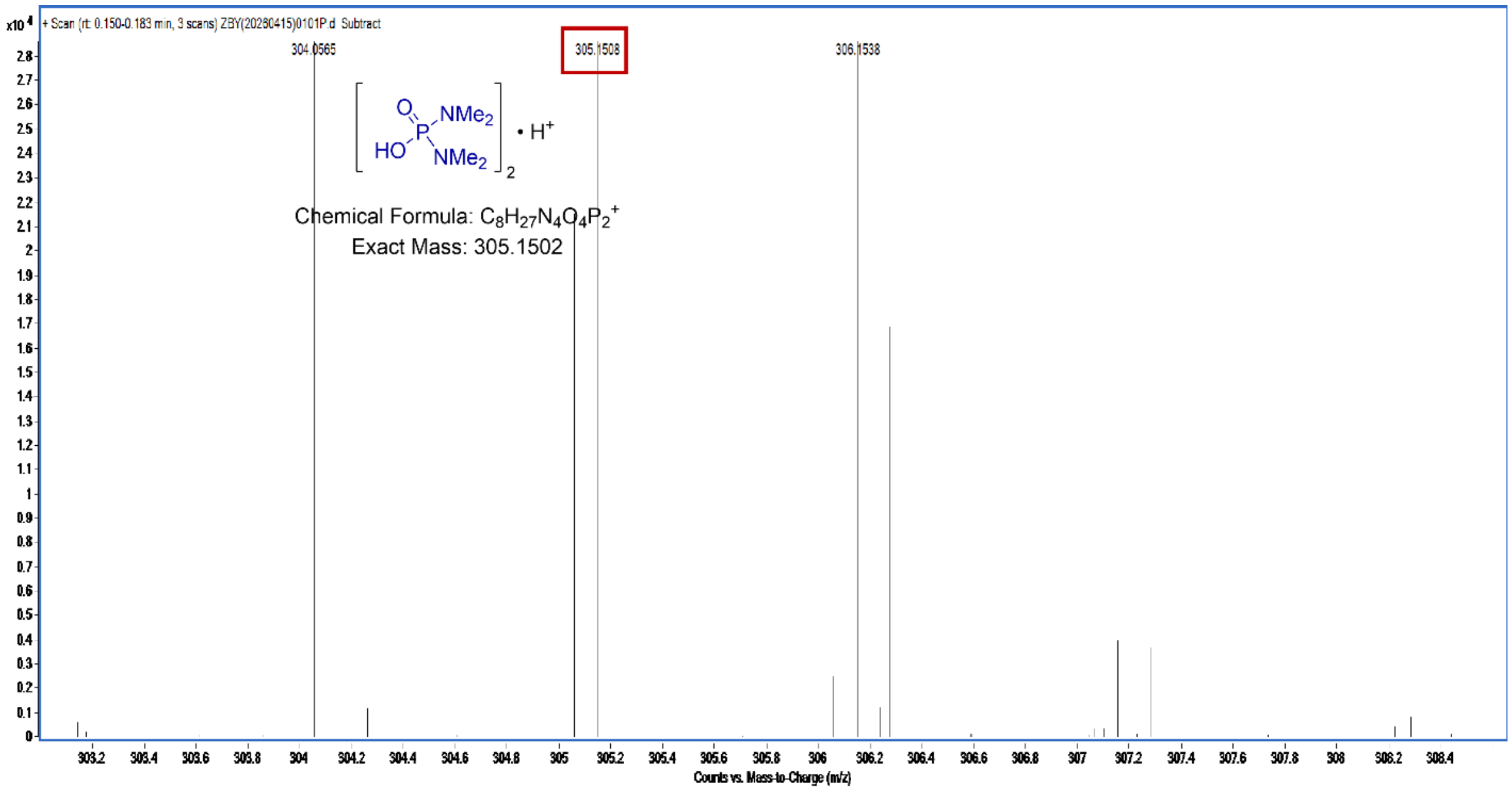

+ Scan (rt: 0.150-0.183 min, 3 scans) ZBY(20280415)0101P.d Subtract
304.0565
305.1508
306.1538
• H+
Chemical Formula: C8H27N4O4P2+
Exact Mass: 305.1502
Counts vs. Mass-to-Charge (m/z)

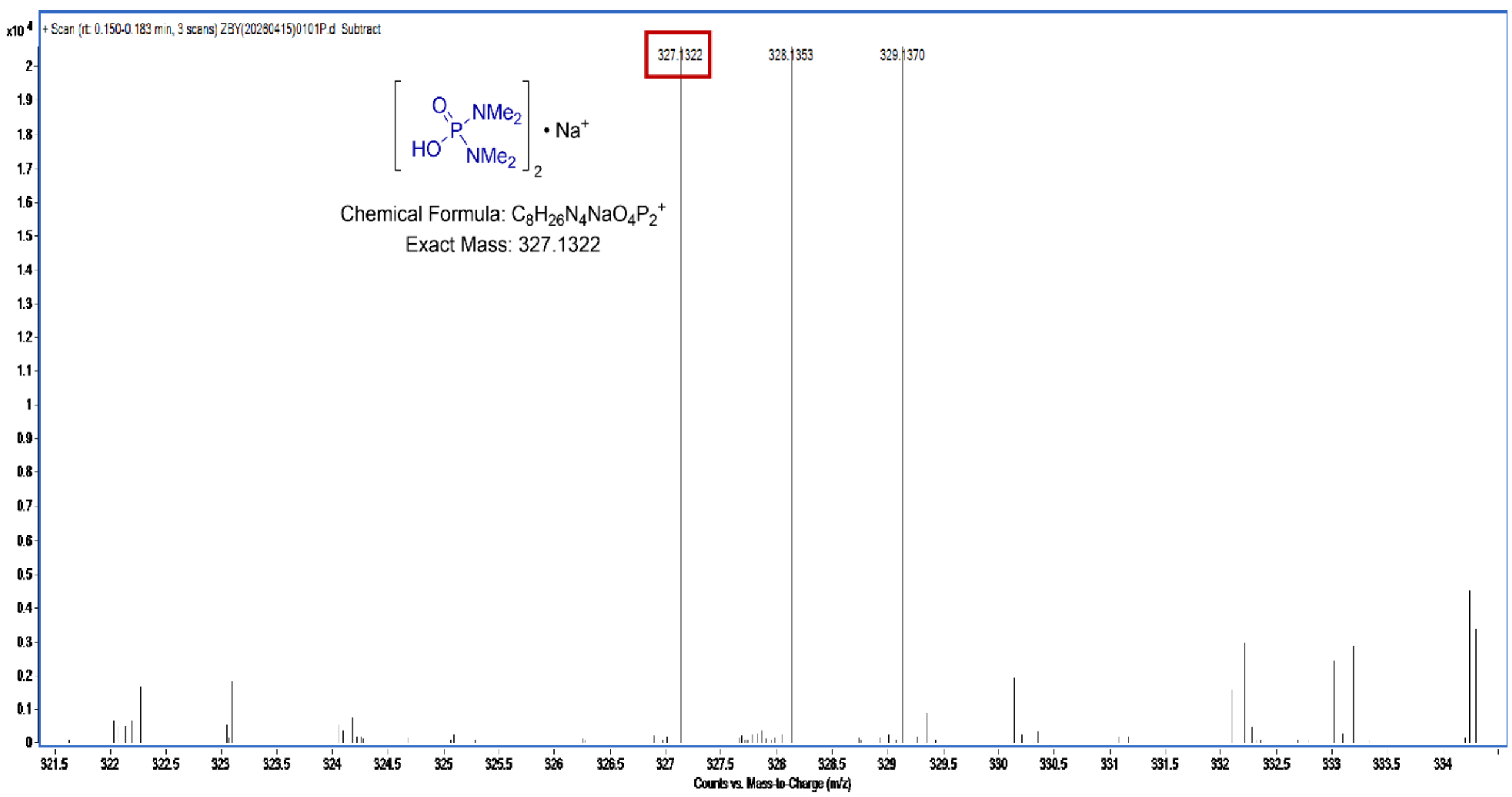

+ Scan (rt: 0.150-0.183 min, 3 scans) ZBY(20280415)0101P.d Subtract
327.1322
328.1353
329.1370
• Na+
Chemical Formula: C8H26N4NaO4P2+
Exact Mass: 327.1322
Counts vs. Mass-to-Charge (m/z)

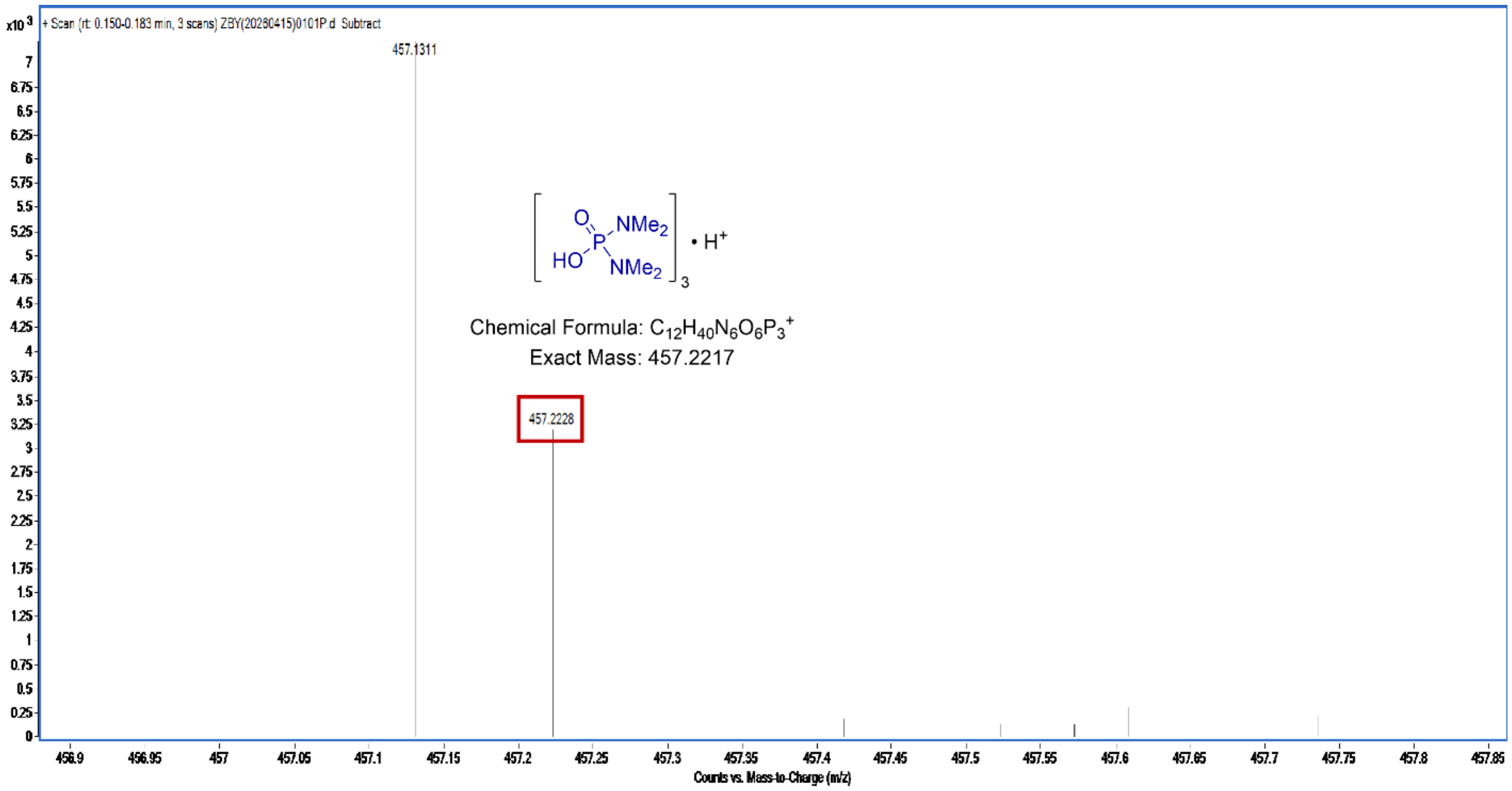
Chemical Formula: C12H40N6O6P3+
Exact Mass: 457.2217
457.1311
457.2228
Counts vs. Mass-to-Charge (m/z)

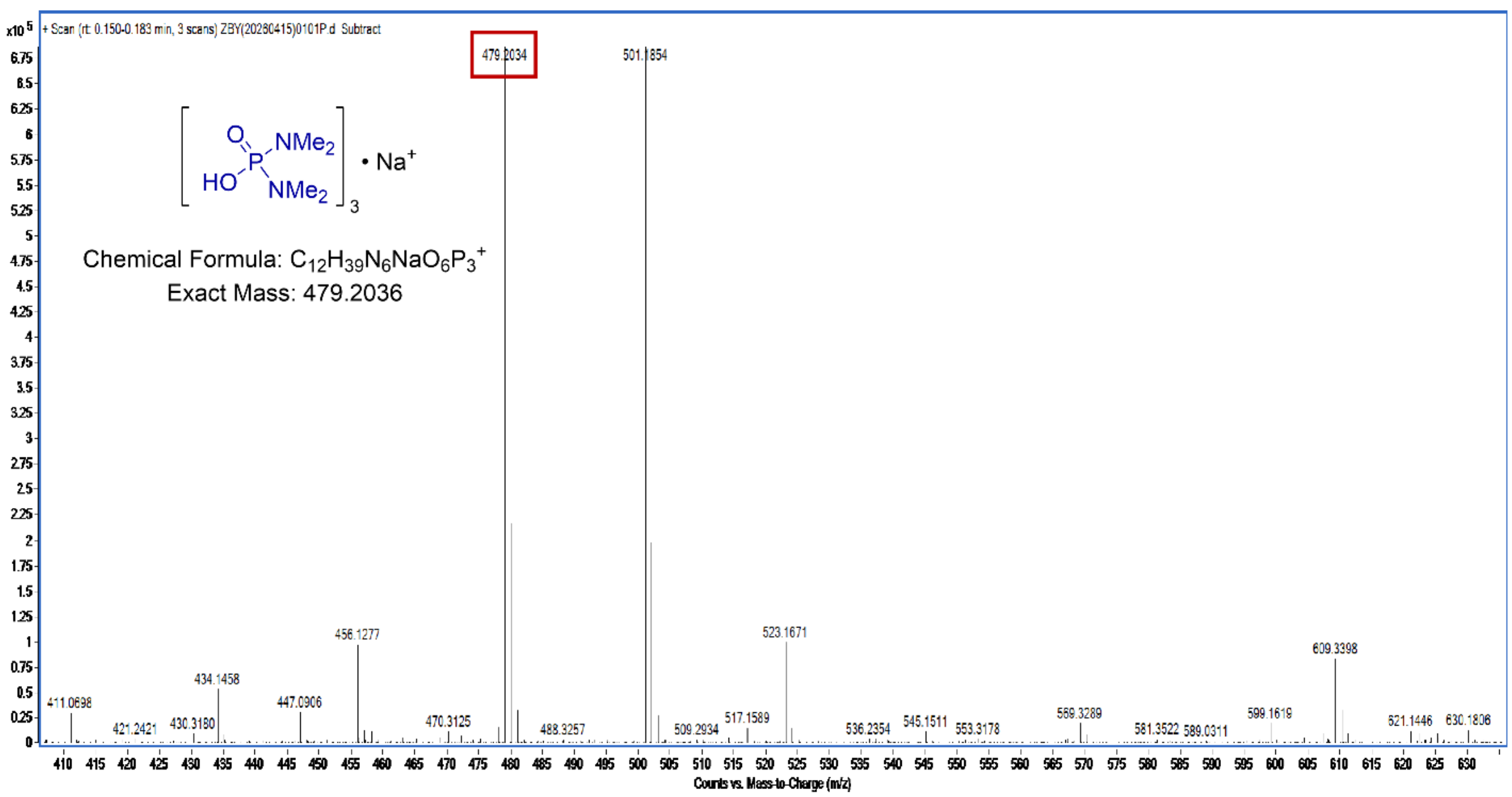
Chemical Formula: C12H39N6NaO6P3+
Exact Mass: 479.2036
479.2034
501.1854
Counts vs. Mass-to-Charge (m/z)

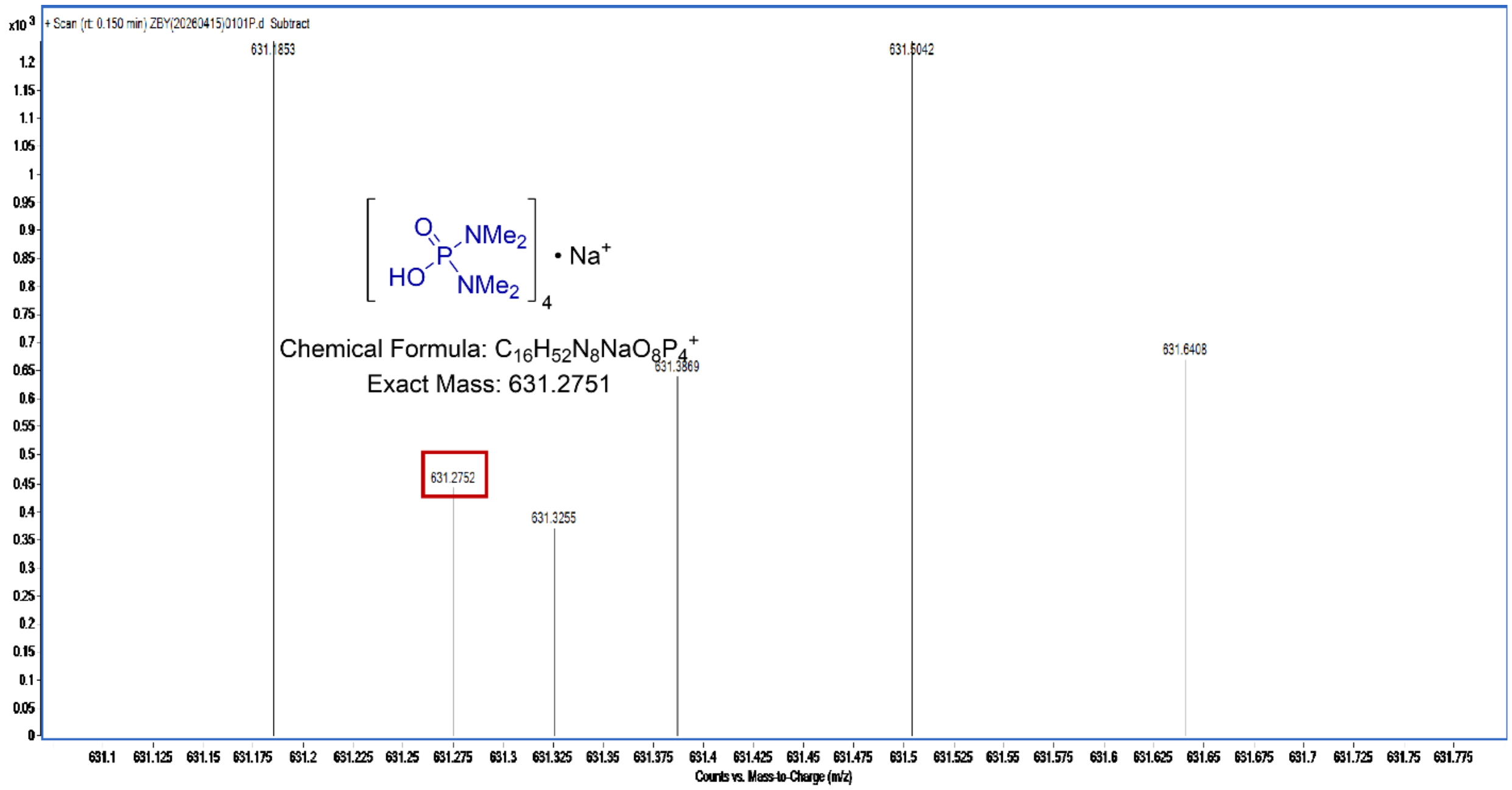

Figure S10. HRMS spectra of BDMDAP with representative aggregate structures assigned.

Concentration-dependent ^{1}H NMR spectra of BDMDAP:

A series of BDMDAP solutions with varying concentrations (0.01–0.72 M) were subjected to ^{1}H NMR analysis (400 MHz, D_2O). From 0.01 M to 0.02 M, the chemical shift of the methyl protons (-N(CH_3)$_2$) remained largely constant (Figure S11). From 0.02 M to 0.05 M, a notable upfield shift of the methyl signals was observed ($\Delta\delta \approx -0.06$ ppm), and continued with increasing concentration (from 0.09 M to 0.18 M, $\Delta\delta \approx -0.13$ ppm; from 0.18 M to 0.36 M, $\Delta\delta \approx -0.04$ ppm; from 0.36 M to 0.72 M, $\Delta\delta \approx -0.03$ ppm).

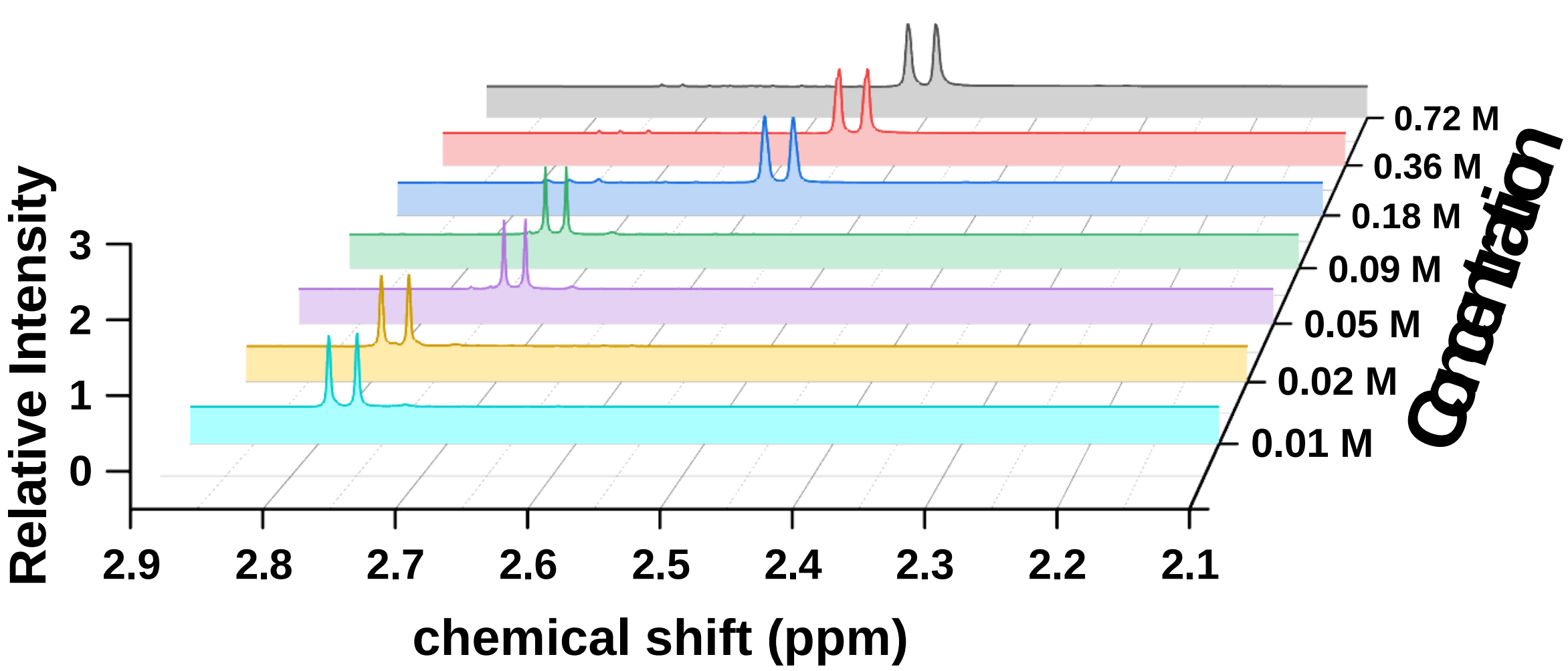

Figure S11. ^{1}H NMR spectra of BDMDAP at different concentrations (400 MHz, D_2O).

Single crystal X-ray diffraction data of BDMDAP:

Single crystals suitable for X-ray diffraction were selected for structural analysis (Figure S12 and Figure S13). Single-crystal X-ray diffraction data were collected on a CCD area detector using Mo Kα radiation (λ = 0.71073 Å) at 193 K with φ and ω scans. Data reduction was performed using SAINT V8.41, and a multi-scan absorption correction was applied using SADABS-2016/2. The structure was solved by SHELXT 2018/2 and refined by full-matrix least-squares on F^2 using SHELXL-2025/1 within the Olex2 1.5 interface. Non-hydrogen atoms were refined anisotropically, and hydrogen atoms were treated using constrained refinement procedures. BDMDAP crystallizes in the monoclinic crystal system with the C2/c space group. The refined empirical formula is $C_8H_{42}N_4O_{13}P_2Na_2$, corresponding to a hydrated sodium-containing crystalline structure.

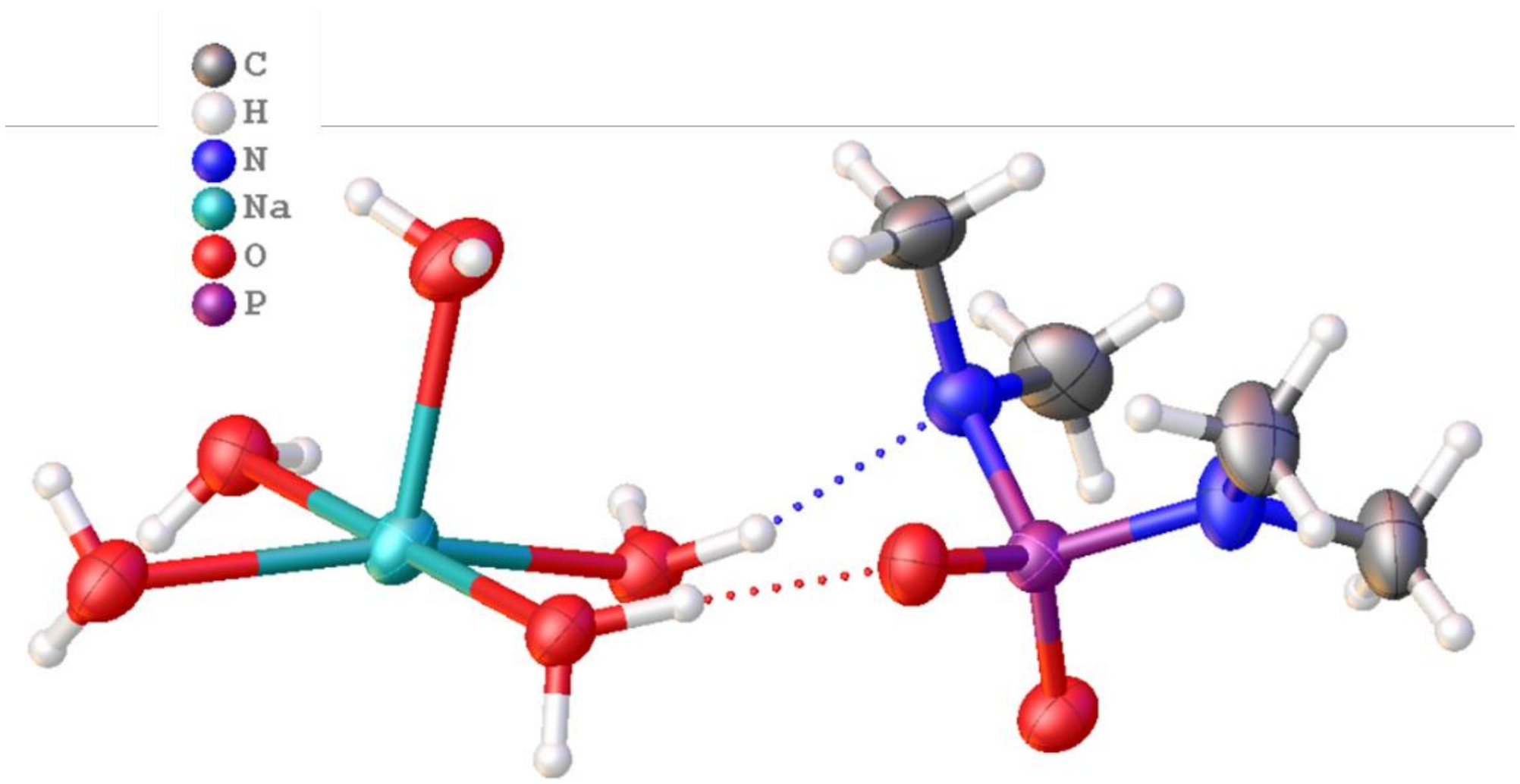

Figure S12. Single-crystal structure of BDMDAP showing the hydrated sodium-containing coordination environment with displacement ellipsoids. Dashed lines indicate selected intermolecular interactions.

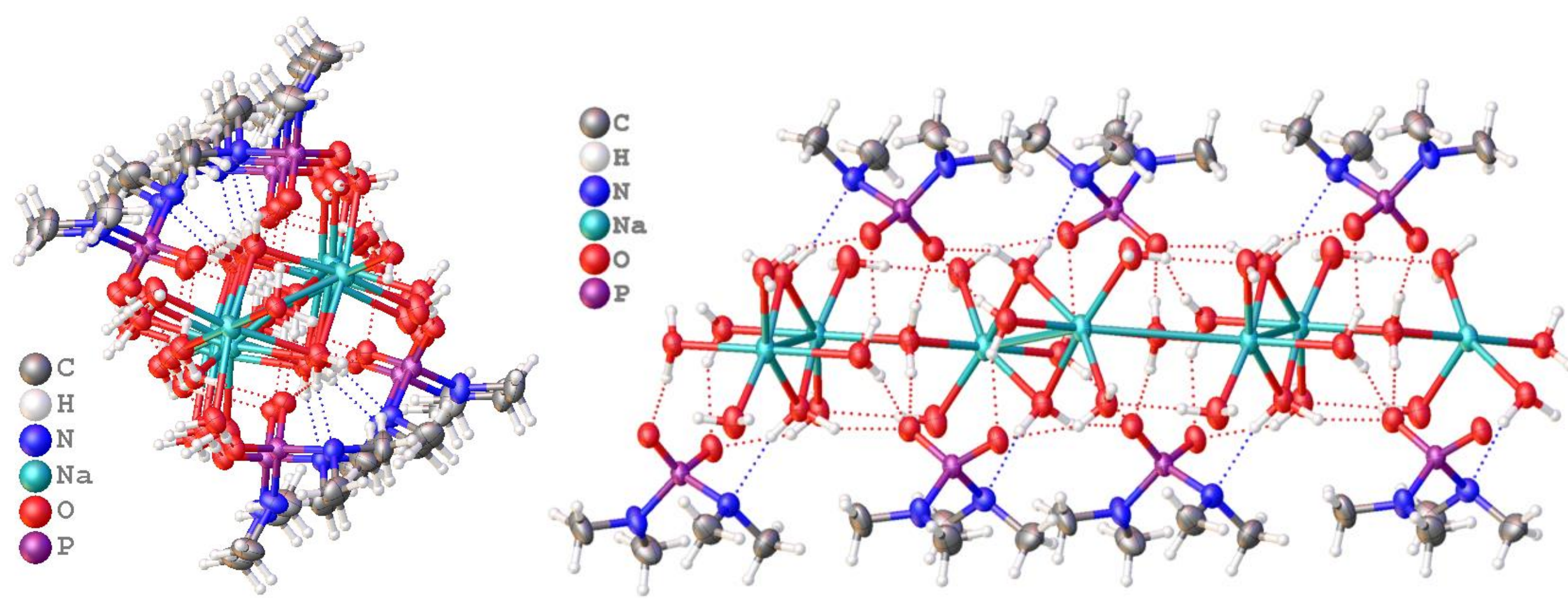

Figure S13. Crystal packing structure of BDMDAP showing the extended hydrated sodium coordination network. Dashed lines indicate selected intermolecular interactions.

Table S2. Crystal data and structure refinement parameters for BDMDAP.

**Parameter**	**BDMDAP**
Empirical formula	$C_8H_{42}N_4O_{13}P_2Na_2$
Formula weight	510.37
Temperature/K	193.00
Crystal system	Monoclinic
Space group	C2/c
a/Å	26.712(11)
b/Å	8.581(3)
c/Å	11.888(5)
α/°	90
β/°	107.497(16)
γ/°	90
Volume/Å^3	2598.9(18)
Z	4
ρcalc/g cm^{-3}	1.304
μ/mm^{-1}	0.257
F(000)	1096
Crystal size/mm^3	0.15 × 0.13 × 0.12
Crystal colour and habit	Colourless block
Radiation	Mo Kα
Wavelength/Å	0.71073
Absorption correction	Multi-scan
Tmin, Tmax	0.4871, 0.7455
θ range for data collection/°	2.505–27.634
Index ranges	$-34 \leq h \leq 34$, $-11 \leq k \leq 7$, $-15 \leq l \leq 14$
Reflections collected	8684
Independent reflections	2995
Observed reflections	1990
Rint	0.0788
Completeness to θmax	0.989
Data/restraints/parameters	2995 / 0 / 139
Goodness-of-fit on F^2	1.020
Final R indexes [I > 2σ(I)]	R1 = 0.0602, wR2 = 0.1387
Final R indexes [all data]	R1 = 0.0996, wR2 = 0.1622
Largest diff. peak/hole/e Å^{-3}	0.414 / −0.442
CCDC number	2556505

Table S3. Selected bond lengths and bond angles for BDMDAP.

Selected bond lengths

**Bond**	**Length/Å**
P1–O1	1.496(2)
P1–O2	1.497(2)
P1–N1	1.693(3)
P1–N2	1.655(3)
N1–C3	1.468(4)
N1–C4	1.477(5)
N2–C1	1.448(5)
N2–C2	1.446(4)
Na1–O3	2.433(3)
Na1–O3^{i}	2.483(3)
Na1–O4	2.396(2)
Na1–O5	2.335(3)
Na1–O6	2.433(2)
Na1–O7	2.425(3)

Symmetry code: (i) −x + 1, −y + 1, −z + 1.

Selected bond angles

**Angle**	**Degree/°**
O1–P1–O2	118.32(13)
O1–P1–N1	105.02(13)
O1–P1–N2	109.08(15)
O2–P1–N1	106.36(14)
O2–P1–N2	107.86(13)
N2–P1–N1	109.98(15)
C3–N1–P1	116.5(3)
C4–N1–P1	115.4(2)
C1–N2–P1	121.8(2)
C2–N2–P1	123.1(2)

Crystallographic data deposition:

Crystallographic data for BDMDAP have been deposited with the Cambridge Crystallographic Data Centre as supplementary publication no. CCDC 2556505. Copies of the data can be obtained free of charge from the CCDC via www.ccdc.cam.ac.uk/structures/.

Concentration-dependent UV-Vis absorption of DAP:

A series of DAP solutions were prepared, with concentrations of 0.001 M, 0.003 M, 0.005 M, 0.006 M, 0.007 M, 0.008 M, 0.009 M, 0.01 M, 0.02 M, and 0.03 M. UV-Vis spectra were recorded for these solutions (Figure S14). The results demonstrated that a sharp new band at 235 nm emerged abruptly at 0.01 M. Moreover, another sharp new absorption band was observed at 287 nm starting from 0.02 M, suggesting the emergence of higher-order aggregates. CAC interval: 0.009–0.01 M (First CAC, 235 nm), 0.01–0.02 M (Second CAC, 287 nm) (Figure S15). The curve of extinction coefficient as a function of wavelength shows clear concentration-dependent spectral reshaping (Figure S16).

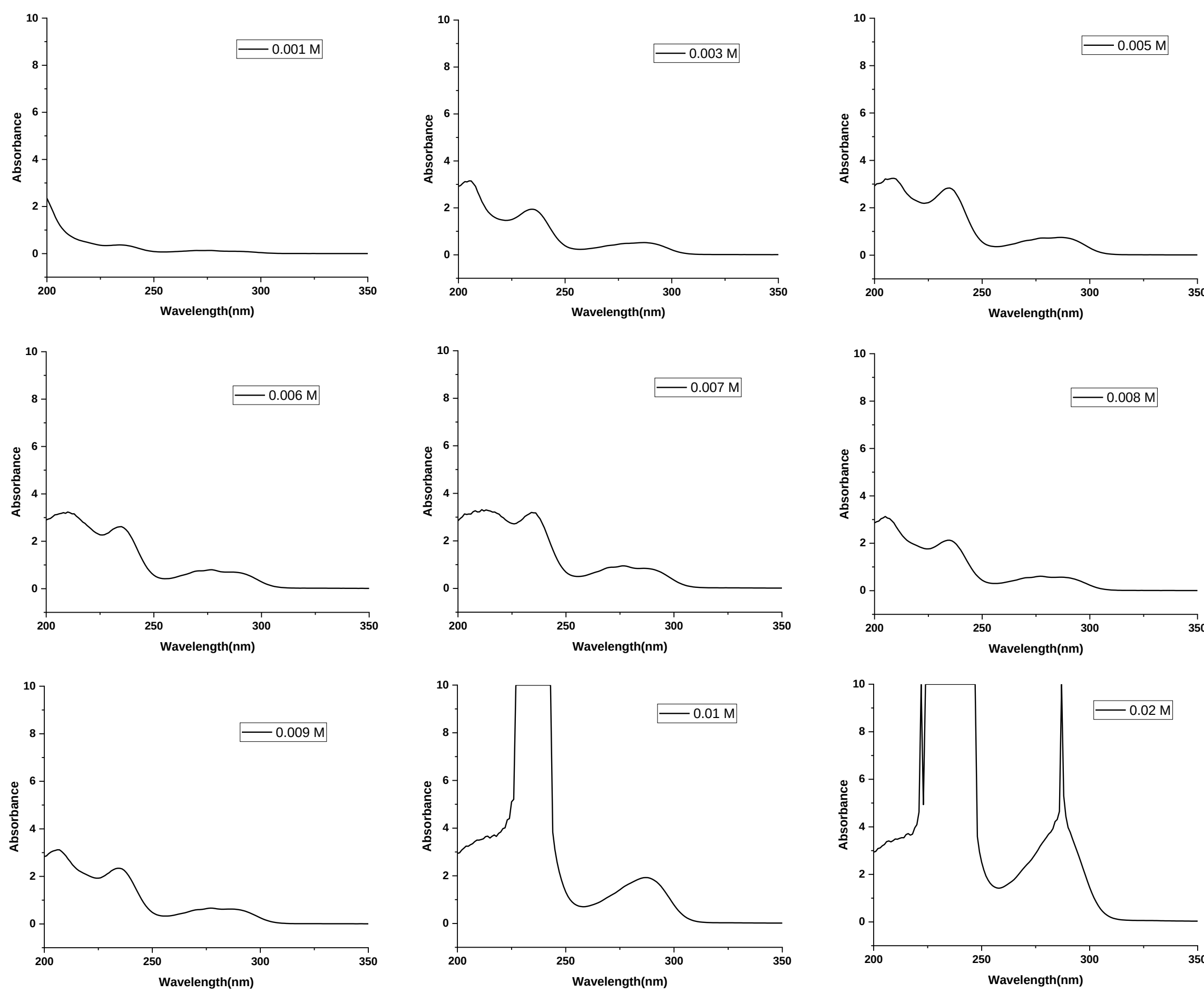

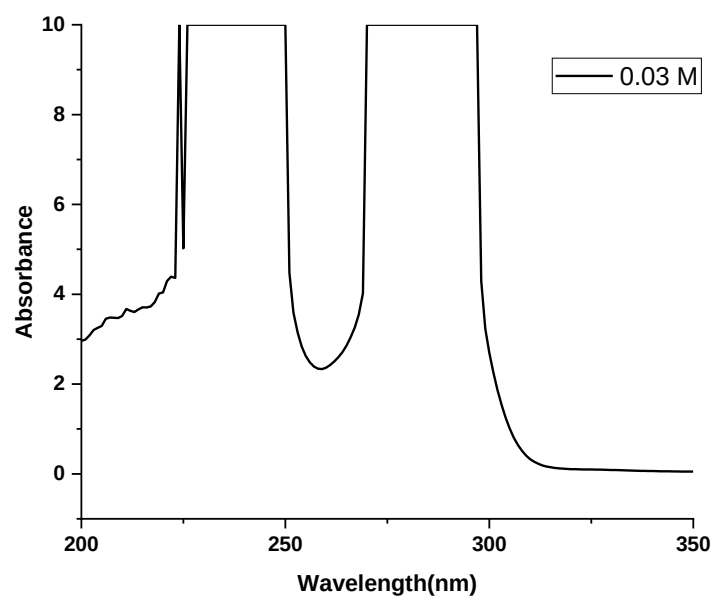

Figure S14. UV-Vis spectra of DAP at different concentrations.

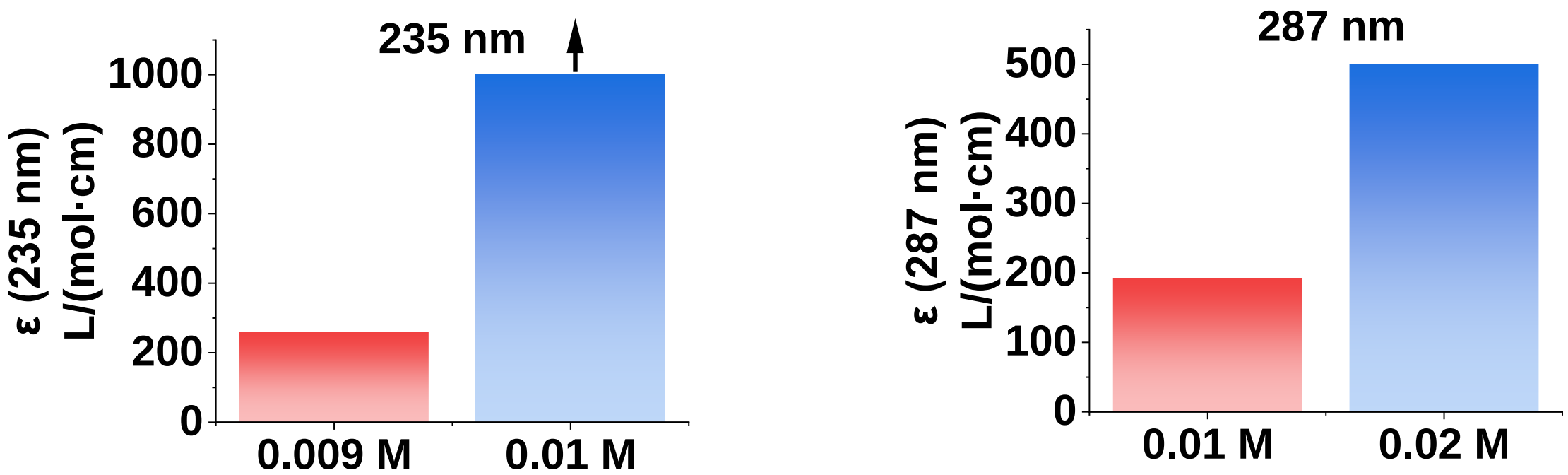

Figure S15. Determination of the AIA-related transition regions of DAP based on extinction coefficient changes. (**A**) Extinction coefficient at 235 nm, showing a pronounced increase from 0.009 M to 0.01 M (the upper arrow indicates that the actual value may be higher, as the absorbance is near or above the detection limit). (**B**) Extinction coefficient at 287 nm, showing a pronounced increase from 0.01 M to 0.02 M. The transition at 235 nm was used to estimate the first CAC range of DAP, while the change at 287 nm suggests further aggregate evolution at higher concentration.

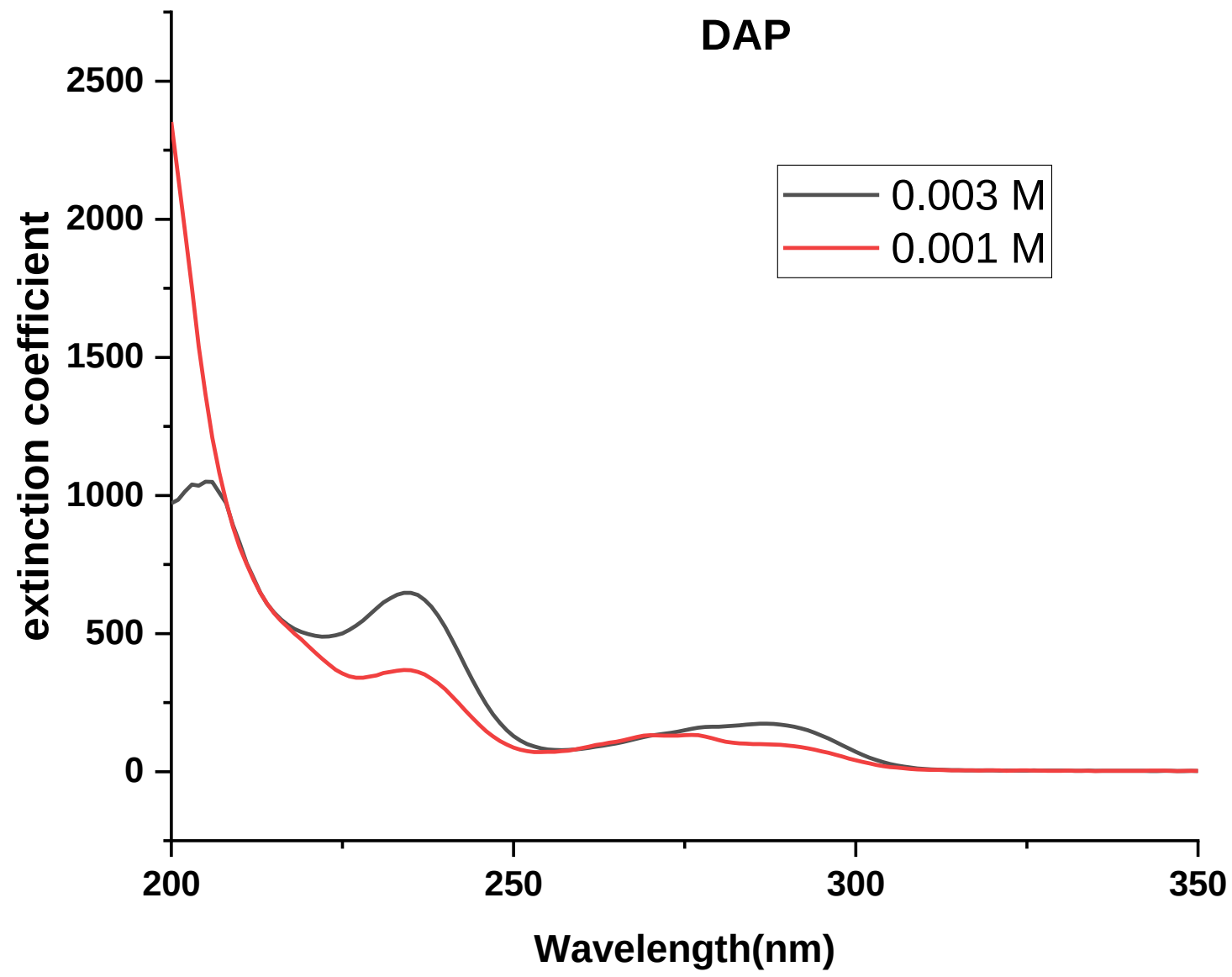

Figure S16. Extinction coefficient as a function of wavelength for DAP.

Concentration-dependent UV-Vis absorption of BDMDAP:

A series of BDMDAP solutions were prepared, with concentrations of 0.005 M, 0.01 M, 0.02 M, 0.03 M, 0.06 M, 0.11 M, 0.15 M, 0.25 M, and 0.36 M. UV-Vis spectra were recorded for these solutions (Figure S17). Starting from 0.02 M, a new absorption band emerged at 228 nm. CAC interval (228 nm): 0.01–0.02 M (Figure S18). The curve of extinction coefficient as a function of wavelength shows clear concentration-dependent spectral reshaping (Figure S19). Consistent with the wavelengths highlighted in the bar charts, an abrupt change in extinction coefficient was observed within the CAC interval.

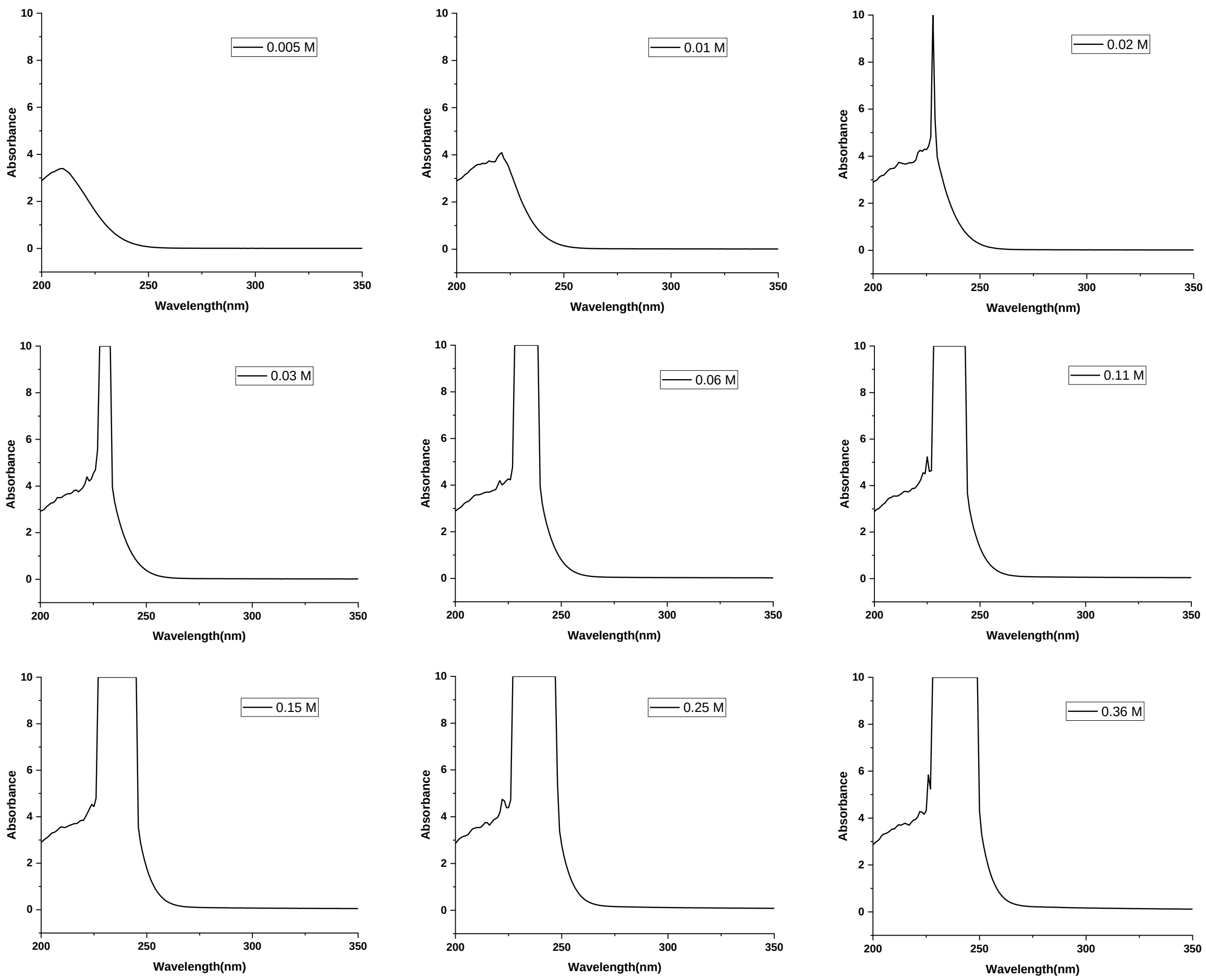

Figure S17. UV-Vis spectra of BDMDAP at different concentrations.

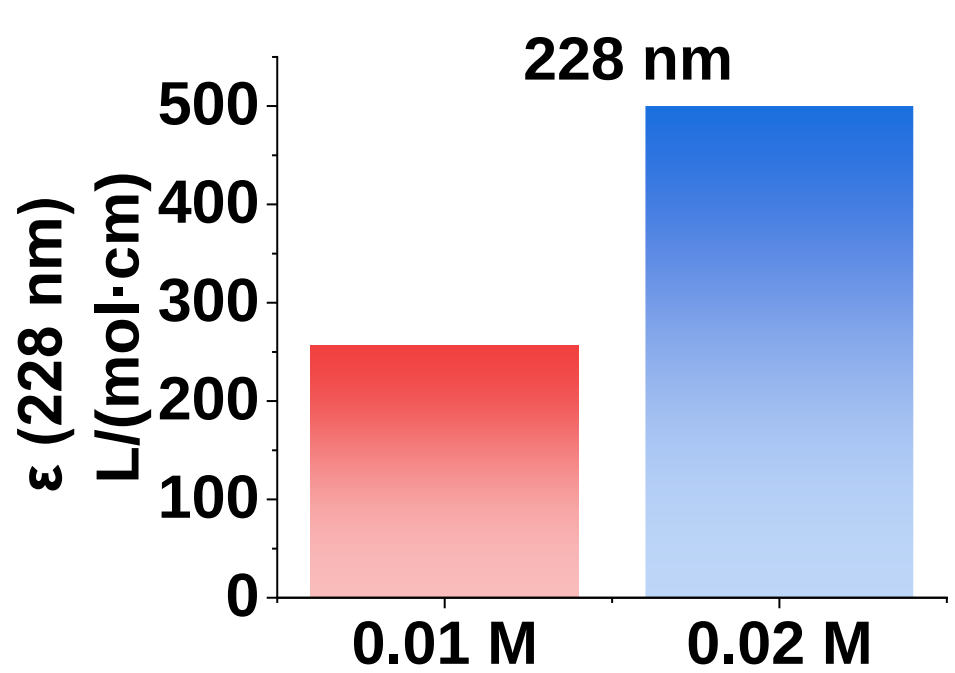

Figure S18. Determination of the AIA-related transition regions of BDMDAP based on extinction coefficient changes (at 228 nm, showing a pronounced increase from 0.01 M to 0.02 M).

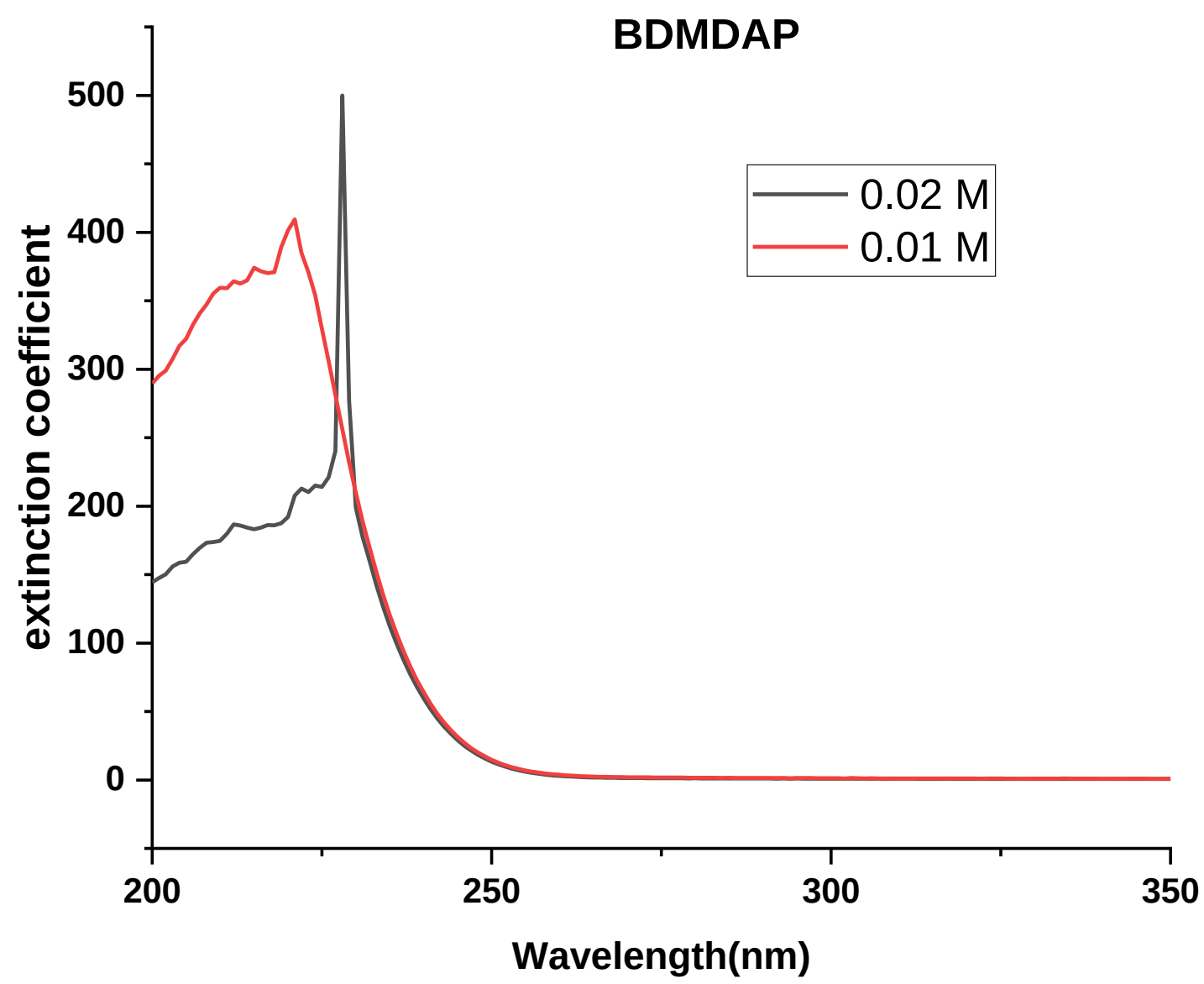

Figure S19. Extinction coefficient as a function of wavelength for BDMDAP.

Concentration-dependent UV-Vis absorption of cysteine:

A series of cysteine solutions were prepared, with concentrations of 0.005 M, 0.01 M, 0.025 M, 0.05 M, 0.1 M, 0.25 M, and 0.5 M. UV-Vis spectra were recorded for these solutions (Figure S20). Starting from 0.05 M, multiple narrow absorption bands emerged around 225 nm. CAC interval (229 nm): 0.025–0.05 M (Figure S21). The curve of extinction coefficient as a function of wavelength shows clear concentration-dependent spectral reshaping (Figure S22).

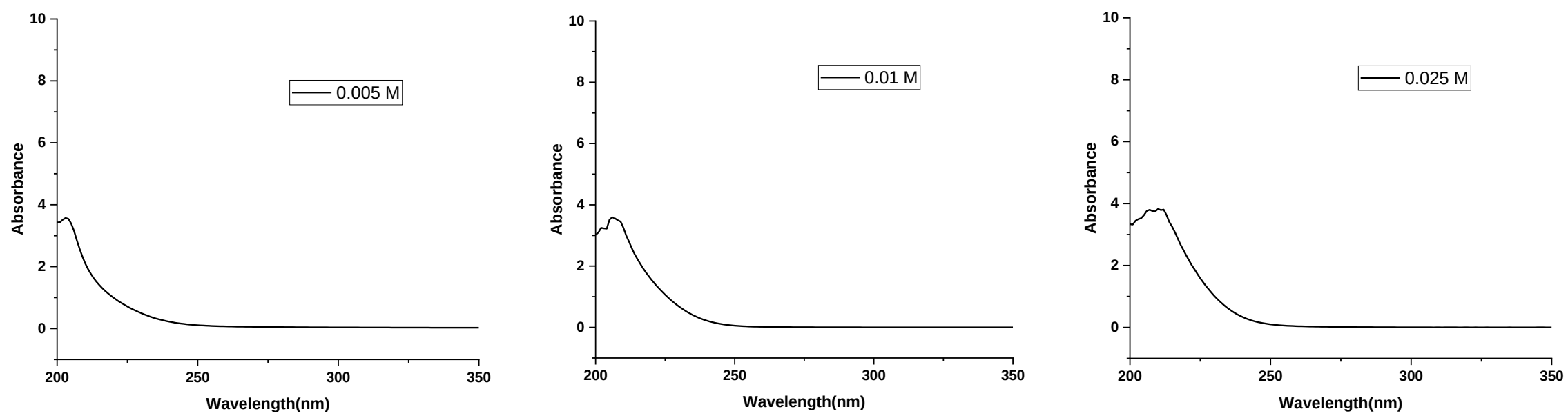

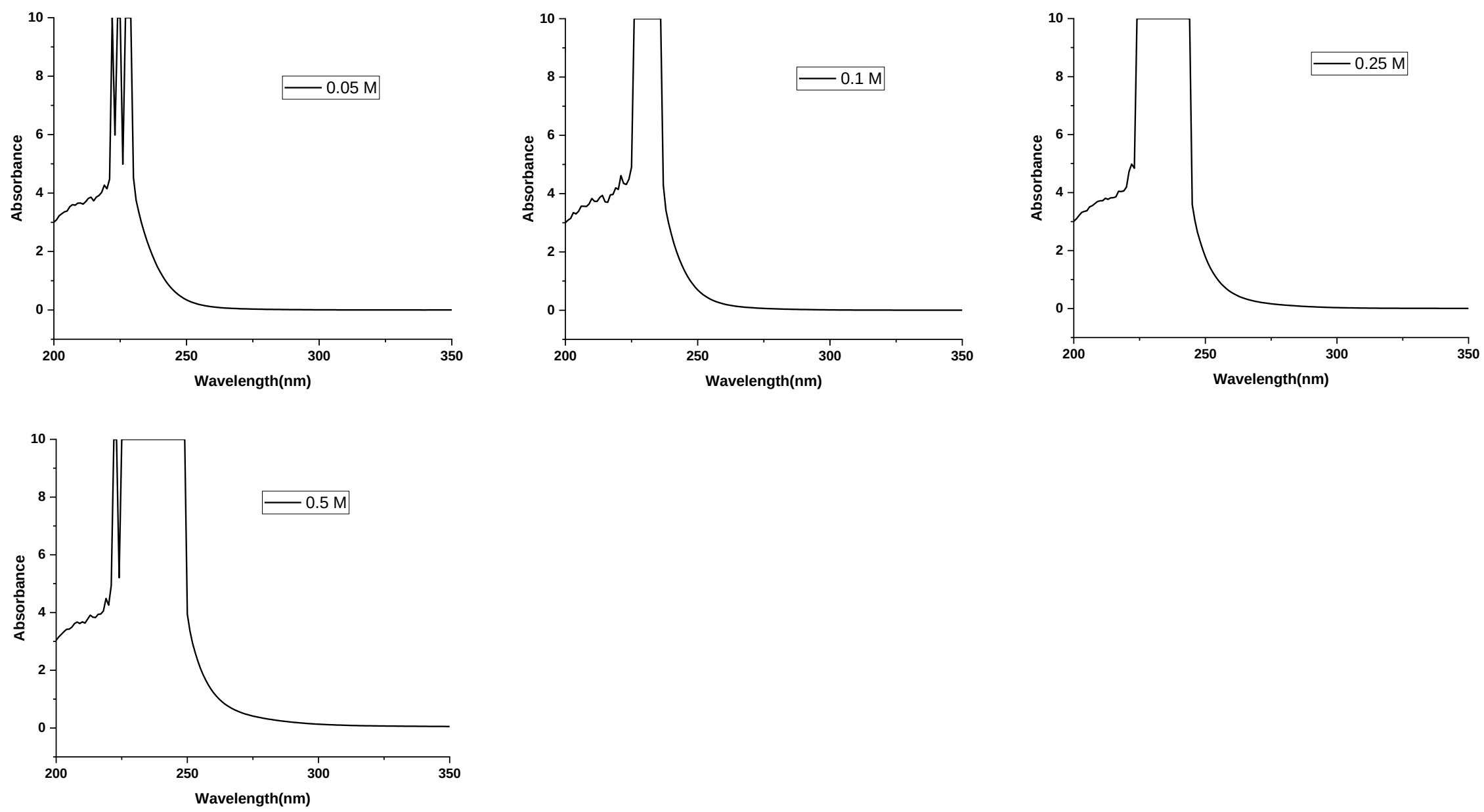

Figure S20. UV-Vis spectra of cysteine at different concentrations.

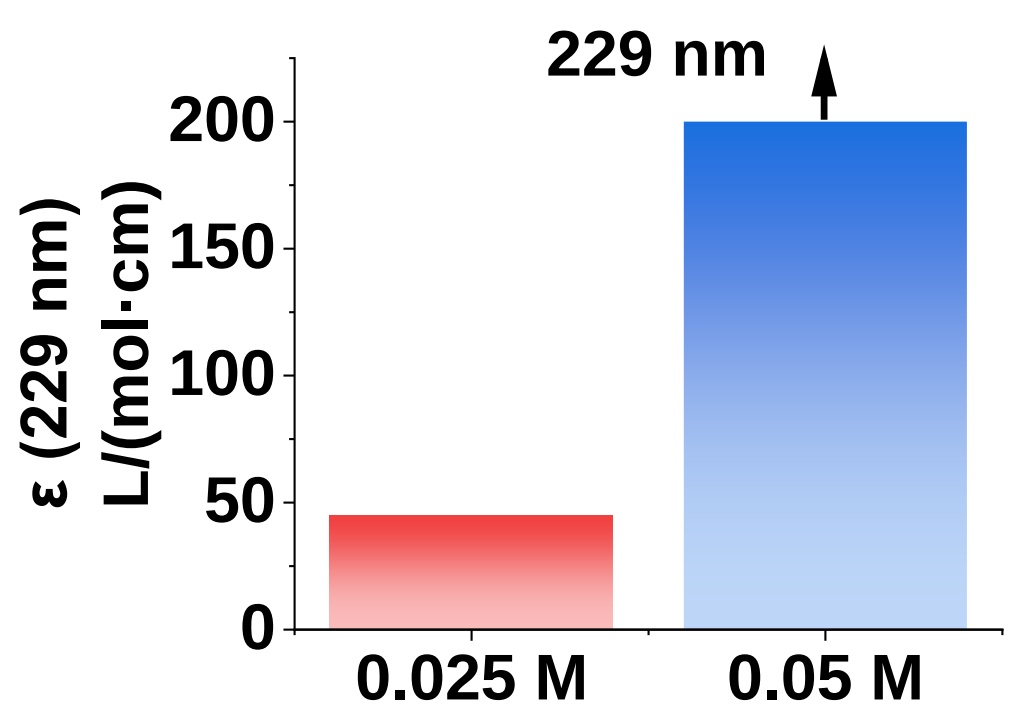

Figure S21. Determination of the AIA-related transition regions of cysteine based on extinction coefficient changes (at 229 nm, showing a pronounced increase from 0.025 M to 0.05 M).

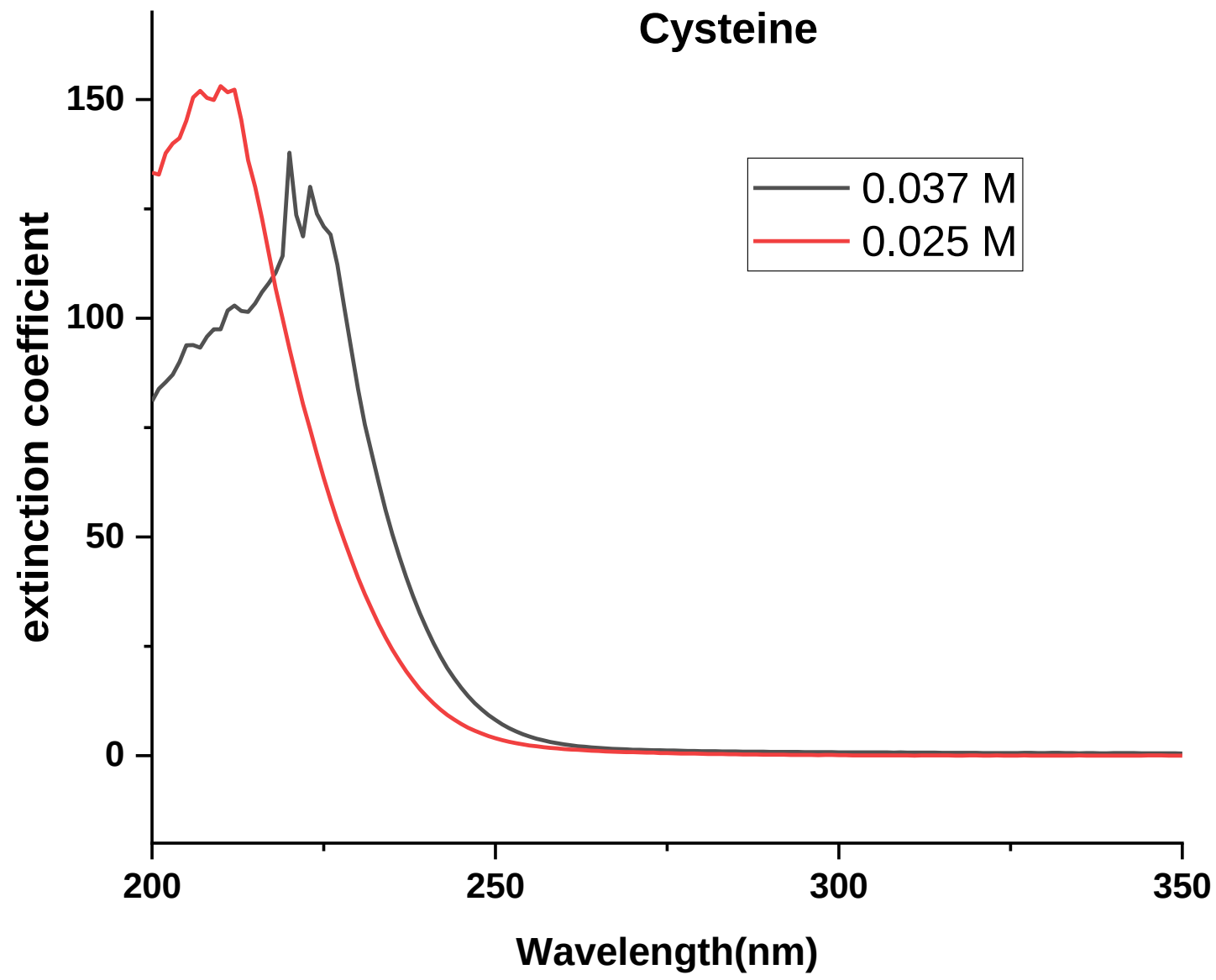

Figure S22. Extinction coefficient as a function of wavelength for cysteine.

Concentration-dependent UV-Vis absorption of lysine:

A series of lysine solutions were prepared, with concentrations of 0.01 M, 0.03 M, 0.06 M, 0.1 M, 0.12 M, 0.15 M, 0.2 M, 0.3 M, and 0.6 M. UV-Vis spectra were recorded for these solutions (Figure S23). The results demonstrated that starting from 0.06 M, multiple narrow absorption bands emerged around 225 nm. At 0.6 M, multiple narrow bands emerged at wavelengths around 350 nm. These absorption bands extended to approximately 380 nm (and further). CAC interval (228 nm): 0.03–0.06 M (Figure S24).

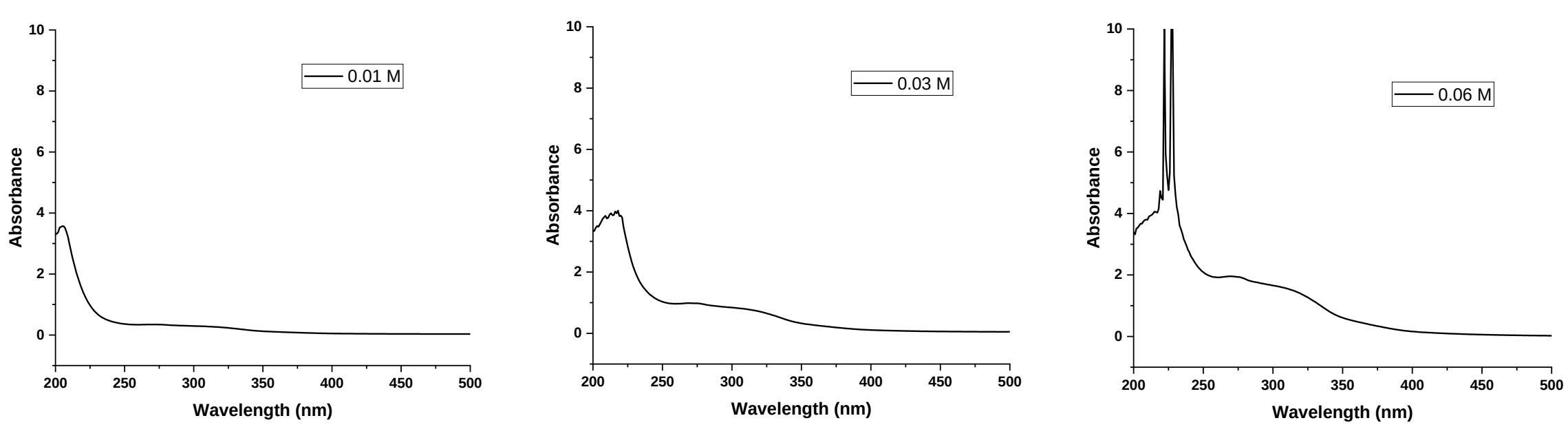

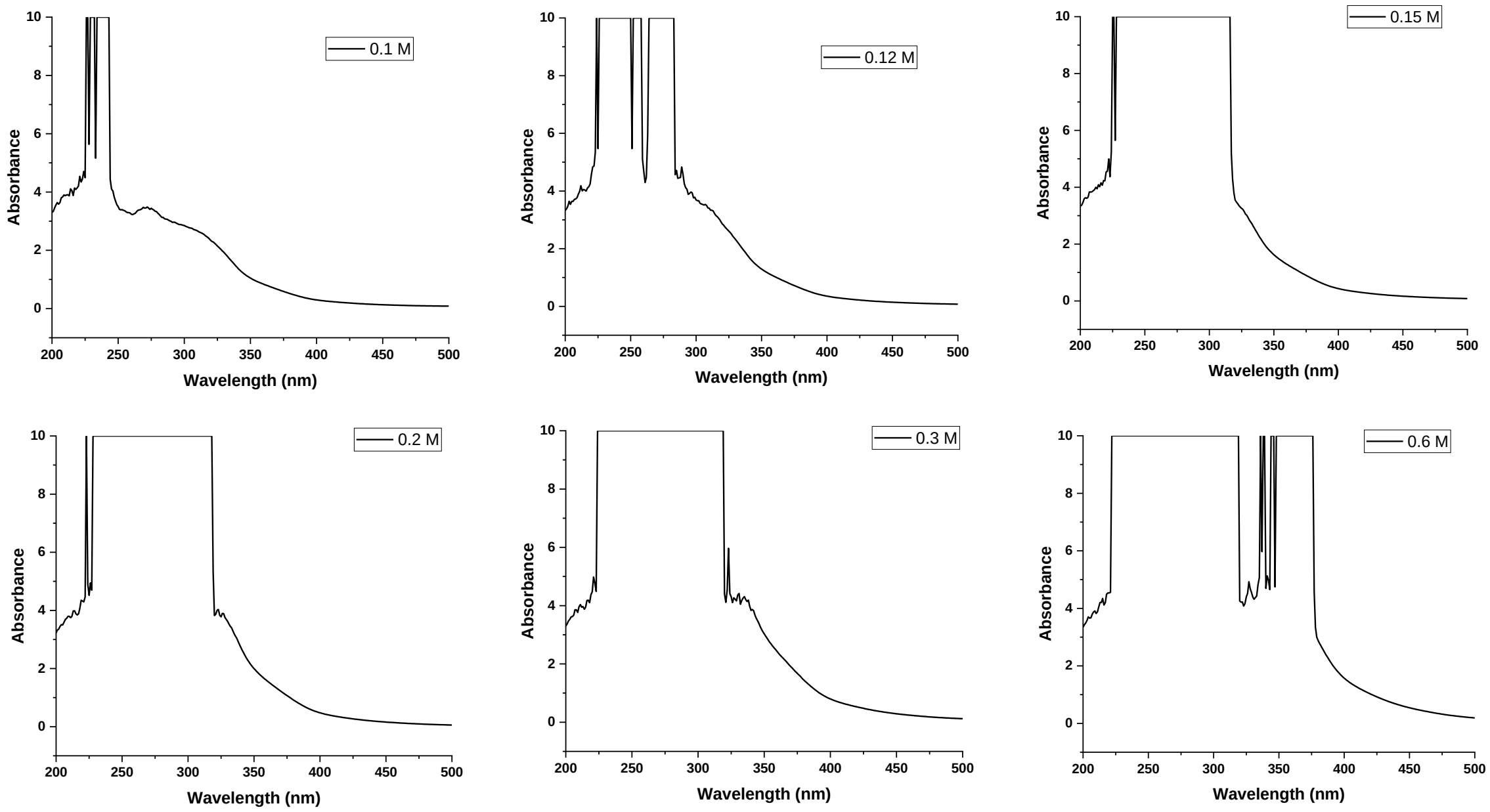

Figure S23. UV-Vis spectra of lysine at different concentrations.

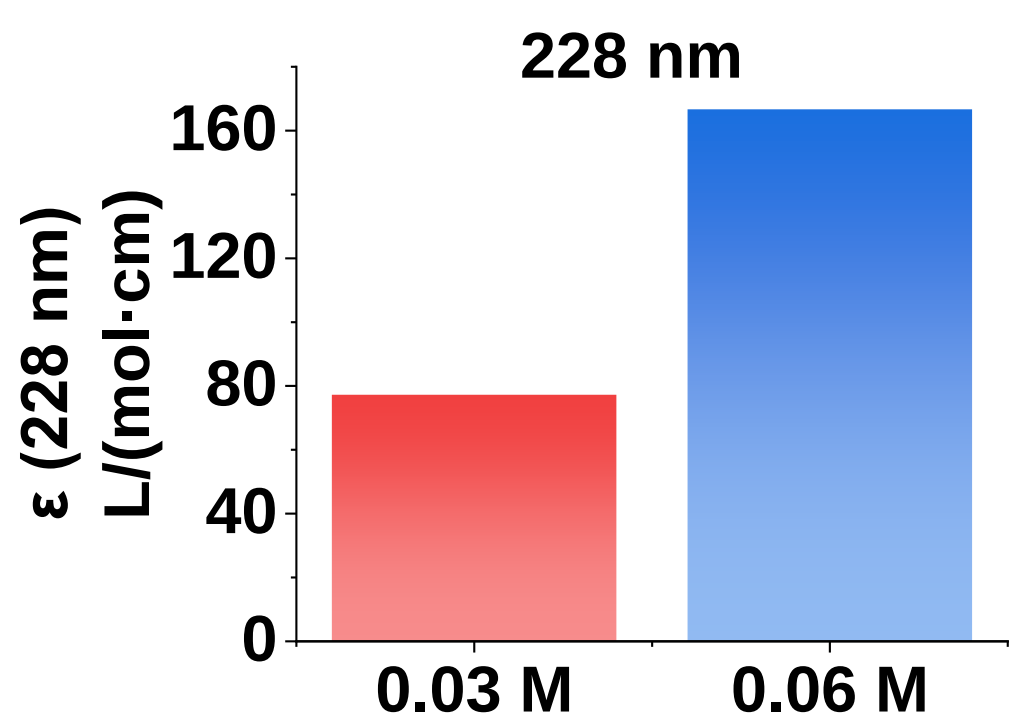

Figure S24. Determination of the AIA-related transition regions of lysine based on extinction coefficient changes (at 228 nm, showing a pronounced increase from 0.03 M to 0.06 M).

Concentration-dependent UV-Vis absorption of arginine:

A series of arginine solutions were prepared, with concentrations of 0.005 M, 0.01 M, 0.09 M, 0.1 M, 0.3 M, and 1 M. UV-Vis spectra were recorded for these solutions (Figure S25). A sharp new band emerged at 222 nm, starting from 0.1 M. Another new band emerged at around 230 nm at 0.3 M. CAC interval (222 nm): 0.09–0.1 M (Figure S26).

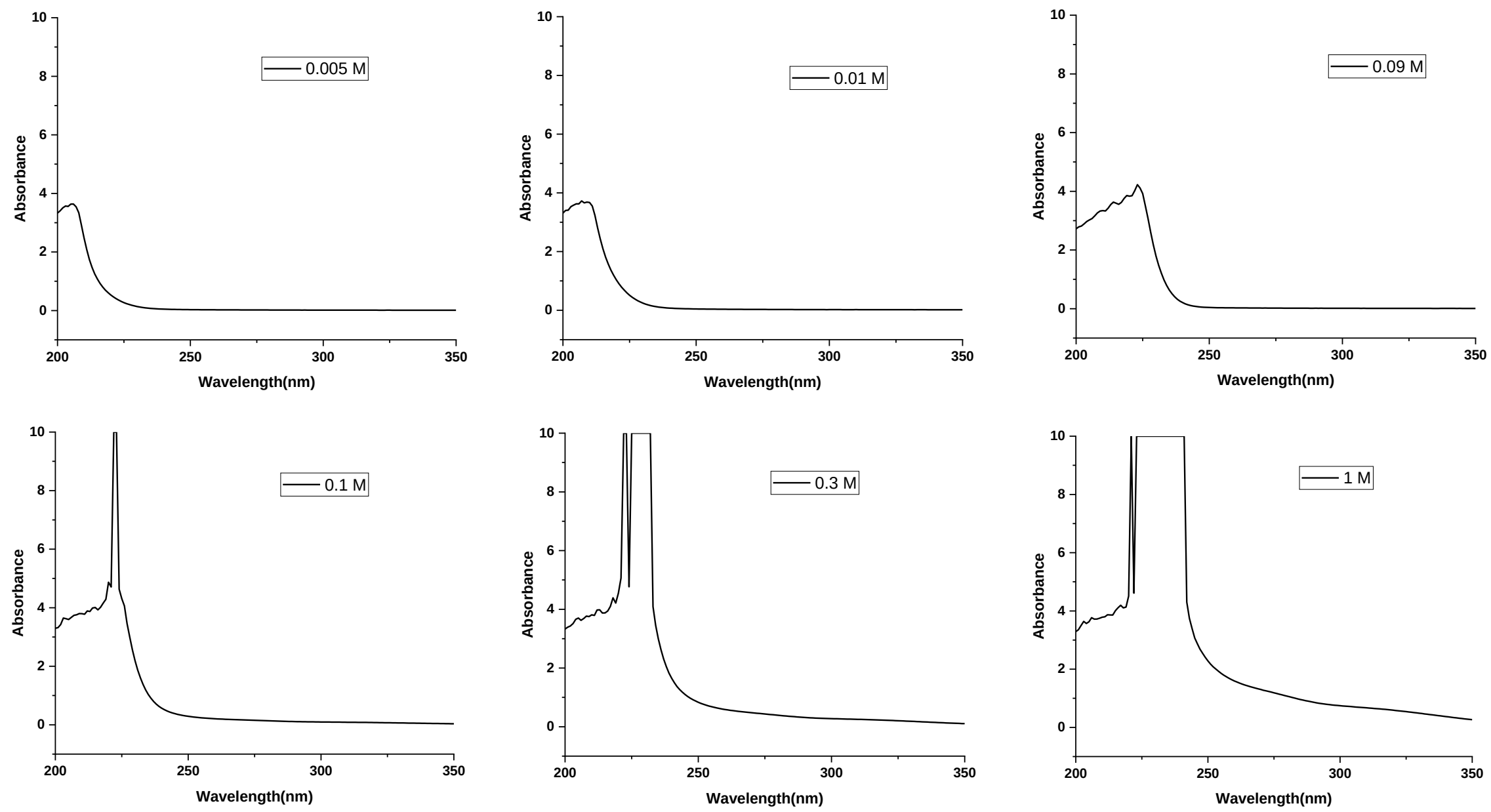

Figure S25. UV-Vis spectra of arginine at different concentrations.

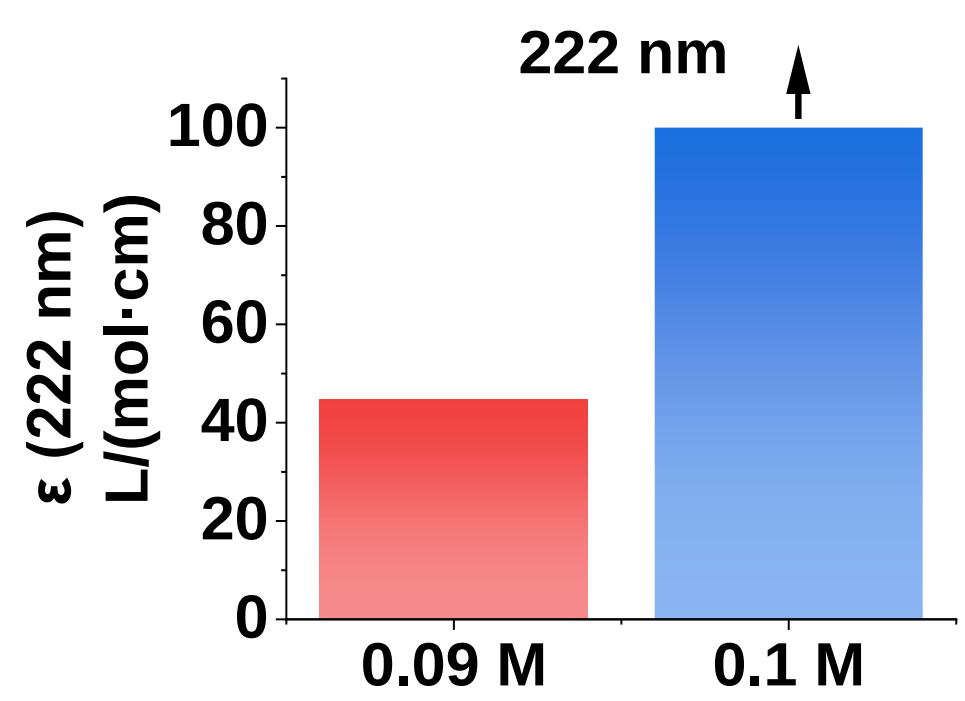

Figure S26. Determination of the AIA-related transition regions of arginine based on extinction coefficient changes (at 222 nm, showing a pronounced increase from 0.09 M to 0.1 M. The upper arrow indicates that the actual value may be higher, as the absorbance is near or above the detection limit).

Concentration-dependent UV-Vis absorption of lactic acid:

A series of lactic acid solutions were prepared, with concentrations of 0.003 M, 0.01 M, 0.03 M, 0.1 M, 0.15 M, 0.3 M, 1 M, and 3 M. UV-Vis spectra were recorded for these solutions (Figure S27). A large narrow band emerged at 230 nm starting from 0.15 M, which continued to extend toward longer wavelengths. CAC interval (230 nm): 0.1–0.15 M (Figure S28).

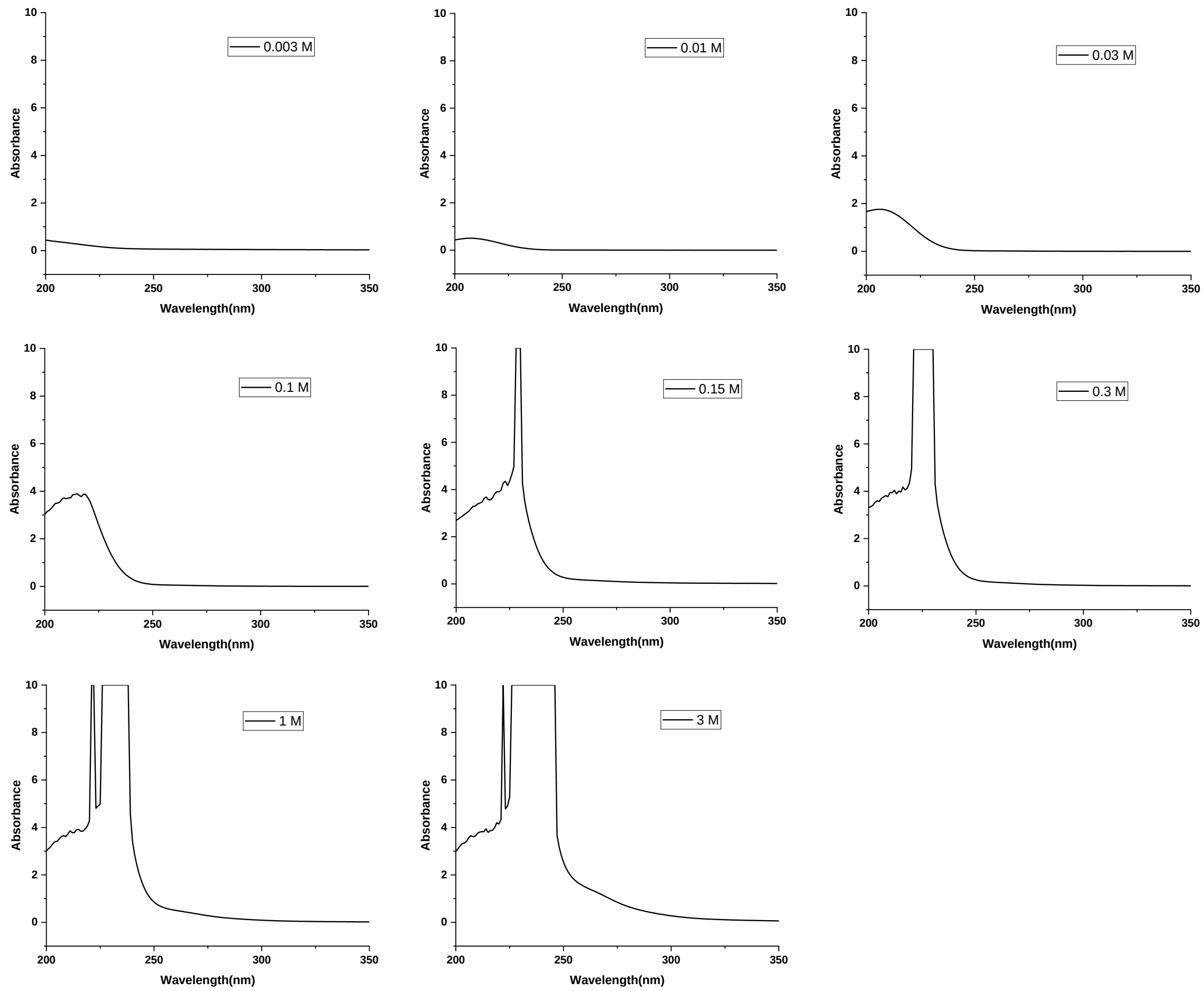

Figure S27. UV-Vis spectra of lactic acid at different concentrations.

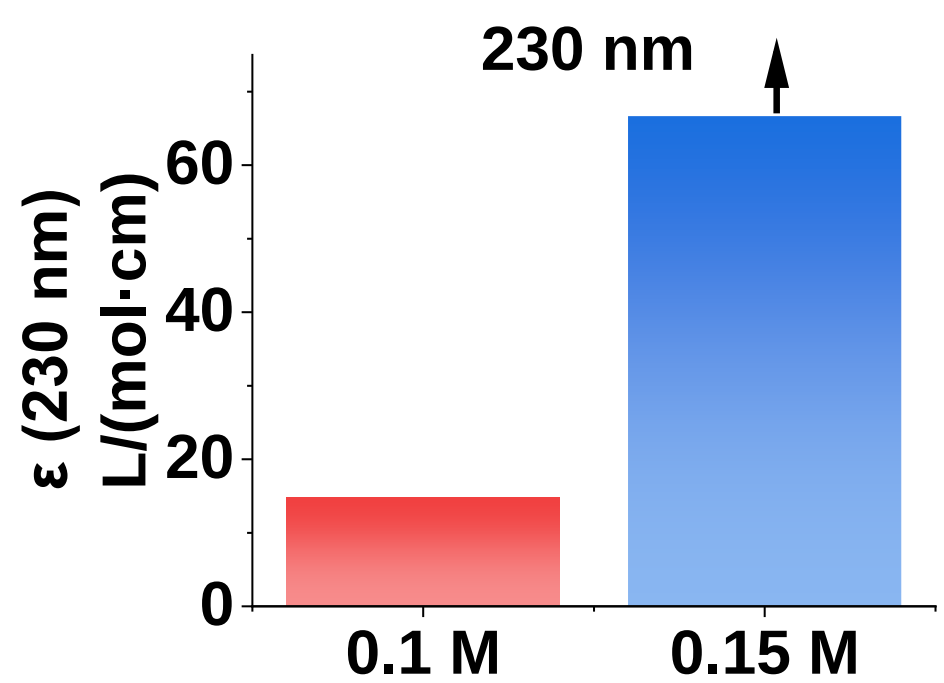

Figure S28. Determination of the AIA-related transition regions of lactic acid based on extinction coefficient changes (at 230 nm, showing a pronounced increase from 0.1 M to 0.15 M. The upper arrow indicates that the actual value may be higher, as the absorbance is near or above the detection limit).

Concentration-dependent UV-Vis absorption of citric acid:

A series of citric acid solutions were prepared, with concentrations of 0.001 M, 0.003 M, 0.01 M, 0.05 M, 0.06 M, 0.1 M, 0.3 M, 1 M, and 3 M. UV-Vis spectra were recorded for these solutions (Figure S29). A narrow band emerged at 228 nm starting from 0.06 M. CAC interval (228 nm): 0.05–0.06 M (Figure S30).

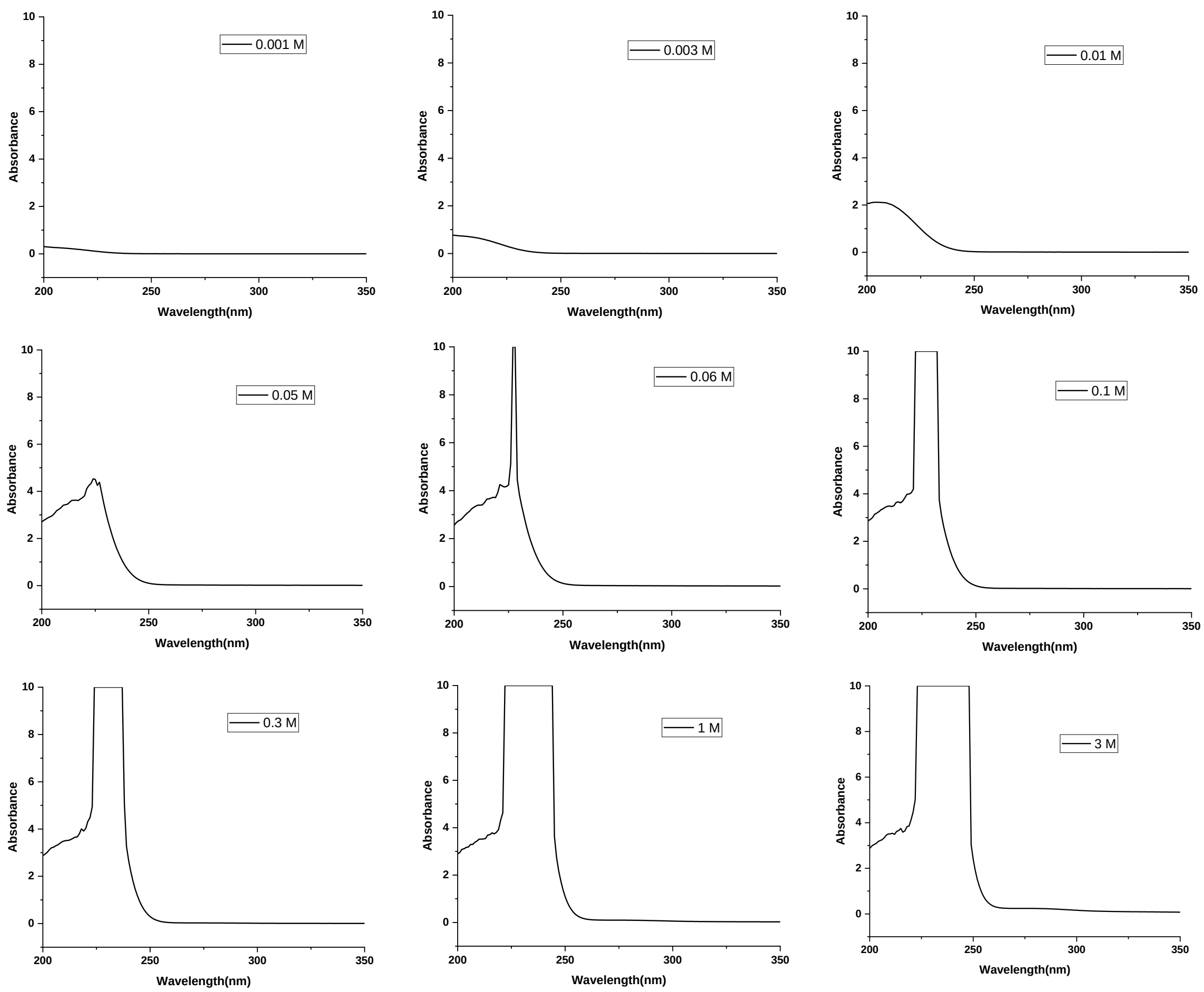

Figure S29. UV-Vis spectra of citric acid at different concentrations.

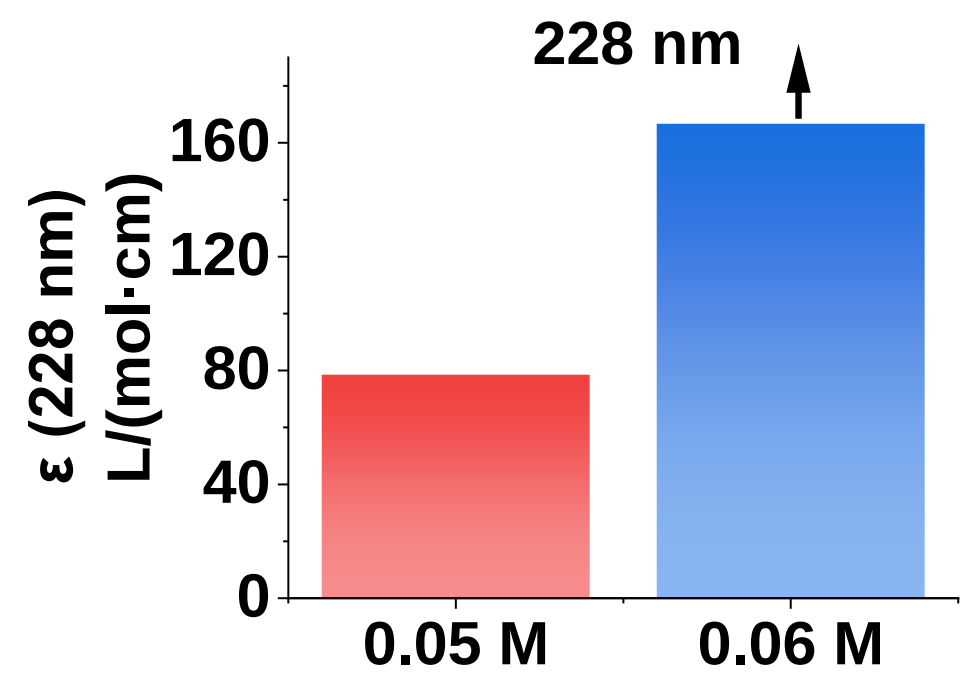

Figure S30. Determination of the AIA-related transition regions of citric acid based on extinction coefficient changes (at 228 nm, showing a pronounced increase from 0.05 M to 0.06 M. The upper arrow indicates that the actual value may be higher, as the absorbance is near or above the detection limit).

Concentration-dependent UV-Vis absorption of oxalic acid:

A series of oxalic acid solutions were prepared, with concentrations of 0.005 M, 0.01 M, 0.02 M, 0.03 M, 0.05 M, 0.06 M, 0.065 M, 0.07 M, and 0.1 M. UV-Vis spectra were recorded for these solutions (Figure S31). Multiple narrow absorption bands at around 225 nm showed up starting from 0.02 M. CAC interval (225 nm): 0.01–0.02 M (Figure S32).

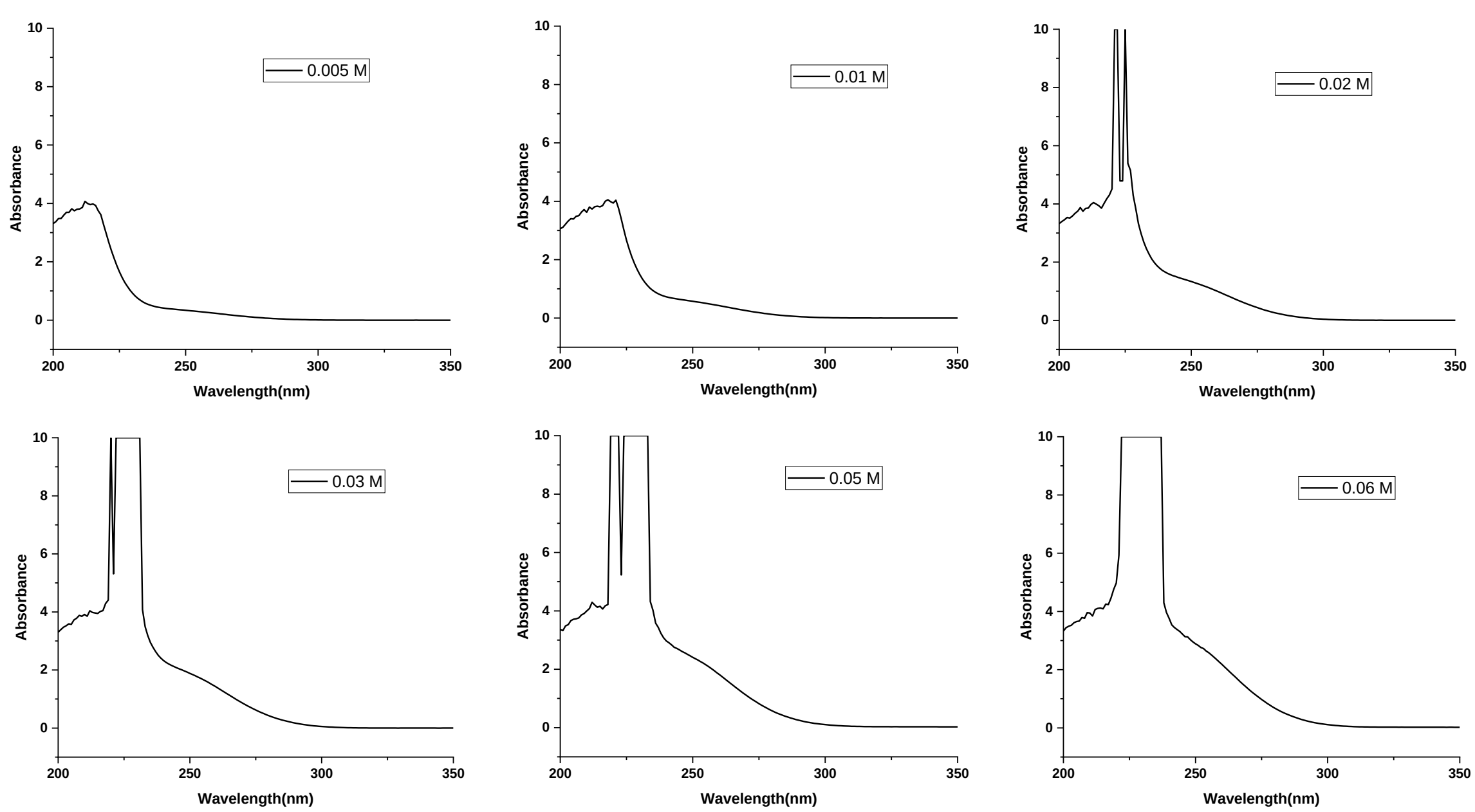

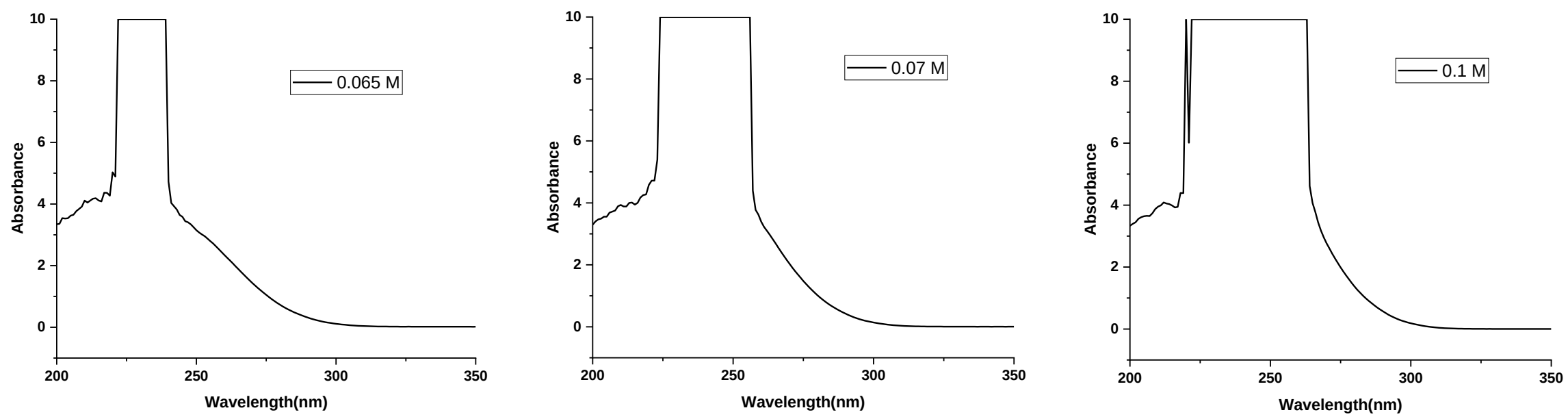

Figure S31. UV-Vis spectra of oxalic acid at different concentrations.

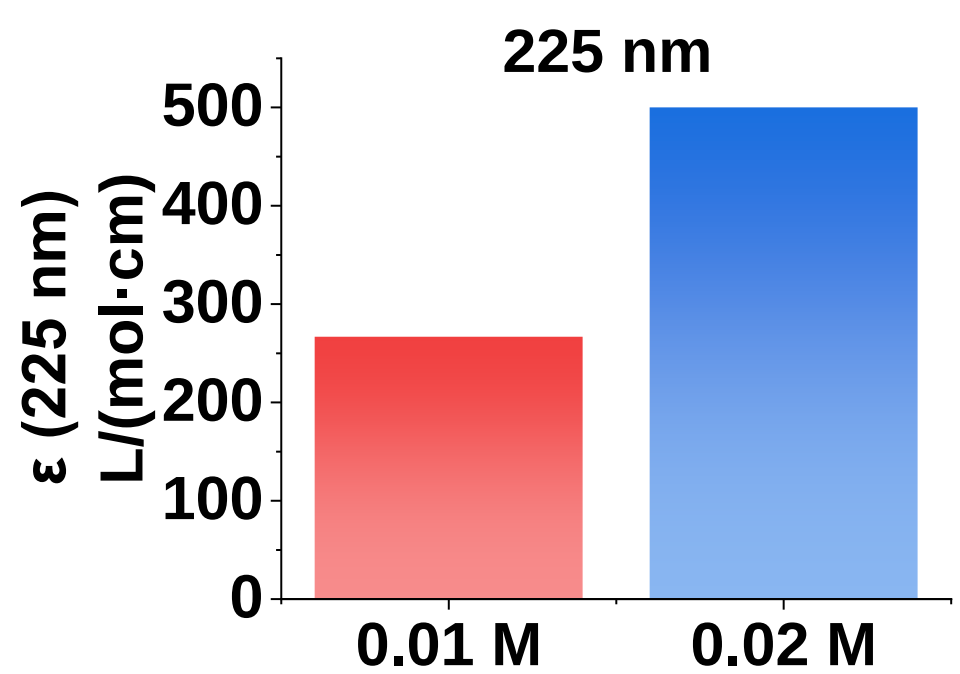

Figure S32. Determination of the AIA-related transition regions of oxalic acid based on extinction coefficient changes (at 225 nm, showing a pronounced increase from 0.01 M to 0.02 M).

Concentration-dependent UV-Vis absorption of histidine:

A series of histidine solutions were prepared, with concentrations of 0.001 M, 0.003 M, 0.005 M, 0.006 M, 0.007 M, 0.01 M, and 0.1 M, 0.27 M (saturated). Due to the limited solubility of histidine, the tested concentration could not exceed 0.27 M. UV-Vis spectra were recorded for these solutions (Figure S33). A sharp band emerged at 227 nm starting from 0.007 M. CAC interval (227 nm): 0.006–0.007 M (Figure S34).

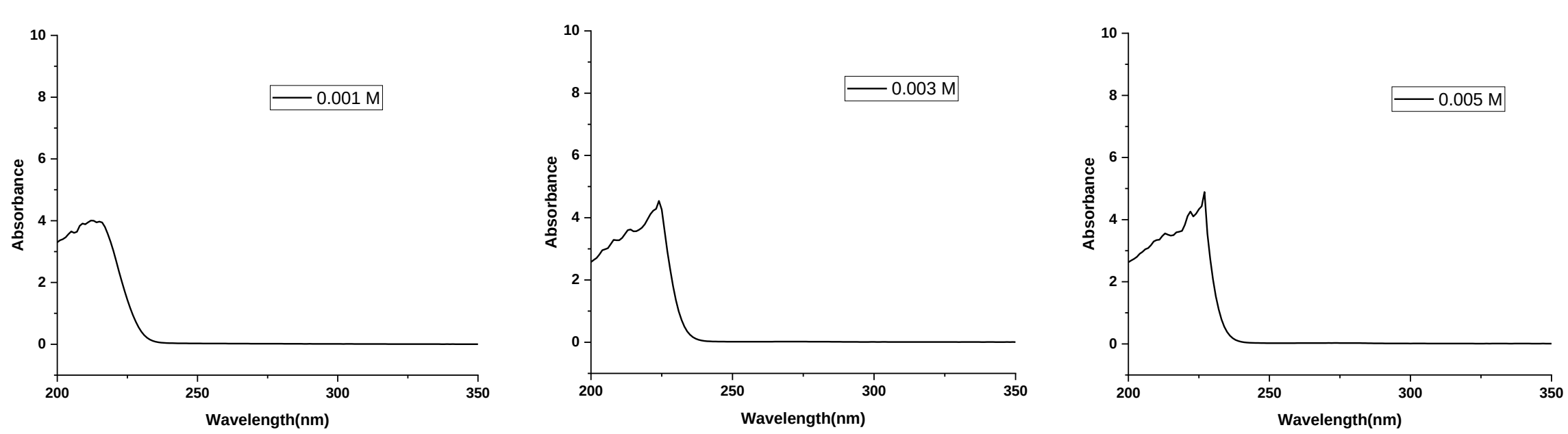

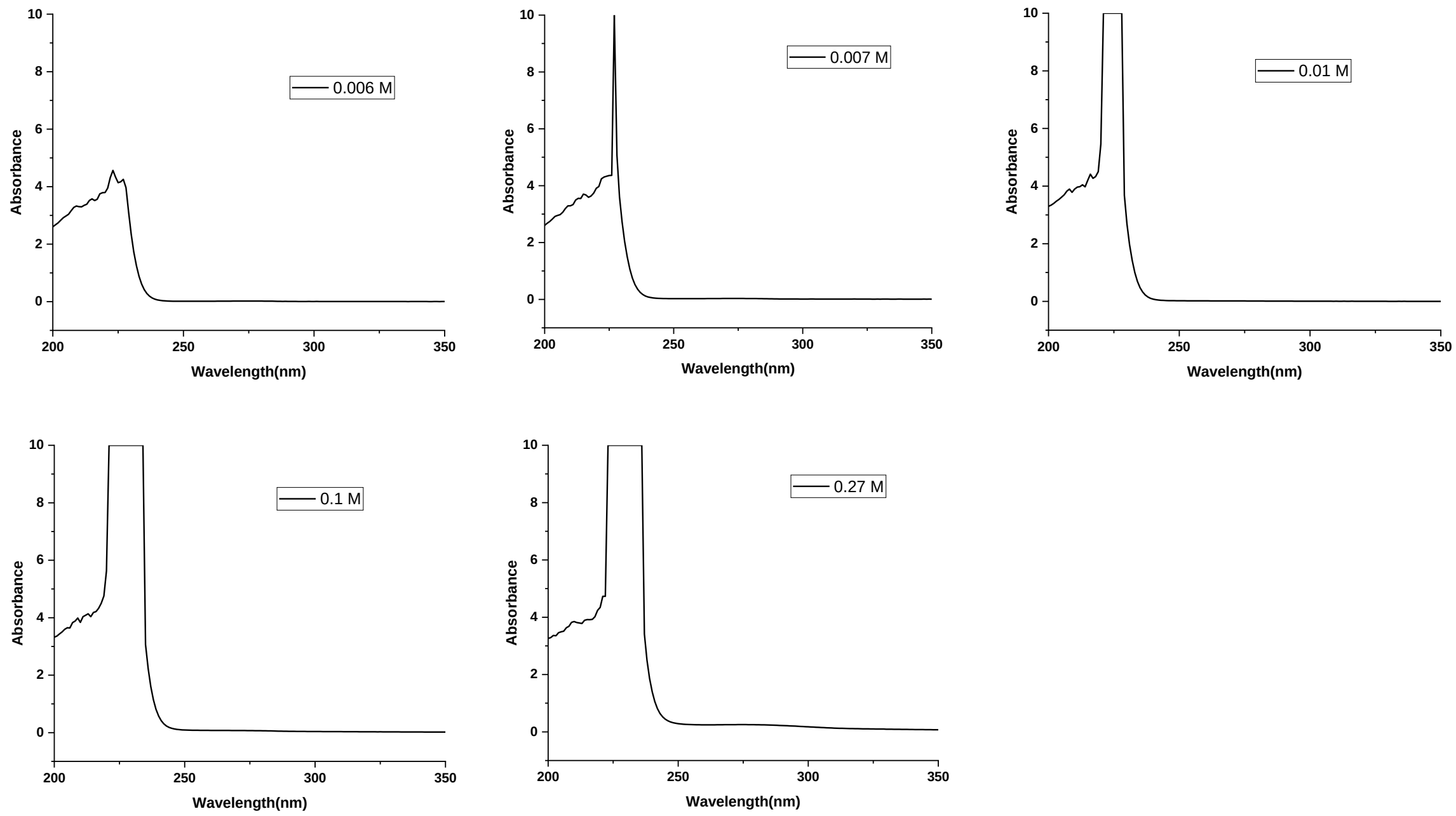

Figure S33. UV-Vis spectra of histidine at different concentrations.

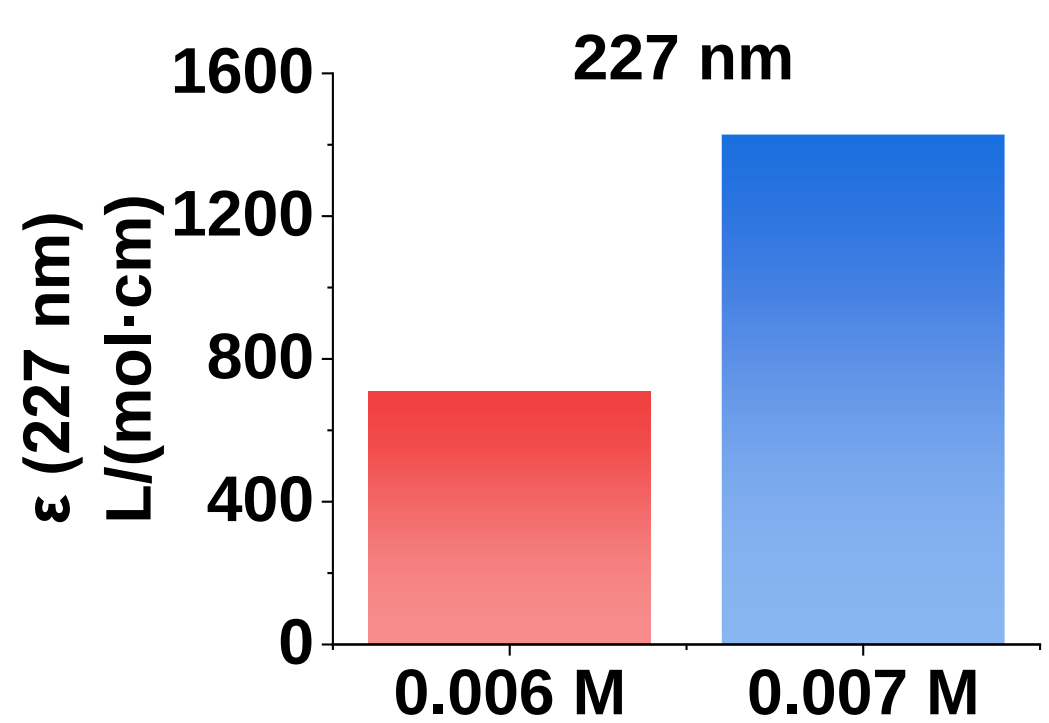

Figure S34. Determination of the AIA-related transition regions of histidine based on extinction coefficient changes (at 227 nm, showing a pronounced increase from 0.006 M to 0.007 M).

Concentration-dependent UV-Vis absorption of alanine:

A series of alanine solutions were prepared, with concentrations of 0.01 M, 0.1 M, 0.69 M, 0.7 M and 1 M. UV-Vis spectra were recorded for these solutions (Figure S35). A sharp band emerged at 222 nm at 0.7 M. Another sharp band emerged at around 225 nm starting from 1.0 M. CAC interval (222 nm): 0.69–0.7 M (Figure S36).

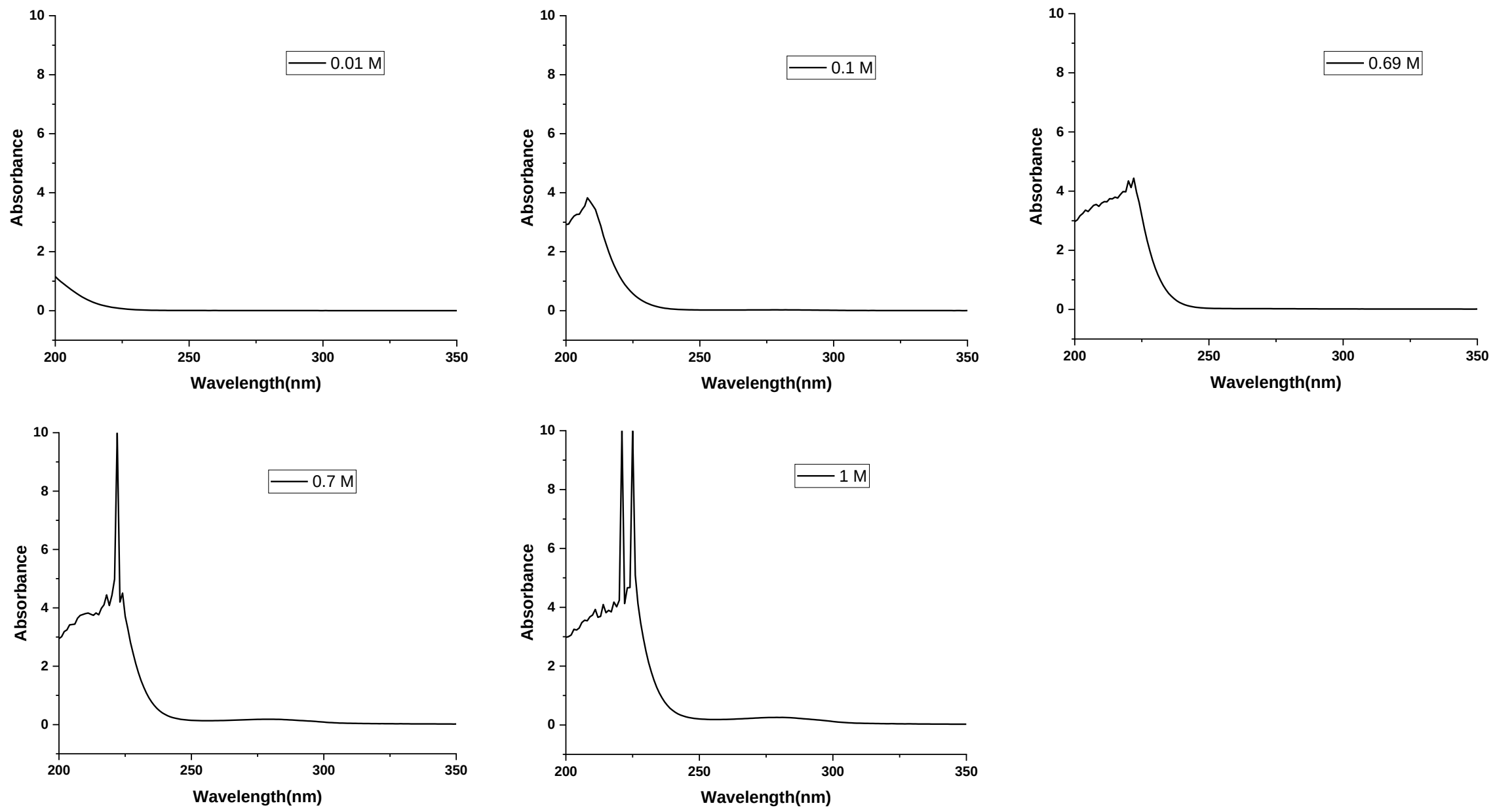

Figure S35. UV-Vis spectra of alanine at different concentrations.

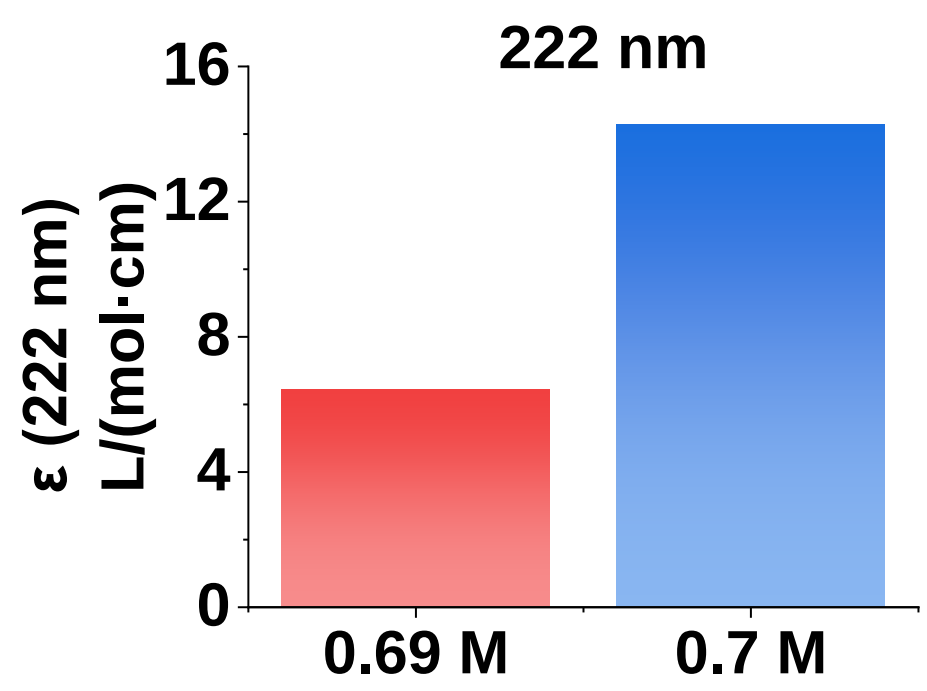

Figure S36. Determination of the AIA-related transition regions of alanine based on extinction coefficient changes (at 222 nm, showing a pronounced increase from 0.69 M to 0.7 M).

Concentration-dependent UV-Vis absorption of glutamic acid:

A series of glutamic acid solutions were prepared, with concentrations of 0.005 M, 0.015 M, 0.02 M, 0.03 M, 0.04 M and 0.05 M. Due to the limited solubility of glutamic acid, the tested concentration could not exceed 0.05 M. UV-Vis spectra were recorded for these solutions (Figure S37). Within the tested concentration range, no obvious AIA effect was observed for glutamic acid.

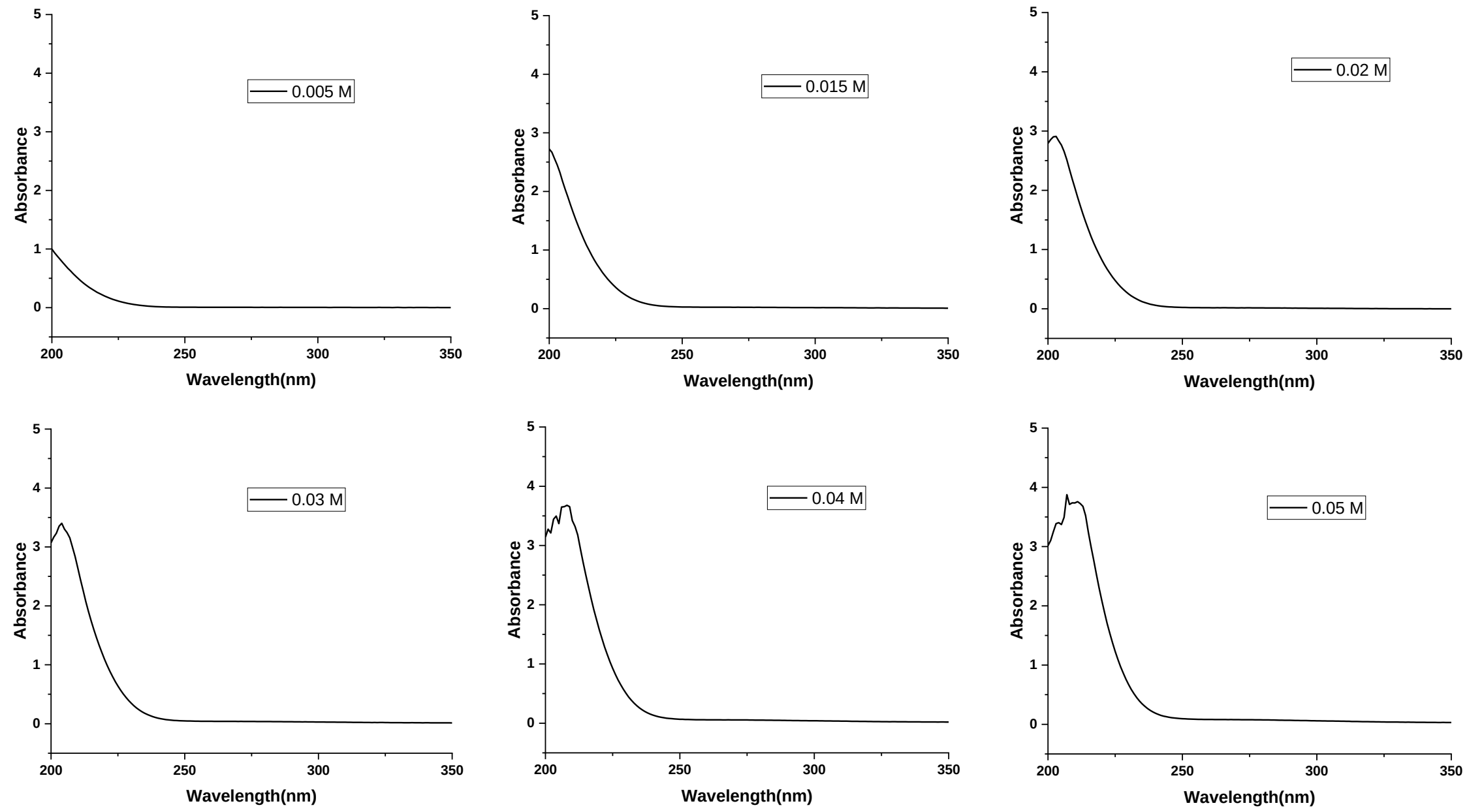

Figure S37. UV-Vis spectra of glutamic acid at different concentrations.

Concentration-dependent UV-Vis absorption of serine:

A series of serine solutions were prepared, with concentrations of 0.01 M, 0.1 M, 0.69 M, 0.7 M and 1 M. UV-Vis spectra were recorded for these solutions (Figure S38). A sharp band emerged at around 223 nm at 0.69 M. Another two sharp bands emerged at around 230 nm starting from 1.0 M. CAC interval (223 nm): 0.69–0.7 M (Figure S39).

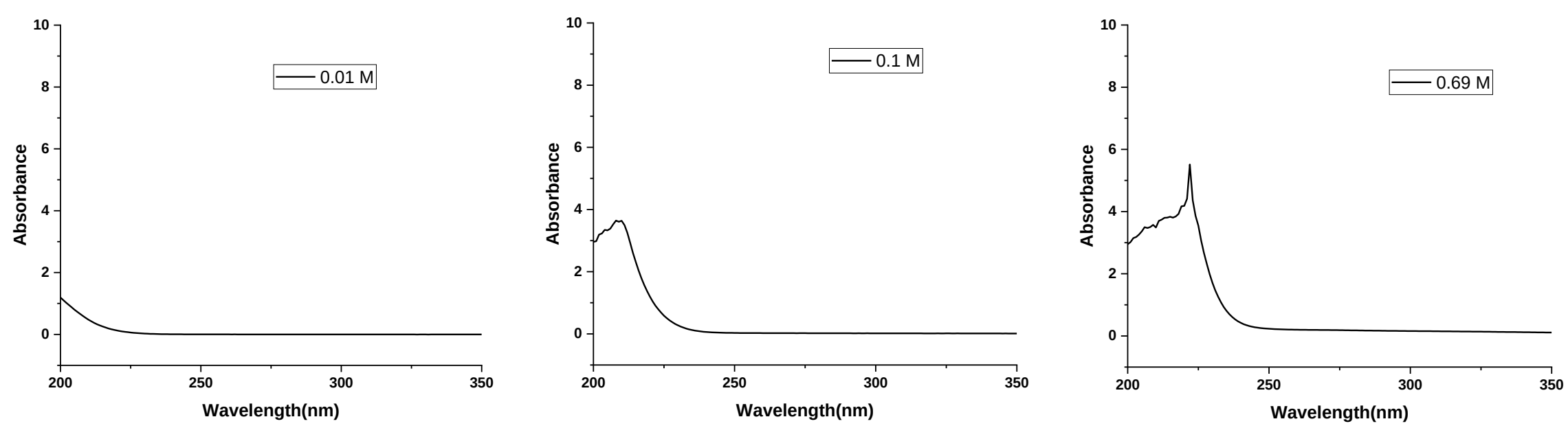

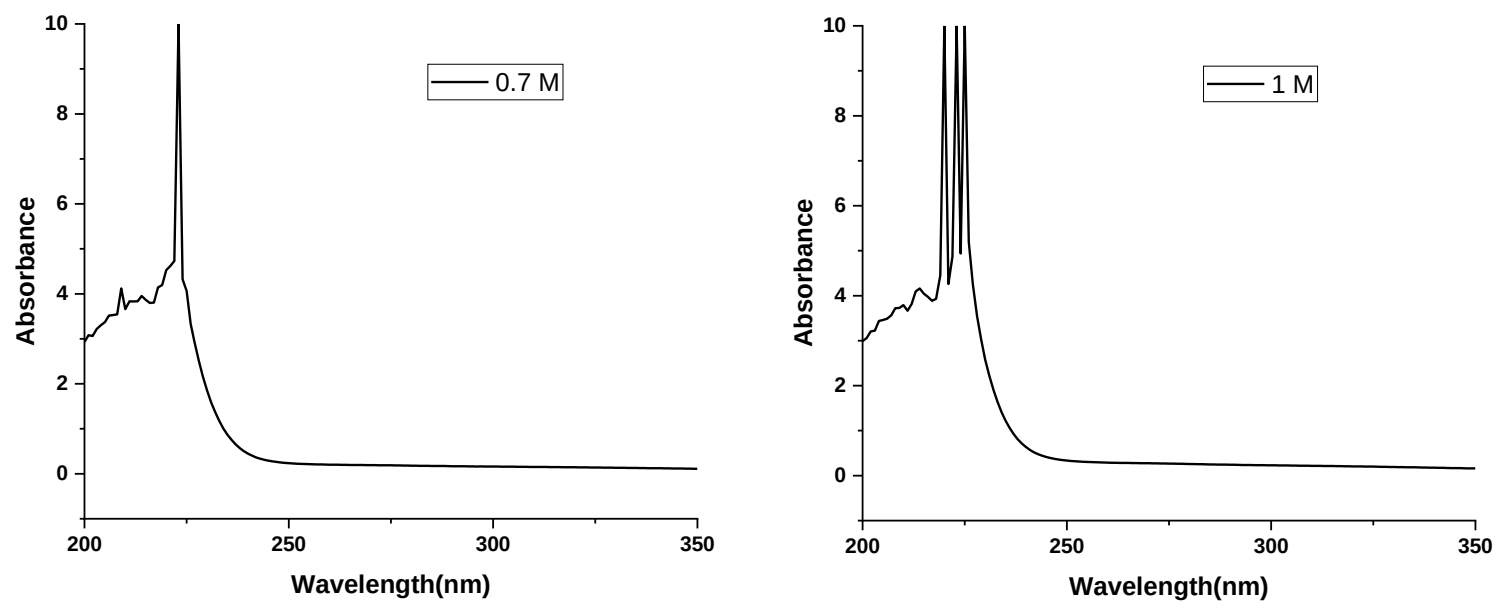

Figure S38. UV-Vis spectra of serine at different concentrations.

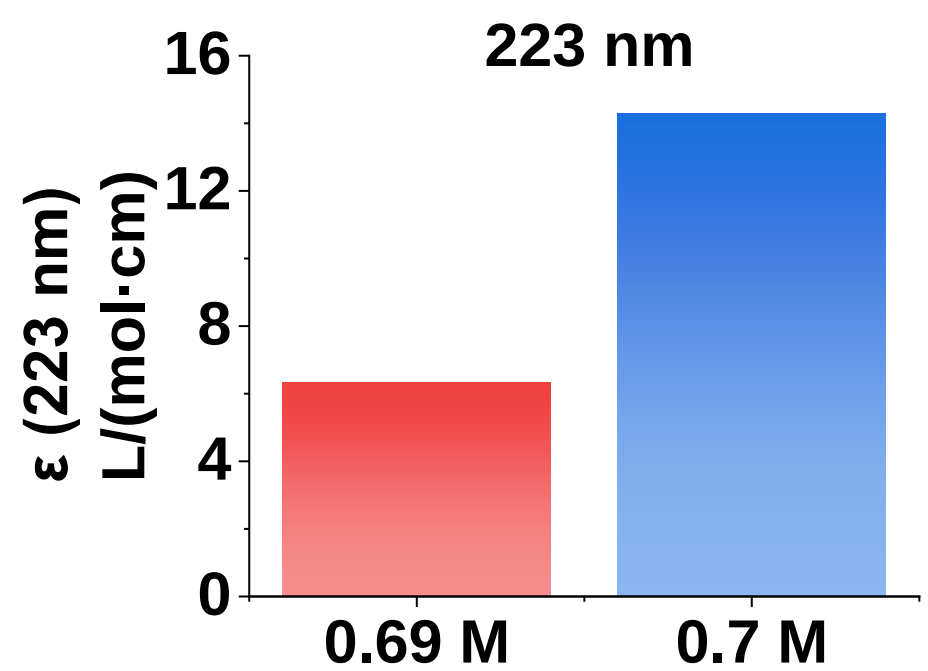

Figure S39. Determination of the AIA-related transition regions of serine based on extinction coefficient changes (at 223 nm, showing a pronounced increase from 0.69 M to 0.7 M).

Concentration-dependent UV-Vis absorption of glycine:

A series of glycine solutions were prepared, with concentrations of 0.001 M, 0.03 M, 0.05 M, 0.1 M, 0.9 M and 1 M. UV-Vis spectra were recorded for these solutions (Figure S40). Within the tested concentration range, no obvious AIA effect was observed for glycine.

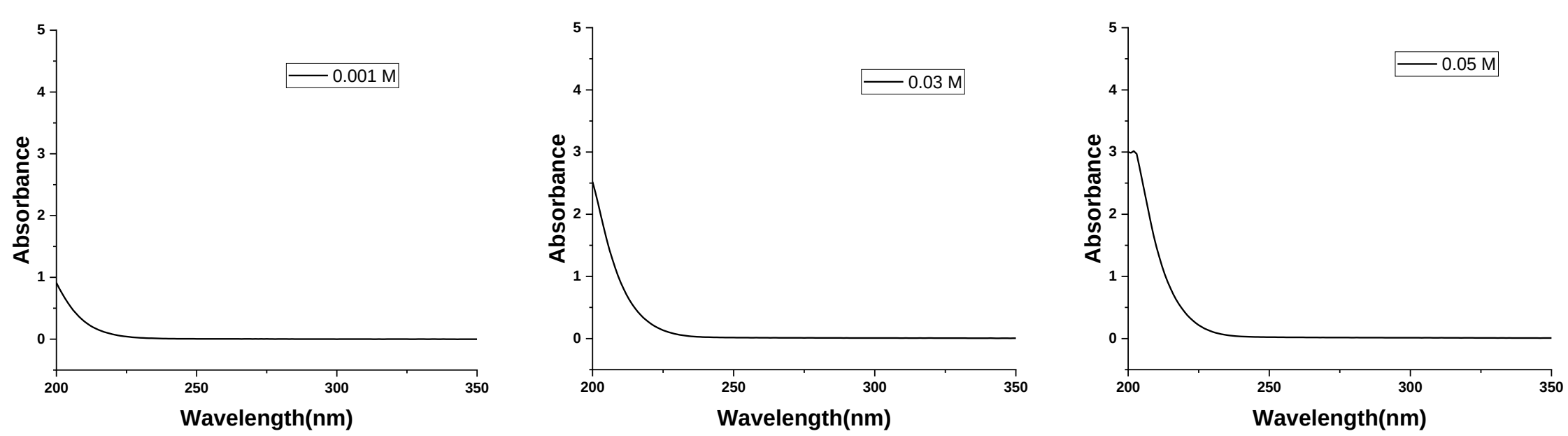

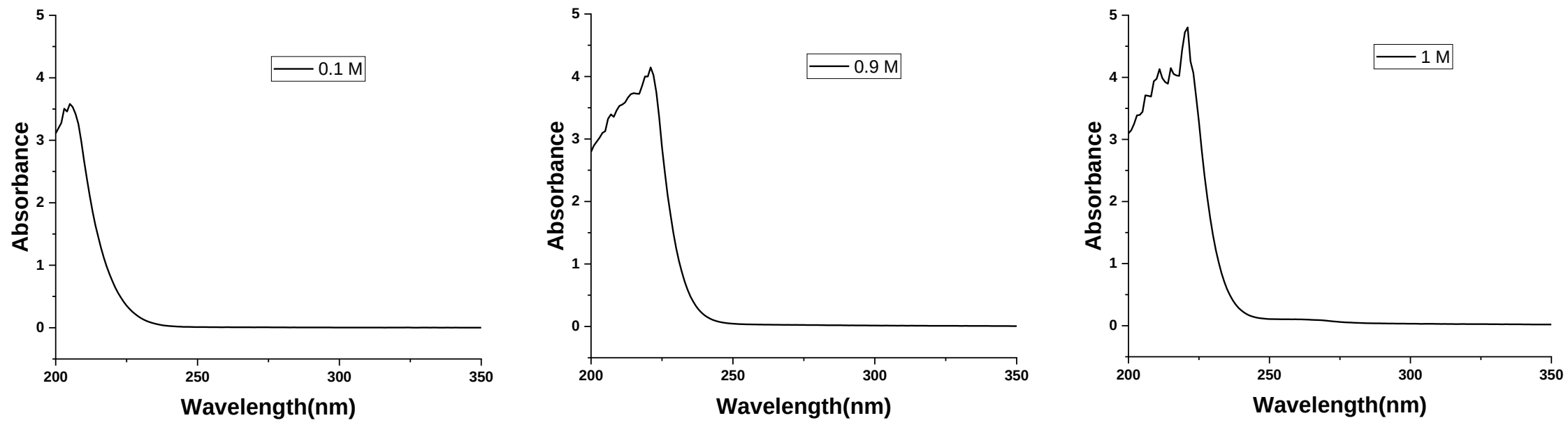

Figure S40. UV-Vis spectra of glycine at different concentrations.

Concentration-dependent UV-Vis absorption of glycolaldehyde:

A series of glycolaldehyde solutions were prepared, with concentrations of 0.01 M, 0.03 M, 0.1 M, 0.3 M, 1 M, and 3 M. UV-Vis spectra were recorded for these solutions (Figure S41). Across 200–350 nm, the absorbance was negligible at low concentrations. The redshift of absorption bands was quite trivial across the entire concentration range.

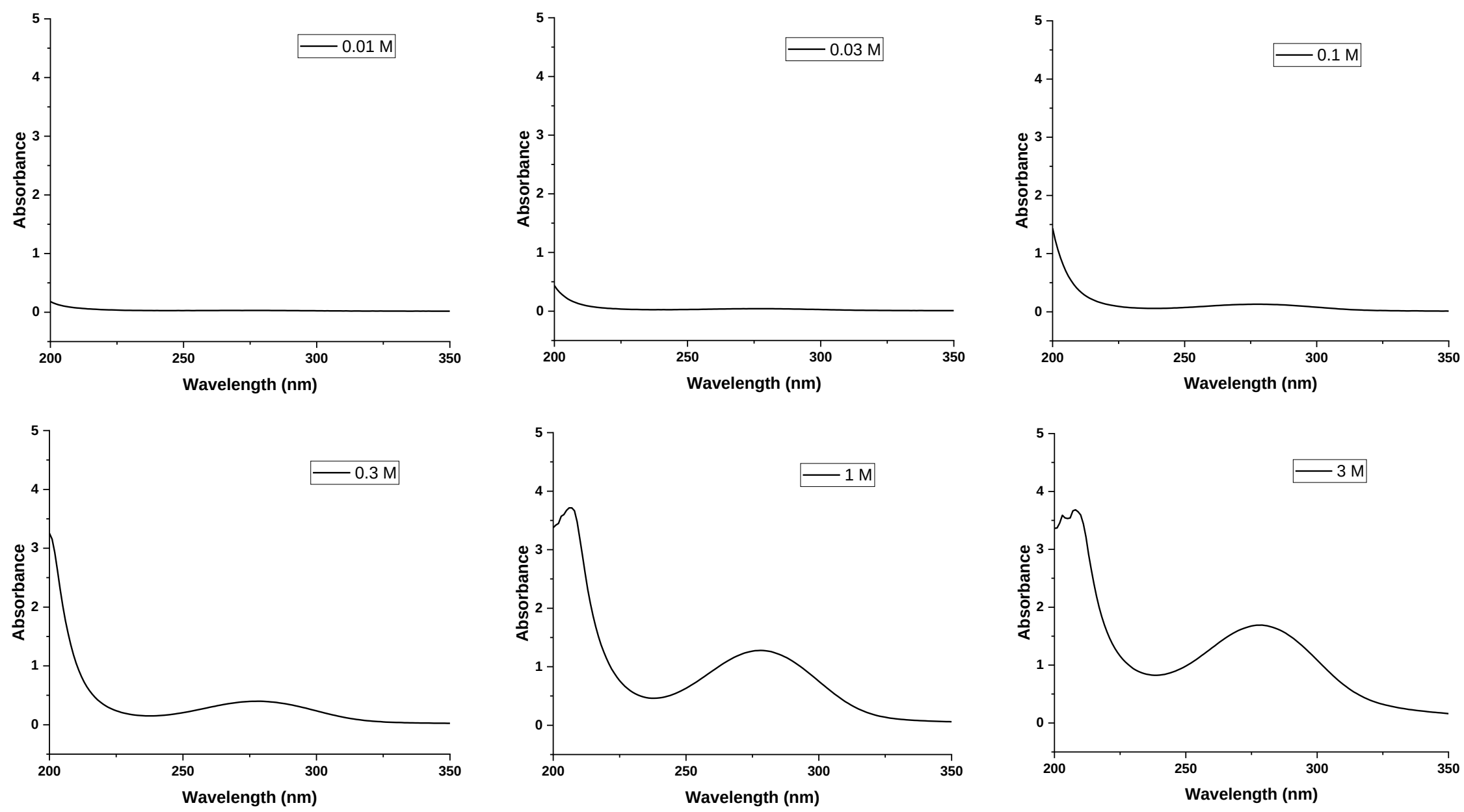

Figure S41. UV-Vis spectra of glycolaldehyde at different concentrations.

Concentration-dependent UV-Vis absorption of glyceraldehyde:

A series of glyceraldehyde solutions were prepared, with concentrations of 0.001 M, 0.01 M, 0.05 M, 0.09 M and 0.1 M. Due to the limited solubility of glyceraldehyde, the tested concentration could not exceed 0.1 M. UV-Vis spectra were recorded for these solutions (Figure S42). Across

200–350 nm, the absorbance was negligible at low concentrations. The redshift of absorption bands was quite trivial across the entire concentration range.

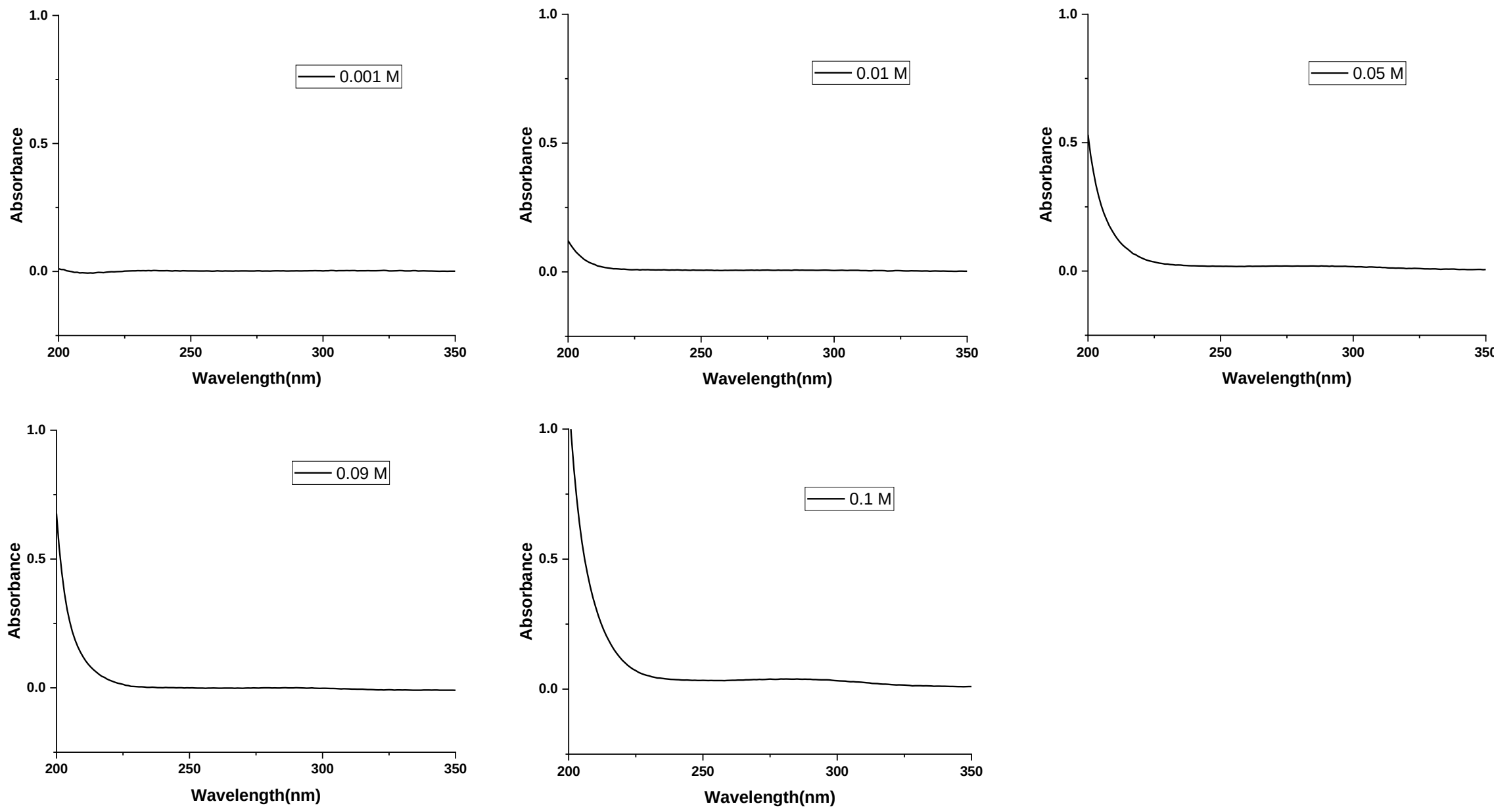

Figure S42. UV-Vis spectra of glyceraldehyde at different concentrations.

Concentration-dependent UV-Vis absorption of $NH_4H_2PO_4$:

A series of $NH_4H_2PO_4$ solutions were prepared, with concentrations of 0.01 M, 0.03 M, 0.06 M, 0.11 M, 0.36 M, 1 M, and 2 M. UV-Vis spectra were recorded for these solutions (Figure S43). Across 200–350 nm, the absorbance was small throughout the entire concentration range.

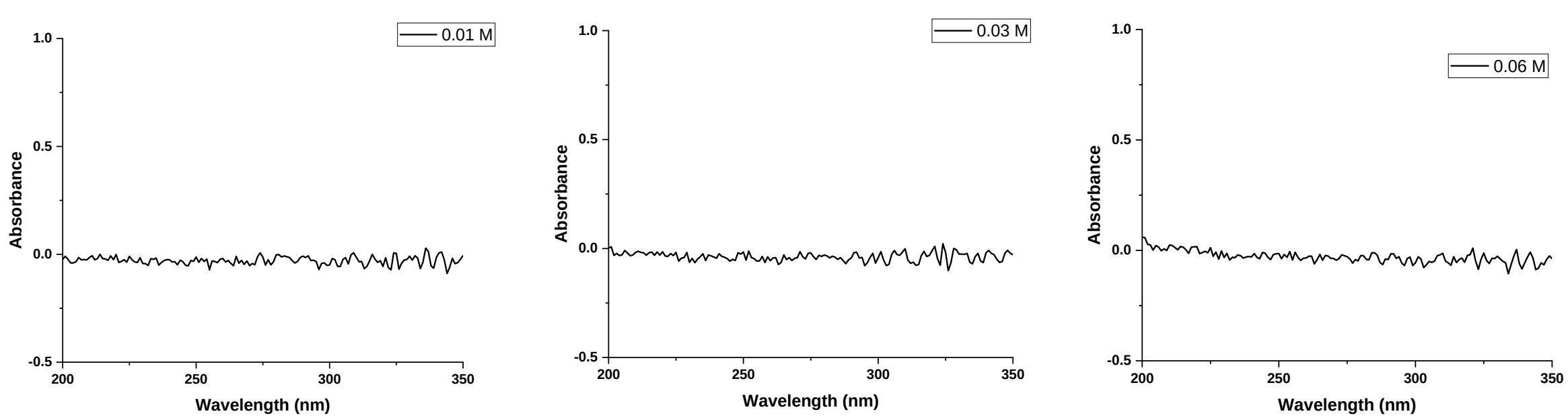

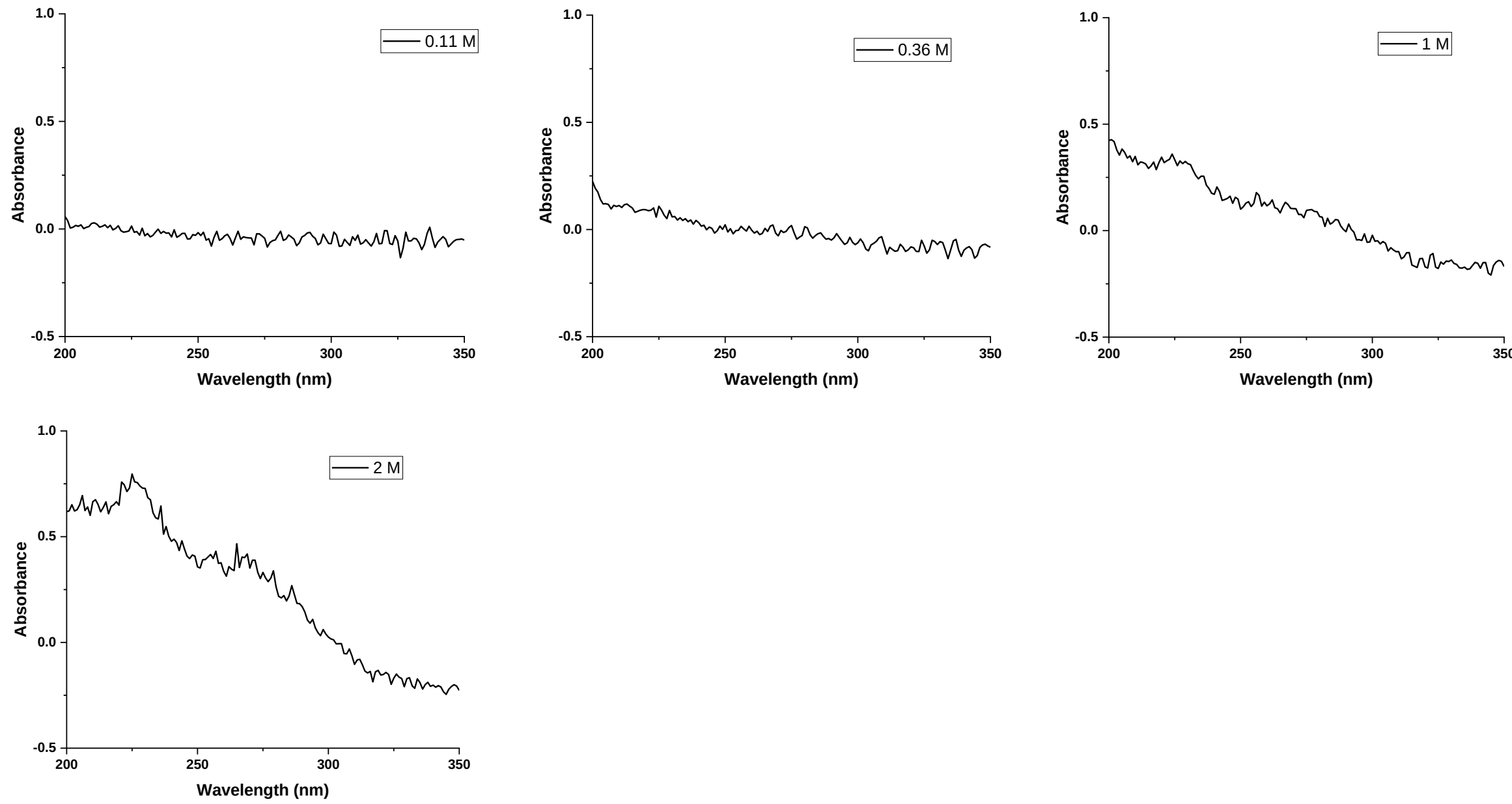

Figure S43. UV-Vis spectra of $NH_4H_2PO_4$ at different concentrations.

UV absorption of DAP before and after centrifugation:

UV-Vis spectrum was recorded for an aqueous DAP solution (0.01 M). Centrifugation of this solution was then conducted at 8000 r/min for 10 min on a centrifuge manufactured by Flying Pigeon. The centrifuged solution was carefully taken out for UV-Vis analysis. The spectra before and after centrifugation looked almost identical (Figure S44).

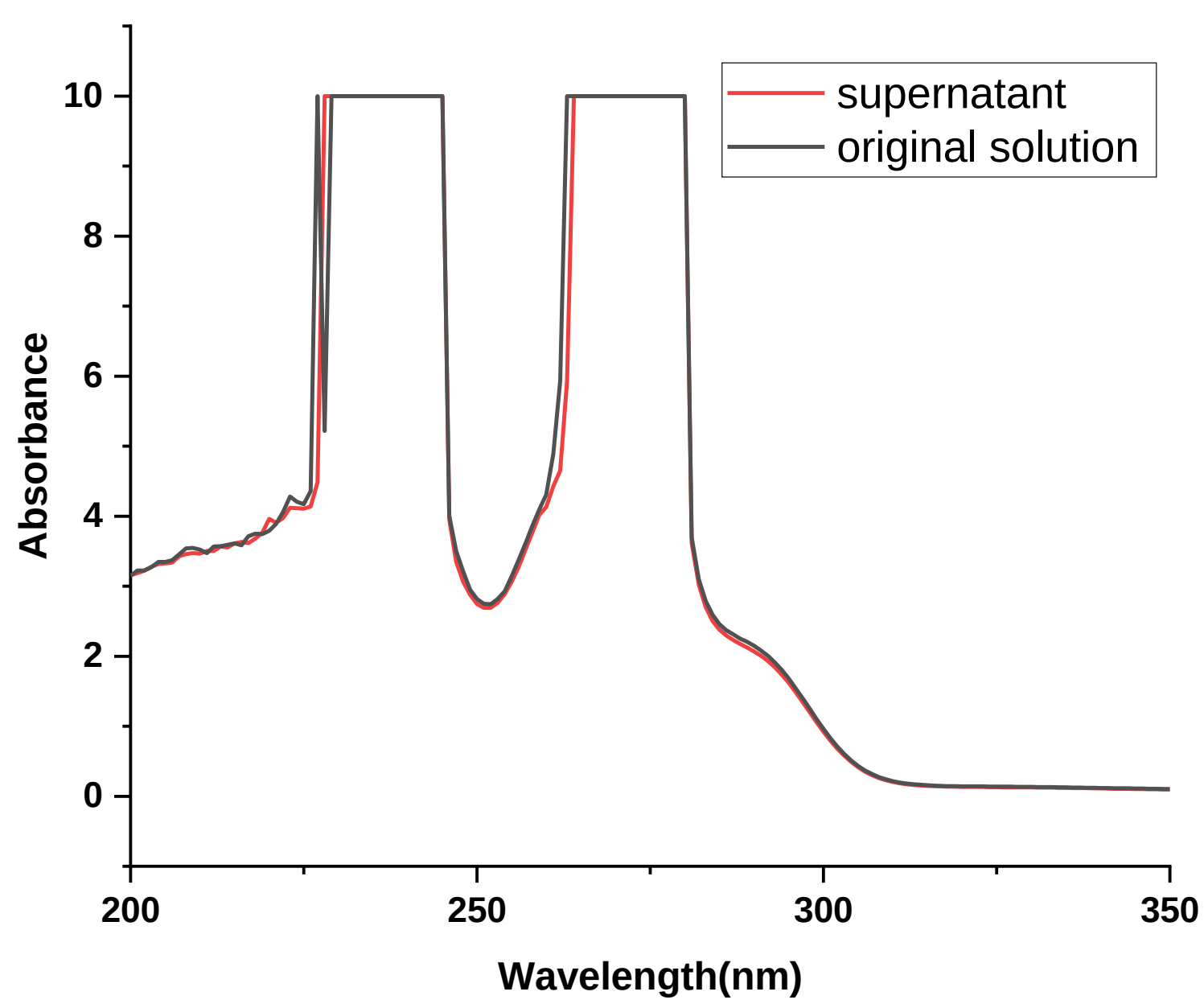

Figure S44. UV spectra of DAP solution before and after centrifugation.

Temperature-dependent UV-Vis absorption measurements of DAP:

Temperature-dependent UV-Vis absorption spectra were recorded using a 0.02 M aqueous solution of DAP (Figure S45). The sample was first measured at room temperature, then heated to 70 °C and measured after thermal equilibration. Subsequently, the sample was allowed to cool naturally back to room temperature, and the UV-Vis spectrum was recorded again to evaluate the reversibility of the temperature-dependent spectral changes. Unlike non-specific aggregates which often undergo irreversible precipitation or ill-defined spectral changes upon heating, our system displayed a fully reversible transition. The sharp J-band gradually broadened into a featureless band at elevated temperatures without reverting to the monomer spectrum. This reversible "disordering" of the J-band into a more heterogeneous ensemble of oligomers argues against non-specific, kinetically trapped clustering. Instead, it supports a dynamic supramolecular system where ordered and less-ordered species exist in a temperature-dependent equilibrium.

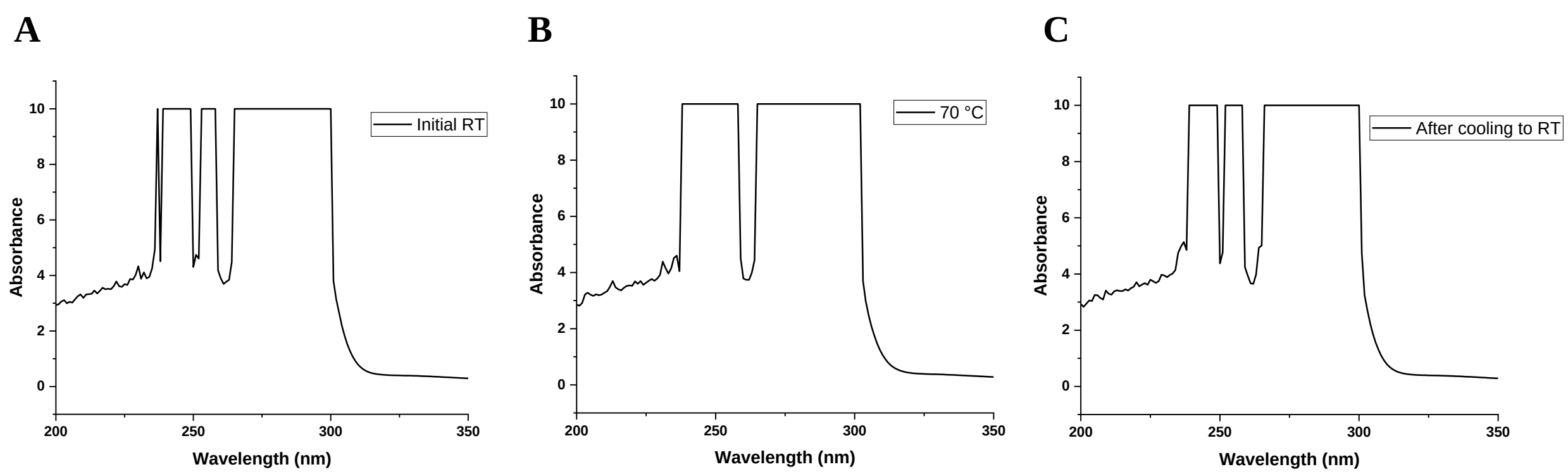

Figure S45. Temperature-dependent UV-Vis absorption spectra of DAP (0.02 M in water). (**A**) At room temperature; (**B**) After heating to 70 °C; (**C**) After cooling back to room temperature.

pH-Dependent UV-Vis absorption of lysine:

To examine the role of protonation state, pH-dependent UV-Vis absorption measurements was performed using lysine as a representative amino acid (Figure S46). Specifically, UV-Vis spectra of 0.05 M lysine were collected at pH 1, 7 and 13. The spectra showed clear pH-dependent changes. At pH 1, lysine displayed only one main absorption feature (centered at ~240 nm). At pH 7, two sharp absorption bands appeared at around 270 nm, indicating that the change in protonation state promotes a more structured absorption response. At pH 13, the absorption bands at 270 nm became more intense, and additional absorption bands also emerged. These results demonstrate that the UV absorption behavior of lysine is strongly affected by pH. Changes in the protonation state of lysine's most basic group (ε-NH_2 on the side chain as the proton buffer) may modulate molecular packing, thereby affecting aggregate formation and optical response.

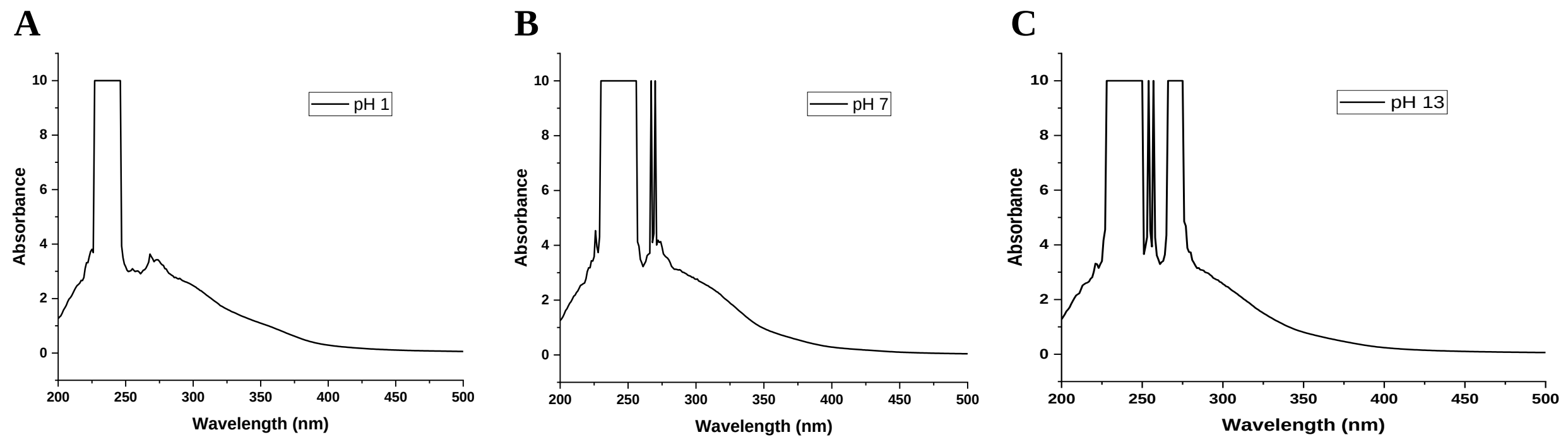

Figure S46. pH-Dependent UV-Vis absorption spectra of lysine. (**A**) pH 1; (**B**) pH 7; (**C**) pH 13.

Circular dichroism evidence for chiral excitonic coupling in AIA aggregates:

Circular dichroism spectroscopy of several chiral AIA molecules (L-lysine, D-lysine, L-cysteine and L-lactic acid) was performed to probe the possible excitonic interactions in ordered chiral aggregates (Figure S47). The CD spectrum showed a bisignate Cotton effect in the absorption region, which is consistent with exciton coupling between ordered transition dipoles in the aggregated state. The CD spectrum of L-lysine exhibited clear concentration dependence. The characteristic bisignate Cotton effect, indicative of chiral aggregation, emerged only above a threshold concentration and its intensity increased with concentration. The opposite signs for L- and D-lysine demonstrate that the optical activity stems from chiral supramolecular packing, not artifacts or non-specific aggregation. These results provide additional spectroscopic evidence for ordered intermolecular exciton coupling.

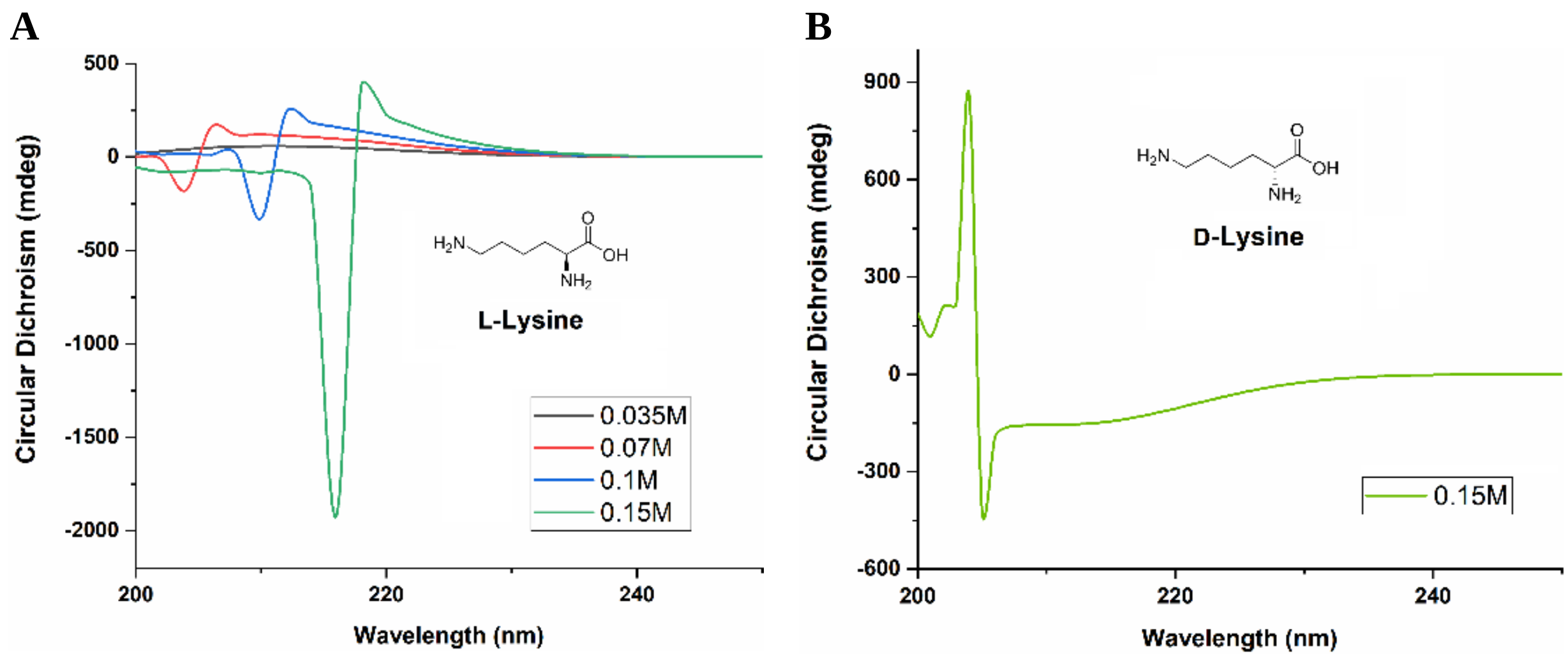

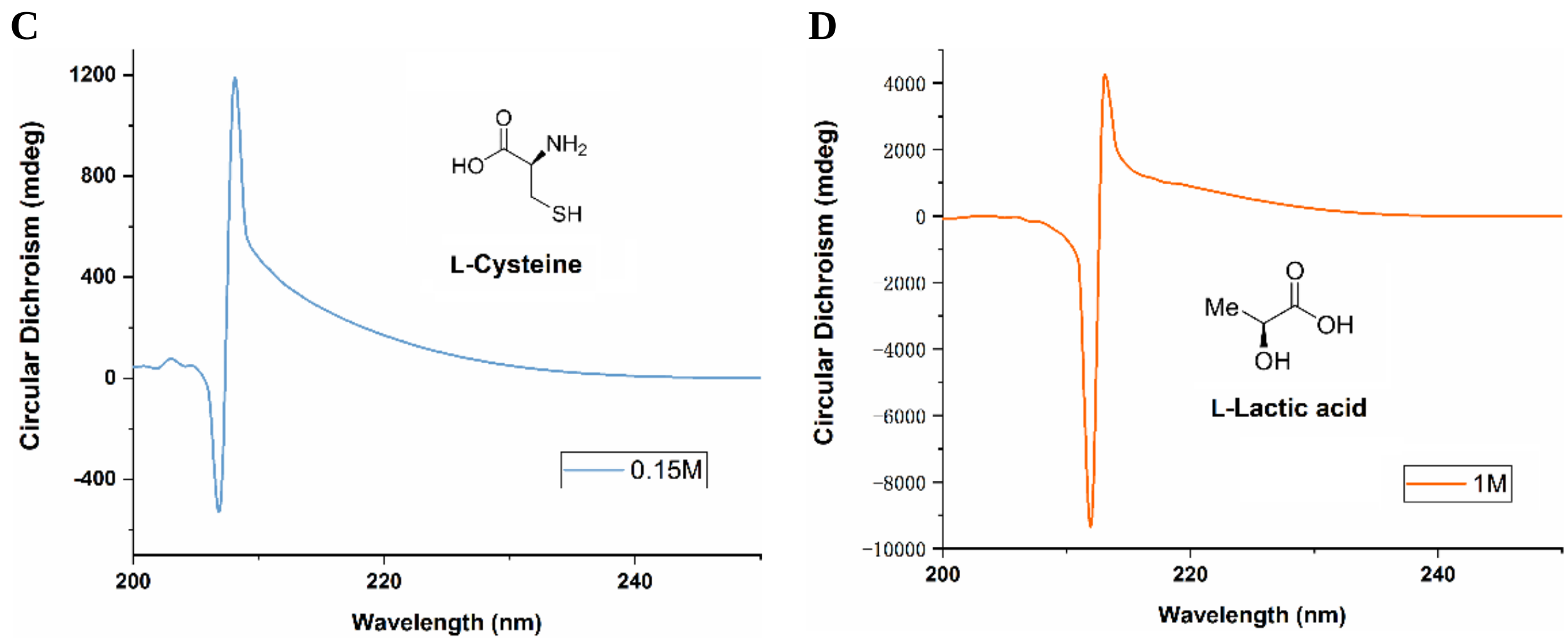

Figure S47. Circular dichroism spectra of chiral AIA-active molecules. (**A**) L-lysine at different concentrations, (**B**) D-lysine, (**C**) L-cysteine (**D**) L-lactic acid.

Concentration-dependent UV-Vis absorption of Cys-Cys dipeptide:

A series of Cys-Cys dipeptide solutions were prepared, with concentrations of 0.01 mM, 0.1 mM, 1 mM, and 10 mM. UV-Vis spectra were recorded for these solutions (Figure S48). At 10 mM, a sharp band showed up at around 230 nm.

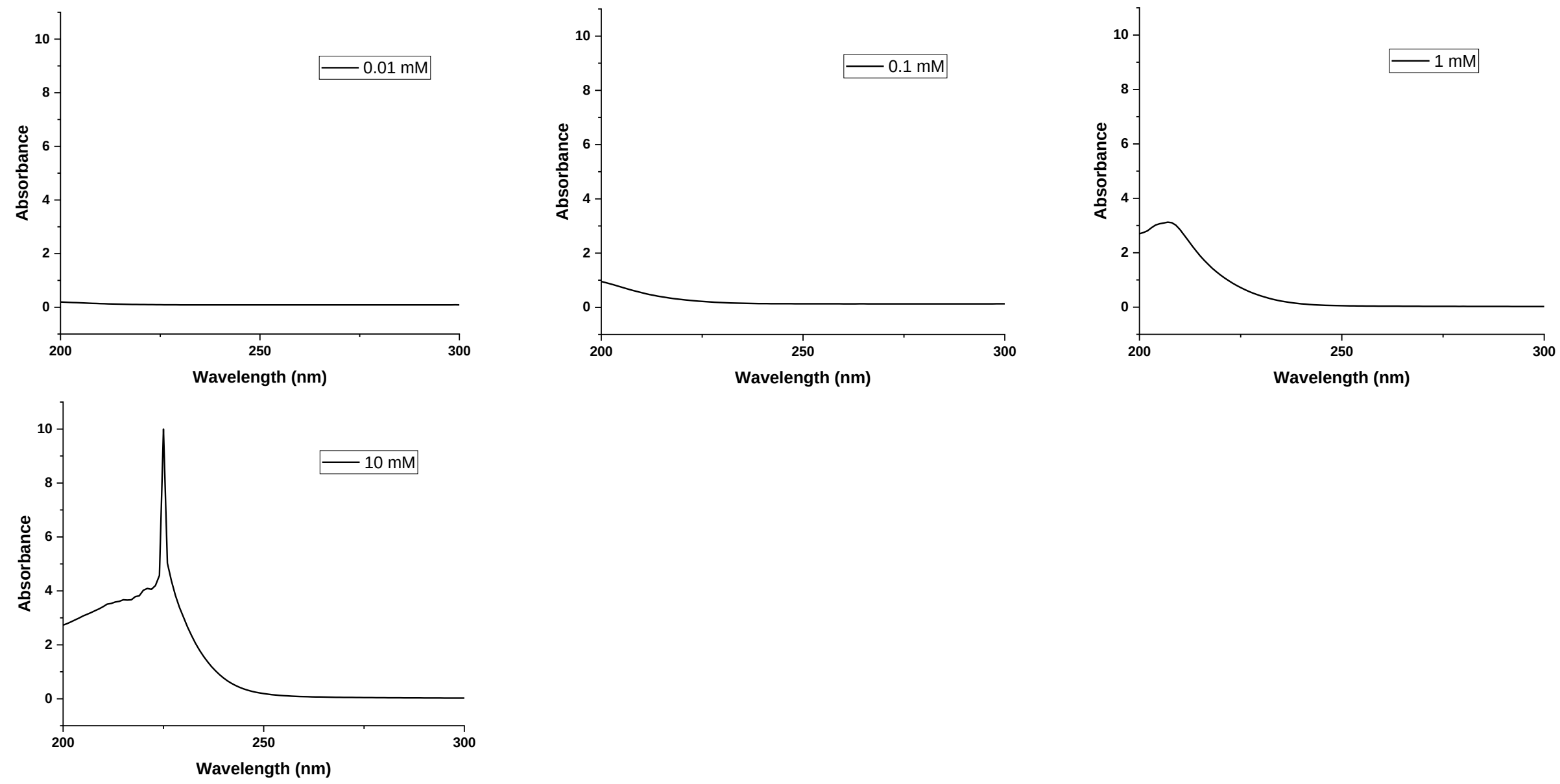

Figure S48. UV-Vis spectra of Cys-Cys dipeptide at different concentrations.

Concentration-dependent UV-Vis absorption of hexapeptide-9:

A series of hexapeptide-9 solutions were prepared, with concentrations of 0.01 mM, 0.1 mM, 1 mM, and 10 mM. UV-Vis spectra were recorded for these solutions (Figure S49). At 10 mM, a narrow band showed up at around 230 nm.

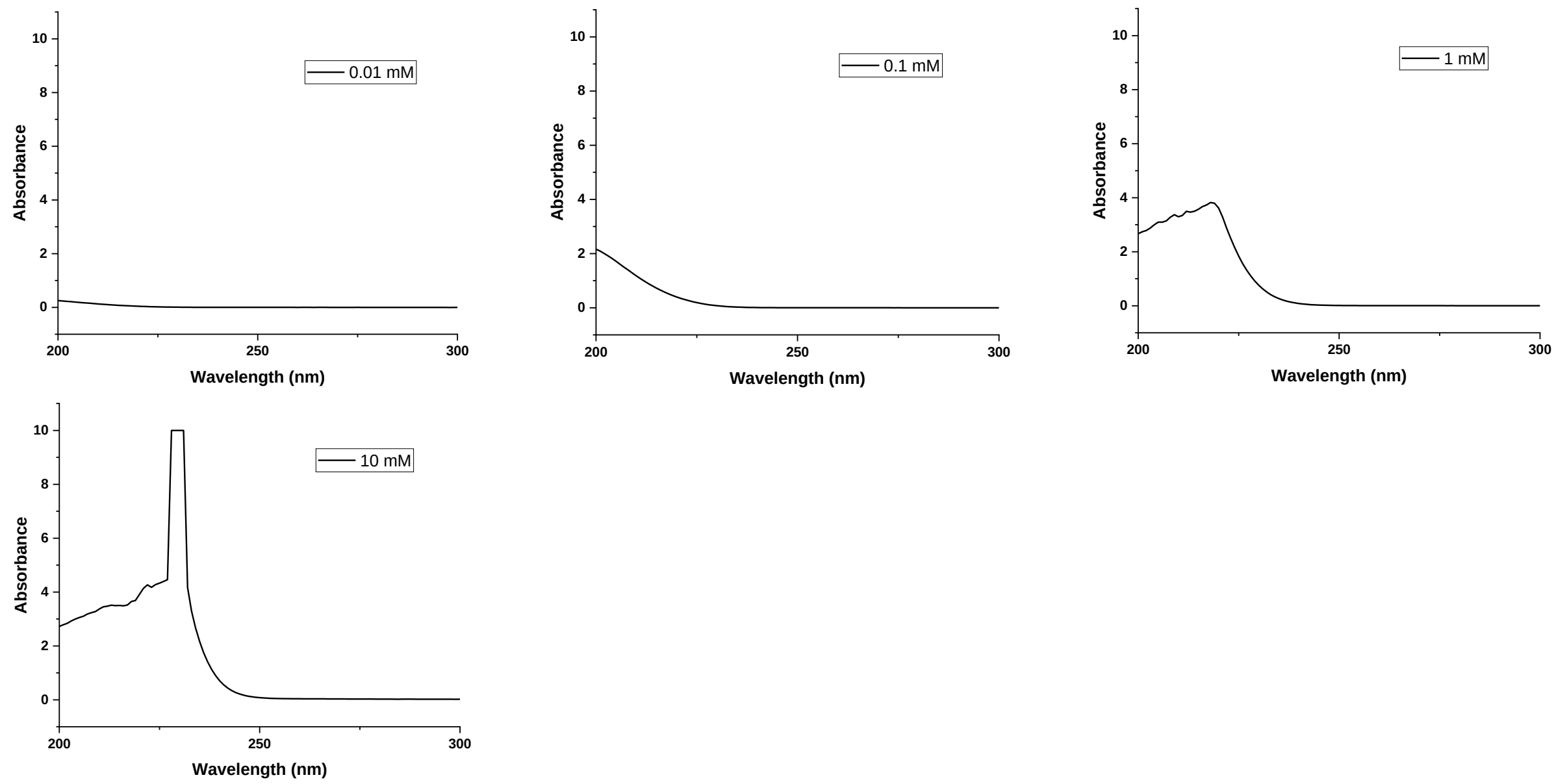

Figure S49. UV-Vis spectra of hexapeptide-9 at different concentrations.

Concentration-dependent UV-Vis absorption of acetyl tetrapeptide-11:

A series of acetyl tetrapeptide-11 solutions were prepared, with concentrations of 0.018 mM, 0.18 mM, and 1.8 mM. UV-Vis spectra were recorded for these solutions (Figure S50). At 1.8 mM, a narrow absorption band showed up at around 235 nm, with a broader new absorption band observed at around 280 nm.

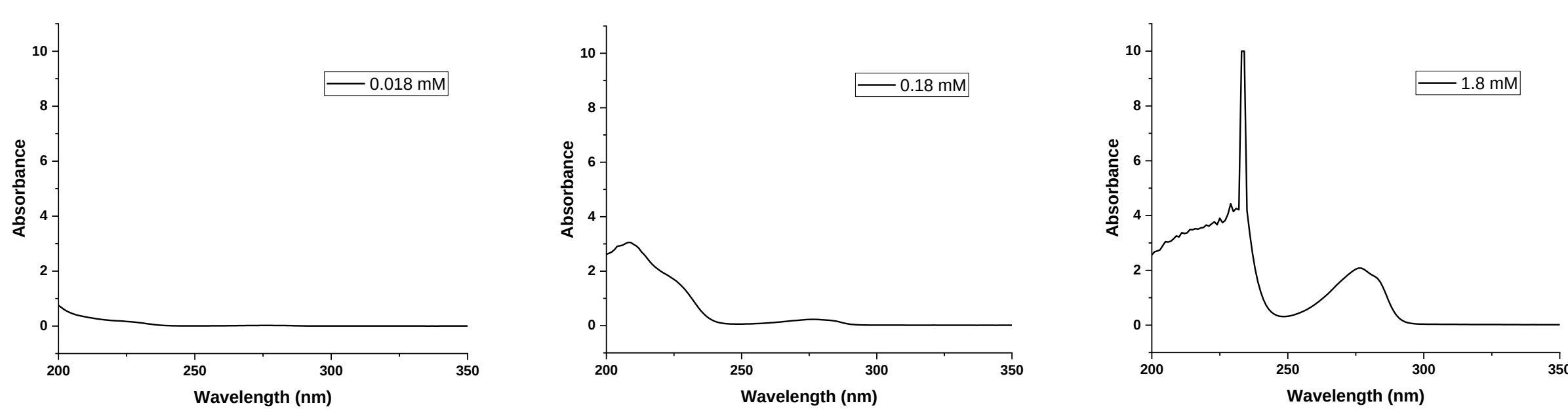

Figure S50. UV-Vis spectra of acetyl tetrapeptide-11 at different concentrations.

Photoprotection of 2-aminooxazole by phosphoramidate:

A set of external and internal quartz vials was used (Figure S51). The internal quartz vial was charged with a 5 mM aqueous 2-aminooxazole solution.[3] The external quartz vial was filled with a 1 M aqueous solution of phosphoramidate, or water. The resulting nested assemblies were irradiated with UV light at 254 nm from two low-pressure mercury vapor lamps, which were placed on both sides of the quartz vials (Figure S51). Reactions were performed at room temperature. Due to heat generation from the lamps, a small electric fan was directed towards the lamp assembly to provide air cooling. HPLC analysis was performed at a flow rate of 1 mL/min at 215 nm. The gradient condition was: 0→3 min, 100% A; 3→25 min, 100% A to 96% A; 25→26 min, 96% A to 100% A. After 8 h irradiation, no significant decomposition of 2-aminooxazole was observed in the presence of BDMDAP or DAP (Figure S52). In contrast, a complete decomposition occurred in the sample with the external vial charged with water.

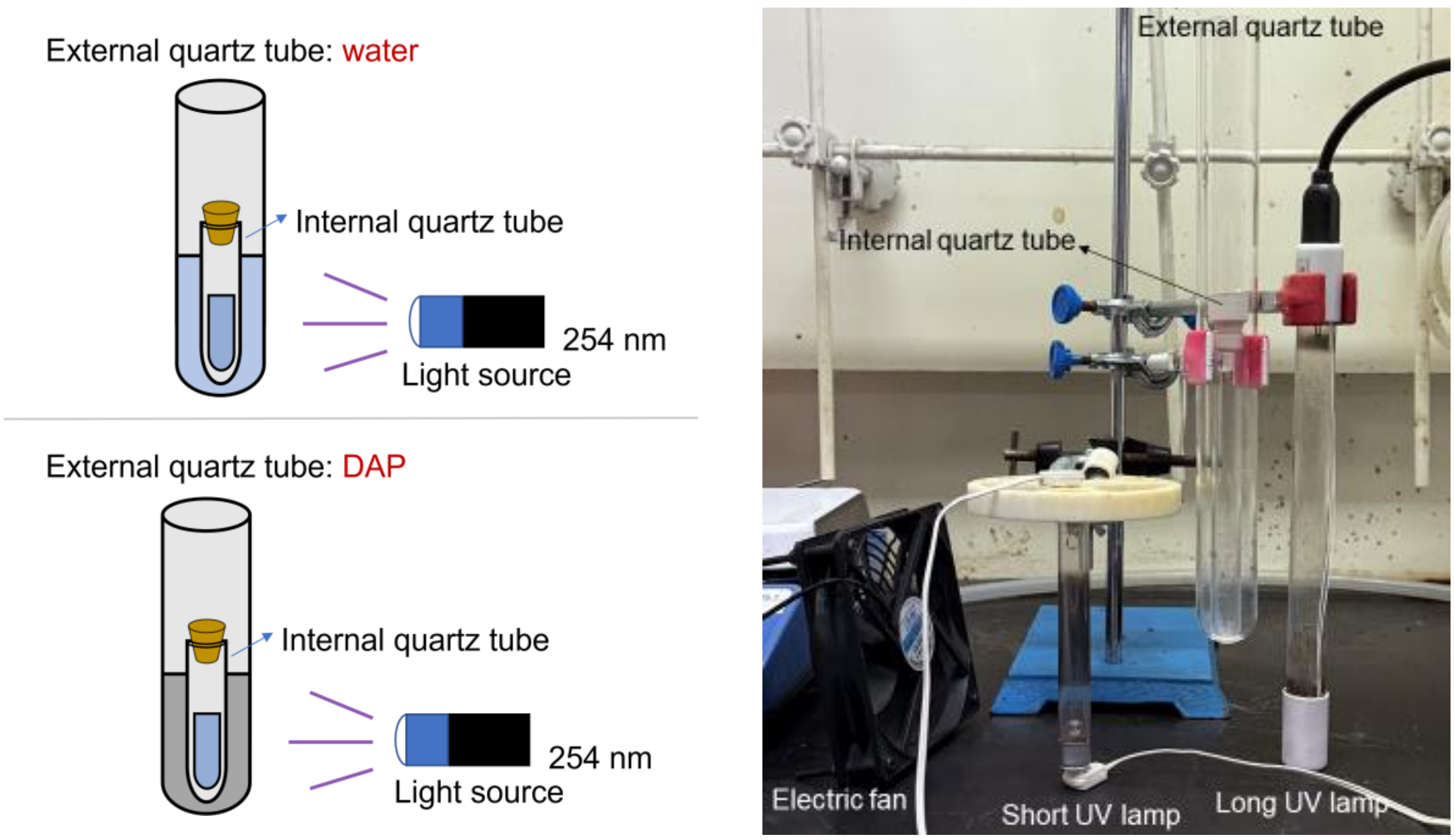

Figure S51. Schematics and photo of the setup of the UV irradiation experiments.

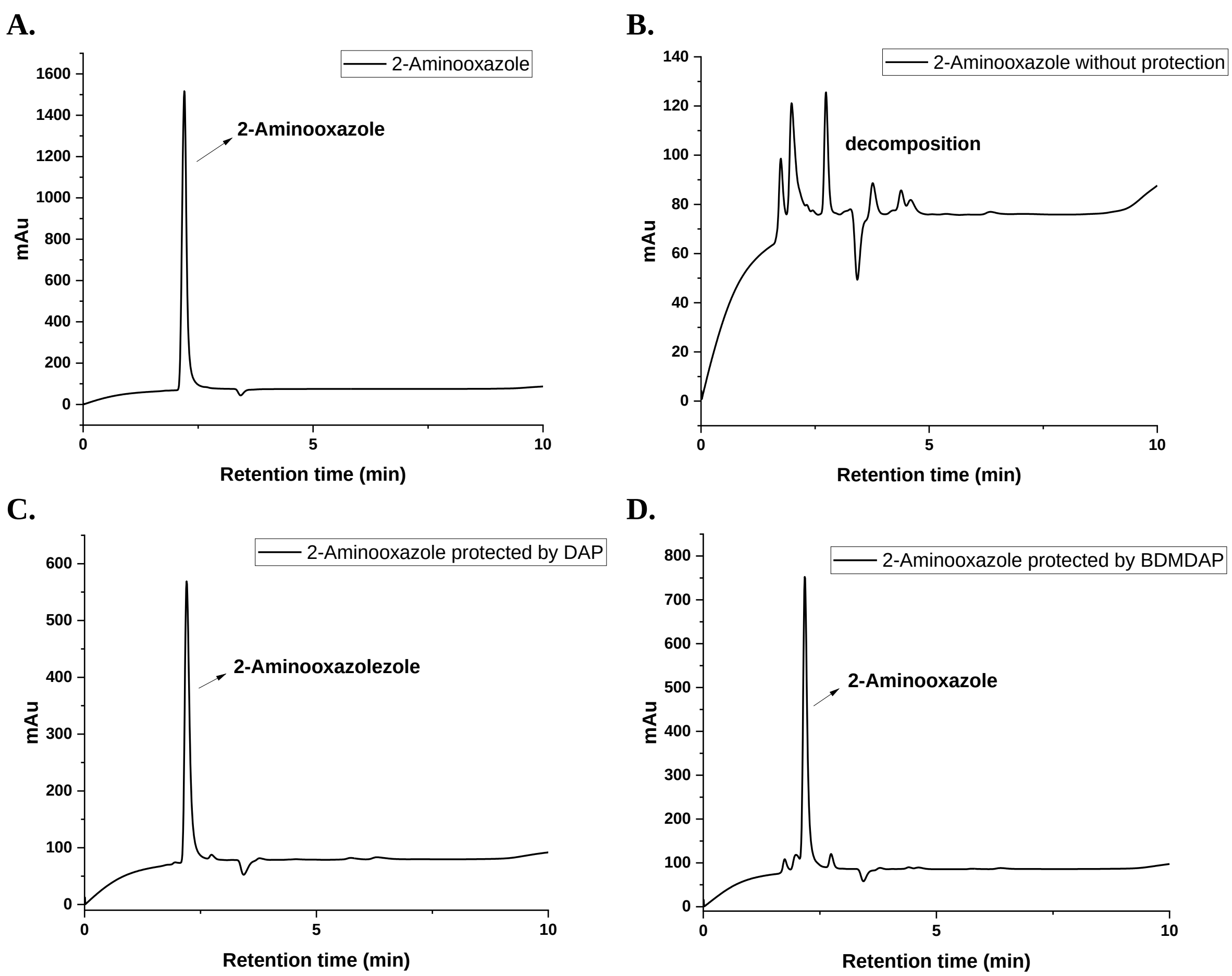

Figure S52. HPLC chromatograms of 2-aminooxazole under different protection conditions. (**A**) Original solution of 2-aminooxazole. (**B**) UV-irradiated solution of 2-aminooxazole without protectant. (**C**) UV-irradiated solution of 2-aminooxazole shieled by DAP solution (1 M). (**D**) UV-irradiated solution of 2-aminooxazole shieled by BDMDAP solution (1 M).

Photoreaction between bicarbonate and sulfite to produce C1 compounds:[4]

A set of external and internal quartz vials was used, same as the setup in Figure S51. The internal quartz vial was charged with 0.02 M $NaHCO_3$ and 0.04 M Na_2SO_3. The external quartz vial was filled with 0.001 M DAP solution, or water. The resulting nested assemblies were irradiated with UV light at 254 nm for 4 h at room temperature. Due to heat generation from the lamps, a small electric fan was directed towards the lamp assembly to provide air cooling. Regardless of the ingredient in the external vial (water or 0.001 M DAP solution), ^{1}H NMR signals attributable to methanol and formic acid were detected for the internal solution (Figure S53).

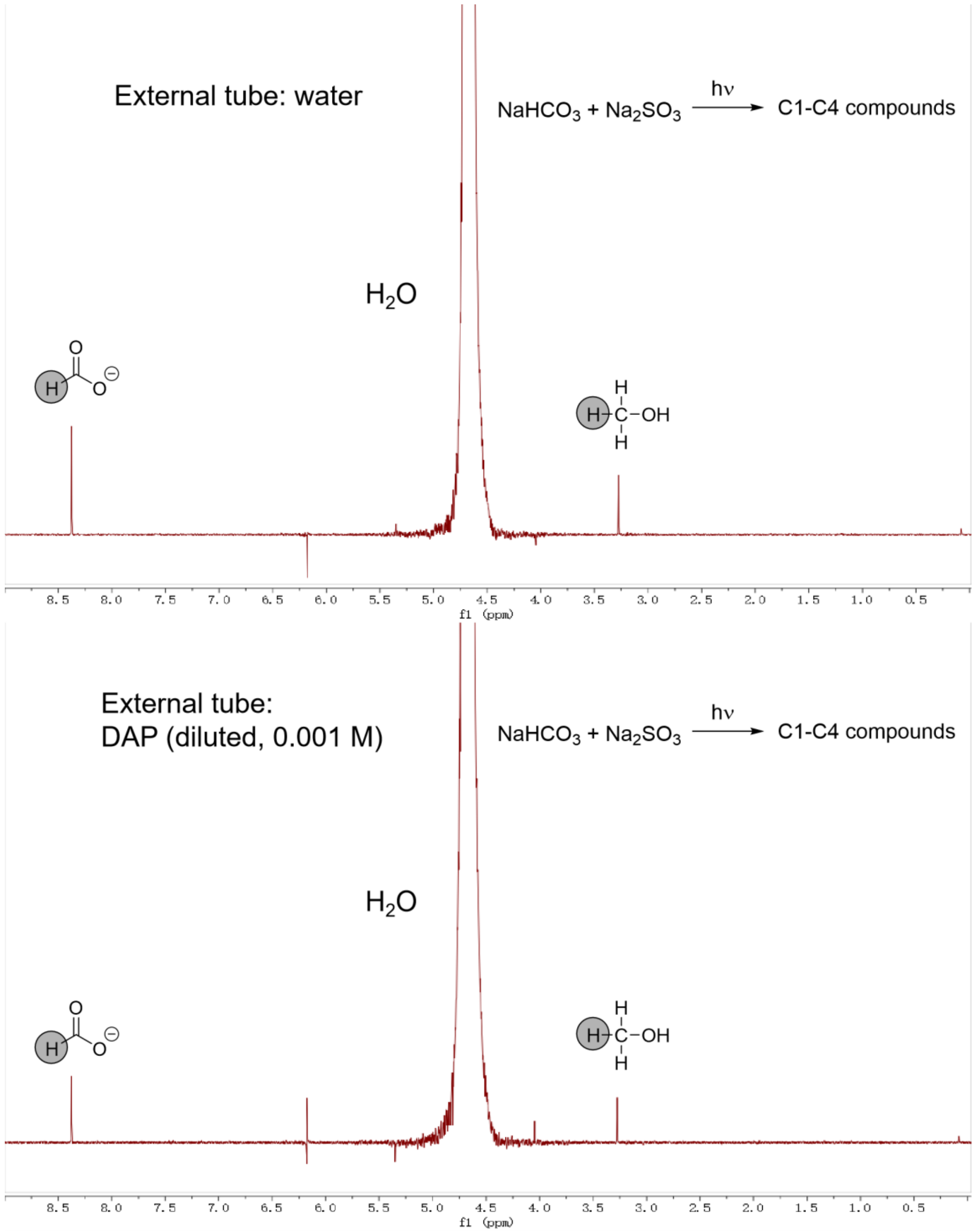

Figure S53. ^{1}H NMR spectra of the reaction mixtures after 4 h irradiation at 254 nm (400 MHz, D_2O). Top: with the external quartz vial containing only water. Bottom: with the external quartz vial containing dilute DAP solution (0.001 M).

On-site photoprotection of an RNA oligomer by DAP:

The RNA oligomer solution was prepared by dissolving 30 μg of a 5-nt RNA (5′-UACGU-3′) in 200 μL of RNase-free water. Two quartz vials (150 mm length, 15 mm inner diameter, 1 mm wall thickness) were each filled with 30 μL of this RNA solution, mixed with either 3 mL of water or 3 mL of 0.03 M DAP solution. Both mixtures were then irradiated using a dual low-pressure Hg lamp set (λ = 254 nm, placed on both sides of the quartz vials for even irradiation) for 4 h. No concentric vials setup was used (Figure S54). Due to heat generation from the lamps, a small electric fan was directed towards the lamp assembly to provide air cooling. The reaction was monitored by HPLC to analyze the RNA solutions before and after UV irradiation, using an injection volume of 50 μL, a detection wavelength of 254 nm, a mobile phase of water (with 0.1% formic acid, as phase A) and acetonitrile (as phase B) at a flow rate of 1 mL/min, and with the gradient elution as follows: 0→10 min, 95% A; 10→12 min, 95% A to 0% A; 12→14 min, 0% A; 14→16 min, 0% A to 95% A. A new peak of a decomposition product at t_R = 3.4 min was detected in the reaction without photoprotectant (Figure S55A). The structure of the decomposition product is shown in Figure S29B. MS (ESI^+): chemical formula: $C_9H_{14}N_3O_9P$; calculated: *m*/*z* 339; found: *m*/*z* 340 $[M+H]^+$, 362 $[M+Na]^+$ (Figure S55B).

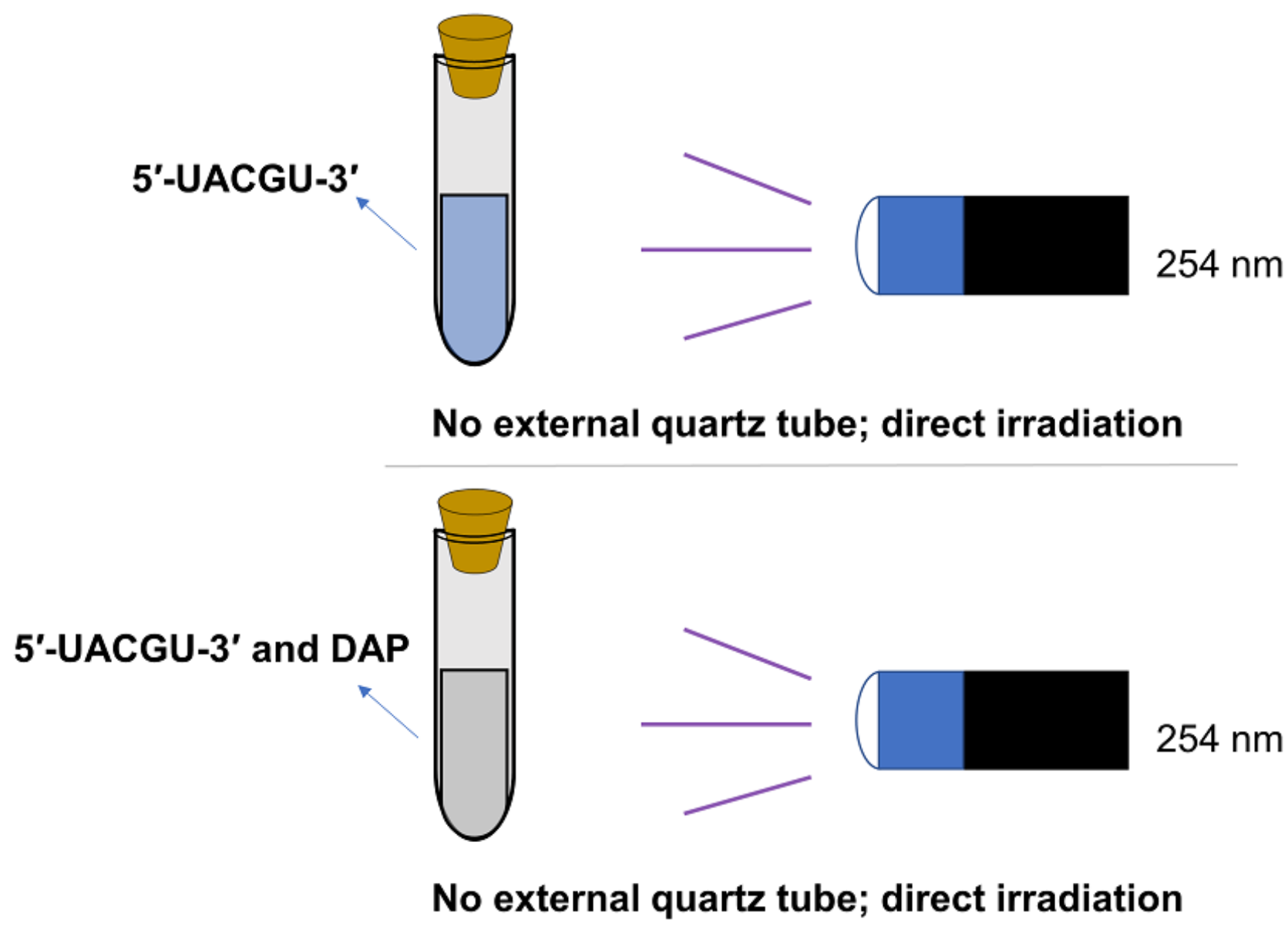

Figure S54. Schematics of the setup of the UV irradiation of an RNA oligomer.

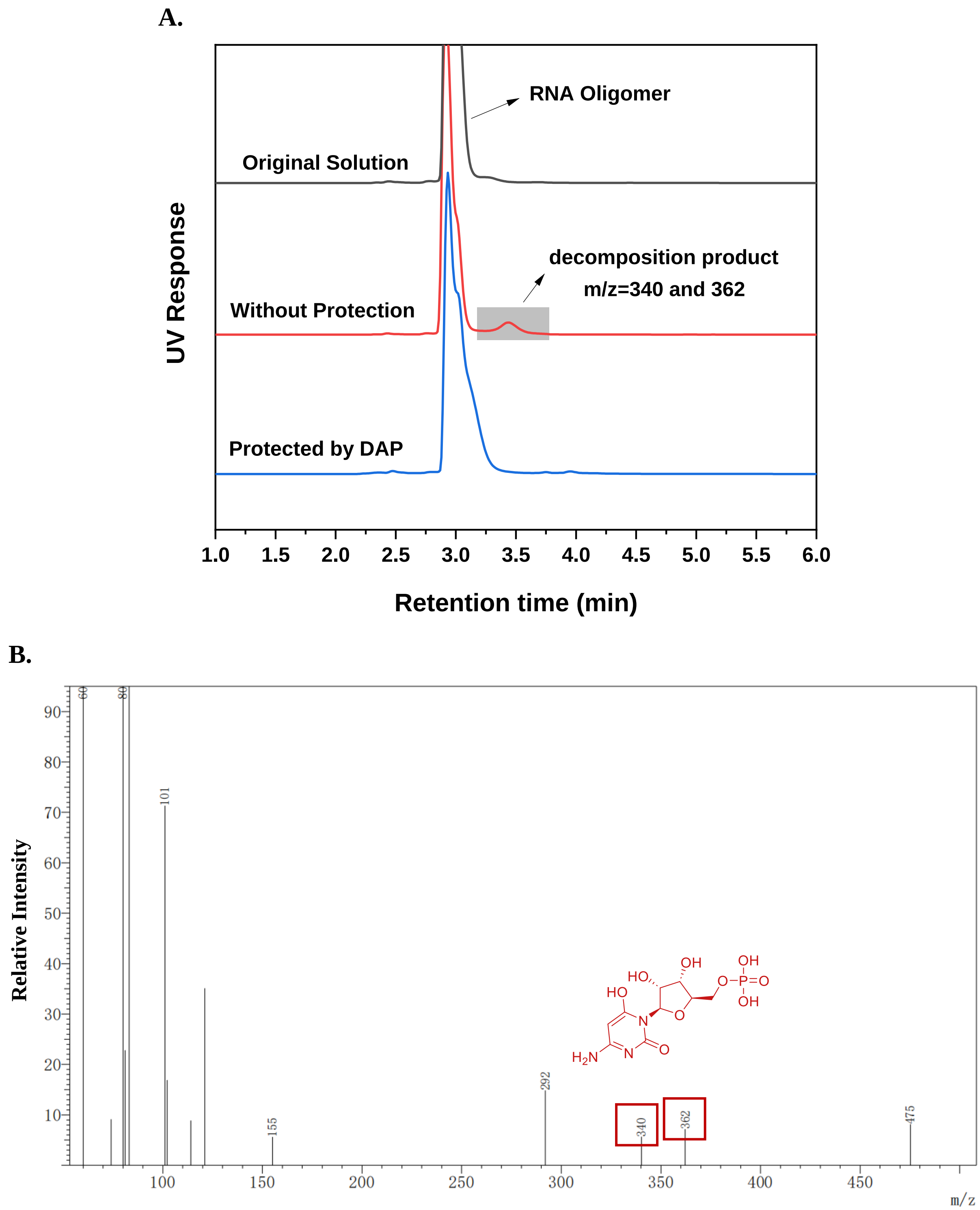

Figure S55. Evaluation of the photoprotective effect of DAP. (**A**) LC traces of solutions of an RNA oligomer (5′-UACGU-3′) in the absence and presence of photoprotectant. (**B**) MS spectrum of the decomposition product (t_R = 3.4 min) in the absence of DAP.

On-site photoprotection of a hexapeptide by cysteine:

Two quartz vials (150 mm length, 15 mm inner diameter, 1 mm wall thickness) were each filled with 1 mL of 5 mM hexapeptide-11 solution, followed by the addition of 3 mL of water to one vial and 3 mL of 0.5 M cysteine solution to the other. Both mixtures were then irradiated using a dual low-pressure Hg lamp set (λ = 254 nm, placed on both sides of the quartz vials for even irradiation) for 4 h. No concentric vials setup was used (Figure S56). Due to heat generation from the lamps, a small electric fan was directed towards the lamp assembly to provide air cooling. Subsequently, the hexapeptide-11 solutions before and after UV irradiation were analyzed by HPLC using an injection volume of 5 μL, a detection wavelength of 254 nm, at a flow rate of 1 mL/min, and with the gradient elution as follows: 0→10 min, 95% A; 10→12 min, 95% A to 0% A; 12→14 min, 0% A; 14→16 min, 0% A to 95% A. A new peak of a decomposition product at t_R = 15.1 min was detected in the reaction without photoprotectant (Figure S57A). The structure of the decomposition product is shown in Figure S31B. MS (ESI^+): chemical formula: $C_{16}H_{25}N_3O_2$; calculated: *m*/*z* 291; found: *m*/*z* 292 $[M+H]^+$ (Figure S57B).

An additional control experiment was performed using ammonium dihydrogen phosphate ($NH_4H_2PO_4$), a phosphorus compound that shows little/no AIA effect and has no potential undesired reaction with the substrate. The control protection experiment was conducted under the same conditions as the protection experiments reported in the manuscript, using hexapeptide-11 as the substrate. Three samples were compared: (1) the original hexapeptide-11 solution without UV irradiation, (2) hexapeptide-11 after UV irradiation without protection, and (3) hexapeptide-11 after UV irradiation in the presence of $NH_4H_2PO_4$.

HPLC analysis showed that the non-irradiated hexapeptide-11 solution displayed a single major peak at 14.4 min. After UV irradiation without protection, an additional peak appeared at 15.0 min, indicating partial photodegradation of hexapeptide-11. In the $NH_4H_2PO_4$ group, the degradation peak at approximately 15.0 min was still observed, indicating that $NH_4H_2PO_4$ did not effectively suppress the photodegradation pathway (Figure S58). These results indicate that a molecule with little/no AIA effect does not provide comparable photoprotection to those with aggregation-induced UV absorption.

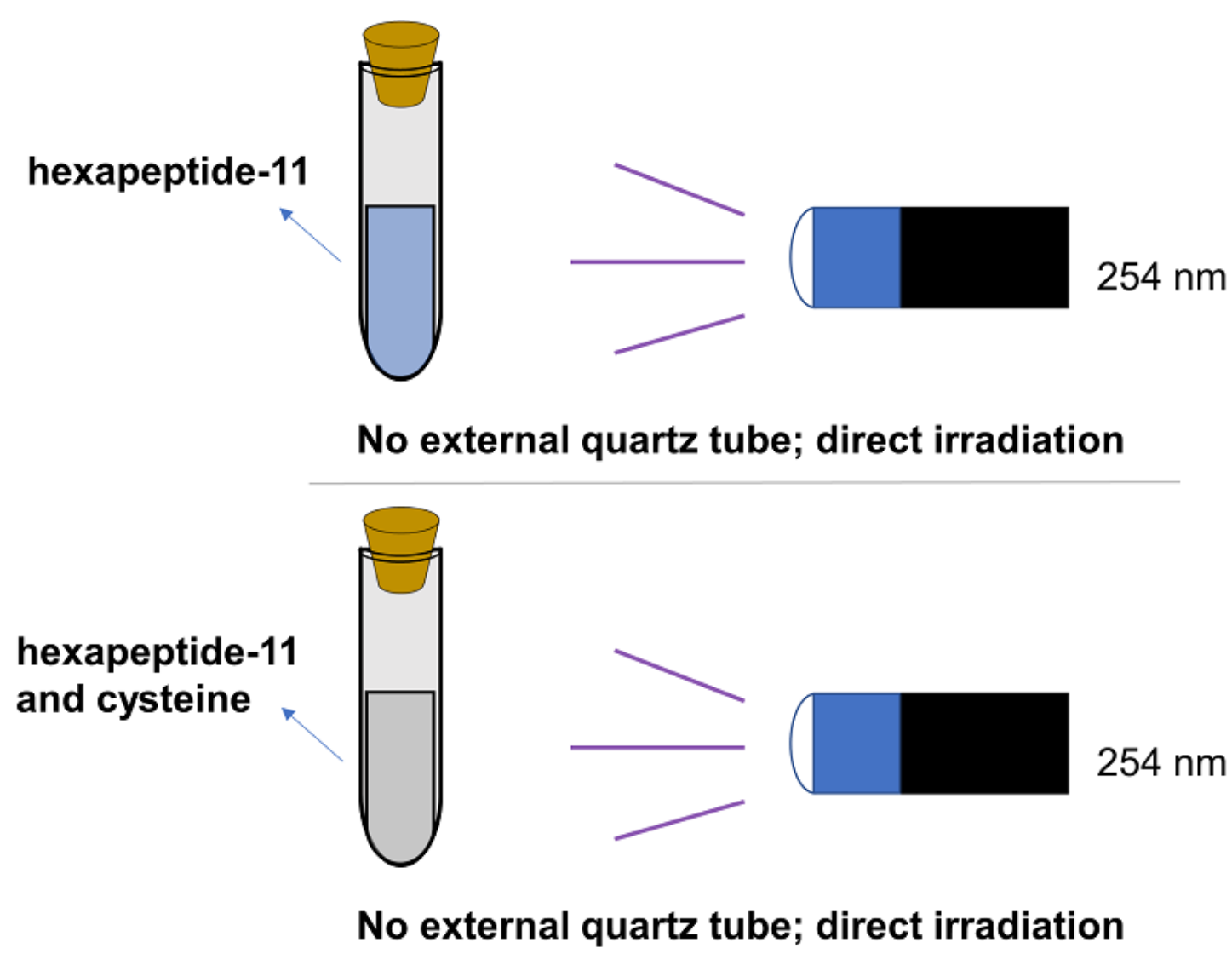

Figure S56. Schematics of the setup of the UV irradiation of a hexapeptide.

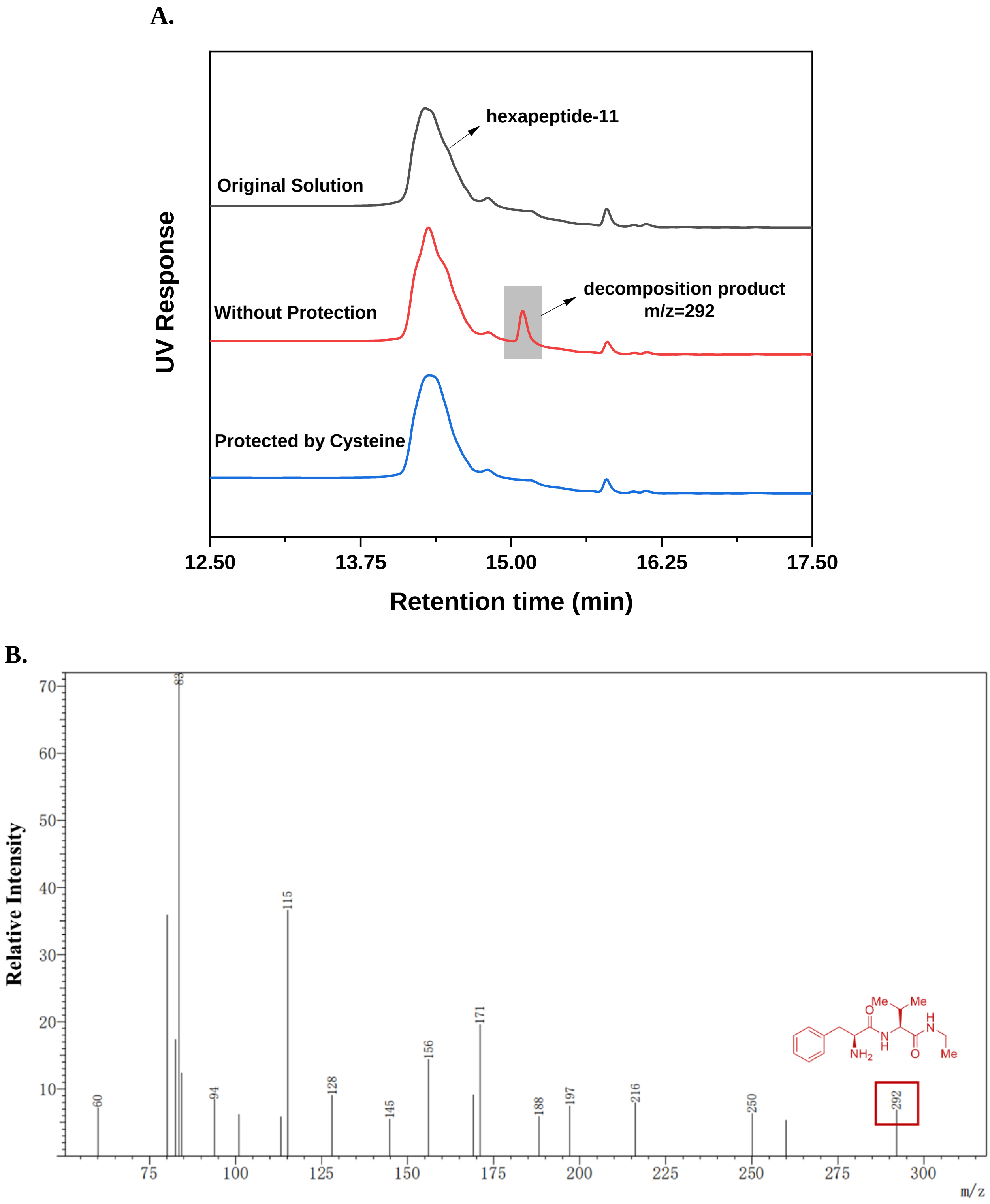

Figure S57. Evaluation of the photoprotective effect of cysteine. (**A**) LC traces (254 nm) of solutions of hexapeptide-11 in the absence and presence of photoprotectant. (**B**) MS spectrum of the decomposition product (t_R = 15.1 min) in the absence of cysteine.

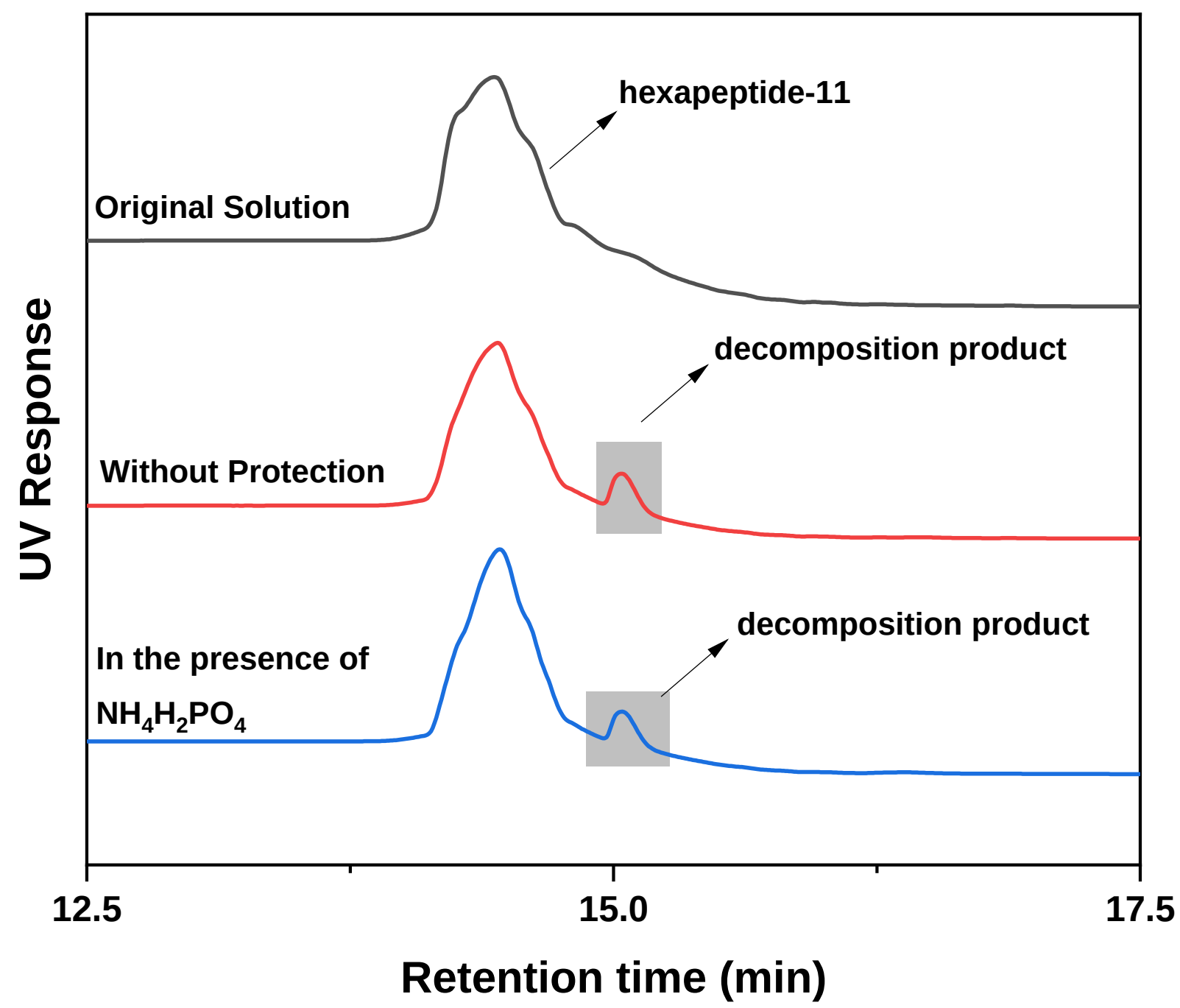

Figure S58. LC traces (254 nm) of the control photoprotection experiment with $NH_4H_2PO_4$.

Dynamic light scattering (DLS) analysis of DAP solution:

A DAP solution (0.01 M) was prepared and filtered with a syringe filter (0.22 μm). The DLS measurement was performed on a Brookhaven (USA) DLS instrument for 121 s, with a baseline difference of 57.563% and a data retention rate of 100.00%. Other key parameters were: temperature 25.0 °C, laser wavelength 532.0 nm, detection angle 90.0°, solvent refractive index 1.334. The results showed a hydrodynamic effective diameter of 932.6 nm (Figure S59).

STOPPED		Samples:	2.1430e+07
Signal:	Auto A	Atot:	2.6427e+05
Run:	234	A CR (avg.):	2.5 kcps
First Delay:	5.0 μsec	Normalization:	M. Base
Last Delay:	100.0 msec	M. Base:	5.1349e+03
Elapsed Time:	00:02:01	C. Base:	3.2590e+03
Wavelength:	532.0 nm	Base diff:	57.563%
Angle:	90.0	Dust Cutoff:	N/A
Temp:	25.0 deg C	Data Ret:	100.00%
Liquid:	Water	Eff Dia:	932.6 nm
Viscosity:	0.890 cP	Poly:	0.459
Ref Index:	1.334		

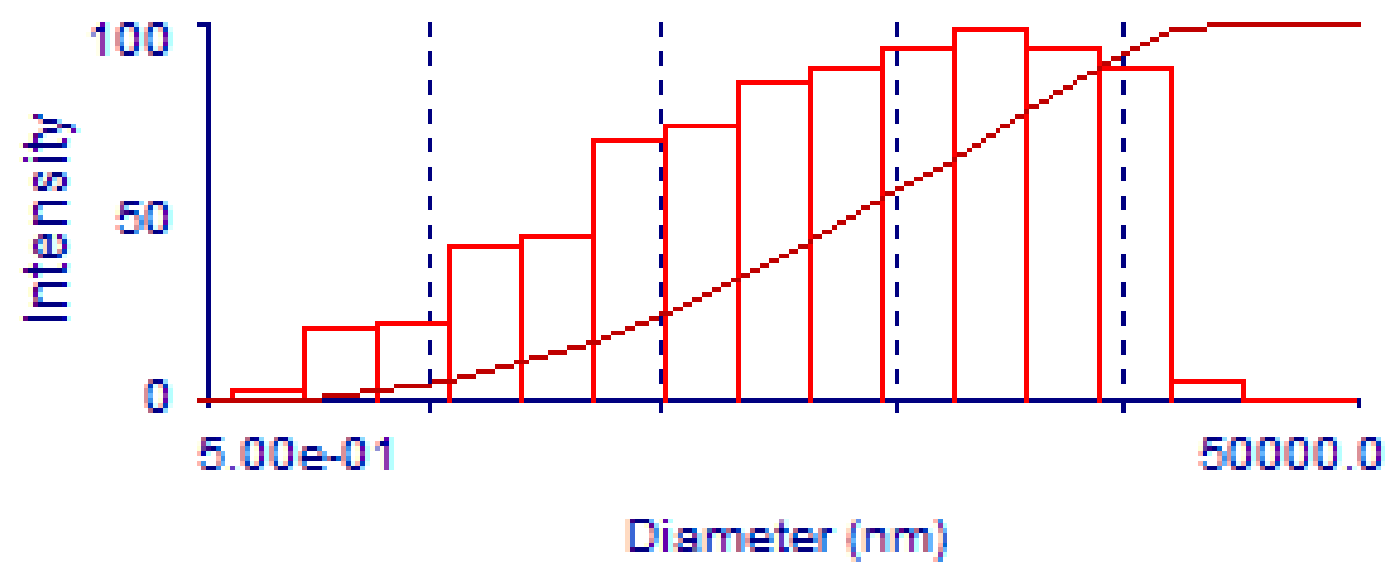

d	G(d)	C(d)	d	G(d)	C(d)	d	G(d)	C(d)
0.10	0	0	273.84	90	54			
0.21	0	0	562.34	95	65			
0.42	0	0	1154.78	100	77			
0.87	4	0	2371.37	95	89			
1.78	20	3	4869.67	90	99			
3.65	21	5	10000.00	6	100			
7.50	42	10						
15.40	45	16						
31.62	71	24						
64.94	75	33						
133.35	86	43						

Figure S59. DLS analysis of an aqueous solution of DAP (0.01 M).

Dynamic light scattering (DLS) analysis of lysine solution:

A lysine solution (0.6 M) was prepared and filtered with a syringe filter (0.22 µm). The DLS measurement was performed on a Brookhaven (USA) DLS instrument for 120 s, with a baseline difference of -0.082% and a data retention rate of 98.47%. Other key parameters were: temperature 25.0 °C, laser wavelength 532.0 nm, detection angle 90.0°, solvent refractive index 1.334. The results showed a hydrodynamic effective diameter of 144.2 nm (Figure S60).

STOPPED			
Signal:	Auto A	Atot:	1.1961e+06
Run:	61	A CR (avg.):	11.4 kcps
First Delay:	5.0 µsec	MS 0 Chan:	Undefined
Last Delay:	100.0 msec	Normalization:	C. Base
Elapsed Time:	00:02:00	M. Base:	6.7849e+04
Wavelength:	532.0 nm	C. Base:	6.7904e+04
Angle:	90.0	Base diff:	-0.082%
Temp:	25.0 deg C	Dust Cutoff:	30.00
Liquid:	Water	Data Ret:	98.47%
Ref Index:	1.334	Eff Dia:	144.2 nm
Samples:	2.1070e+07	Poly:	0.261

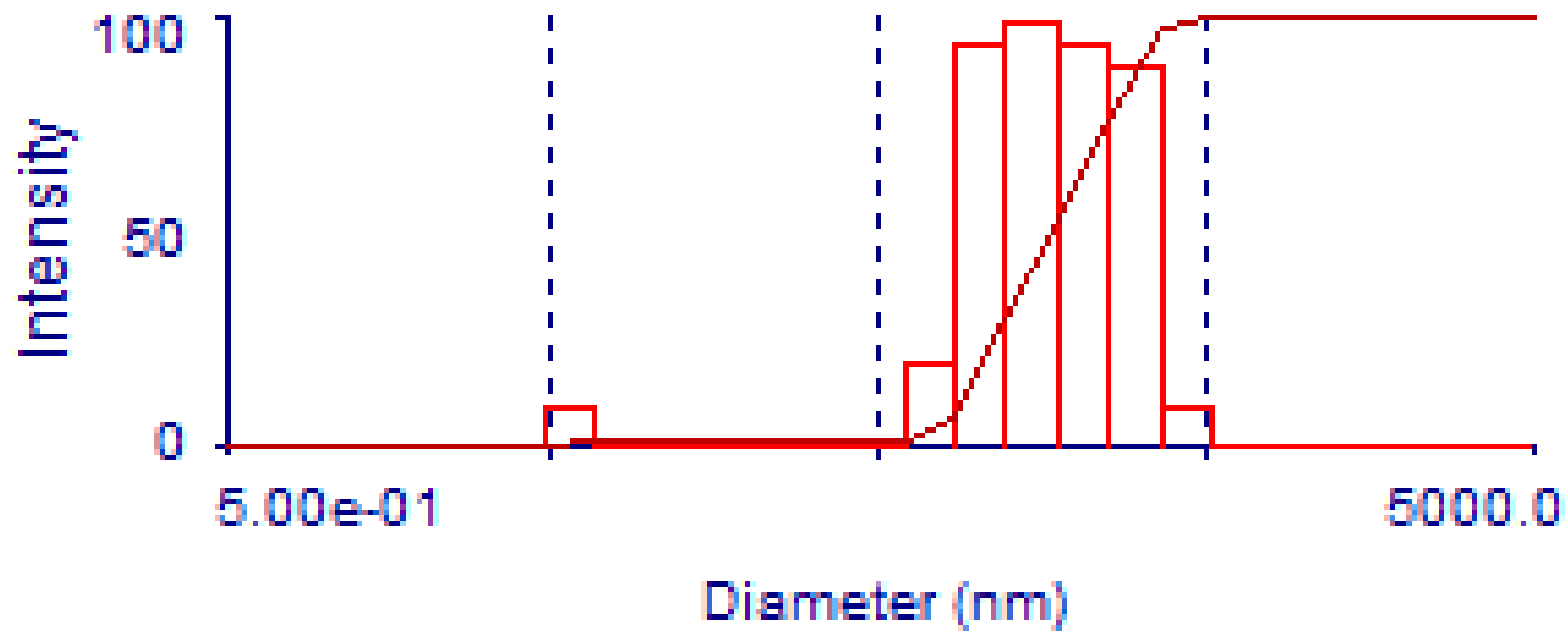

d	G(d)	C(d)	d	G(d)	C(d)	d	G(d)	C(d)
2.67	0	0	142.10	100	54			
3.83	0	0	203.94	95	76			
5.50	10	2	292.69	90	98			
7.89	0	2	420.07	9	100			
11.33	0	2	602.89	0	100			
16.26	0	2	865.26	0	100			
23.34	0	2						
33.49	0	2						
48.07	0	2						
68.99	20	7						
99.01	95	30						

Figure S60. DLS analysis of an aqueous solution of lysine (0.6 M).

Dynamic light scattering (DLS) analysis of filtered water:

To exclude the possibility that the DLS signals originated from contaminants introduced by the syringe filter, a control experiment was performed, in which water was passed through the syringe filter. No reliable particle-size distribution or aggregate-related DLS signal was detected for the filtered water, indicating that the observed DLS response does not arise from filter-derived contaminants (Figure S61).

STOPPED		Samples:	3.3751e+07
Signal:	Auto A	Atot:	5.3946e+04
Run:	224	A CR (avg.):	319.7 cps
First Delay:	5.0 μsec	Normalization:	M. Base
Last Delay:	100.0 msec	M. Base:	8.6642e+01
Elapsed Time:	00:03:01	C. Base:	8.6223e+01
Wavelength:	532.0 nm	Base diff:	0.485%
Angle:	90.0	Dust Cutoff:	30.00
Temp:	25.0 deg C	Data Ret:	98.38%
Liquid:	Water	Eff Dia:	0.1 nm
Viscosity:	0.890 cP	Poly:	0.030
Ref Index:	1.334		

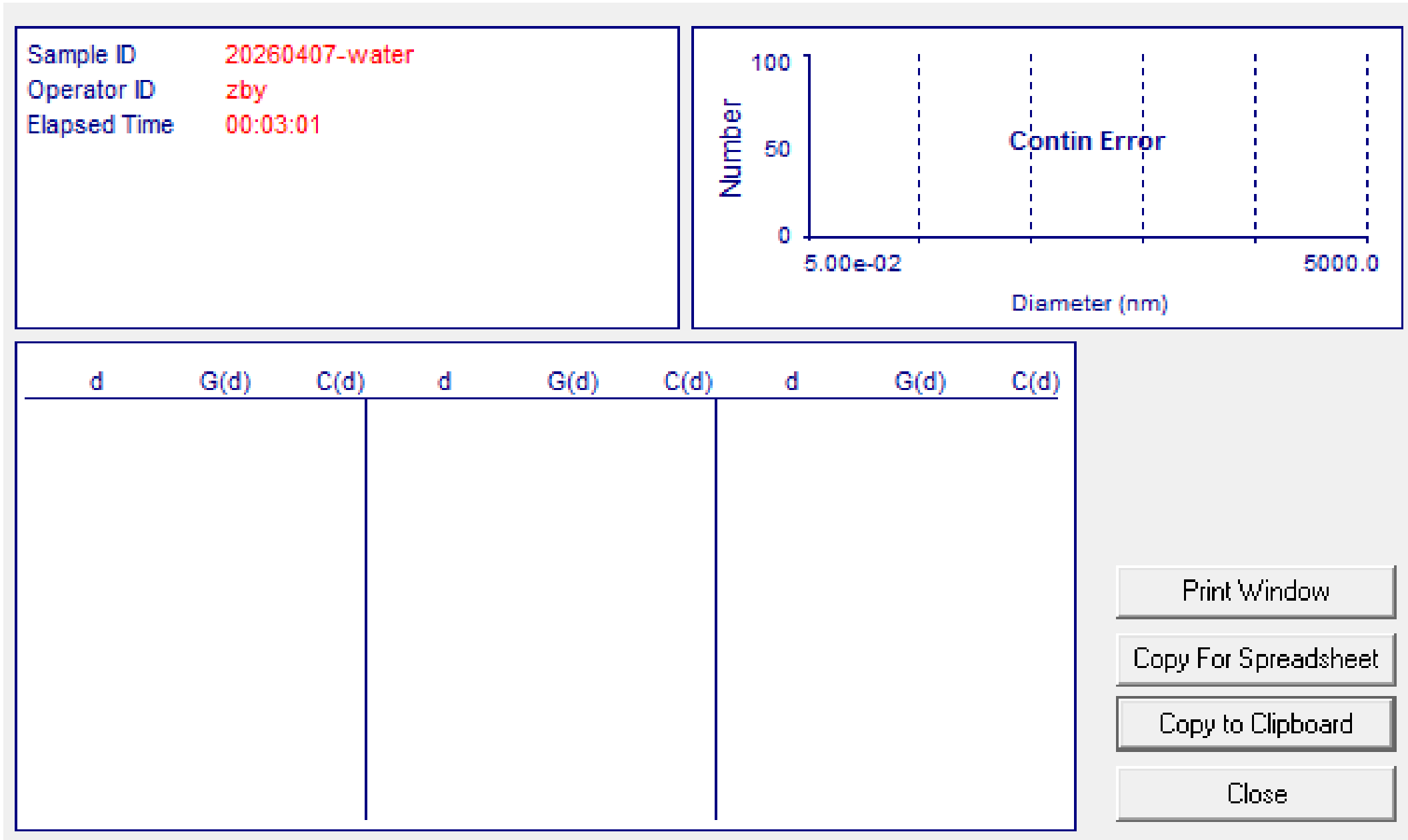

Figure S61. DLS analysis of water that passed through syringe filter.

Dynamic light scattering (DLS) analysis of glyceraldehyde:

In contrast to the DLS results of DAP, glyceraldehyde did not show any aggregate population (Figure S62). The weak signals were close to the lower detection/background range of the DLS measurement, and did not correspond to a stable nano/submicron-sized particle population.

STOPPED		Samples:	3.3653e+07
Signal:	Auto A	Atot:	7.5640e+04
Run:	226	A CR (avg.):	449.5 cps
First Delay:	5.0 μsec	Normalization:	M. Base
Last Delay:	100.0 msec	M. Base:	1.7706e+02
Elapsed Time:	00:03:00	C. Base:	1.7002e+02
Wavelength:	532.0 nm	Base diff:	4.141%
Angle:	90.0	Dust Cutoff:	30.00
Temp:	25.0 deg C	Data Ret:	98.85%
Liquid:	Water	Eff Dia:	1.8 nm
Viscosity:	0.890 cP	Poly:	0.475
Ref Index:	1.334		

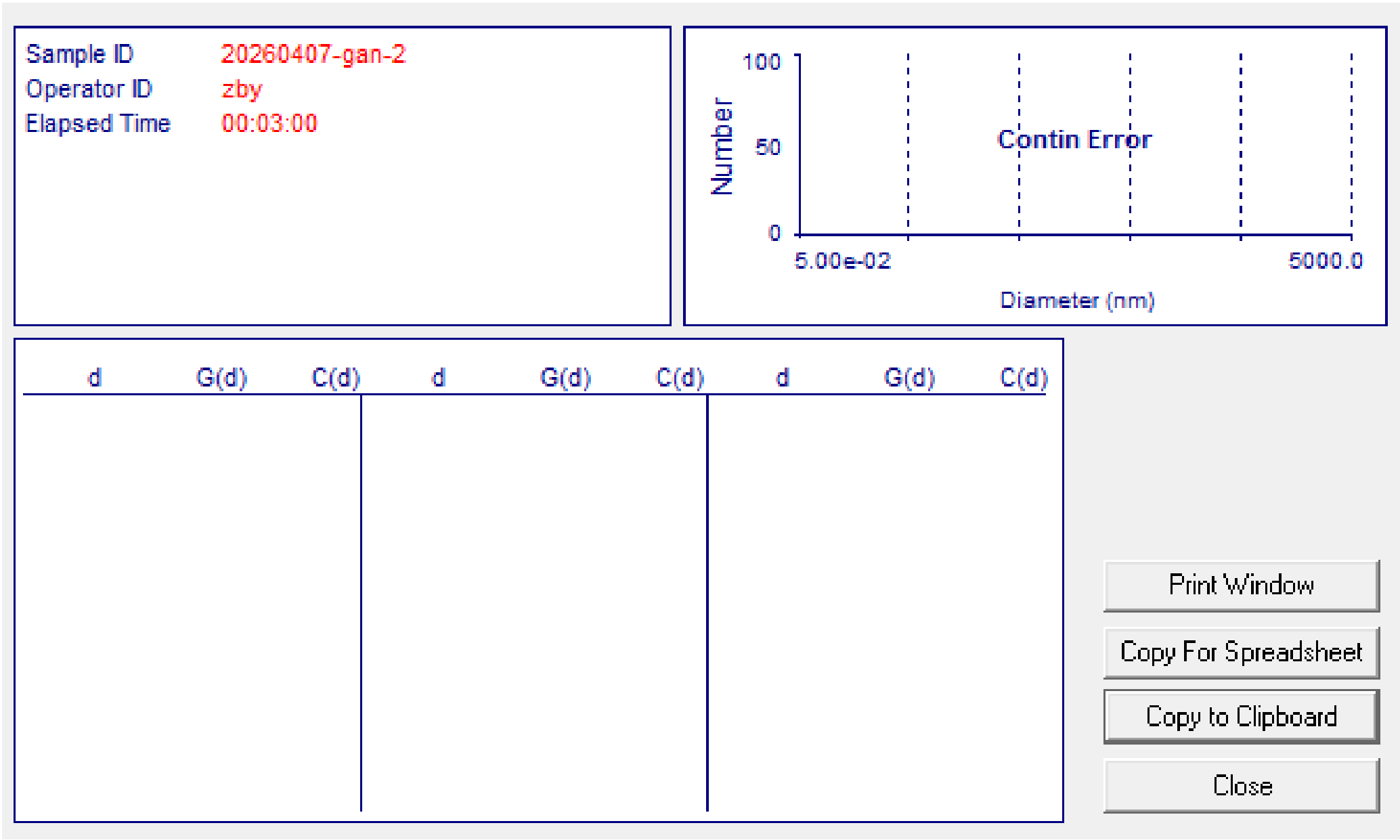

Figure S62. DLS analysis of glyceraldehyde (0.1 M in water).

Intrinsic photostability test for lactic acid:

A solution of lactic acid (3 M, 5 mL) was transferred into a quartz vial (150 mm in length, 15 mm inner diameter, 1 mm wall thickness), which was sealed with a stopper to prevent evaporation. The sample was irradiated using a dual low-pressure Hg lamp set (λ = 254 nm, placed on both sides of the quartz vials for even irradiation) for 12 h. No concentric vials setup was used. Due to heat generation from the lamps, a small electric fan was directed towards the lamp assembly to provide air cooling. The solutions before and after irradiation were analyzed by high-performance liquid chromatography (HPLC) to evaluate the intrinsic photostability of lactic acid.

HPLC analysis was performed at a detection wavelength of 220 nm, with water (with 0.1% formic acid, as phase A) and acetonitrile (as phase B) as the eluents at a flow rate of 1 mL/min, with an injection volume of 5 µL. The gradient condition was: 0→3 min, 100% A; 3→25 min, 100% A to 96% A; 25→26 min, 96% A to 100% A. The ratio of peak areas of lactic acid (after to before UV irradiation) was 0.91 (Figure S63), indicating that it remained largely stable with no significant degradation under the applied UV conditions.

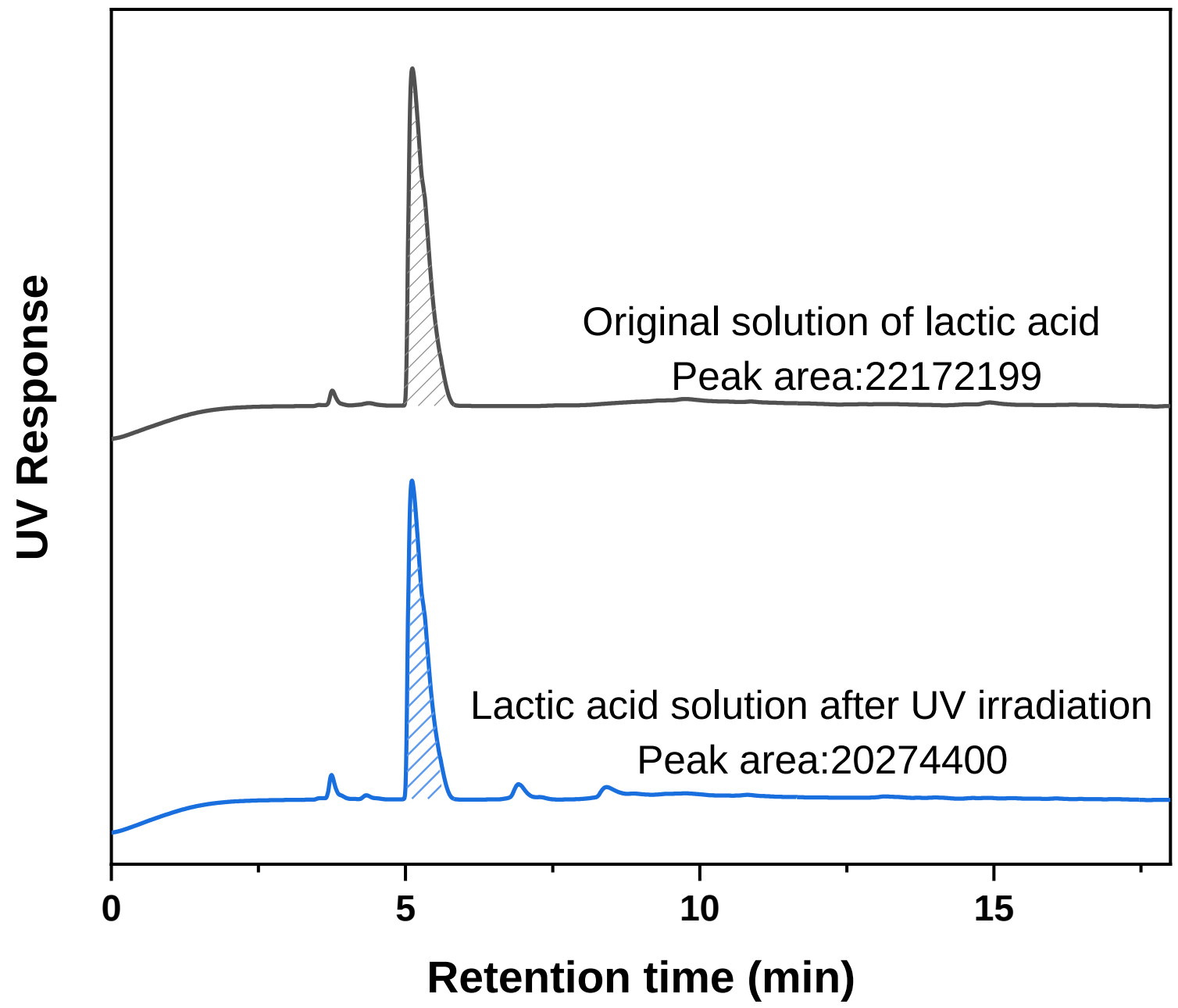

Figure S63. HPLC chromatograms and peak areas of lactic acid before and after irradiation.

UV-protective performance of aggregatable molecules for synthetic polymeric materials:

For polypropylene (PP): Two experiments were conducted in parallel: identical polypropylene (PP) specimens were separately placed horizontally in two clean Petri dishes (dumbbell-shaped plastic specimens with the total length of 150 mm and a parallel section of 60.00 × 10.20 × 2.00 mm, see Figure S64). One Petri dish was added with 40 mL of water to ensure that the specimen was completely submerged (free of attached air bubbles). Separately, 40 mL of 3 M aqueous solution of lactic acid (or 0.6 M aqueous solution of lysine) was added to the other Petri dish, with the same requirement of complete submersion of the specimen guaranteed. Both petri dishes were placed in the identical UV irradiation apparatus employed for the stability test of lactic acid. After continuous irradiation for 24 h, the specimens were flipped over (to avoid uneven UV exposure on a single side) and subjected to further irradiation for an additional 24 h, leading to a cumulative UV irradiation duration of 48 h. Upon completion of irradiation, the specimens were retrieved for mechanical property tests to evaluate the UV-protective efficacy of lactic acid/lysine. Tensile tests of all specimens were performed using a universal material testing machine (see General Information) at a strain rate of 50 mm/min, and the stress-strain curves are shown in Figure 9C in the main text.

For polycarbonate (PC): Two experiments were conducted in parallel: identical polycarbonate (PC) specimens were separately placed horizontally in two clean Petri dishes (dumbbell-shaped plastic specimens with the total length of 150 mm and a parallel section of 60.00 × 10.20 × 2.00 mm, see Figure S64). One Petri dish was added with 40 mL of water to ensure that the specimen was completely submerged (free of attached air bubbles). Separately, 40 mL of 3 M aqueous solution of lactic acid (or 0.6 M aqueous solution of lysine) was added to the other Petri dish, with the same requirement of complete submersion of the specimen guaranteed. Both petri

dishes were placed in the identical UV irradiation apparatus employed for the stability test of lactic acid. After continuous irradiation for 24 h, the specimens were flipped over (to avoid uneven UV exposure on a single side) and subjected to further irradiation for an additional 24 h, leading to a cumulative UV irradiation duration of 48 h. Upon completion of irradiation, the specimens were retrieved for mechanical property tests to evaluate the UV-protective efficacy of lactic acid/lysine. Tensile tests of all specimens were performed using a universal material testing machine (see General Information) at a strain rate of 5 mm/min, and the stress-strain curves are shown in Figure 9D in the main text.

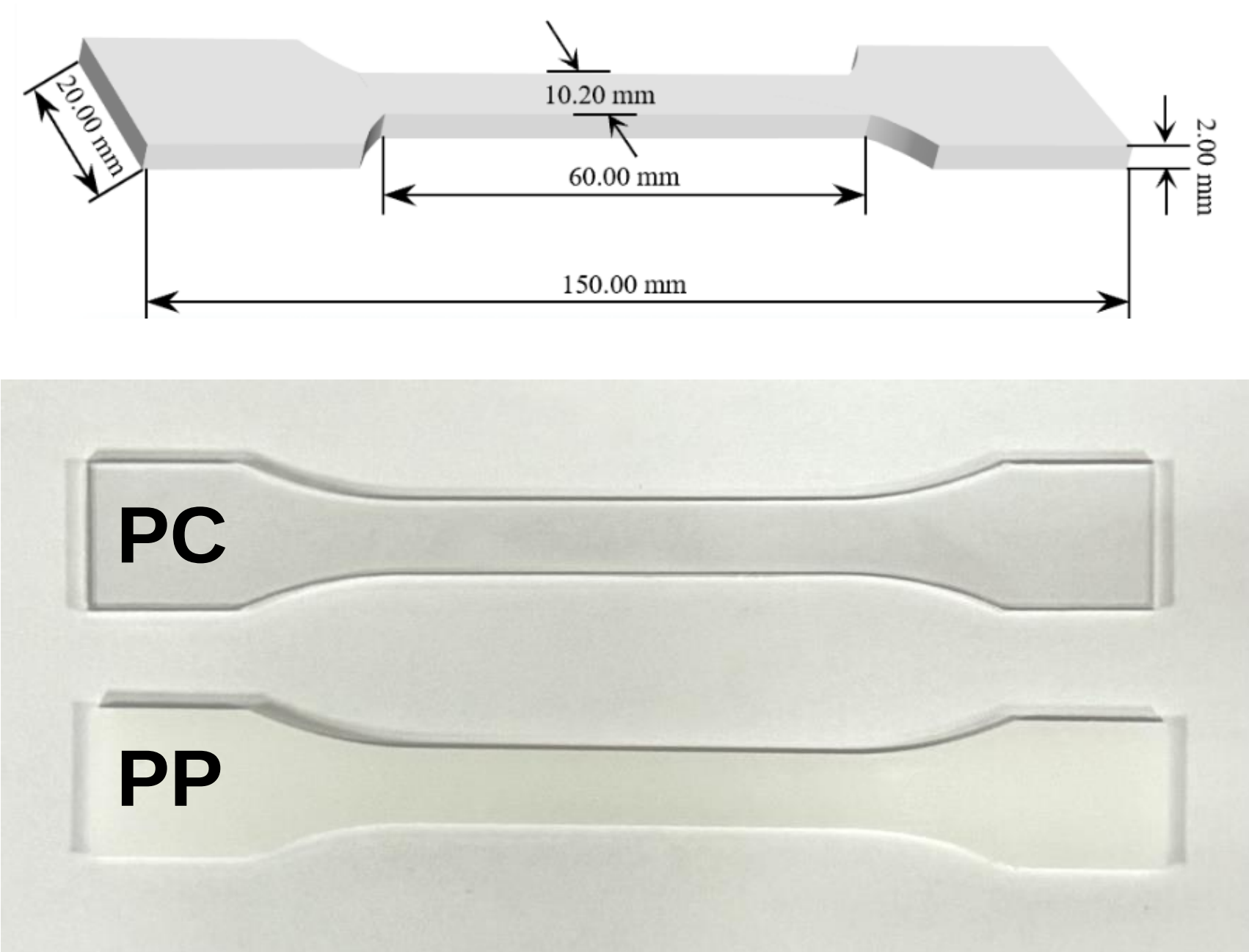

Figure S64. Specific dimensions and photos of the PP and PC specimens.

Photoprotection of polypropylene sheet by spraying aqueous lactic acid solution:

To evaluate the efficacy of photoprotection by lactic acid, PP sheets (20 mm × 10 mm × 0.5 mm) were sprayed either with pure water, or with a lactic acid solution (3 M in water, after 10 min of ultrasonic homogenization). Both sheets were then irradiated for 24 h under a dual low-pressure Hg lamp set (λ = 254 nm), with the lamps positioned above to ensure uniform UV exposure.

To prevent water evaporation, the control PP sample were periodically sprayed with water to prevent full evaporation over the irradiation period. Meanwhile, the PP sample protected with lactic acid were periodically sprayed with fresh 3 M aqueous solution of lactic acid (approximately every 4 h), which continuously washed off and replaced the old solution and kept the plastic surface constantly wet to prevent full evaporation of water.

After UV irradiation, the unprotected PP sheet exhibited brittleness and developed cracks upon bending (see Supplementary Video 1). In contrast, the lactic acid-protected sheet maintained

its flexibility even when bent to approximately 180°, demonstrating that lactic acid can effectively mitigate UV-induced aging (see Supplementary Video 2).

DFT calculation of the geometries and excited states of DAP monomer and aggregates in water:

Density functional theory (DFT) calculations were performed with Gaussian 16, Revision A.03.[5] The geometries were optimized using the CAM-B3LYP density functional, with 6-31+G(d,p) as the basis set. Excited states (*n* = 10) were calculated with TD-SCF method, with CAM-B3LYP and 6-311++G(d,p) for enhanced accuracy. Solvation effect in water was considered (PCM). The optimized geometries, Cartesian coordinates, absorption wavelengths and oscillator strengths of DAP monomer and aggregates are listed below.

DAP Monomer:

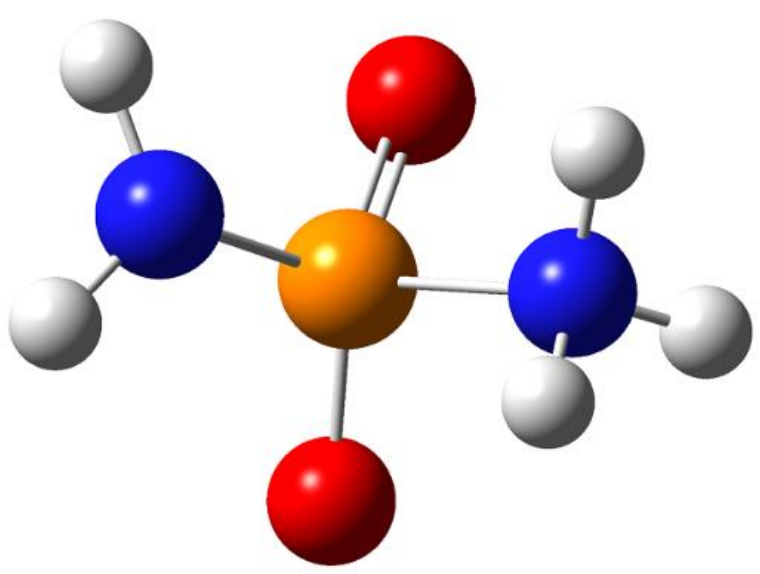

Center Number	Atomic Number	Atomic Type	Coordinates (Angstroms) X	Y	Z
1	15	0	-0.161697	-0.214144	0.000719
2	8	0	-0.137696	-0.899775	-1.325725
3	8	0	-0.137391	-0.890178	1.332078
4	7	0	-1.162689	1.102181	-0.003780
5	7	0	1.505411	0.660292	-0.002458
6	1	0	2.261131	-0.027386	0.000101
7	1	0	1.620958	1.245706	-0.831395
8	1	0	-1.638189	1.359364	-0.857270
9	1	0	-1.637565	1.365179	0.848291
10	1	0	1.620770	1.251606	0.822335

Excitation energies and oscillator strengths:

```
Excited State   1:      Singlet-A      6.8577 eV  180.80 nm  f=0.0297  <S**2>=0.000
     25 -> 26         0.64834
     25 -> 27        -0.19937
This state for optimization and/or second-order correction.
Total Energy, E(TD-HF/TD-DFT) =  -604.182640496
Copying the excited state density for this state as the 1-particle RhoCI density.

Excited State   2:      Singlet-A      7.0437 eV  176.02 nm  f=0.0260  <S**2>=0.000
     24 -> 26         0.65667
     24 -> 29        -0.16468

Excited State   3:      Singlet-A      7.3836 eV  167.92 nm  f=0.0004  <S**2>=0.000
     23 -> 26         0.63679
     23 -> 29        -0.18435
     25 -> 28         0.12162

Excited State   4:      Singlet-A      7.8255 eV  158.44 nm  f=0.1278  <S**2>=0.000
     21 -> 26        -0.24836
     22 -> 26        -0.34712
     23 -> 28         0.10815
     25 -> 26         0.15685
     25 -> 27         0.46003
     25 -> 29        -0.11069
     25 -> 30        -0.12744

Excited State   5:      Singlet-A      7.9829 eV  155.31 nm  f=0.1032  <S**2>=0.000
     21 -> 26        -0.27838
     21 -> 29         0.10034
     22 -> 26         0.49209
     22 -> 29        -0.10640
     24 -> 28         0.26126
     24 -> 32         0.12198
     25 -> 27         0.18111

Excited State   6:      Singlet-A      8.1829 eV  151.52 nm  f=0.0628  <S**2>=0.000
     21 -> 26         0.38180
     22 -> 26         0.18307
     24 -> 28        -0.22031
     25 -> 26         0.10688
     25 -> 27         0.39328
     25 -> 29         0.21004

Excited State   7:      Singlet-A      8.2016 eV  151.17 nm  f=0.0012  <S**2>=0.000
     24 -> 27         0.61733
     24 -> 29         0.16543
```

```
     24 -> 30         0.16222
     25 -> 28         0.12696

Excited State   8:      Singlet-A      8.2105 eV  151.01 nm  f=0.0004  <S**2>=0.000
     24 -> 27        -0.12181
     25 -> 28         0.60835
     25 -> 31         0.21643

Excited State   9:      Singlet-A      8.4460 eV  146.80 nm  f=0.0005  <S**2>=0.000
     23 -> 27         0.15729
     24 -> 26         0.15609
     24 -> 27        -0.20202
     24 -> 29         0.58796
     24 -> 30         0.10322
     24 -> 34        -0.14485

Excited State  10:      Singlet-A      8.4953 eV  145.94 nm  f=0.0014  <S**2>=0.000
     22 -> 26        -0.19765
     24 -> 28         0.27090
     25 -> 29         0.55346
     25 -> 30         0.16785
```

DAP Dimer (Hydrogen-bonded, "Equatorial-Equatorial"):

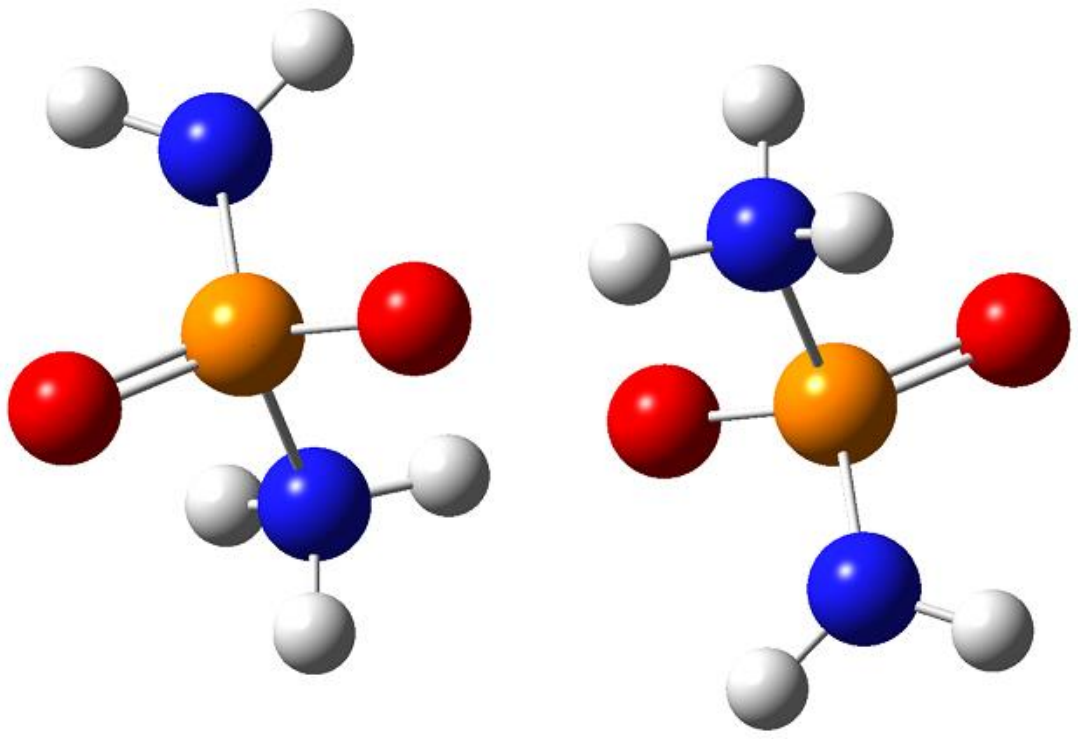

Center Number	Atomic Number	Atomic Type	Coordinates (Angstroms) X	Y	Z
1	15	0	2.237424	-0.057945	-0.191194
2	8	0	1.173922	-0.491023	-1.167360
3	7	0	2.420108	-1.174733	1.012822

4	15	0	-2.237522	0.057566	0.191243
5	8	0	-1.174142	0.483716	1.170637
6	7	0	-2.413605	1.178604	-1.010117
7	1	0	3.326255	-1.302125	1.440594
8	1	0	1.804268	-1.974101	1.057954
9	1	0	-1.796226	1.977143	-1.049219
10	1	0	-3.319440	1.311818	-1.436854
11	8	0	-3.538572	-0.552202	0.593890
12	8	0	3.535377	0.555569	-0.598149
13	7	0	-1.381651	-1.294628	-0.720484
14	1	0	-0.388539	-1.017890	-0.943846
15	1	0	-1.354116	-2.140011	-0.147144
16	7	0	1.378626	1.294365	0.718213
17	1	0	1.346957	2.137693	0.142070
18	1	0	0.386954	1.015214	0.944789
19	1	0	-1.870757	-1.527754	-1.585882
20	1	0	1.869081	1.531968	1.581638

Excitation energies and oscillator strengths:

Excited State 1: Singlet-A 6.9491 eV 178.42 nm f=0.0000 <S**2>=0.000
48 -> 51 0.12769
49 -> 52 -0.30427
49 -> 53 -0.15686
50 -> 51 0.55105
50 -> 64 -0.10843
This state for optimization and/or second-order correction.
Total Energy, E(TD-HF/TD-DFT) = -1208.63964312
Copying the excited state density for this state as the 1-particle RhoCI density.

Excited State 2: Singlet-A 6.9780 eV 177.68 nm f=0.0487 <S**2>=0.000
49 -> 51 0.54376
49 -> 64 -0.10531
50 -> 52 -0.31645
50 -> 53 -0.16135

Excited State 3: Singlet-A 7.2762 eV 170.40 nm f=0.0000 <S**2>=0.000
47 -> 52 -0.20264
47 -> 54 0.13575
48 -> 51 0.58723
48 -> 64 -0.10052
50 -> 51 -0.16140

```
Excited State   4:      Singlet-A      7.3673 eV  168.29 nm  f=0.0705  <S**2>=0.000
     47 -> 51         0.55983
     48 -> 52        -0.25813
     48 -> 54         0.15114
     49 -> 51         0.13062
     50 -> 52         0.10023

Excited State   5:      Singlet-A      7.5882 eV  163.39 nm  f=0.0000  <S**2>=0.000
     45 -> 52        -0.21950
     45 -> 54         0.13890
     46 -> 51         0.52846
     46 -> 57        -0.10688
     47 -> 52         0.11441
     49 -> 52         0.17978
     49 -> 53        -0.14829

Excited State   6:      Singlet-A      7.6149 eV  162.82 nm  f=0.0387  <S**2>=0.000
     45 -> 51         0.52119
     46 -> 52        -0.23936
     46 -> 54         0.14374
     48 -> 52        -0.14167
     50 -> 52         0.12727
     50 -> 53        -0.17719

Excited State   7:      Singlet-A      7.9572 eV  155.81 nm  f=0.0000  <S**2>=0.000
     41 -> 51        -0.13379
     43 -> 51         0.33850
     46 -> 51        -0.15529
     47 -> 52         0.16148
     49 -> 53        -0.33617
     50 -> 51        -0.20628
     50 -> 55        -0.24971

Excited State   8:      Singlet-A      7.9908 eV  155.16 nm  f=0.1644  <S**2>=0.000
     42 -> 51        -0.11478
     43 -> 52         0.10494
     44 -> 51         0.34729
     45 -> 51         0.23099
     48 -> 52         0.15896
     49 -> 51         0.17448
     49 -> 55         0.20508
     50 -> 53         0.33938
     50 -> 54         0.10106

Excited State   9:      Singlet-A      8.0655 eV  153.72 nm  f=0.0679  <S**2>=0.000
```

```
      42 -> 51          0.14616
      44 -> 51          0.23529
      47 -> 51          0.14209
      48 -> 52          0.30546
      48 -> 53         -0.20632
      49 -> 56         -0.22730
      50 -> 52          0.19859
      50 -> 53         -0.29494

 Excited State  10:      Singlet-A      8.1261 eV  152.58 nm  f=0.0002  <S**2>=0.000
      45 -> 52          0.11286
      46 -> 51         -0.13485
      47 -> 52         -0.16055
      49 -> 52          0.35127
      49 -> 53         -0.28675
      49 -> 59         -0.14675
      50 -> 51          0.12955
      50 -> 55          0.17343
      50 -> 56         -0.28435
```

DAP Dimer (Hydrogen-bonded, "Axial-Axial"):

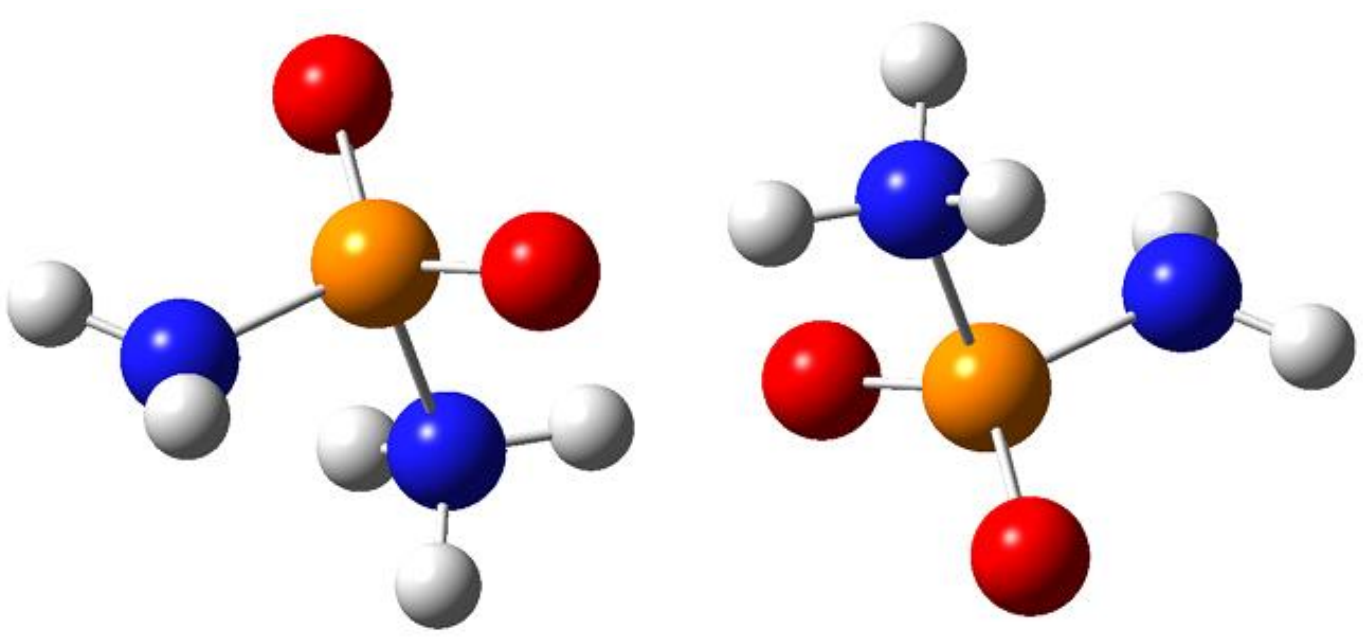

Center Number	Atomic Number	Atomic Type	Coordinates (Angstroms) X	Y	Z
1	15	0	2.189087	0.134670	-0.151806
2	8	0	1.216916	1.203656	0.279890
3	15	0	-2.188870	-0.134913	0.151509
4	8	0	-1.216765	-1.202733	-0.283233
5	7	0	-1.376545	1.387140	-0.488633
6	1	0	-0.364006	1.391045	-0.185275
7	1	0	-1.398789	1.421860	-1.509017

8	7	0	1.375455	-1.386124	0.489707
9	1	0	1.395943	-1.418862	1.510195
10	1	0	0.363444	-1.390506	0.184656
11	1	0	-1.838827	2.227094	-0.137236
12	1	0	1.838299	-2.226787	0.140754
13	8	0	2.476488	-0.212042	-1.575167
14	8	0	-2.474404	0.209427	1.575842
15	7	0	3.558187	0.201583	0.772177
16	1	0	4.444720	-0.037331	0.350587
17	1	0	3.610265	0.867072	1.530799
18	7	0	-3.559221	-0.200975	-0.770614
19	1	0	-3.612044	-0.864923	-1.530527
20	1	0	-4.445274	0.037163	-0.347593

```
---------------------------------------------------------------------

Excitation energies and oscillator strengths:

 Excited State   1:      Singlet-A      6.9393 eV  178.67 nm  f=0.0000  <S**2>=0.000
      48 -> 52         0.10452
      49 -> 52        -0.36535
      50 -> 51         0.53896
      50 -> 55        -0.11291
 This state for optimization and/or second-order correction.
 Total Energy, E(TD-HF/TD-DFT) =  -1208.64051800
 Copying the excited state density for this state as the 1-particle RhoCI density.

 Excited State   2:      Singlet-A      6.9663 eV  177.98 nm  f=0.0602  <S**2>=0.000
      48 -> 51        -0.10797
      49 -> 51         0.49423
      49 -> 55        -0.10830
      50 -> 52        -0.41946

 Excited State   3:      Singlet-A      7.3099 eV  169.61 nm  f=0.0559  <S**2>=0.000
      47 -> 52         0.24411
      47 -> 54        -0.11809
      48 -> 51         0.57499
      49 -> 51         0.16168

 Excited State   4:      Singlet-A      7.3463 eV  168.77 nm  f=0.0000  <S**2>=0.000
      47 -> 51         0.58404
      48 -> 52         0.25381
      48 -> 54        -0.11842
      48 -> 59        -0.10117
      49 -> 52         0.10007
```

```
Excited State   5:      Singlet-A      7.6460 eV  162.16 nm  f=0.0000  <S**2>=0.000
     45 -> 52         0.21557
     45 -> 54        -0.10401
     46 -> 51         0.56152
     46 -> 60        -0.10386
     49 -> 54         0.15345

Excited State   6:      Singlet-A      7.6751 eV  161.54 nm  f=0.0268  <S**2>=0.000
     45 -> 51         0.52842
     46 -> 52         0.24921
     46 -> 54        -0.13599
     46 -> 59        -0.10478
     50 -> 53        -0.11655
     50 -> 54        -0.12928

Excited State   7:      Singlet-A      7.9662 eV  155.64 nm  f=0.2272  <S**2>=0.000
     43 -> 51         0.36564
     45 -> 51        -0.22814
     48 -> 51         0.11758
     49 -> 51        -0.25025
     49 -> 55        -0.27430
     50 -> 52        -0.21678
     50 -> 59        -0.11438

Excited State   8:      Singlet-A      7.9906 eV  155.16 nm  f=0.0000  <S**2>=0.000
     41 -> 51         0.19655
     49 -> 52         0.21528
     49 -> 53         0.18993
     49 -> 59         0.12864
     50 -> 51         0.30314
     50 -> 55         0.42935

Excited State   9:      Singlet-A      8.1017 eV  153.03 nm  f=0.0000  <S**2>=0.000
     42 -> 52        -0.10820
     43 -> 52        -0.12790
     44 -> 51         0.53217
     47 -> 51         0.10322
     47 -> 56        -0.10177
     48 -> 52        -0.16983
     48 -> 53         0.13341
     48 -> 54         0.14492
     49 -> 53         0.10160

Excited State  10:      Singlet-A      8.2291 eV  150.66 nm  f=0.0211  <S**2>=0.000
     43 -> 51         0.16043
```

49	-> 55	0.19843
49	-> 56	0.23047
49	-> 57	0.10143
50	-> 52	-0.13454
50	-> 53	0.50122
50	-> 61	-0.11329

DAP Dimer (Hydrogen-bonded, "Equatorial-Axial"):

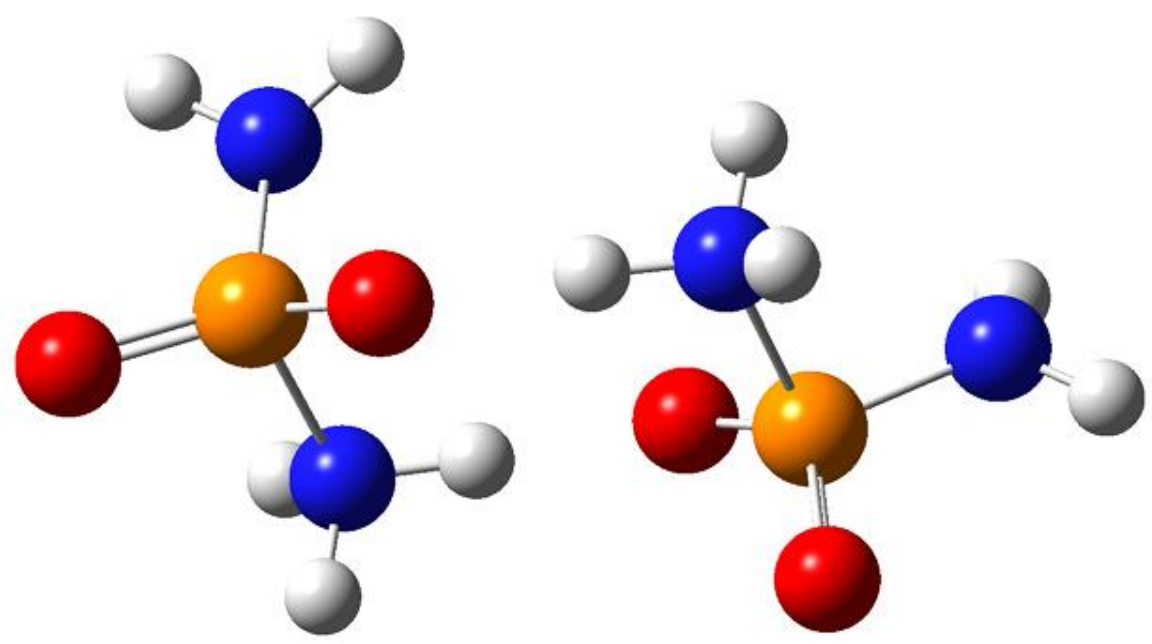

Center Number	Atomic Number	Atomic Type	Coordinates (Angstroms) X	Y	Z
1	15	0	2.172785	-0.202116	0.016419
2	8	0	1.159397	-0.778993	-0.939632
3	15	0	-2.263818	0.108671	0.143803
4	8	0	-1.230798	0.778478	1.013159
5	7	0	-1.399303	-1.446230	-0.328693
6	1	0	-0.399887	-1.242681	-0.603367
7	1	0	-1.385255	-2.092746	0.462492
8	7	0	1.364950	1.381605	0.498379
9	1	0	1.378619	2.056612	-0.267898
10	1	0	0.356740	1.195153	0.748924
11	1	0	-1.874837	-1.916825	-1.099974
12	1	0	1.838668	1.806892	1.296657
13	8	0	2.528042	-0.812633	1.331466
14	7	0	3.498114	0.333809	-0.812611
15	1	0	4.406876	0.277513	-0.374899
16	1	0	3.503657	0.285662	-1.821879
17	7	0	-2.375893	0.858586	-1.324676
18	1	0	-3.265976	0.887180	-1.801297
19	1	0	-1.734578	1.601964	-1.562324
20	8	0	-3.590842	-0.355783	0.644188

```
 ---------------------------------------------------------------------

 Excitation energies and oscillator strengths:

 Excited State   1:      Singlet-A      6.9450 eV  178.52 nm  f=0.0239  <S**2>=0.000
      47 -> 51        -0.11552
      47 -> 52         0.12101
      49 -> 51        -0.17818
      49 -> 52         0.21034
      50 -> 51         0.49896
      50 -> 52        -0.28255
 This state for optimization and/or second-order correction.
 Total Energy, E(TD-HF/TD-DFT) =  -1208.63998808
 Copying the excited state density for this state as the 1-particle RhoCI density.

 Excited State   2:      Singlet-A      6.9639 eV  178.04 nm  f=0.0248  <S**2>=0.000
      49 -> 51         0.51599
      49 -> 52         0.23645
      49 -> 53        -0.15068
      50 -> 51         0.19994
      50 -> 52         0.20220

 Excited State   3:      Singlet-A      7.3222 eV  169.33 nm  f=0.0246  <S**2>=0.000
      48 -> 51         0.58678
      48 -> 52        -0.21505
      48 -> 53         0.13178
      48 -> 57        -0.12941
      49 -> 51         0.10232

 Excited State   4:      Singlet-A      7.3380 eV  168.96 nm  f=0.0414  <S**2>=0.000
      47 -> 51         0.53456
      47 -> 52         0.26410
      49 -> 52        -0.10013
      50 -> 51         0.15724
      50 -> 52         0.13997

 Excited State   5:      Singlet-A      7.5846 eV  163.47 nm  f=0.0116  <S**2>=0.000
      45 -> 52        -0.19795
      46 -> 51         0.52415
      46 -> 52         0.17434
      49 -> 53        -0.13198
      50 -> 52        -0.11226

 Excited State   6:      Singlet-A      7.6681 eV  161.69 nm  f=0.0236  <S**2>=0.000
      45 -> 51         0.51092
```

```
      46 -> 51         0.15366
      46 -> 52        -0.22027
      46 -> 57        -0.10364
      50 -> 54         0.14494

 Excited State   7:      Singlet-A      7.9478 eV  156.00 nm  f=0.1592  <S**2>=0.000
      43 -> 51        -0.17089
      44 -> 51         0.26092
      45 -> 51         0.19078
      47 -> 51         0.13842
      49 -> 52         0.14032
      49 -> 53        -0.14830
      50 -> 51        -0.23510
      50 -> 52        -0.17351
      50 -> 54        -0.21687
      50 -> 55        -0.20334

 Excited State   8:      Singlet-A      8.0061 eV  154.86 nm  f=0.0106  <S**2>=0.000
      42 -> 51         0.11133
      43 -> 51         0.28575
      44 -> 51         0.21756
      46 -> 51        -0.12299
      47 -> 51        -0.10763
      49 -> 51        -0.16947
      49 -> 52        -0.10111
      49 -> 53        -0.25773
      49 -> 54        -0.17404
      49 -> 55        -0.11340
      49 -> 57        -0.10264
      50 -> 52         0.10050
      50 -> 53        -0.18416

 Excited State   9:      Singlet-A      8.0913 eV  153.23 nm  f=0.0042  <S**2>=0.000
      43 -> 51         0.11895
      46 -> 52         0.11481
      48 -> 52        -0.12118
      49 -> 51        -0.13597
      49 -> 52         0.31919
      49 -> 53         0.22541
      49 -> 55        -0.23085
      49 -> 56        -0.11867
      49 -> 59        -0.10184
      50 -> 52         0.21009
      50 -> 53         0.13176
      50 -> 55        -0.11409
```

```
Excited State  10:      Singlet-A      8.1112 eV  152.86 nm  f=0.0806  <S**2>=0.000
      42 -> 51         0.14120
      43 -> 51         0.21931
      44 -> 51        -0.21416
      47 -> 51        -0.11309
      47 -> 52         0.28972
      49 -> 53        -0.12836
      49 -> 54         0.13727
      49 -> 55         0.13268
      50 -> 51        -0.11839
      50 -> 53         0.21806
      50 -> 54        -0.14374
      50 -> 55        -0.16293
```

DAP Dimer (Stacked):

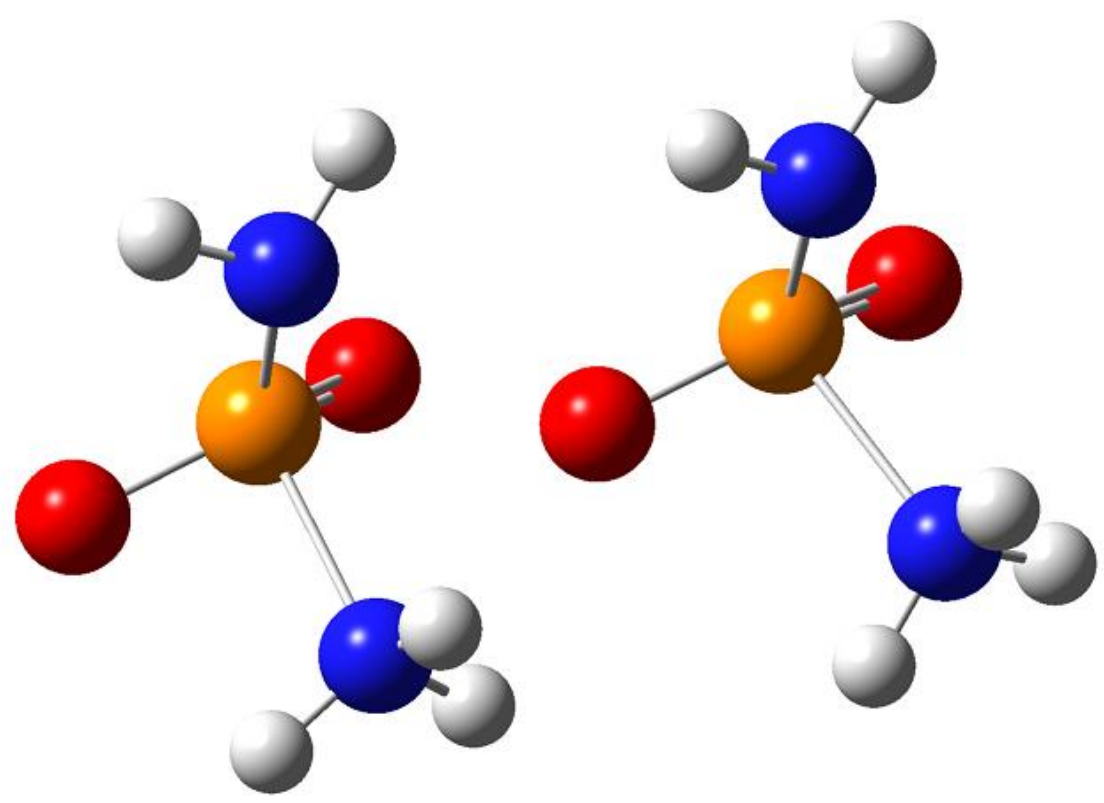

Center Number	Atomic Number	Atomic Type	Coordinates (Angstroms) X	Y	Z
1	15	0	-2.065794	-0.169409	0.247907
2	8	0	-1.381966	-0.306559	1.546679
3	8	0	-3.473577	0.261659	-0.039188
4	7	0	-1.604386	-1.300682	-1.026012
5	7	0	-1.447265	1.575310	-0.548850
6	1	0	-0.884563	1.411204	-1.386874
7	1	0	-2.288112	2.117161	-0.790459
8	1	0	-2.383376	-1.692821	-1.563704
9	1	0	-0.964199	-2.049277	-0.767383
10	1	0	-0.878298	2.086301	0.129658
11	15	0	1.758169	-0.193534	0.233861

```
     12          8           0        2.582676   -0.317507    1.449233
     13          8           0        0.364178    0.227787   -0.048768
     14          7           0        2.316217   -1.289920   -1.001865
     15          7           0        2.727377    1.459228   -0.531961
     16          1           0        3.421053    1.774311    0.158944
     17          1           0        3.224553    1.229267   -1.398326
     18          1           0        1.626312   -1.670984   -1.656182
     19          1           0        2.977692   -2.023259   -0.730762
     20          1           0        2.089228    2.231655   -0.724260
 ---------------------------------------------------------------------

Excitation energies and oscillator strengths:

 Excited State   1:      Singlet-A      6.3337 eV  195.75 nm  f=0.0279  <S**2>=0.000
      49 -> 51         0.17701
      49 -> 52         0.11088
      50 -> 51         0.53959
      50 -> 52         0.30636
 This state for optimization and/or second-order correction.
 Total Energy, E(TD-HF/TD-DFT) =  -1208.57953430
 Copying the excited state density for this state as the 1-particle RhoCI density.

 Excited State   2:      Singlet-A      6.5622 eV  188.94 nm  f=0.0092  <S**2>=0.000
      48 -> 51         0.10295
      49 -> 51         0.55451
      49 -> 52         0.22093
      50 -> 51        -0.20516

 Excited State   3:      Singlet-A      6.6583 eV  186.21 nm  f=0.0515  <S**2>=0.000
      47 -> 51        -0.13283
      48 -> 51         0.56403
      48 -> 52        -0.16725
      48 -> 53        -0.19686
      50 -> 52         0.10974

 Excited State   4:      Singlet-A      7.0064 eV  176.96 nm  f=0.0658  <S**2>=0.000
      47 -> 51         0.48297
      47 -> 52         0.32843
      48 -> 51         0.12333
      48 -> 52         0.15558
      49 -> 52         0.10177
      50 -> 53         0.12422

 Excited State   5:      Singlet-A      7.2096 eV  171.97 nm  f=0.0888  <S**2>=0.000
      46 -> 51         0.43237
```

```
      46 -> 52         0.12830
      49 -> 51        -0.12387
      49 -> 52         0.30890
      50 -> 52        -0.19065
      50 -> 53        -0.11200
      50 -> 56        -0.10089

 Excited State   6:      Singlet-A      7.3295 eV  169.16 nm  f=0.0029  <S**2>=0.000
      42 -> 51         0.10322
      43 -> 51        -0.22425
      45 -> 51         0.20283
      47 -> 52        -0.12077
      50 -> 51        -0.24689
      50 -> 52         0.35948
      50 -> 53        -0.14482
      50 -> 55        -0.18903
      50 -> 58         0.10374

 Excited State   7:      Singlet-A      7.5104 eV  165.08 nm  f=0.0268  <S**2>=0.000
      45 -> 51         0.31686
      45 -> 54        -0.10712
      46 -> 52        -0.14322
      47 -> 52        -0.11314
      49 -> 52         0.17957
      49 -> 53         0.13139
      50 -> 53         0.38309
      50 -> 55         0.12876

 Excited State   8:      Singlet-A      7.6215 eV  162.68 nm  f=0.0371  <S**2>=0.000
      45 -> 51        -0.19509
      46 -> 51         0.24009
      46 -> 52         0.24702
      49 -> 53         0.12057
      50 -> 52         0.23054
      50 -> 53         0.29718
      50 -> 60         0.10878

 Excited State   9:      Singlet-A      7.6442 eV  162.19 nm  f=0.0904  <S**2>=0.000
      43 -> 51         0.16799
      44 -> 51         0.25972
      44 -> 52        -0.13165
      48 -> 53         0.12381
      49 -> 53         0.38105
      50 -> 53        -0.17464
      50 -> 65         0.10986
```

```
Excited State  10:      Singlet-A      7.6754 eV  161.53 nm  f=0.0693  <S**2>=0.000
      43 -> 51         0.10961
      44 -> 51         0.36538
      44 -> 52        -0.11206
      44 -> 54        -0.10130
      46 -> 52        -0.12823
      49 -> 52         0.11268
      49 -> 53        -0.25535
      49 -> 55        -0.13353
      50 -> 53         0.12181
      50 -> 54         0.10902
      50 -> 57         0.10870
```

DAP Trimer (Stacked):

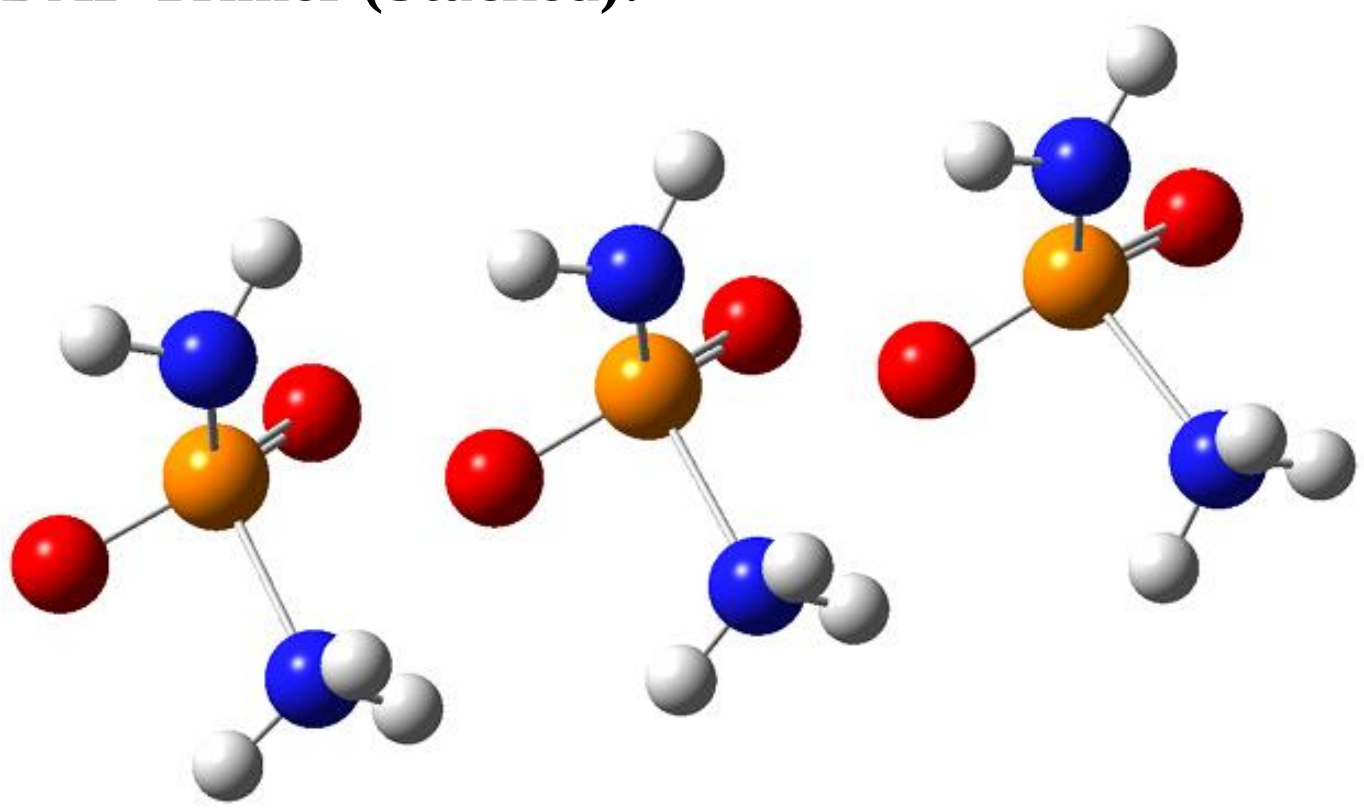

Center Number	Atomic Number	Atomic Type	Coordinates (Angstroms) X	Y	Z
1	15	0	-3.939199	-0.193941	0.229312
2	8	0	-3.303047	-0.376790	1.549166
3	8	0	-5.363889	0.198364	-0.049538
4	7	0	-3.464464	-1.343210	-1.032224
5	7	0	-3.377431	1.563363	-0.529422
6	1	0	-2.803116	1.422820	-1.365932
7	1	0	-4.230784	2.088287	-0.765457
8	1	0	-4.248133	-1.774111	-1.531930
9	1	0	-2.813657	-2.072906	-0.743965
10	1	0	-2.820125	2.071963	0.163192
11	15	0	-0.134747	-0.129505	0.251337
12	8	0	0.567995	-0.274736	1.533309

13	8	0	-1.547886	0.300078	-0.053010
14	7	0	0.288411	-1.247339	-1.030603
15	7	0	0.526778	1.628361	-0.519163
16	1	0	1.122200	2.085305	0.178917
17	1	0	1.094228	1.443625	-1.352807
18	1	0	-0.497241	-1.600640	-1.588648
19	1	0	0.922359	-2.010768	-0.802896
20	1	0	-0.258120	2.232259	-0.761355
21	15	0	3.682874	-0.236771	0.234611
22	8	0	4.507606	-0.348398	1.444963
23	8	0	2.301314	0.214731	-0.076877
24	7	0	4.248388	-1.297117	-1.021796
25	7	0	4.650653	1.450669	-0.517753
26	1	0	5.326841	1.772109	0.185869
27	1	0	5.165869	1.229735	-1.374499
28	1	0	3.571057	-1.660462	-1.698162
29	1	0	4.925082	-2.026554	-0.779504
30	1	0	4.006530	2.213499	-0.719086

```
Excitation energies and oscillator strengths:

 Excited State   1:      Singlet-A      6.1399 eV  201.93 nm  f=0.0054  <S**2>=0.000
      73 -> 76         0.10132
      74 -> 77         0.11993
      75 -> 76         0.51765
      75 -> 77        -0.36350
 This state for optimization and/or second-order correction.
 Total Energy, E(TD-HF/TD-DFT) =  -1812.97651392
 Copying the excited state density for this state as the 1-particle RhoCI density.

 Excited State   2:      Singlet-A      6.3134 eV  196.38 nm  f=0.0315  <S**2>=0.000
      72 -> 77        -0.11530
      73 -> 76         0.10448
      74 -> 76         0.46252
      74 -> 77        -0.36955
      75 -> 76         0.14089
      75 -> 79         0.10631

 Excited State   3:      Singlet-A      6.4413 eV  192.48 nm  f=0.0249  <S**2>=0.000
      73 -> 76         0.54214
      73 -> 77        -0.10547
      73 -> 78        -0.14993
      73 -> 79         0.15218
      74 -> 76        -0.10379
```

```
      75 -> 77         0.18043
      75 -> 79         0.10669

 Excited State   4:      Singlet-A      6.6240 eV  187.17 nm  f=0.0268  <S**2>=0.000
      70 -> 76         0.36438
      70 -> 77         0.24627
      70 -> 78        -0.12051
      70 -> 80        -0.10845
      72 -> 76         0.37532
      74 -> 76        -0.10160
      74 -> 77        -0.15595
      75 -> 77        -0.12890

 Excited State   5:      Singlet-A      6.7110 eV  184.75 nm  f=0.0558  <S**2>=0.000
      70 -> 76         0.36557
      70 -> 77         0.19341
      70 -> 78        -0.10325
      71 -> 76         0.13208
      72 -> 76        -0.31857
      72 -> 77         0.11069
      74 -> 77         0.10702
      75 -> 77         0.16030
      75 -> 79         0.12173

 Excited State   6:      Singlet-A      6.9243 eV  179.06 nm  f=0.0765  <S**2>=0.000
      71 -> 76        -0.37014
      71 -> 77         0.38700
      71 -> 79         0.15916
      72 -> 77         0.16483
      73 -> 77         0.13592

 Excited State   7:      Singlet-A      7.0319 eV  176.32 nm  f=0.0353  <S**2>=0.000
      68 -> 76         0.17211
      69 -> 76        -0.25534
      69 -> 77         0.12958
      72 -> 77         0.12248
      74 -> 76        -0.16424
      75 -> 76         0.16211
      75 -> 77         0.13139
      75 -> 78         0.25148
      75 -> 79         0.25748

 Excited State   8:      Singlet-A      7.1290 eV  173.91 nm  f=0.0910  <S**2>=0.000
      68 -> 76         0.31912
      68 -> 77        -0.11200
```

```
      69 -> 76          0.17104
      71 -> 77          0.13020
      72 -> 76         -0.10555
      72 -> 77         -0.22870
      73 -> 77         -0.13178
      74 -> 78         -0.15121
      74 -> 79         -0.11595
      74 -> 80          0.12106
      75 -> 80         -0.12591

 Excited State   9:      Singlet-A      7.2062 eV  172.05 nm  f=0.0192  <S**2>=0.000
      66 -> 76         -0.16197
      69 -> 76          0.13640
      72 -> 77         -0.10825
      73 -> 76          0.11501
      73 -> 77          0.25996
      74 -> 77          0.12219
      75 -> 77          0.15282
      75 -> 78          0.27970
      75 -> 80         -0.19148

 Excited State  10:      Singlet-A      7.2594 eV  170.79 nm  f=0.0069  <S**2>=0.000
      72 -> 76         -0.10246
      72 -> 77          0.14267
      73 -> 76          0.10790
      73 -> 77         -0.16480
      74 -> 78          0.12967
      75 -> 76         -0.19330
      75 -> 77         -0.26850
      75 -> 78          0.35318
      75 -> 80         -0.18533
```

DAP Tetramer (Stacked):

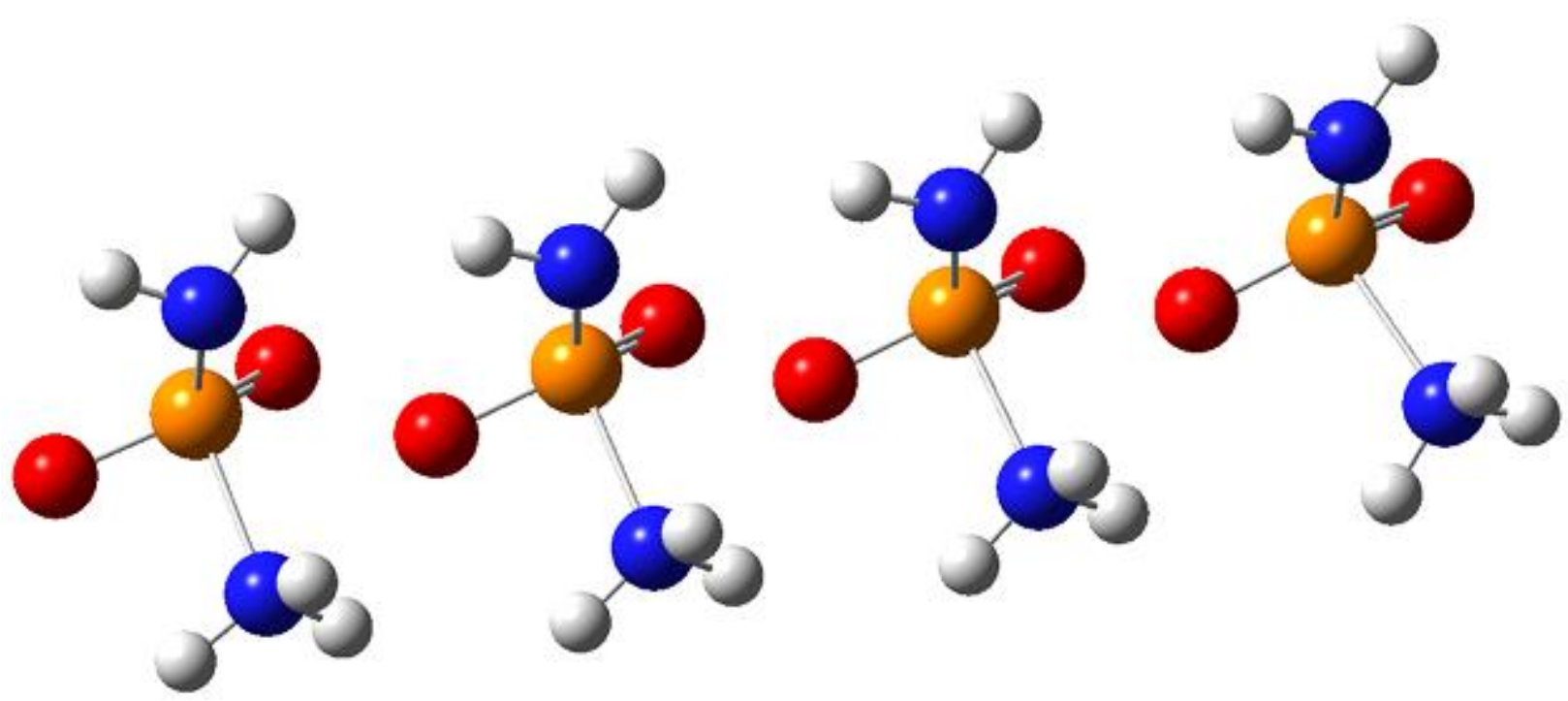

Center Number	Atomic Number	Atomic Type	Coordinates (Angstroms) X	Y	Z
1	15	0	-5.832627	-0.232688	0.222076
2	8	0	-5.218957	-0.407339	1.553260
3	8	0	-7.261368	0.136532	-0.074442
4	7	0	-5.334531	-1.387190	-1.023846
5	7	0	-5.293115	1.531244	-0.540329
6	1	0	-4.710122	1.390842	-1.371255
7	1	0	-6.151466	2.042393	-0.784637
8	1	0	-6.107853	-1.854657	-1.505448
9	1	0	-4.652426	-2.086035	-0.731679
10	1	0	-4.745925	2.048197	0.153956
11	15	0	-2.032072	-0.113893	0.261836
12	8	0	-1.367953	-0.253738	1.563390
13	8	0	-3.455269	0.292987	-0.056315
14	7	0	-1.603190	-1.247486	-1.005081
15	7	0	-1.419771	1.652628	-0.511667
16	1	0	-0.826645	2.115583	0.184867
17	1	0	-0.851243	1.468487	-1.345866
18	1	0	-2.389192	-1.641251	-1.532527
19	1	0	-0.936038	-1.979981	-0.771692
20	1	0	-2.217450	2.239135	-0.750466
21	15	0	1.781260	-0.125979	0.225283
22	8	0	2.468028	-0.251813	1.516721
23	8	0	0.368878	0.300700	-0.096360
24	7	0	2.210423	-1.253552	-1.043539
25	7	0	2.470092	1.620469	-0.542722
26	1	0	3.048253	1.423739	-1.367171
27	1	0	1.702990	2.239485	-0.799211
28	1	0	1.431948	-1.628541	-1.594914
29	1	0	2.868359	-1.997013	-0.818245
30	1	0	3.064072	2.067800	0.164066
31	15	0	5.603959	-0.267590	0.239091
32	8	0	6.413094	-0.364296	1.460779
33	8	0	4.223897	0.179190	-0.089959
34	7	0	6.179724	-1.339070	-1.003112
35	7	0	6.590308	1.407188	-0.509557
36	1	0	7.262455	1.722305	0.201911
37	1	0	7.113701	1.179410	-1.359939
38	1	0	5.510138	-1.708827	-1.683156
39	1	0	6.857711	-2.064042	-0.750711
40	1	0	5.958545	2.177824	-0.719796

```
Excitation energies and oscillator strengths:

 Excited State   1:      Singlet-A      6.0621 eV  204.52 nm  f=0.0018  <S**2>=0.000
      99 ->102         0.12017
      99 ->103        -0.12732
     100 ->101         0.45744
     100 ->102         0.42315
 This state for optimization and/or second-order correction.
 Total Energy, E(TD-HF/TD-DFT) =  -2417.37155227
 Copying the excited state density for this state as the 1-particle RhoCI density.

 Excited State   2:      Singlet-A      6.2275 eV  199.09 nm  f=0.0108  <S**2>=0.000
      97 ->102         0.12949
      97 ->103        -0.11273
      98 ->102        -0.16430
      98 ->103         0.12211
      99 ->101         0.44366
      99 ->102         0.28677
     100 ->101        -0.13316
     100 ->103        -0.17760

 Excited State   3:      Singlet-A      6.3443 eV  195.43 nm  f=0.0558  <S**2>=0.000
      96 ->101         0.14540
      96 ->104        -0.10120
      97 ->101        -0.15473
      98 ->101         0.41072
      98 ->102         0.22557
      98 ->104        -0.10380
      99 ->101         0.16887
      99 ->102        -0.17177
      99 ->103         0.19487

 Excited State   4:      Singlet-A      6.3853 eV  194.17 nm  f=0.0026  <S**2>=0.000
      96 ->101        -0.10812
      96 ->102         0.14605
      97 ->101         0.40904
      97 ->102         0.22494
      97 ->104        -0.11495
      98 ->101         0.22764
      98 ->103         0.11695
      99 ->102         0.11653
     100 ->102        -0.15654
     100 ->104        -0.12223
```

```
Excited State   5:      Singlet-A      6.5335 eV  189.77 nm  f=0.0326  <S**2>=0.000
      96 ->101         0.47208
      96 ->104        -0.14052
      97 ->102         0.23391
      98 ->101        -0.11837
     100 ->103         0.16603

Excited State   6:      Singlet-A      6.6431 eV  186.64 nm  f=0.0365  <S**2>=0.000
      93 ->101         0.37174
      93 ->102        -0.30847
      93 ->103        -0.16595
      93 ->105        -0.13139
      94 ->101        -0.19899
      94 ->102         0.10714
      95 ->101         0.21728
      98 ->102         0.13792

Excited State   7:      Singlet-A      6.7647 eV  183.28 nm  f=0.0759  <S**2>=0.000
      93 ->101        -0.27496
      93 ->102         0.15246
      94 ->101        -0.12881
      95 ->101         0.32242
      96 ->102        -0.10058
      98 ->102         0.21068
      99 ->102         0.16754
     100 ->103        -0.13004

Excited State   8:      Singlet-A      6.8939 eV  179.85 nm  f=0.0757  <S**2>=0.000
      94 ->101         0.25978
      94 ->102         0.30474
      94 ->103        -0.18149
      95 ->101         0.12612
      95 ->102         0.29158
      95 ->103        -0.15677
      98 ->103        -0.11100
     100 ->103         0.10136

Excited State   9:      Singlet-A      6.9784 eV  177.67 nm  f=0.0187  <S**2>=0.000
      92 ->101         0.25427
      92 ->102         0.24515
      94 ->102        -0.10499
      95 ->103         0.11993
      99 ->101         0.13271
      99 ->103         0.15735
      99 ->105        -0.10224
```

```
      99 ->107          0.10485
     100 ->103          0.18731
     100 ->104          0.15963
     100 ->105         -0.20504
     100 ->107          0.12756

 Excited State  10:      Singlet-A      7.0281 eV  176.41 nm  f=0.0147  <S**2>=0.000
      90 ->101          0.25731
      90 ->103          0.11143
      92 ->101          0.18212
      96 ->102          0.15270
      97 ->103         -0.14017
      97 ->104         -0.10080
      98 ->101         -0.13595
      98 ->103         -0.11153
     100 ->102          0.13306
     100 ->103         -0.12341
     100 ->104         -0.25445
     100 ->105          0.15500
     100 ->107          0.12300
```

References

[1] Gibard, C.; Bhowmik, S.; Karki, M.; Kim, E. K.; Krishnamurthy, R. Phosphorylation, oligomerization and self-assembly in water under potential prebiotic conditions. *Nat. Chem.* **2018**, *10*, 212–217.

[2] Yadav, M.; Krishnamurthy, R. Bis(dimethylamino)phosphorodiamidate: A reagent for the regioselective cyclophosphorylation of *cis*-diols enabling one-step access to high-value target cyclophosphates. *Org. Lett.* **2019**, *21*, 7400–7404.

[3] Todd, Z. R.; Szabla, R.; Szostak, J. W.; Sasselov, D. D. UV photostability of three 2-aminoazoles with key roles in prebiotic chemistry on the early earth. *Chem. Commun.* **2019**, *55*, 10388–10391.

[4] Liu, Z.-W.; Wu, L.-F.; Kufner, C. L.; Sasselov, D. D.; Fischer, W. W.; Sutherland, J. D. Prebiotic photoredox synthesis from carbon dioxide and sulfite. *Nat. Chem.* **2021**, *13*, 1126–1132.

[5] Frisch, M. J.; Trucks, G. W.; Schlegel, H. B.; Scuseria, G. E.; Robb, M. A.; Cheeseman, J. R.; Scalmani, G.; Barone, V.; Petersson, G. A.; Nakatsuji, H.; Li, X.; Caricato, M.; Marenich, A. V.; Bloino, J.; Janesko, B. G.; Gomperts, R.; Mennucci, B.; Hratchian, H. P.; Ortiz, J. V.; Izmaylov, A. F.; Sonnenberg, J. L.; Williams-Young, D.; Ding, F.; Lipparini, F.; Egidi, F.; Goings, J.; Peng, B.; Petrone, A.; Henderson, T.; Ranasinghe, D.; Zakrzewski, V. G.; Gao, J.; Rega, N.; Zheng, G.; Liang, W.; Hada, M.; Ehara, M.; Toyota, K.; Fukuda, R.; Hasegawa, J.; Ishida, M.; Nakajima, T.; Honda, Y.; Kitao, O.; Nakai, H.; Vreven, T.; Throssell, K.; Montgomery, J. A., Jr.; Peralta, J. E.; Ogliaro, F.; Bearpark, M. J.; Heyd, J. J.; Brothers, E. N.; Kudin, K. N.; Staroverov, V. N.; Keith, T. A.; Kobayashi, R.; Normand, J.; Raghavachari, K.; Rendell, A. P.; Burant, J. C.; Iyengar, S. S.; Tomasi, J.; Cossi, M.; Millam, J. M.; Klene, M.; Adamo, C.; Cammi,

R.; Ochterski, J. W.; Martin, R. L.; Morokuma, K.; Farkas, O.; Foresman, J. B.; Fox, D. J. Gaussian 16, Revision A.03; Gaussian, Inc.: Wallingford, CT, 2016.
